# Design and evaluation of an amphiphilic biaryl ether library using a novel cell-based β-arrestin recruitment assay for the octopamine receptors of *Balanus improvisus*

**DOI:** 10.1039/d6ra02553f

**Published:** 2026-09-17

**Authors:** Karoline Nordli, Jonne Laurila, Jørn H. Hansen

**Affiliations:** a Department of Chemistry, UiT The Arctic University of Norway, Chemical Synthesis and Analysis Section N-9037 Tromsø Norway jorn.h.hansen@uit.no; b Institute of Biomedicine, Integrative Physiology & Pharmacology, University of Turku FIN-20520 Turku Finland jomila@utu.fi

## Abstract

A 22-compound amphiphilic biaryl ether library inspired by the antifouling compound medetomidine has been designed and synthesized. The compound library was evaluated in a primary screening using a novel β-arrestin assay in CHO cells expressing the BiOctα-octopamine receptor of *Balanus improvisus*. One new compound was found to act as a weak agonist on both BiOctα octopamine receptor and human α_2A_-adrenoceptors.

## Abstract 2

A 22-compound amphiphilic biaryl ether library inspired by the antifouling compound medetomidine has been designed and synthesized.

## Graphical abstract

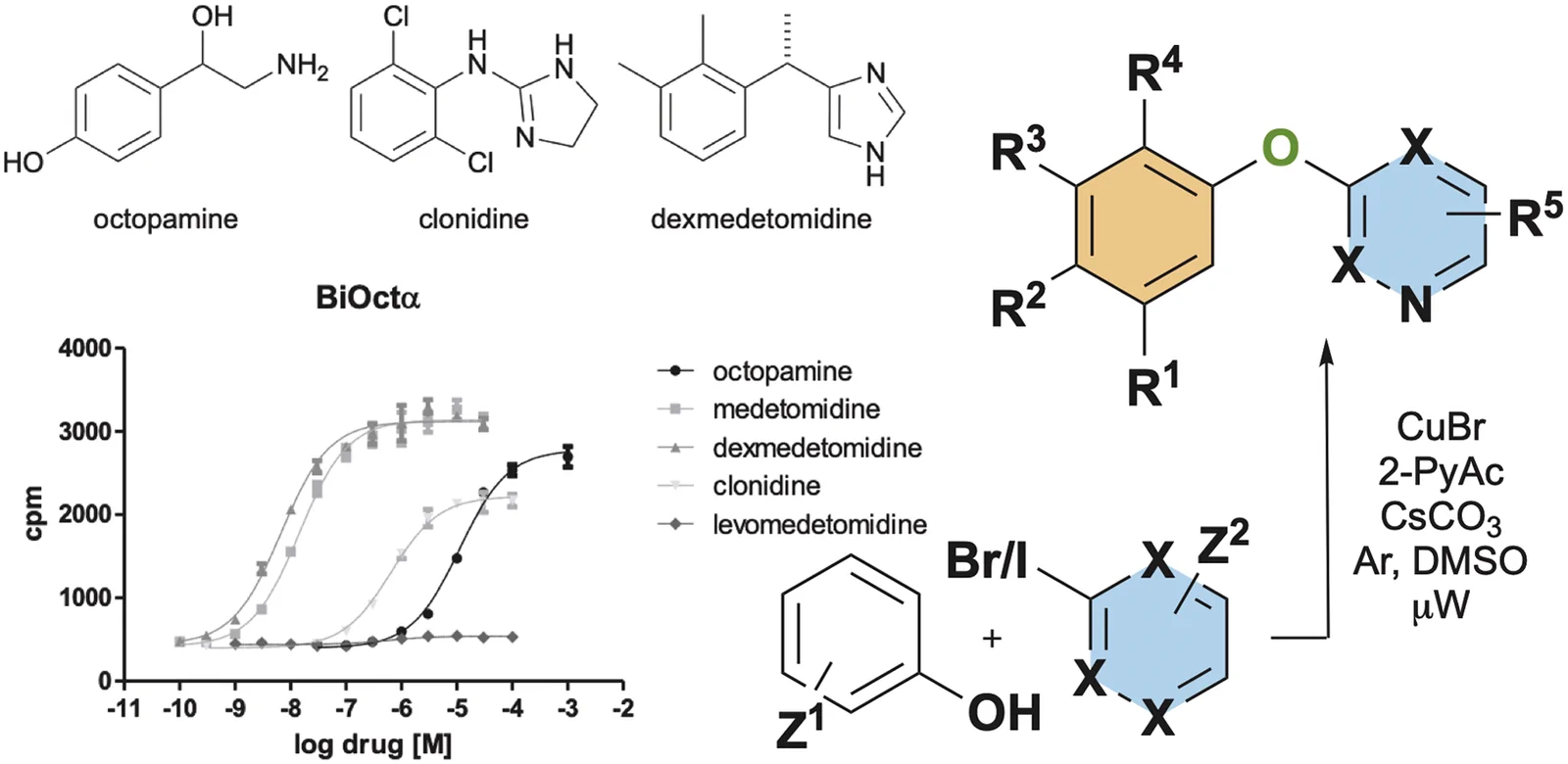

## Introduction

Biofouling, the undesirable accumulation of marine organisms on submerged surfaces such as ship hulls, cooling water systems of power plants, aquaculture installations, and other man-made offshore structures, poses a significant challenge to the maritime industry, including the development of renewable energy technologies.^1^*Balanus improvises*, the barnacle species identified the first time in the Baltic Sea in 1844 as an immigrant species from North America, is a key biofouling target. Especially in the Baltic Sea and nearby regions, *Balanus improvisus* is causing the most severe biofouling problems by settling onto ship hulls and consequently increasing fuel consumption and carbon dioxide emissions.^2^ Previously, antifouling marine paints contained toxic heavy metal additives such as copper and tributyltin (TBT), which have serious negative consequences on the marine environment.^3,4^ The use of TBT has been banned globally,^5^ and there is a great need to develop new environmentally sustainable antifouling agents.^6^

Advances in molecular pharmacology techniques may provide new approaches to this challenge as octopamine receptors of insects and other invertebrates are members of the G protein-coupled receptor (GPCR) superfamily and represent counterparts of the adrenoceptors in vertebrate animals.^7^ Recently, five octopamine receptors have been cloned from the barnacle *Balanus improvisus*, one belonging to the α-adrenoceptor-like subfamily (BiOctα) and four belonging to the β-adrenoceptor-like octopamine receptor subfamily (BiOctβ-R1, BiOctβ-R2, BiOctβ-R3, and BiOctβ-R4).^7^ The α_2_-adrenoreceptor agonist medetomidine ((±)-4[1-(2,3-dimethylphenyl)ethyl]-1*H*-imidazole), known as a sedative agent, has been discovered to inhibit the settling process of barnacles by inducing hyperactivity in the barnacle larvae.^8–10^ The molecular mechanism behind of this action has suggested to be octopamine receptors. Especially the α-adrenoceptor-like BiOctα octopamine receptor has been implicated in the antifouling effect due to its adrenergic-type signaling properties. In recombinant cells, activation of BiOctα by medetomidine has been shown to increase intracellular cAMP and calcium levels.^7^ Recently, the antifouling effect of medetomidine has also led to its commercial use as an environmentally safer alternative to traditional biocides.^11^ Although medetomidine has a non-toxic mechanism of action from an environmental perspective, it may still have significant adverse effects on humans and marine mammals *via* α_2_-adrenoceptor activation. Therefore, medetomidine analogues that selectively activate octopamine receptors in barnacles without affecting vertebrate adrenoceptors may represent safer and more effective antifouling agents.

In this paper, we advance the field of barnacle antifouling research through the design and synthesis of a novel amphiphilic biaryl ether library based on the medetomidine pharmacophore. In addition, for experimental testing of octopamine receptors, we disclose a new bio-assay based on the β-arrestin recruitment in CHO (Chinese Hamster Ovary) cells expressing BiOctα octopamine receptors of *Balanus improvisus*. A signal-to-background ratio of 6–7 was obtained in the β-arrestin assay for BiOctα octopamine receptors using medetomidine, and the EC_50_ values were consistent with previous results from cAMP and calcium-based assays.^7^ In a secondary screening to assess the activity of the synthesised compounds at adrenoceptors, [^35^S]GTPγS-binding assays were performed using CHO cells expressing human α_2A_-adrenoceptors.

## Results and discussion

### Expression of BiOctα receptors in CHO cells

Expression of the BiOctα receptors in transfected CHO cells were characterized primarily using medetomidine and octopamine induced β-arrestin stimulations. The cell line that contained the highest signal-to-background ratio was selected for further studies. From the selected cell line the maximal stimulations (*E*_max_) of medetomidine and octopamine were as follows: medetomidine, 560% over basal; octopamine, 500% over basal. No stimulation was observed in non-transfected cells.††Results not shown. The proper expression of the BiOctα receptor in selected CHO cells was confirmed with a receptor binding assay using the α_2_-adrenoceptor antagonist radioligand [^3^H]RS79948-197. In radioligand based binding assays, CHO cells that were used in the β-arrestin assays were shown to express BiOctα receptors at a density of approximately 3.6 ± 1.1 pmol mg^−1^ membrane protein, which is in the range of the other recombinant GPCRs expressed in CHO cells. The *K*_d_ for [^3^H]RS79948-197 at BiOctα receptor was approximately 6.7 ± 2.9 nM. The *B*_max_ and *K*_d_ values for CHO cell membranes expressing recombinant human α_2A_-adrenoceptors, used as control, were 3.8 ± 0.1 pmol mg^−1^ protein and 0.20 ± 0.01 nM respectively.

### Optimization of the β-arrestin assays

**Fig. 1:**
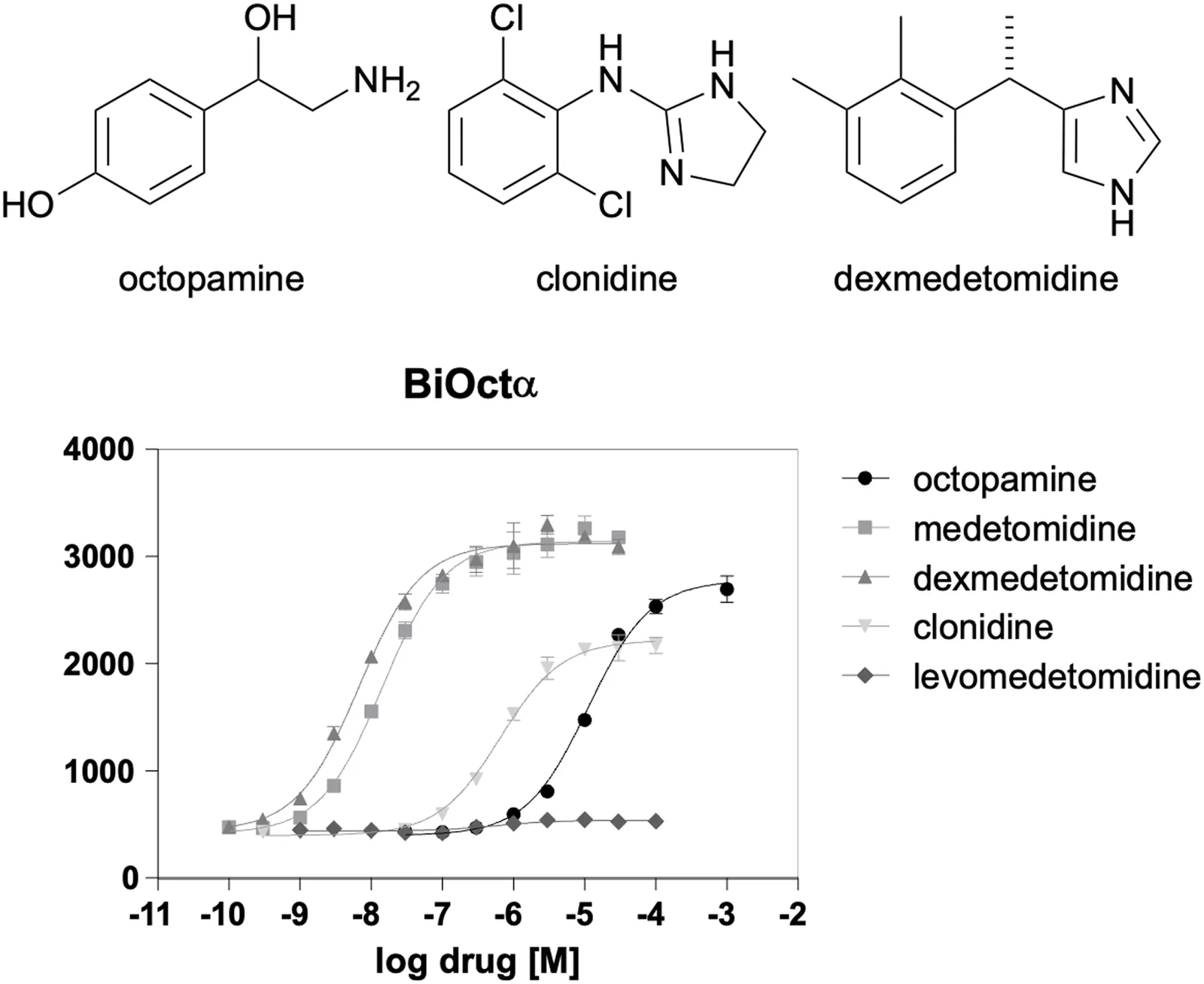
Dose–response confirmation of octopamine, medetomidine, dexmedetomidine, clonidine and levomedetomidine induced β-arrestin stimulation in CHO cells expressing BiOctα receptor of the Balanus improvisus. The error bars represent the s.e.m. of three independent experiments performed in duplicate.

In the PathHunter® β-arrestin assay the signal window is in direct proportion to the number of cells whereas a detection reagent is shattering the cells and causes chemiluminescence. Based on various optimization experiments,† 11 200 cells and 20 µl of detection reagent per well was selected as an optimal quantity in 96-well plate format assays. Thereafter, the activity of clonidine, dexmedetomidine, levomedetomidine and medetomidine to induce β-arrestin stimulations in CHO cells expressing BiOctα receptors of the *Balanus improvisus* were tested using serial dilutions of each compound (Fig. 1). The results show that dexmedetomidine, medetomidine and octopamine have high efficacy, clonidine act as a partial agonist and levomedetomidine induced no stimulation on the BiOctα receptor. The agonist potencies (EC_50_ values) were as follows (mean ± s.e.m): medetomidine 4.4 ± 0.73 nM, octopamine 2800 ± 280 nM, dexmedetomidine 2.6 ± 0.47 nM, and clonidine 74 ± 9.0 nM. These results are in line with the previous calcium release data of BiOctα receptor where medetomidine was shown to be a more potent agonist than octopamine.^7^

### Design and synthesis of a novel compound library

**Fig. 2:**
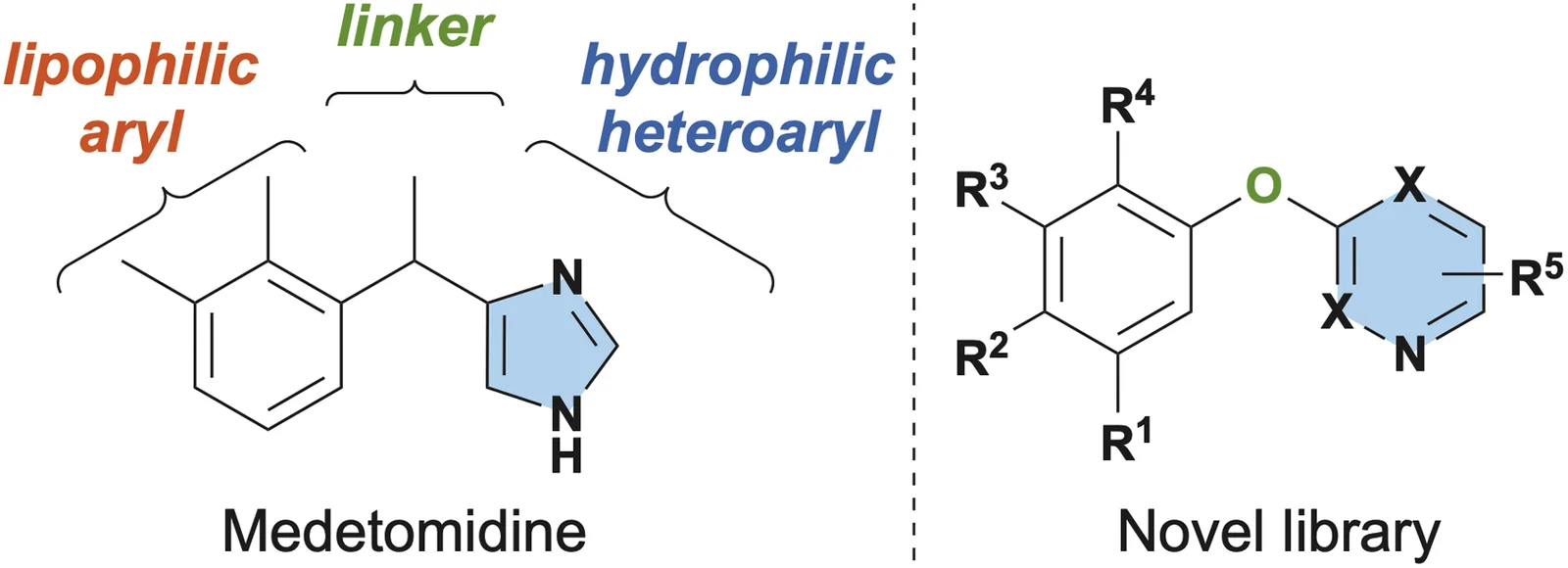
Simplified medetomidine pharmacophore model and the novel library design. Some of the compounds in the library have been reported in the literature, see the SI file for detailed references.

**Fig. 3:**
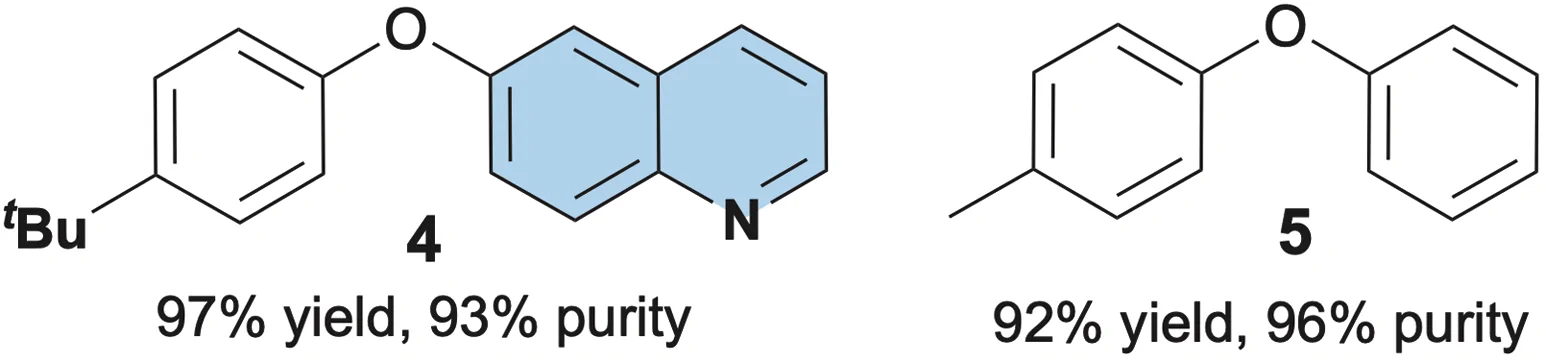
Structures and synthetic results for the only quinoline analogue (4) in the library and a simple, non-amphiphilic reference compound 5.

**Scheme 1:**
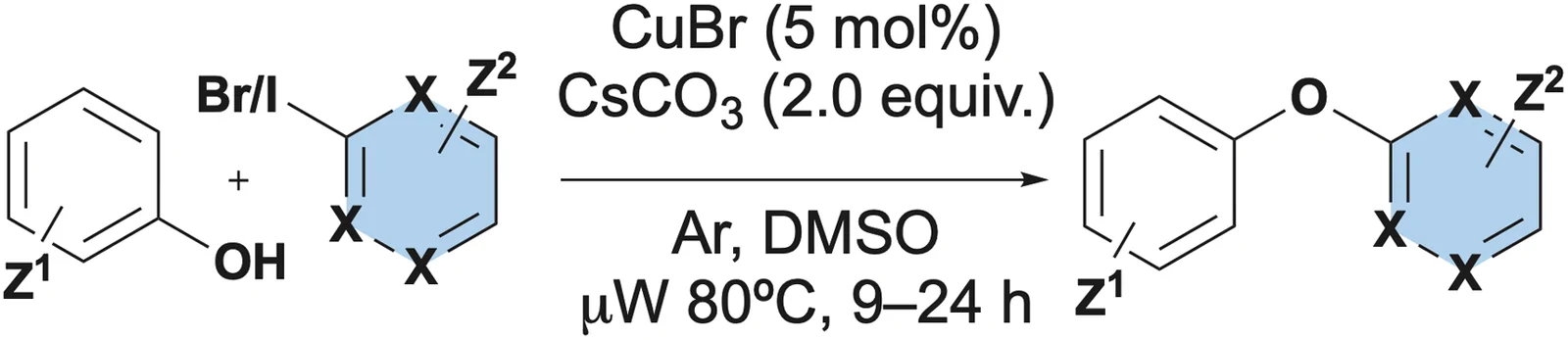
Cu-catalyzed Ullmann-type coupling reaction used to make the library. ^*a*^For aryl iodides 5 mol%, for aryl bromides 10 mol%. ^*b*^(2-Pyridyl)acetone.

The structure of medetomidine, ((±)-4[1-(2,3-dimethylphenyl)ethyl]-1*H*-imidazole), can simplistically be considered in three parts, (1) a lipophilic aryl group; (2) a linker and, (3) a hydrophilic heteroaromatic group (Fig. 2). In lack of extensive structure–activity data on medetomidine analogues,^12^ we envisioned that novel chemical libraries based on this simple model could possess relevant bioactivity and even enhanced activity relative to the lead scaffold medetomidine. At minimum, the library would constitute an exploratory SAR probe and thus provide refining information about the pharmacophore. As such we designed an exploratory library of simplified analogues containing a lipophilic aryl side and a hydrophilic heteroaryl side bridged by an oxygen only, thus reducing the complexity of library generation to simple unsymmetrical biaryl ether synthesis. A limited number of small lipophilic substituents representing steric and positional diversity in the lipophilic aryl groups have been studied, along with a selection of different heteroaromatics (pyridines, pyrazines, quinoline) with some substitutional diversity. The amphiphilic biaryl ether library consisting of 22 compounds (1a–l, 2a–f, 3a–b, 4 and 5) (Tables 1–3 and Fig. 3) was generated employing Ullmann-type couplings between commercially available phenols and aryl iodides or bromides (Scheme 1).^13^ These reactions can be effected in moderate to excellent chemical yields (22–95%) under catalysis by 5 or 10 mol% of CuBr in the presence of Cs_2_CO_3_ as base in DMSO at elevated temperature and with (2-pyridyl)acetone as an added ligand.^13^ Chromatographic purification of the products afforded high purities in the range 91 to >99% as determined by HPLC-UV. The library structures display *C* log *P*-values in the range 2.8–5.4 and tPSA-values in the range 9.2–47.9 (ChemDraw estimates). For comparison, these values are 2.4 and 24.4, respectively, for the lead compound medetomidine, thus indicating that the designed library represents similar as well as an expansion of the property space of medetomidine.

**Table 1:** Structures and results for 2-pyridyl biaryl ethers 1a–l

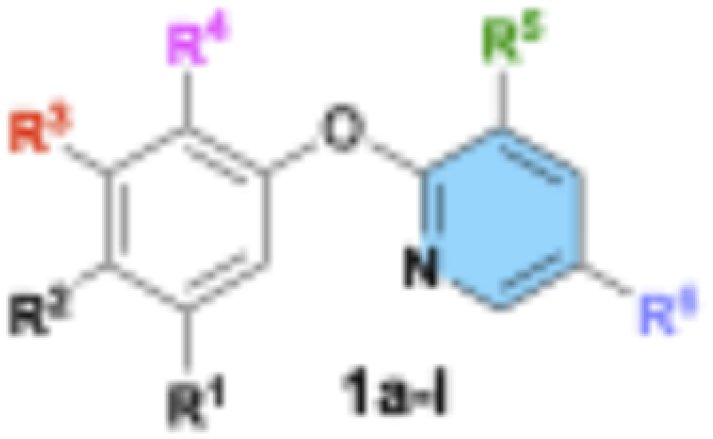								
No.	R^1^	R^2^	R^3^	R^4^	R^5^	R^6^	Yield^a^ (%)	Purity^b^ (%)
1a	H	H	Me	Me	H	H	75	>99
1b	H	H	^*t*^Bu	H	H	H	86	>99
1c	H	^*t*^Bu	H	H	H	H	95	>99
1d	H	^*t*^Bu	H	Br	H	H	93	95
1e	H	^*t*^Bu	H	H	H	CO_2_Me	24	>99
1f	H	^*t*^Bu	H	H	I	H	56	>99
1g	H	H	Me	Me	I	H	45	>99
1h	H	H	^*t*^Bu	H	CO_2_Me	H	36	97
1i	Me	I	Me	H	H	OMe	37	96
1j	H	^*t*^Bu	H	H	F	H	25	97
1k	H	^*t*^Bu	H	H	OMe	H	34	91
1l	H	^*t*^Bu	H	H	OH	H	17	91

a Isolated yields.

b Estimated by relative HPLC-UV peak areas.

**Table 2:** Structures and results for 2-pyrazinyl biaryl ethers 2a–f

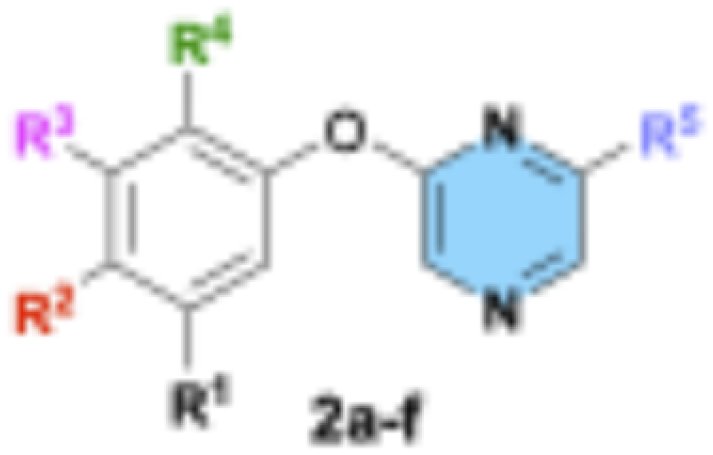							
No.	R^1^	R^2^	R^3^	R^4^	R^5^	Yield^a^ (%)	Purity^b^ (%)
2a	Me	I	Me	H	H	55	96
2b	H	H	Me	Me	H	98	>99
2c	H	^*t*^Bu	H	H	H	91	>99
2d	H	H	^*t*^Bu	H	H	93	>99
2e	H	^*t*^Bu	H	H	OMe	75	98
2f	H	^*t*^Bu	H	Br	H	82	>99

a Isolated yields.

b Estimated by relative HPLC-UV peak areas.

**Table 3:** Structures and results for 3-pyridyl biaryl ethers 3a–b

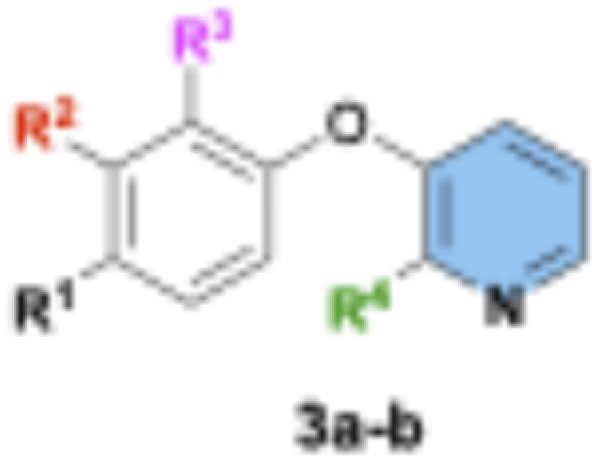						
No.	R^1^	R^2^	R^3^	R^4^	Yield^a^ (%)	Purity^b^ (%)
3a	H	Me	Me	H	73	99
3b	^*t*^Bu	H	H	Br	22	96

a Isolated yields.

b Estimated by relative HPLC-UV peak areas.

### Primary screening against the BiOctα octopamine receptors using the β-arrestin assay

**Fig. 4:**
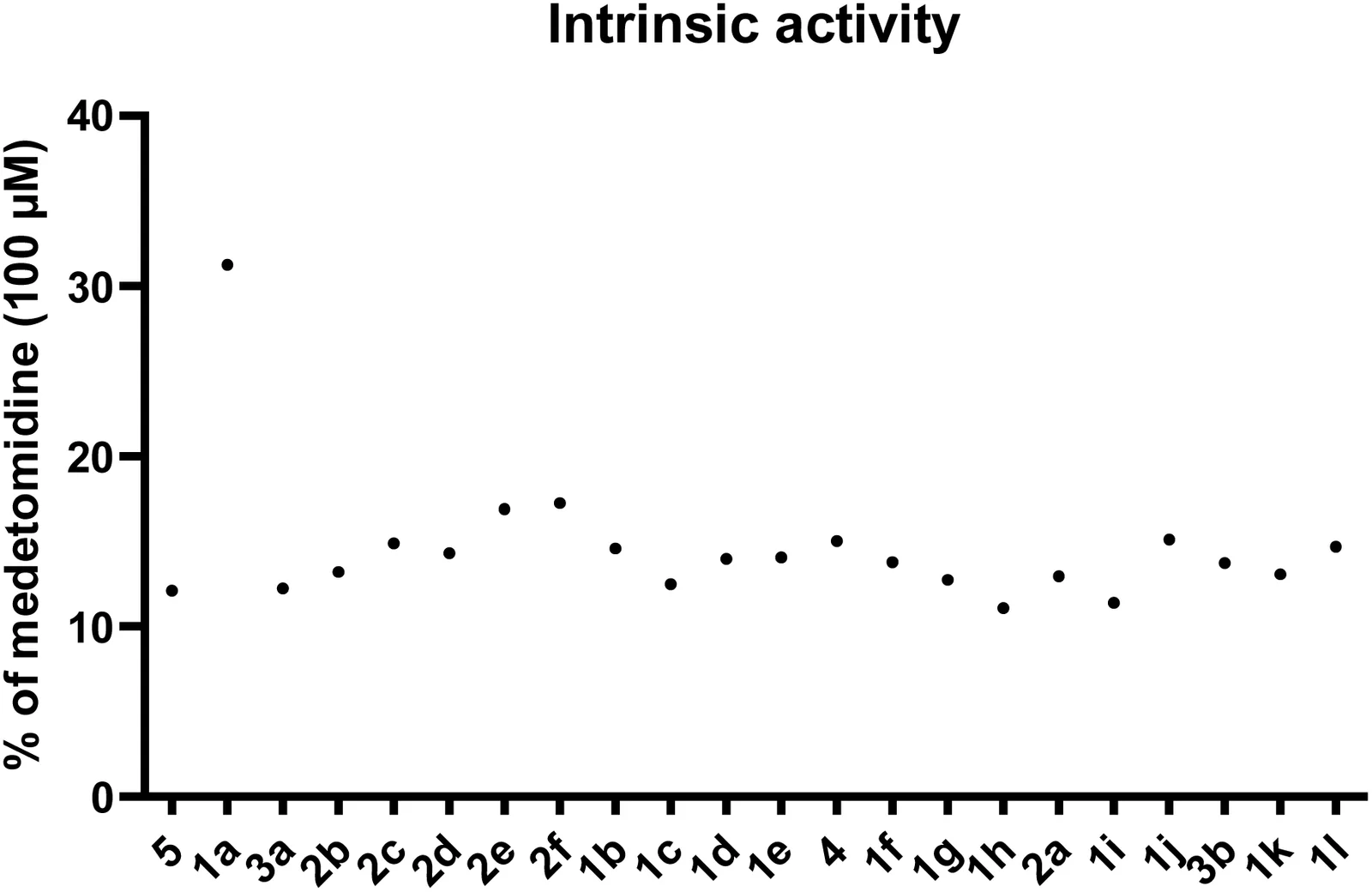
Primary screening results for the 22 synthetized medetomidine analogues. Compounds were tested at a 100 µM concentration using the PathHunter® β-arrestin assay and CHO cells expressing the BiOctα octopamine receptors of *Balanus improvisus*. Intrinsic activity refers to a compound's efficacy relative to the efficacy of the full agonist medetomidine.

A primary screening of the compound library was performed using a novel PathHunter® β-arrestin recruitment assay and CHO cells expressing the BiOctα octopamine receptor of *Balanus improvisus*. All 22 compounds were tested at 100 µM and 1 µM concentrations using medetomidine as a positive control. Based on these results, only compound 1a appears to stimulate β-arrestin in CHO cells expressing BiOctα octopamine receptors (Fig. 4). A calculated intrinsic activity (IA) describes the agonist efficacy and is based on the *E*_max_-value of the positive control. In these experiments IA for 1a was 31% of 100 µM medetomidine stimulation suggesting that 1a act as a partial agonist for BiOctα octopamine receptors. For other compounds stimulations remained very weak or non-existent. At 100 µM concentration 1a induced stimulation approximately a double compared to the baseline. However, at 1 µM concentration, stimulation for 1a remained almost at the basal stimulation level suggesting a weak potency.†

**Fig. 5:**
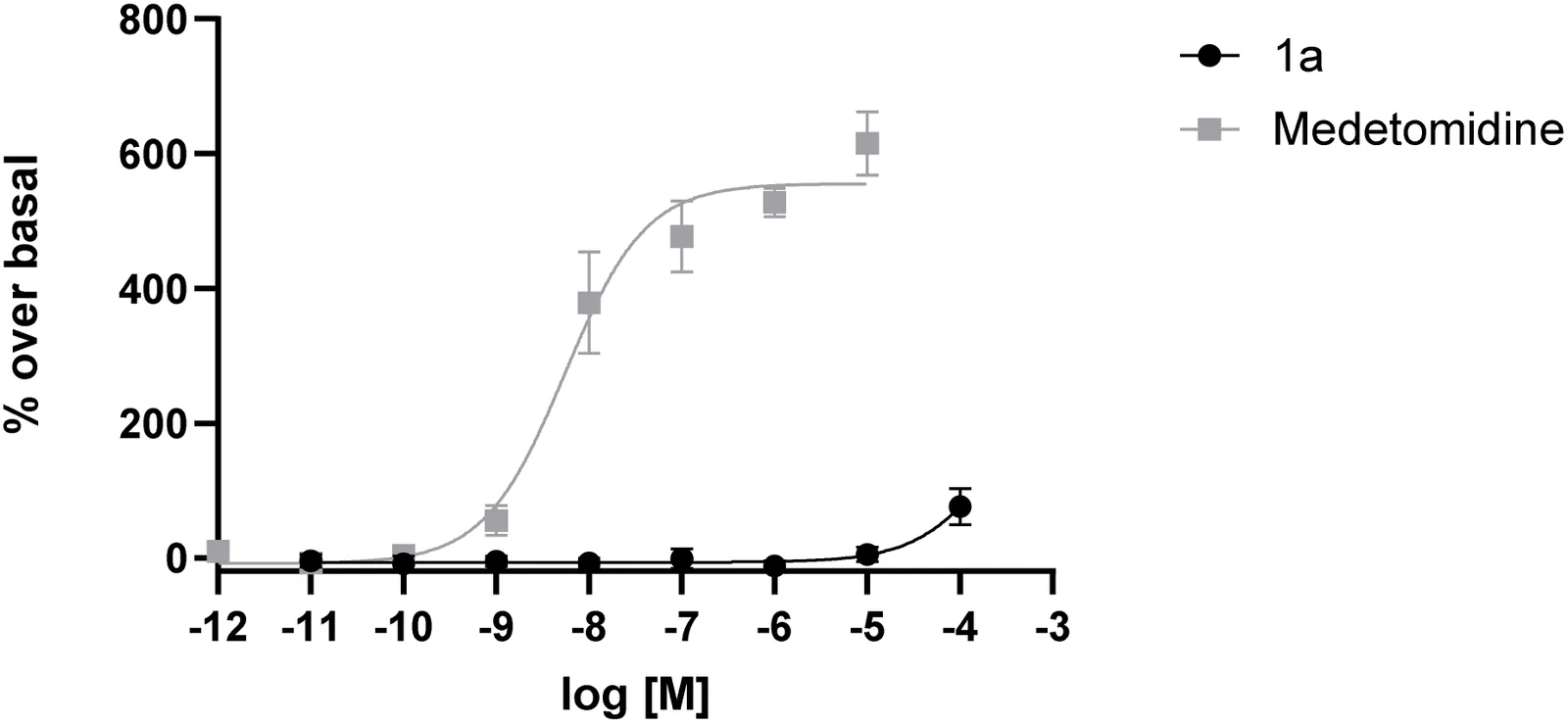
Dose–response curves for 1a and medetomidine based on the PathHunter® β-arrestin recruitment assay in CHO cells expressing BiOctα octopamine receptors of *Balanus improvisus*. The error bars represent the s.e.m. of three independent experiments performed in duplicate.

Dose–response experiments with serial dilutions of test compounds were carried out for 1a and medetomidine using the β-arrestin assay (Fig. 5). The calculated EC_50_-values, a concentration causing half of the maximal effect, and intrinsic activities from these experiments confirm that 1a acts as a weak agonist with micromolar potency at BiOctα octopamine receptors of *Balanus improvisus* (Table 4).

**Table 4:** Ligand induced β-arrestin recruitments in CHO cells expressing BiOctα octopamine receptors of *Balanus improvisus*. Values shown are means ± s.e.m. from three independent experiments performed in duplicate. Intrinsic activity shown as relative to the full agonist medetomidine

Compound	EC_50_ (nM)	Intrinsic activity (% of medetomidine)
1a	14 000 ± 1000	16 ± 4
Medetomidine	5.54 ± 0.61	100

### Secondary screening against the human α_2A_-adrenoreceptors using the [^35^S]GTPγS-binding assay

**Fig. 6:**
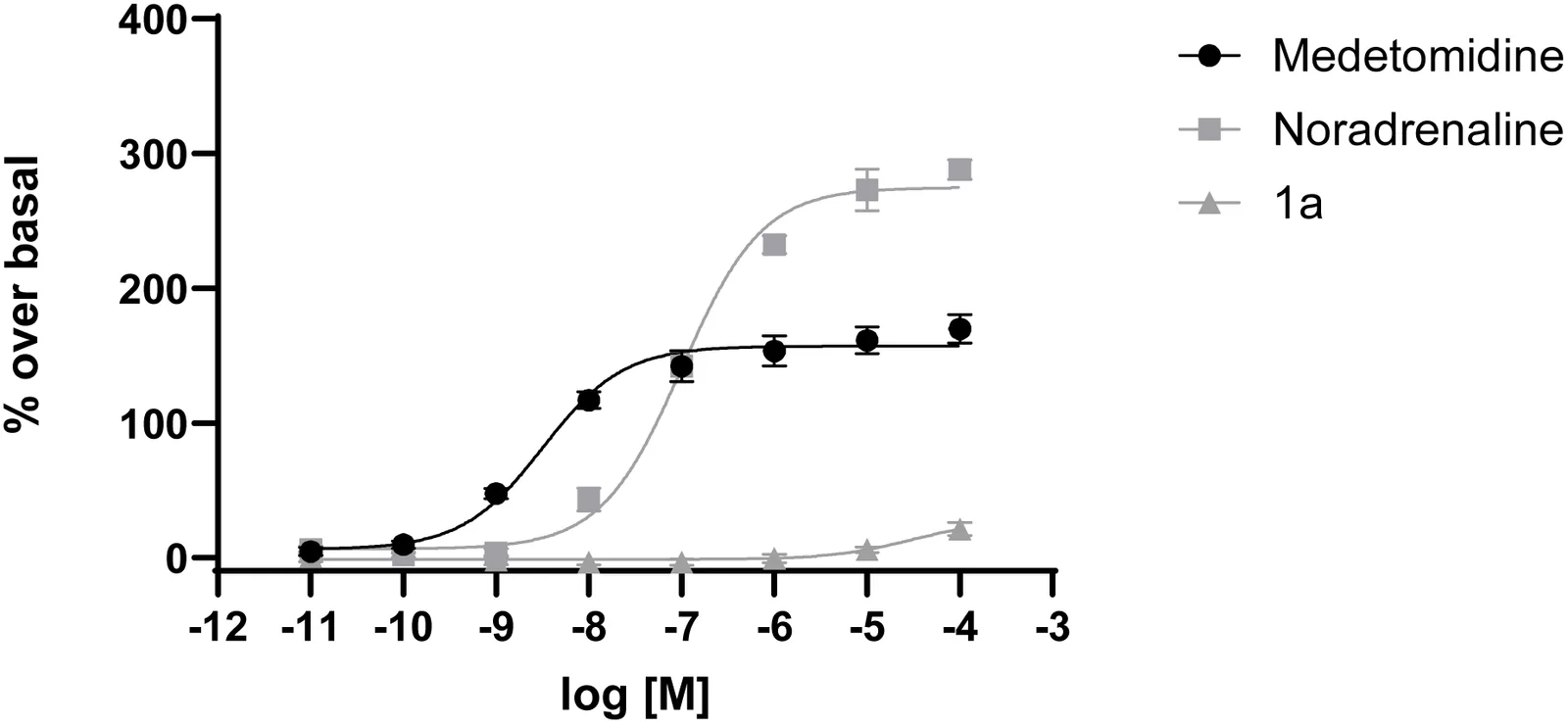
Dose–response curves for 1a, noradrenaline and medetomidine stimulated [^35^S]GTPγS-binding in CHO cells expressing human α_2A_-adrenoceptors. The error bars represent the s.e.m. of three independent experiments performed in duplicate.

In a secondary screening, the most active compound, 1a, was further tested in [^35^S]GTPγS-binding assays against the human α_2A_-adrenoreceptors.^14^ In [^35^S]GTPγS-binding studies an endogenous α_2_-adrenoreceptor agonist noradrenaline was used as a positive control, but also medetomidine was tested in the [^35^S]GTPγS-binding assay against the human α_2A_-adrenoreceptors. The dose–response curves from [^35^S]GTPγS-binding assays are shown in Fig. 6. The calculated EC_50_-values and intrinsic activities of all three compounds is shown in Table 5. Results indicate that 1a acts as a weak agonist with low potency also towards human α_2_-adrenoreceptors.

**Table 5:** Ligand stimulated [^35^S]GTPγS-binding on CHO cell membranes expressing human α_2A_-adrenoceptors. Values shown are means ± s.e.m. from three independent experiments performed in duplicate. Intrinsic activity shown as relative to the full agonist noradrenaline

Compound	EC_50_ (nM)	Intrinsic activity (% of noradrenaline)
1a	37 000 ± 18 000	11 ± 3
Medetomidine	3.20 ± 0.27	57 ± 1
Noradrenaline	101 ± 8	100

### Primary broad activity screening

To broader characterize the library compounds, they were also submitted to the EU-OPENSCREEN screening platform,^15^ where they underwent primary screening against some key microorganisms and in a cell viability assay with HepG2 cells.‡‡See supplementary information (SI) for details. The screening was conducted at one concentration (50 µM) with 2 replicates for antibacterial activity towards *E. faecalis*, Methicillin-susceptible *S. aureus* (MSSA), *A. baumanii*, *P. aeruginosa*, *K. pneumonia*, *E. coli* and antifungal activity towards *A. fumigatus*, *C. albicans* and *C. auris*. None of the compounds were found to be active, defined as >70% growth inhibition in the assays. Two compounds were inconclusive (in the 50–70% growth inhibition range) in the *C. albicans* and *C. auris* assays. Nine compounds from the library were tested and found to be inactive in the HepG2 cell viability assay.‡

## Conclusions

In summary, we have synthesized a chemical library of 22 O-bridged analogues of medetomidine and these compounds were experimentally screened in a new bio-assay based on the β-arrestin recruitment in CHO cells expressing the α-adrenoceptor like BiOctα octopamine receptors of *Balanus improvisus*. Primary screening results show that compound 1a stimulated β-arrestin recruitment as 2-fold in comparison to the baseline activity. In [^35^S]GTPγS-binding assays with CHO cell membranes expressing human α_2A_-adrenoceptors, the intrinsic activity of 1a relative to the natural full agonist noradrenaline was 11%. Based on the primary and secondary screening results, 1a can be considered a weak agonist for both the octopamine- and α_2_-adrenoceptors. Primary screening of activity against *E. faecalis*, Methicillin-susceptible *S. aureus* (MSSA), *A. baumanii*, *P. aeruginosa*,*K. pneumonia*, *E. coli*, *A. fumigatus*, *C. albicans*, *C. auris* and HepG2 cell viability using the EU-OPENSCREEN screening platform did not identify any compounds with significant complementary activity. Overall, this study has demonstrated significant advances in assay technique and our understanding of the medetomidine pharmacophore model.

## Author contributions

K. N. conducted the chemical synthesis, performed data analysis, and contributed to the preparation of the manuscript and the supporting information file. J. L. designed and conducted the bioassays, carried out the bioactivity studies, performed the data analysis of the binding assays, and contributed to the preparation of the manuscript and the supporting information file. J. H. H. contributed to the study design and implementation, supervised the experiments, conducted data analysis, and participated in writing the manuscript. All authors contributed to the quality assurance of the manuscript.

## Conflicts of interest

There are no conflicts to declare.

## Supplementary Material

RA-OLF-D6RA02553F-s001

## Data Availability

Detailed experimental and spectroscopic data for all final products and raw data for biotesting can be found in the supplementary information (SI) and is also available from the corresponding authors upon request. Supplementary information is available. See DOI: https://doi.org/10.1039/d6ra02553f.
