# Supplementary material for: Design and evaluation of an amphiphilic biaryl ether library using a novel cell-based β-arrestin recruitment assay for the octopamine receptors of Balanus improvisus
Source: RSC Adv. 2026 Sep 17;16(44):55655–9. doi: 10.1039/d6ra02553f (PMC13585091; doi:10.1039/d6ra02553f)
Supplement: Supplementary file 1 [file RA-016-D6RA02553F-s001.pdf]

Supplementary Information (SI) for RSC Advances.
This journal is © The Royal Society of Chemistry 2026

Supplementary Information for

Design and evaluation of an amphiphilic biaryl ether library using a novel cell-based β-Arrestin recruitment assay for the octopamine receptors of *Balanus improvisus*

Karoline Nordli[a], Jonne Laurila[b] and Jørn H. Hansen[a]**

[a]Department of Chemistry, UiT The Arctic University of Norway, Chemical Synthesis and Analysis Group, N-9037 Tromsø, Norway.
[b]Institute of Biomedicine, Integrative Physiology and Pharmacology, University of Turku, FI-20014 Turku, Finland.

**Corresponding author e-mail: jorn.h.hansen@uit.no*

Table of contents

1. Experimental section – biotesting 2
 1.1 General considerations........ 2
 1.2 Cloning of ProLink-tagged BiOctα octopamine receptors 2
 1.3 Expression of ProLink-tagged BiOctα receptors in CHO-K1 cells 2
 1.4 PathHunter β-arrestin detection system 3
 1.5 Cell culturing and membrane preparation for CHO cells expressing human α_{2A}-adrenoceptors 3
 1.6 [^{35}S]GTPγS binding assay 3
 1.7 Binding of [^{3}H]RS79948-197 to CHO cell membranes expressing BiOctα receptors 4
 1.8 Additional information 4
2. Experimental section – synthetic chemistry........ 5
 2.1 General considerations........ 5
 2.2 General procedure for CuBr-catalyzed synthesis with aryl iodides 6
 2.3 General procedure for CuBr-catalyzed synthesis with aryl bromides........ 6
3. Compound characterization 7
4. Spectra 17
5. EU-OPENSCREEN primary screening 73
 5.1 Experimental assay details copied from EU-OPENSCREEN........ 73
 5.2 Results from primary screening at EU-OPENSCREEN. 78
6. References........ 79

1. Experimental section – biotesting

1.1 General considerations

Primers for generating ProLink-tagged BiOctα octopamine receptor cDNAs were obtained from TAG Copenhagen A/S (Copenhagen, Denmark). Fetal bovine serum, enzyme-free PBS-based Cell Dissociation Buffer, Ham´s F12 Nutrient Mixture, Penicillin-Streptomycin-Glutamine mixture, Hygromycin B for cell culturing were purchased from Thermo Fisher Scientific (Waltham, MA, USA). Adherent Chinese hamster ovary (CHO) cells (K1 strain) were purchased from American Type Culture Collection (ATCC) (VA, USA). PathHunter Cell Plating 2 reagent and PathHunter Detection Reagent were purchased from Eurofins (Fremont, CA, USA). Radioligand [^{3}H]RS79948-197 was obtained from GE Healthcare (Buckinghamshire, UK). Clonidine and G418 (Geneticin®) were purchased from Sigma-Aldrich (St. Louis, MO, USA). Levomedetomidine and medetomidine were gifts from Orion Pharma (Turku, Finland). Other reagents were of analytical or reagent grade and were purchased from commercial suppliers.

1.2 Cloning of ProLink-tagged BiOctα octopamine receptors

The cDNA encoding the BiOctα octopamine receptor of the barnacle *Balanus improvisus* cloned into the pcDNA3.1+ vector was received as a gift from professor Anders Blomberg (University of Gothenburg, Sweden). The cDNA of BiOctα octopamine receptor was PCR amplified with forward (5´) and reverse (3´) primers using *PfuUltra* II Fusion HS DNA Polymerase (Agilent Technologies, Santa Clara, CA, USA). The 5´-primer contained an artificial NheI restriction site (recognition sequence GCTAGC, coding AS) for subcloning into the pCMV-ProLink vector and a sequence encoding the FLAG peptide epitope at the N-terminus of the receptor. The 3´-primer was designed to remove the stop codon and to maintain reading frame integrity with the ProLink peptide and in addition contained an artificial HindIII restriction site (recognition sequence AAGCTT, coding KL) for subcloning into the pCMV-ProLink vector. The PCR-amplified cDNA was digested with NheI and HindIII and ligated into the pCMV-ARMS2-ProLink vector (DiscoveRx, Fremont, CA, USA) for expression in mammalian cells. The nucleotide sequence in the expression plasmids was confirmed by DNA sequence analysis using vector- and gene-specific primers.

1.3 Expression of ProLink-tagged BiOctα receptors in CHO-K1 cells

Adherent Chinese hamster ovary (CHO) cells (K1 strain) expressing the EA/β-Arrestin fusion protein (Eurofins, Fremont, CA, USA) were cultured in Ham´s F12 Nutrient Mixture supplemented with 10% heat-inactivated fetal bovine serum, 2 mM glutamine, penicillin (100 U/ml), streptomycin (100 µg/ml) and hygromycin B (300 µg/ml). The ProLink-tagged BiOctα receptor construct was transfected into the CHO cells using the FuGENE® HD Transfection Reagent kit (Roche Applied Science, Indianapolis, IN, USA). Transfection mixtures were prepared in serum- and antibiotic-free Ham´s F12 Nutrient Mixture and contained 2-3 mg of plasmid DNA. After 24 h incubation in a humidified 5% CO_2 atmosphere at 37 °C, the pCMV-PK-based transfected cell cultures were expanded using 900 µg/ml of the neomycin analogue G418 (Geneticin®). After selection, cells were detached with enzyme-free Cell Dissociation Buffer, suspensed to the PathHunter Cell Plating 2 reagent (Eurofins, Fremont, CA, USA) and plated on 96-well Cell Carrier plates (Revvity Turku, Finland) at 11 200 cells per well. After 24 h incubation, cells were stimulated with 100 µM clonidine, levomedetomidine, medetomidine and octopamine. The chemiluminescence signal was detected using the PathHunter detection system (see below). The transfected cell clones with the highest stimulation levels were chosen for further experiments and were subsequently maintained in 600 µg/ml G418 and 300 µg/ml hygromycin B. For subsequent experiments, a batch of cells were stored frozen in liquid nitrogen until used.

1.4 PathHunter β-arrestin detection system

Transfected CHO cells expressing BiOctα receptors and the EA/β-Arrestin fusion protein were cultured in Ham´s F12 Nutrient Mixture supplemented with 10% heat-inactivated fetal bovine serum, 2 mM glutamine, penicillin (100 U/ml), streptomycin (100 µg/ml), G418 (600 µg/ml) and hygromycin B (300 µg/ml) in a humidified 5% CO_2 atmosphere at 37 °C. Cultured cells were detached with enzyme-free Cell Dissociation Buffer and suspensed to the PathHunter Cell Plating 2 reagent (Eurofins, Fremont, CA, USA) and plated on 96-well Cell Carrier plates (Revvity, Turku, Finland) at 11 200 cells per well. After 24 h incubation, cells were stimulated with test compounds (90 min), next 20 µl of PathHunter detection reagent was added into each well, incubated 60 min and the chemiluminescence signal was detected using a multiplate reader instrument Ensight (Revvity, Turku, Finland).

1.5 Cell culturing and membrane preparation for CHO cells expressing human α_{2A}-adrenoceptors

CHO cells stably expressing human α_{2A}-adrenoceptors were cultured as described by Pohjanoksa *et al.* 1997. The production of CHO cell line with expression vector based on pMAMneo for expression of wild-type α_{2A}-adrenoceptors has been described previously (Marjamäki *et al.*, 1992, Marjamäki *et al.*, 1993, Pohjanoksa *et al.*, 1997). Before the [^{35}S]GTPγS binding assays, the cultured cells were tested for their capacity to bind the α_2-adrenoceptor antagonist radioligand [^{3}H]RS-79948-197 (GE Healthcare, London, UK). Confluent cells were harvested into chilled phosphate-buffered saline, pelleted and frozen at -70 °C.

For membrane preparations frozen CHO cell pellets were thawed and suspended in hypotonic lysis buffer (10 mM Tris–HCl, 0.1 mM EDTA, 0.32 mM sucrose, pH 7.4) and homogenised using an Ultra-Turrax homogeniser (3 × 10 s at 8000 rpm). The homogenate was centrifuged at 180 g for 15 min to remove cell nuclei, unbroken cells and aggregates. The supernatants were pooled and centrifuged at 50,227 g for 30 min. The pellet was washed with TE buffer (10 mM Tris, 0.1 mM EDTA) and re-centrifugated as above. The membranes were then suspended in TE buffer, aliquoted and stored at -70 °C until used. Protein concentrations were determined with the method of Bradford (Bradford, 1976) using bovine serum albumin as reference.

1.6 [^{35}S]GTPγS binding assay

Agonist-induced stimulation of [^{35}S]GTPγS binding was measured essentially as described previously (Peltonen *et al.,* 1998). Briefly, membranes were thawed and diluted with binding buffer (25 mM Tris, 1 mM EDTA, 5 mM $MgCl_2$, 20 mM NaCl, 1 µM GDP, 1 mM DTT, pH 7.4). Incubations were performed on 96-well Millipore MultiScreen MSFBN glass-fibre filter plates. Samples containing 5 µg of membrane protein were incubated with serial dilutions of the test compounds and 0.1 nM [^{35}S]GTPγS. Reactions were terminated after 30 min incubation at RT by rapid vacuum filtration using a Millipore MultiScreen Vacuum Manifold. The filter plates were washed three times with ice-cold wash buffer (20 mM Tris, 1 mM EDTA, 5 mM $MgCl_2$, pH 7.4). Filters were dried and 50 µl SuperMix scintillation cocktail was added into each well. The incorporated radioactivity was measured using a MicroBeta2 microplate counter (Revvity, Turku, Finland). All experiments were performed in duplicate and repeated at least three times. Analysis of the results with GraphPad Prism software yielded estimates of agonist potency (EC_{50}) and efficacy (intrinsic activity in comparison to the natural full agonist noradrenaline).

1.7 Binding of [^{3}H]RS79948-197 to CHO cell membranes expressing BiOctα receptors

Previously the BiOctα octopamine receptor was shown to bind the α_2-adrenoceptor antagonist radioligand [^{3}H]RS79948-197 with low affinity (supplementary material in Lind *et al.,* 2010). To characterize the BiOctα octopamine receptor expression in transfected CHO cells expressing the EA/β-Arrestin fusion protein saturation binding assays were carried out with [^{3}H]RS79948-197 radioligand solution diluted with 1:5 unlabelled RS79948-197 ligand. Briefly, 8 µg of membrane protein were incubated with final concentration of tritium labelled and unlabelled RS79948-197 ligand solution ranging from 0.78125 nM to 100 nM in 50 mM of K^+-phosphate buffer (pH 7.4). After 30 min at RT, reactions were terminated using rapid filtration through glass fiber filters (Whatman GF/B). The filters were washed (50 mM Tris-HCl, 10 mM EDTA, pH 7.4), placed into scintillation vials with Optiphase "HiSafe" III and the bound radioactivity was measured in a Wallac 1410 scintillation counter (Wallac, Turku, Finland). Non-specific binding was determined with 10 µM phentolamine.

1.8 Additional information

During assay optimization, a DMSO compatibility test was conducted for the PathHunter β-arrestin recruitment assay, and the assay was shown to tolerate DMSO concentrations of up to 2%. Final DMSO concentration in the assay never exceeded 0.5 % (v/v), a level tolerated by the PathHunter system and routinely used also in other functional GPCR assays. No visible precipitation was observed under the assay conditions.

Plate-to-plate reproducibility was assessed using the positive and negative control signals obtained from four independent β-arrestin assay plates. The coefficient of variation (CV) for the medetomidine positive control signals across the four plates was found 8.0%, while the corresponding CV for the negative control (water) was 16.9%. The plate-specific signal-to-noise (S/N) ratios ranged from 5.03 to 6.81, with a mean S/N ratio of 6.01.

For evaluating assay quality assessments also the Z'-factor was determined from the measured signals and calculated according to the following formula:

$$Z'\,factor = 1 - \frac{3 \times (\sigma_p + \sigma_n)}{|\mu_p - \mu_n|}$$

μ: mean
σ: standard deviation
p: positive controls
n: negative controls

where µ and σ represent the mean and population standard deviation, respectively, of the 10 µM medetomidine (p) as positive control, 0.1% DMSO, and water (n) as negative controls. Based on these experiments, the Z'-factor values (mean ± SD) were 0.66 ± 0.04 for 0.1% DMSO and 0.67 ± 0.03 for water. Based on these parameters, the overall performance of the β-arrestin recruitment assay for BiOctα receptors expressed in CHO cells can be considered acceptable.

Separate concentration-response curves for compound 1a and medetomidine, obtained from three independent experiments, each performed in duplicate using the PathHunter β-arrestin recruitment assay, are shown below.

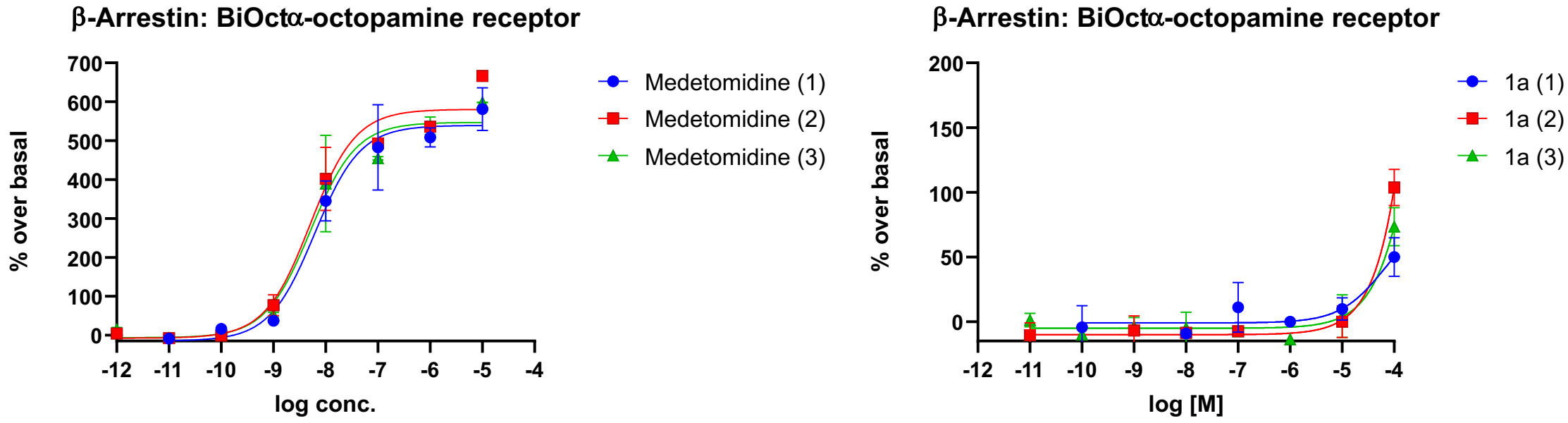

Based on the concentration-response experiments and analyses using the log(agonist) vs. response – Variable slope (four parameters) model, the Hill slopes (mean ± SEM) were 1.52 ± 0.67 for compound 1a and 1.05 ± 0.09 for medetomidine. The corresponding EC_{50} values (nM), with 95% confidence intervals, were 14 000 (5 600 - 24 000) for compound 1a and 5.54 (4.04 - 7.05) for medetomidine.

The 31% activity observed in the primary screening and the 16% activity obtained from the subsequent dose-response study are not directly comparable, as they were determined using different experimental approaches. In the primary screening, the 31% activity was based on the relative stimulation measured from only two wells for both medetomidine and compound 1a, tested at a single concentration of 100 µM. Therefore, this value should be considered only as an initial indication of activity rather than a reliable quantitative measure of efficacy.

The subsequent dose-response study was performed to confirm the activity observed in the primary screen and to provide a more reliable assessment of the compound's activity across a range of concentrations. The 16% activity obtained from the dose-response study therefore represents the confirmed response under the conditions of the concentration-response experiment. This approach is consistent with the general screening workflow, in which primary screening results are considered preliminary and potential hits are subsequently evaluated using concentration-response experiments to confirm activity and characterize potency and efficacy.

2. Experimental section – synthetic chemistry

2.1 General considerations

Unless otherwise noted, purchased chemicals were used as received without further purification. Solvents were dried according to standard procedures over molecular sieves and degassed with argon for at least 5 min. Flash chromatography was carried out on silica gel 60 (230-400 mesh). Autoflash chromatography (normal phase) and prep-HPLC column chromatography was conducted on CombiFlash EZ prep system. Normal-phase chromatography was performed on RediSep®Rf High Performance Gold columns in the appropriate size with the sample preloaded on a precolumn containing celite. Thin layer chromatography was carried out using TLC Silica Gel 60 F254 (Merck) and visualized by short-wavelength ultraviolet light or by treatment with an appropriate stain. Prep-HPLC column chromatography was conducted on a YMC-Actus Triart C18 Semi-preparative HPLC column, 12 nm, S-5 µm 20x150mm 5 µm with a YMC-Triart C18, semi-preparative Guard Cartridge incl. Sealing, 12 nm, S-5 µm, 10 x 20. The sample was liquid loaded pure or mixed with a suitable solvent. Solvent systems are reported as follows: (solventA:solventB [the percentage of solvent]), when the autoflash system was used. High-resolution mass spectra HRMS(ESI) were recorded from methanol solutions on a LTQ Orbitrap XL (Thermo Scientific) in either positive or negative electrospray ionization (ESI) mode. NMR spectra were obtained on a 400 MHz Bruker Avance III HD at 20 °C. The shifts are reported in ppm relative to the solvent residual peak ($CDCl_3$: δH 7.26 and δC 77.16). ^{13}C-NMR spectra were obtained

with ^{1}H decoupling. Data is presented as follows: chemical shift, multiplicity (s = singlet, bs = broad singlet, d = doublet, t = triplet, q = quartet, dt = doublet of triplets, m = multiplet), coupling constant (J in Hz). FT-IR spectra were recorded on a Cary 630 FTIR (Agilent Technologies).

2.2 General procedure for CuBr-catalyzed synthesis with aryl iodides

An oven dried, sealed microwave reactor was charged with a magnetic stir bar, CuBr (0.05 equiv), Cs_2CO_3 (2.0 equiv) and any remaining solids – phenols (1.2 equiv) and/or heteroaryl halides (1.0 equiv). The tube was evacuated and backfilled with argon 3 times. Under a counter-flow of argon, phenols (1.2 equiv) (if liquid), aryl heterohalide (1.0 equiv) (if liquid), DMSO (1.5 mL/mmol) and ligand (2-Pyridyl)acetone (0.1 equiv) were added by syringe. The tube was evacuated and backfilled with argon again 3 times and sealed. The reaction mixture was heated to 80°C for the required time period (9-24 h). After cooling to room temperature, the mixture was diluted with ethyl acetate (10 mL/mmol), passed through a fritted glass filter to remove the inorganic salts and the solvent was removed *in vacuo*. The residue was purified by column chromatography on silica gel (Heptane:EtOAc) and the product was dried under high vacuum for at least 1 h. (Zhang et al., 2009)

2.3 General procedure for CuBr-catalyzed synthesis with aryl bromides

An oven dried, sealed microwave reactor was charged with a magnetic stir bar, CuBr (0.1 equiv), Cs_2CO_3 (2.0 equiv) and any remaining solids – phenols (1.2 equiv.) and/or heteroaryl halides (1.0 equiv.). The tube was evacuated and backfilled with argon (this procedure was repeated three times). Under a counter flow of argon, phenols (1.2 equiv) (if liquid), heteroaryl halide (1.0 equiv.) (if liquid), DMSO (1.5 mL/mmol) and ligand (2-Pyridyl)acetone (0.2 equiv) were added by syringe. The tube was evacuated and backfilled with argon again (this procedure was repeated three times) and sealed. The reaction mixture was heated to 90°C for the required time period (15-24h). After cooling to room temperature, the mixture was diluted with ethyl acetate (10 mL/mmol) passed through a fritted glass filter to remove the inorganic salts and the solvent was removed under vacuum. The residue was purified by column chromatography on silica gel (Heptane:EtOAc) and the product was dried under high vacuum for at least 1 h. (Zhang et al., 2009)

3. Compound characterization

1-methyl-4-phenoxybenzene (5)

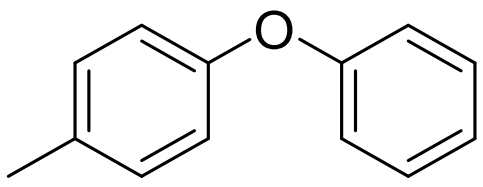

The compound has been previously reported by Zeng et al. (2018). Prepared according to the general procedure for aryl bromides for 24 h at 150 °C. The crude product was purified by silica gel column chromatography (heptane:EtOAc 100:10) to give the diaryl ether as a clear oil (85 mg, 92 %). **^{1}H NMR** (400 MHz, $CDCl_3$) δ 7.32 (t, *J* = 7.7 Hz, 2H), 7.14 (d, *J* = 8.1 Hz, 2H), 7.11 – 7.04 (m, 1H), 6.99 (d, *J* = 8.0 Hz, 2H), 6.93 (d, *J* = 8.1 Hz, 2H), 2.34 (s, 3H). **^{13}C NMR** (101 MHz, $CDCl_3$) δ 158.0, 154.9, 133.1, 130.4, 129.8, 122.9, 119.3, 118.5, 20.9. **HR-MS** (+ESI) *m/z* calculated for $C_{13}H_{13}O^+$ $[M+H]^+$: 185,0963; found: 185,0961. **Purity** 96.0%. The data is consistent with literature (Zeng et al., 2018).

2-(2,3-dimethylphenoxy)pyridine (1a)

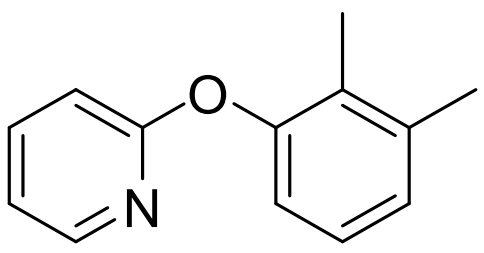

The compound was found in a SciFinder search, but characterization data lacked in the corresponding references. Prepared according to the general procedure for aryl bromides for 15 h at 90 °C. The crude product was purified by silica gel column chromatography (heptane:EtOAc 100:10) and prep-HPLC column chromatography (water:acetonitrile 100:0 to 100:100 gradient) to give the diarylether as a white solid (75 mg, 75 %).

^{1}H NMR (400 MHz, $CDCl_3$) δ 8.23 – 8.15 (m, 1H), 7.65 (ddd, *J* = 8.3, 7.1, 2.0 Hz, 1H), 7.14 (t, *J* = 7.7 Hz, 1H), 7.05 (d, *J* = 7.5 Hz, 1H), 6.99 – 6.88 (m, 2H), 6.85 (dt, *J* = 8.3, 0.9 Hz, 1H), 2.34 (s, 3H), 2.11 (s, 3H). **^{13}C NMR** (101 MHz, $CDCl_3$) δ 164.1, 152.1, 148.0, 139.3, 138.8, 129.5, 126.8, 126.3, 119.5, 117.9, 110.6, 20.2, 12.6. **IR** (neat, cm^{-1}) ν 3015, 2922, 2863, 1584, 1572, 1464, 1426, 1263, 1243, 1191, 1063, 775. **HR-MS** (+ESI) *m/z* calculated for $C_{13}H_{14}NO^+$ $[M+H]^+$: 200,1072; found: 200,1068. **Purity** >99%.

3-(2,3-dimethylphenoxy)pyridine (3a)

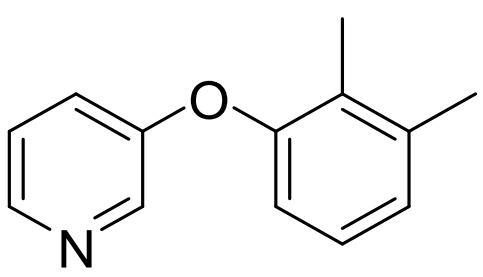

Prepared according to the general procedure for aryl bromides for 15 h at 90 °C. The crude product was purified by silica gel column chromatography (heptane:EtOAc 100:5) and prep-HPLC column

chromatography (water:acetonitrile 100:0 to 100:100 gradient) to give the diaryl ether as a yellow oil (73 mg, 73 %).

^{1}H NMR (400 MHz, $CDCl_3$) δ 8.33 (d, *J* = 2.9 Hz, 1H), 8.29 (dd, *J* = 4.7, 1.4 Hz, 1H), 7.19 (dd, *J* = 8.4, 4.6 Hz, 1H), 7.15 – 7.06 (m, 2H), 7.01 (d, *J* = 7.5 Hz, 1H), 6.78 (d, *J* = 8.0 Hz, 1H), 2.32 (s, 3H), 2.14 (s, 3H). **^{13}C NMR** (101 MHz, $CDCl_3$) δ 154.8, 153.4, 143.5, 140.1, 139.3, 128.8, 126.6, 126.4, 124.0, 123.5, 117.7, 20.2, 12.2. **HR-MS** (+ESI) *m/z* calculated for $C_{13}H_{14}NO^+$ $[M+H]^+$: 200,1072; found: 200,1068. **Purity** 99%.

2-(2,3-dimethylphenoxy)pyrazine (2b)

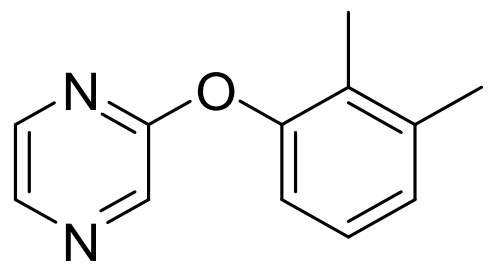

Prepared according to the general procedure for aryl iodides for 20 h at 80 °C. The crude product was purified by silica gel column chromatography (heptane:EtOAc 100:5) and prep-HPLC column chromatography (water:acetonitrile 100:0 to 100:100 gradient) to give the diarylether as a white solid (98 mg, 98 %).

^{1}H NMR (400 MHz, $CDCl_3$) δ 8.41 (d, *J* = 1.4 Hz, 1H), 8.23 (d, *J* = 2.8 Hz, 1H), 8.08 (dd, *J* = 2.7, 1.4 Hz, 1H), 7.16 (t, *J* = 7.7 Hz, 1H), 7.09 (d, *J* = 7.4 Hz, 1H), 6.92 (dd, *J* = 8.0, 1.4 Hz, 1H), 2.33 (s, 3H), 2.09 (s, 3H). **^{13}C NMR** (101 MHz, $CDCl_3$) δ 160.5, 151.2, 141.4, 139.1, 138.2, 135.5, 129.4, 127.5, 126.5, 119.4, 20.3, 12.7. **IR** (neat, cm^{-1}) ν 3061, 2919, 1530, 1465, 1398, 1278, 1232, 1177, 1151, 1065, 1007, 841, 786. **HR-MS** (+ESI) *m/z* calculated for $C_{12}H_{13}N_2O^+$ $[M+H]^+$: 201,1025; found: 201,1020. **Purity** > 99%.

2-(4-*tert*-butylphenoxy)pyrazine (2c)

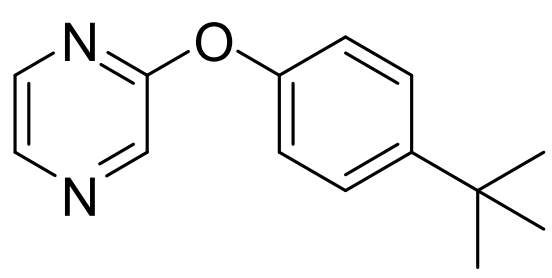

The compound has been previously reported by Bhilare et al. (2018). Prepared according to the general procedure for aryl iodides for 20 h at 80 °C. The crude product was purified by silica gel column chromatography (heptane:EtOAc 100:5) and prep-HPLC column chromatography (water:acetonitrile 100:0 to 100:100 gradient) to give the diarylether as a white solid (104 mg, 91 %).

^{1}H NMR (400 MHz, $CDCl_3$) δ 8.41 (d, *J* = 1.4 Hz, 1H), 8.25 (d, *J* = 2.7 Hz, 1H), 8.11 (dd, *J* = 2.7, 1.4 Hz, 1H), 7.48 – 7.40 (m, 2H), 7.13 – 7.05 (m, 2H), 1.34 (s, 9H). **^{13}C NMR** (101 MHz, $CDCl_3$) δ 160.5, 150.7, 148.3, 141.2, 138.4, 136.1, 126.9, 120.7, 34.6, 31.6. **IR** (neat, cm^{-1}) ν 3059, 2961, 2904, 2868, 1507, 1467,

1399, 1280, 1210, 1171, 1008, 890, 838. **HR-MS** (+ESI) *m/z* calculated for $C_{14}H_{17}N_2O^+$ $[M+H]^+$: 229,1337; found: 229,1332. **Purity** >99%. The data is consistent with literature (Bhilare et al., 2018).

2-(3-*tert*-butylphenoxy)pyrazine (2d)

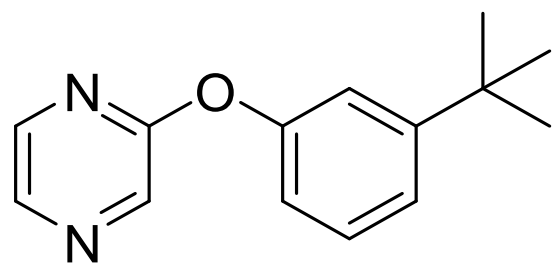

Prepared according to the general procedure for aryl iodides for 8 h at 80 °C. The crude product was purified by silica gel column chromatography (heptane:EtOAc 100:5) and prep-HPLC column chromatography (water:acetonitrile 100:0 to 100:100 gradient) to give the diaryl ether as a clear oil (106 mg, 93 %).

^{1}H NMR (400 MHz, $CDCl_3$) δ 8.40 (d, *J* = 1.4 Hz, 2H), 8.25 (d, *J* = 2.7 Hz, 1H), 8.12 (dd, *J* = 2.7, 1.4 Hz, 1H), 7.40 – 7.27 (m, 2H), 7.17 (t, *J* = 2.1 Hz, 1H), 6.97 (ddd, *J* = 7.9, 2.4, 1.1 Hz, 1H), 1.33 (s, 9H). **^{13}C NMR** (101 MHz, $CDCl_3$) δ 160.5, 153.7, 153.0, 141.4, 138.5, 135.9, 129.4, 122.7, 118.5, 118.2, 35.0, 31.4. **HR-MS** (+ESI) *m/z* calculated for $C_{14}H_{17}N_2O^+$ $[M+H]^+$: 229,1337; found: 229,1331. **Purity** >99%.

2-methoxy-4-(4-*tert*-butylphenoxy)pyrazine (2e)

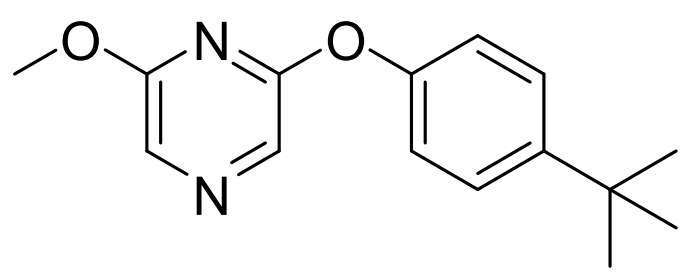

Prepared according to the general procedure for aryl iodides for 16 h at 80 °C. The crude product was purified by silica gel column chromatography (heptane:EtOAc 100:5) and prep-HPLC column chromatography (water:acetonitrile 100:0 to 100:100 gradient) to give the diaryl ether as a white solid (96 mg, 75 %).

^{1}H NMR (400 MHz, $CDCl_3$) δ 7.93 (d, *J* = 0.5 Hz, 1H), 7.76 (d, *J* = 0.6 Hz, 1H), 7.44 – 7.37 (m, 2H), 7.13 – 7.06 (m, 2H), 3.85 (s, 3H), 1.34 (s, 9H). **^{13}C NMR** (101 MHz, $CDCl_3$) δ 159.2, 158.5, 151.2, 148.2, 127.9, 126.7, 124.0, 120.4, 53.9, 34.6, 31.6. **HR-MS** (+ESI) *m/z* calculated for $C_{15}H_{19}N_2O_2^+$ $[M+H]^+$: 259,1442; found: 259,1432. **Purity** 98%.

2-(2-bromo-4-*tert*-butylphenoxy)pyrazine (2f)

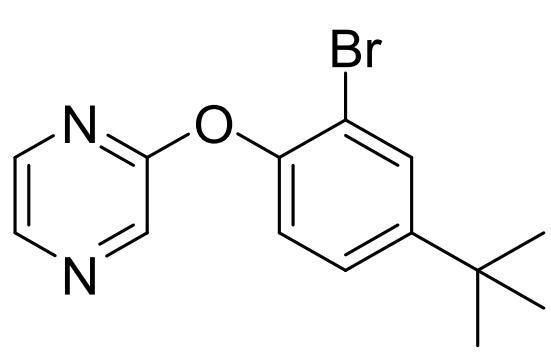

Prepared according to the general procedure for aryl iodides for 22 h at 80 °C. The crude product was purified by silica gel column chromatography (heptane:EtOAc 100:10) and prep-HPLC column chromatography (water:acetonitrile 100:0 to 100:100 gradient) to give the diarylether as a clear oil (126 mg, 82 %).

^{1}H NMR (400 MHz, $CDCl_3$) δ 8.48 (d, *J* = 1.5 Hz, 1H), 8.27 (d, *J* = 2.6 Hz, 1H), 8.08 (dd, *J* = 2.7, 1.4 Hz, 1H), 7.64 (d, *J* = 2.3 Hz, 1H), 7.39 (dt, *J* = 8.5, 2.0 Hz, 1H), 7.14 (dd, *J* = 8.6, 1.5 Hz, 1H), 1.34 (s, 9H). **^{13}C NMR** (101 MHz, $CDCl_3$) δ 159.6, 150.5, 147.7, 141.0, 138.6, 135.8, 130.8, 125.9, 123.1, 115.9, 34.7, 31.4. **IR** (neat, cm^{-1}) ν 2960, 2868, 1533, 1491, 1466, 1398, 1274. **HR-MS** (+ESI) *m/z* calculated for $C_{14}H_{16}BrN_2O^+$ $[M+H]^+$: 307,0442; found: 307,0439. **Purity** >99%.

2-(3-*tert*-butylphenoxy)pyridine (1b)

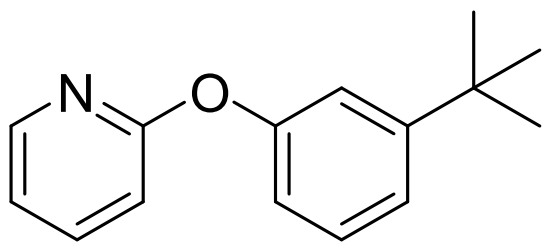

The compound has been previously reported by Li et al. (2017). Prepared according to the general procedure for aryl bromides for 15 h at 90 °C. The crude product was purified by silica gel column chromatography (heptane:EtOAc 100:15) and prep-HPLC column chromatography (water:acetonitrile 100:0 to 100:100 gradient) to give the diarylether as a white solid (98 mg, 86 %).

^{1}H NMR (400 MHz, $CDCl_3$) δ 8.22 (ddd, *J* = 5.0, 2.0, 0.8 Hz, 1H), 7.67 (ddd, *J* = 8.3, 7.2, 2.1 Hz, 1H), 7.33 (t, *J* = 7.9 Hz, 1H), 7.23 (ddd, *J* = 7.9, 2.0, 1.1 Hz, 1H), 7.17 (t, *J* = 2.1 Hz, 1H), 7.02 – 6.92 (m, 2H), 6.88 (dt, *J* = 8.3, 0.9 Hz, 1H), 1.33 (s, 9H). **^{13}C NMR** (101 MHz, $CDCl_3$) δ 164.0, 154.1, 153.4, 148.0, 139.5, 129.2, 121.8, 118.4, 118.4, 118.1, 111.5, 34.9, 31.4. **HR-MS** (+ESI) *m/z* calculated for $C_{15}H_{18}NO^+$ $[M+H]^+$: 228,1384; found: 228,1380. **Purity** >99%. The data is consistent with literature. (Li et al., 2017)

2-(4-*tert*-butylphenoxy)pyridine (1c)

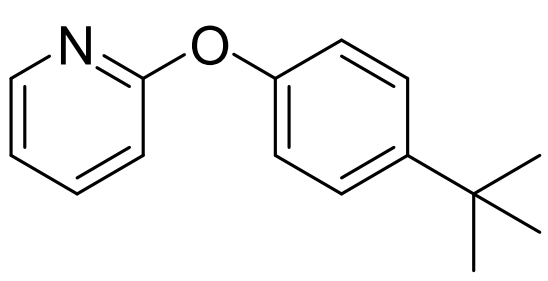

The compound has been previously reported by Kinuta et al. (2015). Prepared according to the general procedure for aryl bromides for 15 h at 90 °C. The crude product was purified by silica gel column chromatography (heptane:EtOAc 100:15) and prep-HPLC column chromatography (water:acetonitrile 100:0 to 100:100 gradient) to give the diaryl ether as a white solid (108 mg, 95 %).

^{1}H NMR (400 MHz, $CDCl_3$) δ 8.21 (ddd, *J* = 5.0, 2.1, 0.8 Hz, 1H), 7.66 (ddd, *J* = 8.3, 7.2, 2.0 Hz, 1H), 7.48 – 7.36 (m, 2H), 7.11 – 7.03 (m, 2H), 6.97 (ddd, *J* = 7.2, 5.0, 1.0 Hz, 1H), 6.89 (dt, *J* = 8.3, 0.9 Hz, 1H), 1.34 (s, 9H). **^{13}C NMR** (101 MHz, $CDCl_3$) δ 164.0, 151.8, 147.9, 147.4, 139.5, 126.7, 120.6, 118.4, 111.5, 34.5, 31.6. **HR-MS** (+ESI) *m/z* calculated for $C_{15}H_{18}NO^+$ $[M+H]^+$: 228,1384; found: 228,1380. **Purity** >99%. The data is consistent with literature. (Kinuta et al., 2015)

2-(2-bromo-4-*tert*-butylphenoxy)pyridine (1d)

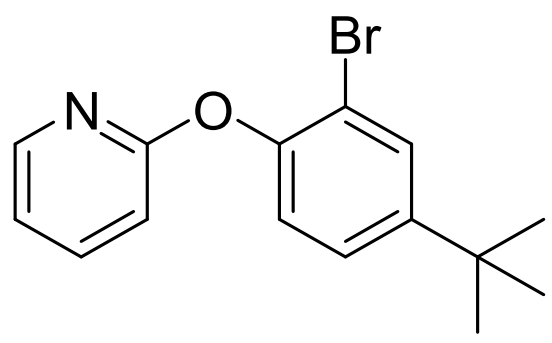

Prepared according to the general procedure for aryl bromides for 22 h at 90 °C. The crude product was purified by silica gel column chromatography (heptane:EtOAc 100:10) and prep-HPLC column chromatography (water:acetonitrile 100:0 to 100:100 gradient) to give the diaryl ether as a clear oil (213 mg, 93 %).

^{1}H NMR (400 MHz, $CDCl_3$) δ 8.17 (ddd, *J* = 4.9, 2.0, 0.8 Hz, 1H), 7.70 (ddd, *J* = 8.3, 7.2, 2.0 Hz, 1H), 7.63 (d, *J* = 2.3 Hz, 1H), 7.36 (dd, *J* = 8.5, 2.3 Hz, 1H), 7.12 (d, *J* = 8.5 Hz, 1H), 7.03 – 6.92 (m, 2H), 1.33 (s, 9H). **^{13}C NMR** (101 MHz, $CDCl_3$) δ 163.3, 149.7, 148.7, 147.7, 139.6, 130.8, 125.9, 123.2, 118.6, 116.1, 111.4, 34.7, 31.5. **IR** (neat, cm^{-1}) ν 2962, 2869, 1591, 1575, 1493, 1466, 1428, 1266, 1245. **HR-MS** (+ESI) *m/z* calculated for $C_{15}H_{17}BrNO^+$ $[M+H]^+$: 306,0489; found: 306,0488. **Purity** 95%.

Methyl 6-(4-*tert*-butylphenoxy)nicotinate (1e)

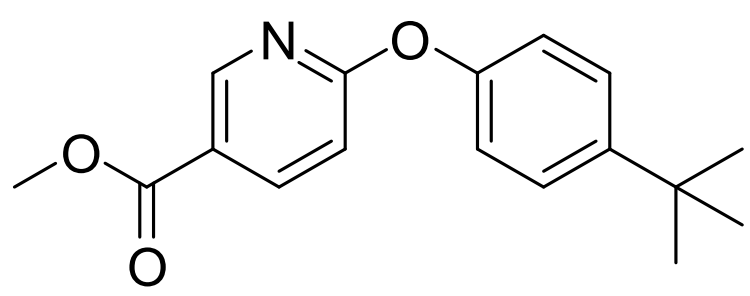

Prepared according to the general procedure for aryl bromides for 22 h at 90 °C. The crude product was purified by silica gel column chromatography (heptane:EtOAc 100:10) and prep-HPLC column chromatography (water:acetonitrile 100:0 to 100:100 gradient) to give the diaryl ether as a white solid (52 mg, 24 %).

^{1}H NMR (400 MHz, $CDCl_3$) δ 8.86 (d, *J* = 2.3 Hz, 1H), 8.28 (dd, *J* = 8.6, 2.4 Hz, 1H), 7.50 – 7.42 (m, 2H), 7.14 – 7.06 (m, 2H), 6.94 (d, *J* = 8.7 Hz, 1H), 3.93 (s, 3H), 1.37 (s, 9H). **^{13}C NMR** (101 MHz, $CDCl_3$) δ 166.7, 165.7, 151.0, 150.6, 148.2, 140.7, 126.8, 121.1, 120.8, 110.8, 52.3, 34.6, 31.6. **HR-MS** (+ESI) *m/z* calculated for $C_{17}H_{20}NO_3^+$ $[M+H]^+$: 286,1438; found: 286,1428. **Purity** 98%.

6-(4-*tert*-butylphenoxy)quinoline (4)

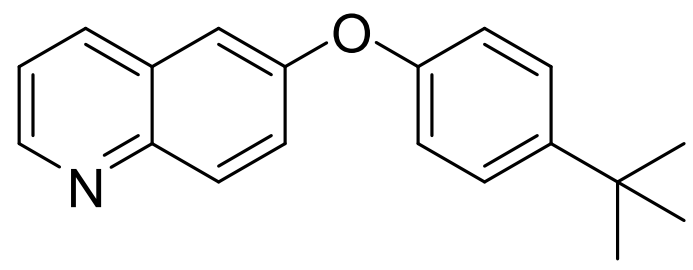

Prepared according to the general procedure for aryl bromides for 23 h at 90 °C. The crude product was purified by silica gel column chromatography (heptane:EtOAc 100:10) and prep-HPLC column chromatography (water:acetonitrile 100:0 to 100:100 gradient) to give the diarylether as a yellow solid (201 mg, 97 %).

^{1}H NMR (400 MHz, $CDCl_3$) δ 8.82 (dd, *J* = 4.2, 1.7 Hz, 1H), 8.08 (d, *J* = 9.1 Hz, 1H), 8.04 – 7.98 (m, 1H), 7.49 (dd, *J* = 9.2, 2.7 Hz, 1H), 7.45 – 7.37 (m, 2H), 7.35 (dd, *J* = 8.3, 4.2 Hz, 1H), 7.21 (d, *J* = 2.7 Hz, 1H), 7.07 – 6.99 (m, 2H), 1.35 (s, 9H). **^{13}C NMR** (101 MHz, $CDCl_3$) δ 156.1, 154.1, 149.1, 147.1, 145.2, 135.3, 131.4, 129.3, 126.9, 123.3, 121.6, 119.3, 112.5, 34.6, 31.6. **IR** (neat, cm^{-1}) ν 2867, 2960, 1508, 1464, 1224, 1177, 921, 833. **HR-MS** (+ESI) *m/z* calculated for $C_{19}H_{20}NO^{+}$ $[M+H]^{+}$: 278,1540; found: 278,1538. **Purity** 93%.

3-iodo-2-(4-*tert*-butylphenoxy)pyridine (1f)

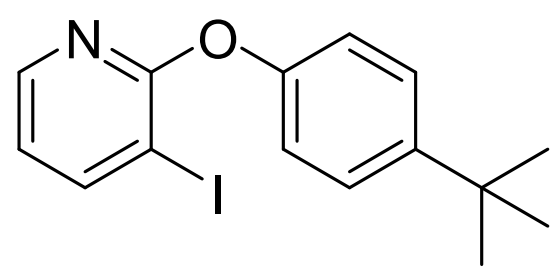

Prepared according to the general procedure for aryl iodides for 22 h at 80 °C. The crude product was purified by silica gel column chromatography (heptane:EtOAc 100:10) and prep-HPLC column chromatography (water:acetonitrile 100:0 to 100:100 gradient) to give the diaryl ether as a white solid (149 mg, 56 %).

^{1}H NMR (400 MHz, $CDCl_3$) δ 8.20 – 8.06 (m, 2H), 7.49 – 7.38 (m, 2H), 7.16 – 7.04 (m, 2H), 6.74 (dd, *J* = 7.6, 4.8 Hz, 1H), 1.35 (s, 9H). **^{13}C NMR** (101 MHz, $CDCl_3$) δ 162.1, 151.6, 149.0, 147.7, 147.1, 126.6, 120.7, 119.8, 80.6, 34.5, 31.6. **IR** (neat, cm^{-1}) ν 2960, 2865, 1573, 1508, 1409, 1281, 1242, 1204, 1169, 1014, 894, 839, 786. **HR-MS** (+ESI) *m/z* calculated for $C_{15}H_{17}INO^{+}$ $[M+H]^{+}$: 354,0351; found: 354,0348. **Purity** >99%.

2(2,3-dimethylphenoxy)-3-iodopyridine (1g)

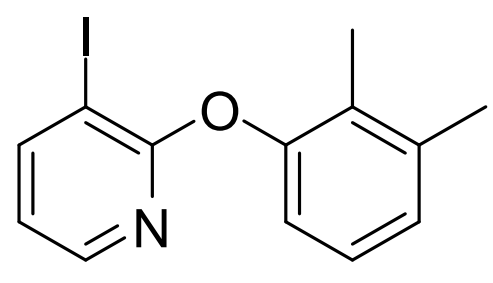

Prepared according to the general procedure for aryl iodides for 22 h at 80 °C. The crude product was purified by silica gel column chromatography (heptane:EtOAc 100:10) and prep-HPLC column chromatography (water:acetonitrile 100:0 to 100:100 gradient) to give the diaryl ether as a white solid (109 mg, 45 %).

^{1}**H NMR** (400 MHz, $CDCl_3$) δ 8.14 (dd, *J* = 7.6, 1.7 Hz, 1H), 8.05 (dd, *J* = 4.9, 1.7 Hz, 1H), 7.15 (t, *J* = 7.8 Hz, 1H), 7.06 (d, *J* = 7.5 Hz, 1H), 6.97 – 6.91 (m, 1H), 6.71 (dd, *J* = 7.6, 4.8 Hz, 1H), 2.33 (s, 3H), 2.08 (s, 3H). ^{13}**C NMR** (101 MHz, $CDCl_3$) δ 162.0, 152.2, 149.0, 147.2, 138.8, 129.3, 127.2, 126.2, 119.7, 119.5, 79.9, 20.3, 12.7. **IR** (neat, cm^{-1}) ν 3047, 2974, 2917, 2853, 1571, 1406, 1283, 1242, 1188, 1070, 1012, 1058, 783. **HR-MS** (+ESI) *m/z* calculated for $C_{13}H_{13}INO^+$ $[M+H]^+$: 326,0039; found: 326,0034. **Purity** >99%.

Methyl 2-(3-*tert*-butylphenoxy)nicotinate (1h)

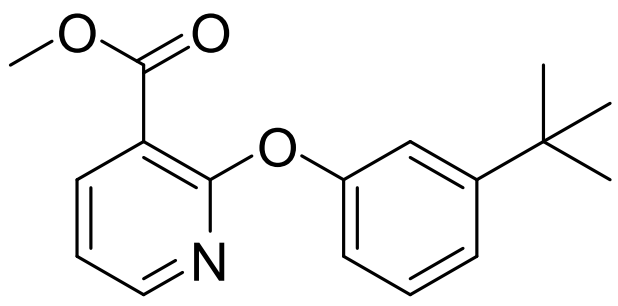

Prepared according to the general procedure for aryl bromides for 22 h at 90 °C. The crude product was purified by silica gel column chromatography (heptane:EtOAc 100:10) and prep-HPLC column chromatography (water:acetonitrile 100:0 to 100:100 gradient) to give the diarylether as a yellow solid (78 mg, 36 %).

^{1}**H NMR** (400 MHz, $CDCl_3$) δ 8.31 – 8.22 (m, 2H), 7.34 (t, *J* = 7.9 Hz, 1H), 7.25 (ddd, *J* = 7.8, 1.9, 1.1 Hz, 1H), 7.18 (t, *J* = 2.1 Hz, 1H), 7.05 (dd, *J* = 7.5, 4.9 Hz, 1H), 6.96 (ddd, *J* = 7.9, 2.4, 1.0 Hz, 1H), 3.95 (s, 3H), 1.33 (s, 9H). ^{13}**C NMR** (101 MHz, $CDCl_3$) δ 165.5, 162.0, 153.6, 153.2, 151.2, 141.7, 129.0, 122.1, 118.8, 118.6, 118.0, 115.2, 52.6, 34.9, 31.4. **IR** (neat, cm^{-1}) ν 2954, 2906, 2868, 1736, 1712, 1573, 1418, 1310, 1255, 1191, 1130, 1084, 931, 776. **HR-MS** (+ESI) *m/z* calculated for $C_{17}H_{20}NO_3^+$ $[M+H]^+$: 286,1438; found: 286,1437. **Purity** 97%.

2-(4-iodo-3,5-dimethylphenoxy)pyrazine (2a)

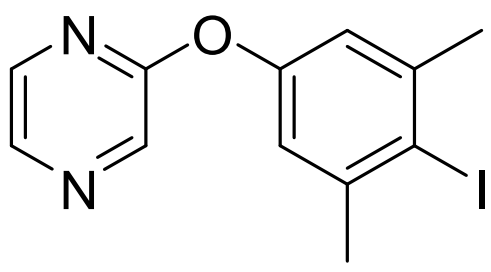

Prepared according to the general procedure for aryl iodides for 22 h at 80 °C. The crude product was purified by silica gel column chromatography (heptane:EtOAc 100:10) and prep-HPLC column

chromatography (water:acetonitrile 100:0 to 100:100 gradient) to give the diarylether as a yellow solid (90 mg, 55 %).

^{1}H NMR (400 MHz, $CDCl_3$) δ 8.42 (d, *J* = 1.4 Hz, 1H), 8.27 (d, *J* = 2.7 Hz, 1H), 8.10 (dt, *J* = 2.2, 1.1 Hz, 1H), 6.90 (s, 2H), 2.48 (s, 6H). **^{13}C NMR** (101 MHz, $CDCl_3$) δ 160.2, 152.7, 143.9, 141.3, 138.7, 136.1, 119.9, 104.0, 29.9. **IR** (neat, cm^{-1}) ν 1585, 1531, 1455, 1402, 1298, 1179, 1145, 1010. **HR-MS** (+ESI) *m/z* calculated for $C_{12}H_{12}IN_2O^+$ $[M+H]^+$: 326,9992; found: 326,9985. **Purity** 96%.

5-methoxy-2-(4-iodo-3,5-dimethylphenoxy)pyridine (1i)

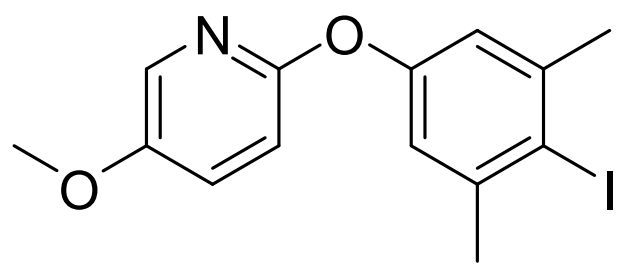

Prepared according to the general procedure for aryl iodides for 22 h at 80 °C. The crude product was purified by silica gel column chromatography (heptane:EtOAc 100:10) and prep-HPLC column chromatography (water:acetonitrile 100:0 to 100:100 gradient) to give the diaryl ether as a yellow solid (66 mg, 37%).

^{1}H NMR (400 MHz, $CDCl_3$) δ 7.90 – 7.84 (m, 1H), 7.28 (ddt, *J* = 9.3, 3.4, 1.7 Hz, 1H), 6.91 – 6.84 (m, 1H), 6.83 (s, 2H), 3.83 (s, 3H), 2.45 (s, 6H). **^{13}C NMR** (101 MHz, $CDCl_3$) δ 157.6, 154.9, 152.6, 143.5, 133.0, 126.5, 119.2, 112.6, 102.1, 56.3, 29.9. **HR-MS** (+ESI) *m/z* calculated for $C_{14}H_{15}INO_2^+$ $[M+H]^+$: 356,0144; found: 356,0133. **Purity** 96%.

3-fluoro-2-(4-tert-butylphenoxy)pyridine (1j)

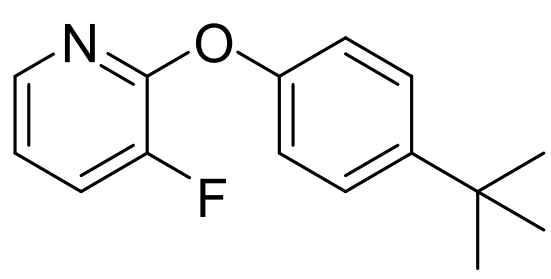

Prepared according to the general procedure for aryl bromides for 24 h at 90 °C. The crude product was purified by silica gel column chromatography (heptane:EtOAc 100:10) and prep-HPLC column chromatography (water:acetonitrile 100:0 to 100:100 gradient) to give the diaryl ether as a clear solid (47 mg, 25 %).

^{1}H NMR (400 MHz, $CDCl_3$) δ 7.96 (dd, *J* = 4.9, 1.6 Hz, 1H), 7.52 – 7.42 (m, 3H), 7.17 – 7.08 (m, 2H), 7.00 (ddd, *J* = 8.0, 4.9, 3.2 Hz, 1H), 1.36 (s, 9H). **^{13}C NMR** (101 MHz, $CDCl_3$) δ 152.7, 151.2, 148.1 (d, J^{1}_{CF} = 257 Hz), 147.7, 142.0 (d, *J* = 7 Hz), 126.7, 124.5 (d, *J* = 16 Hz), 120.5, 119.0 (d, *J* = 2 Hz), 34.6, 31.6. **IR** (neat, cm^{-1}) ν 2960, 2906, 2868, 1510, 1450, 1244, 1215, 1184, 1104, 897, 843, 794. **HR-MS** (+ESI) *m/z* calculated for $C_{15}H_{17}FNO^+$ $[M+H]^+$: 246,1290; found: 246,1286. **Purity** 97%.

2-bromo-3-(4-*tert*-butylphenoxy)pyridine (3b)

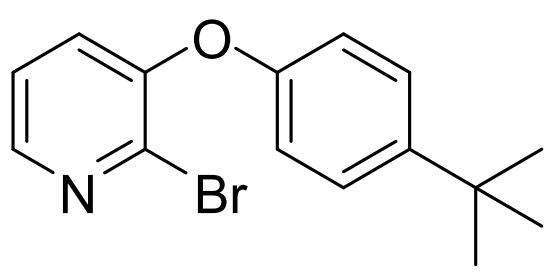

Prepared according to the general procedure for aryl bromides for 24 h at 90 °C. The crude product was purified by silica gel column chromatography (heptane:EtOAc 100:10) and prep-HPLC column chromatography (water:acetonitrile 100:0 to 100:100 gradient) to give the diaryl ether as a white solid (49 mg, 22 %).

^{1}H NMR (400 MHz, $CDCl_3$) δ 8.15 – 8.10 (m, 1H), 7.45 – 7.35 (m, 2H), 7.18 – 7.15 (m, 2H), 6.99 – 6.91 (m, 2H), 1.33 (s, 9H). **^{13}C NMR** (101 MHz, $CDCl_3$) δ 153.3, 152.0, 147.7, 144.0, 127.1, 126.1, 123.6, 118.7, 34.6, 31.6. **IR** (neat, cm^{-1}) ν 3058, 2960, 2903, 2867, 1561, 1507, 1445, 1405, 1267, 1215, 1170, 1068, 1055. **HR-MS** (+ESI) *m/z* calculated for $C_{15}H_{17}BrNO^+$ $[M+H]^+$: 308,0469; found: 308,0463. **Purity** 96%.

2-(4-*tert*-butylphenoxy)-3-methoxypyridine (1k)

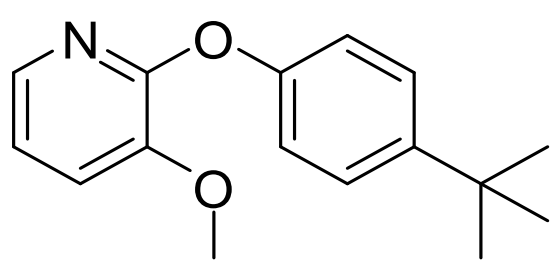

The compound has been previously reported by Londregan et al. (2011). Prepared according to the general procedure for aryl iodides for 16 h at 80 °C. The crude product was purified by silica gel column chromatography (heptane:EtOAc 100:10) to give the diaryl ether as a yellow solid (177 mg, 34 %).

^{1}H NMR (400 MHz, $CDCl_3$) δ 7.73 (dd, *J* = 5.0, 1.5 Hz, 1H), 7.42 – 7.35 (m, 2H), 7.23 (dd, *J* = 7.8, 1.5 Hz, 1H), 7.09 – 7.04 (m, 2H), 6.98 (dd, *J* = 7.9, 5.0 Hz, 1H), 3.94 (s, 3H), 1.32 (s, 9H). **^{13}C NMR** (101 MHz, $CDCl_3$) δ 153.9, 151.7, 147.4, 145.2, 137.3, 126.6, 120.5, 119.5, 119.0, 56.1, 34.5, 31.6. **HR-MS** (+ESI) *m/z* calculated for $C_{16}H_{20}NO_2^+$ $[M+H]^+$: 258,1489; found: 258,1486. **Purity** 91%. The data is consistent with literature. (Londregan et al., 2011)

2-(4-*tert*-butylphenoxy)pyridine-3-ol (1l)

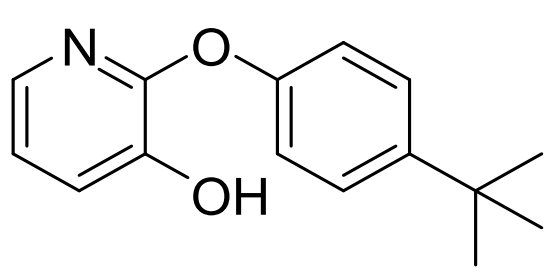

To a solution of diaryl ether **1k** (1,0 equiv.) in THF (4,9 mL/mmol) was added L-selectride 3,0 equiv.) under an argon atmosphere. The mixture turned yellow, then orange. After being refluxed and monitored by TLC (26h), the reaction mixture was quenched with MeOH and evaporated in vacuo. The

residue was purified by silical gel column chromatography (heptane:EtOAc 100:20 --> 100:50) to give the diaryl ether as a white solid (29 mg, 17 %).

^{1}H NMR (400 MHz, $CDCl_3$) δ 7.69 (dd, *J* = 5.0, 1.6 Hz, 1H), 7.43 – 7.34 (m, 2H), 7.22 (dd, *J* = 7.8, 1.7 Hz, 1H), 7.10 – 7.03 (m, 2H), 6.90 (dd, *J* = 7.8, 4.9 Hz, 1H), 6.24 (s, 1H), 1.32 (s, 9H). **^{13}C NMR** (101 MHz, $CDCl_3$) δ 152.1, 151.2, 147.6, 141.5, 137.7, 126.6, 123.0, 120.5, 119.7, 34.5, 31.5. **HR-MS** (+ESI) *m/z* calculated for $C_{15}H_{18}NO_2^+$ $[M+H]^+$: 244,1333; found: 244,1327. **Purity** 91%.

4. Spectra

5

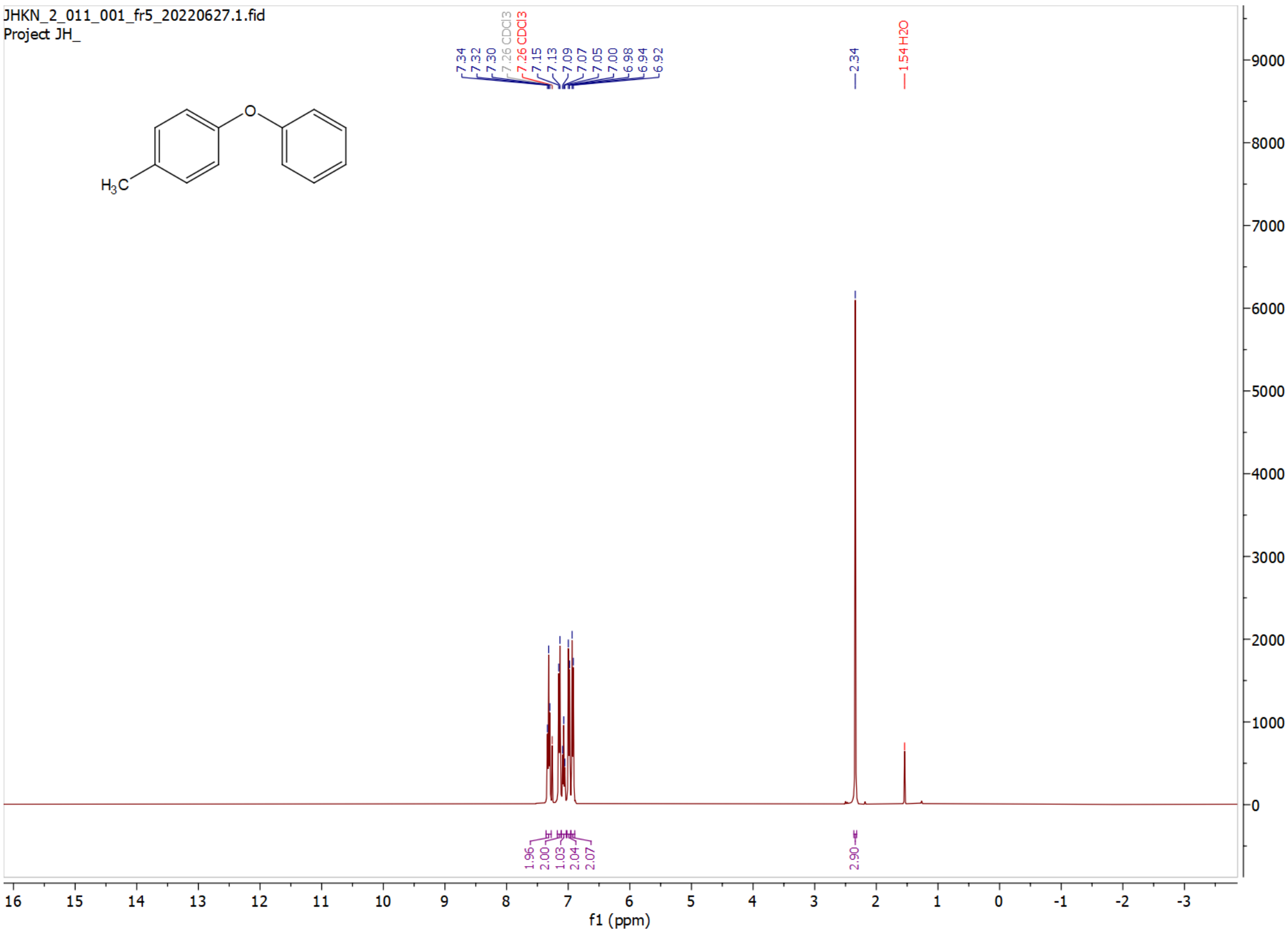
JHKN_2_011_001_fr5_20220627.1.fid
Project JH_
H3C
O
f1 (ppm)

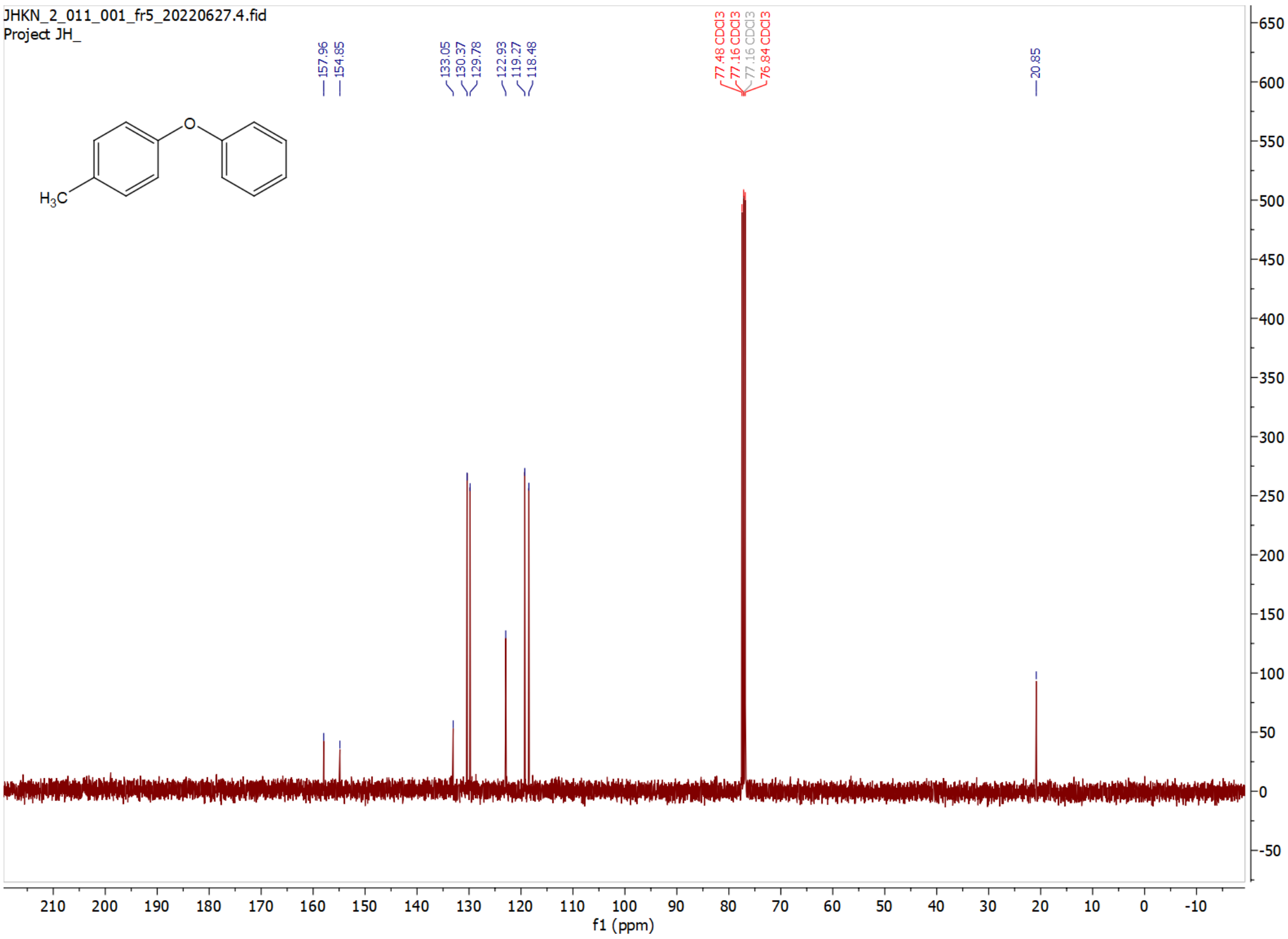
JHKN_2_011_001_fr5_20220627.4.fid
Project JH_
H3C
O
f1 (ppm)

D:\Data\Karoline\LCMS-2022-06-03\JHKN_2_011_001_fr4-6_ESI.raw 1/10/2023 11:23:17 AM

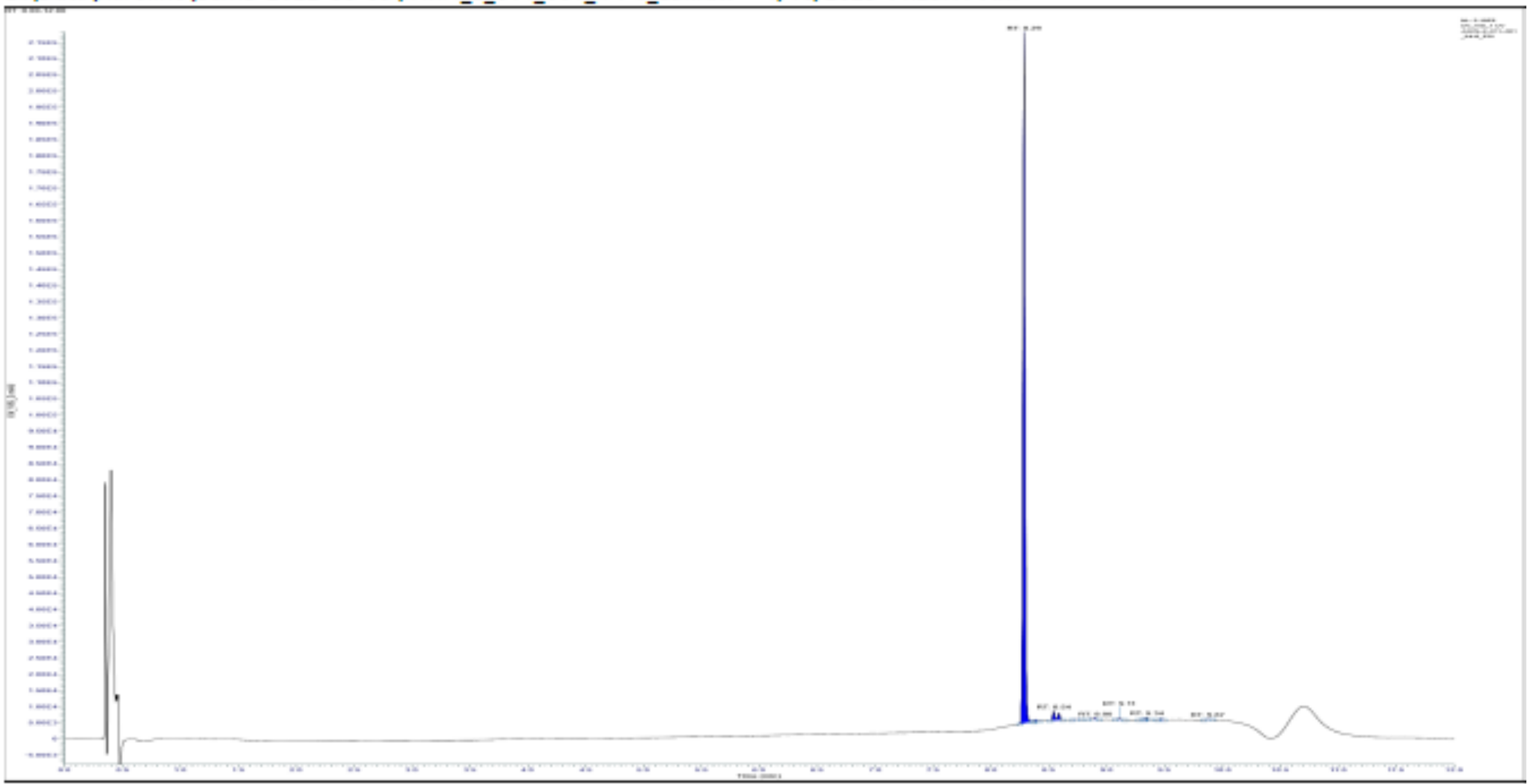

JHKN_2_011_001_fr4-6_ESI #1869 RT: 8.32 AV: 1 NL: 4.26E6
T: FTMS + c ESI Full ms [100.0000-1000.0000]

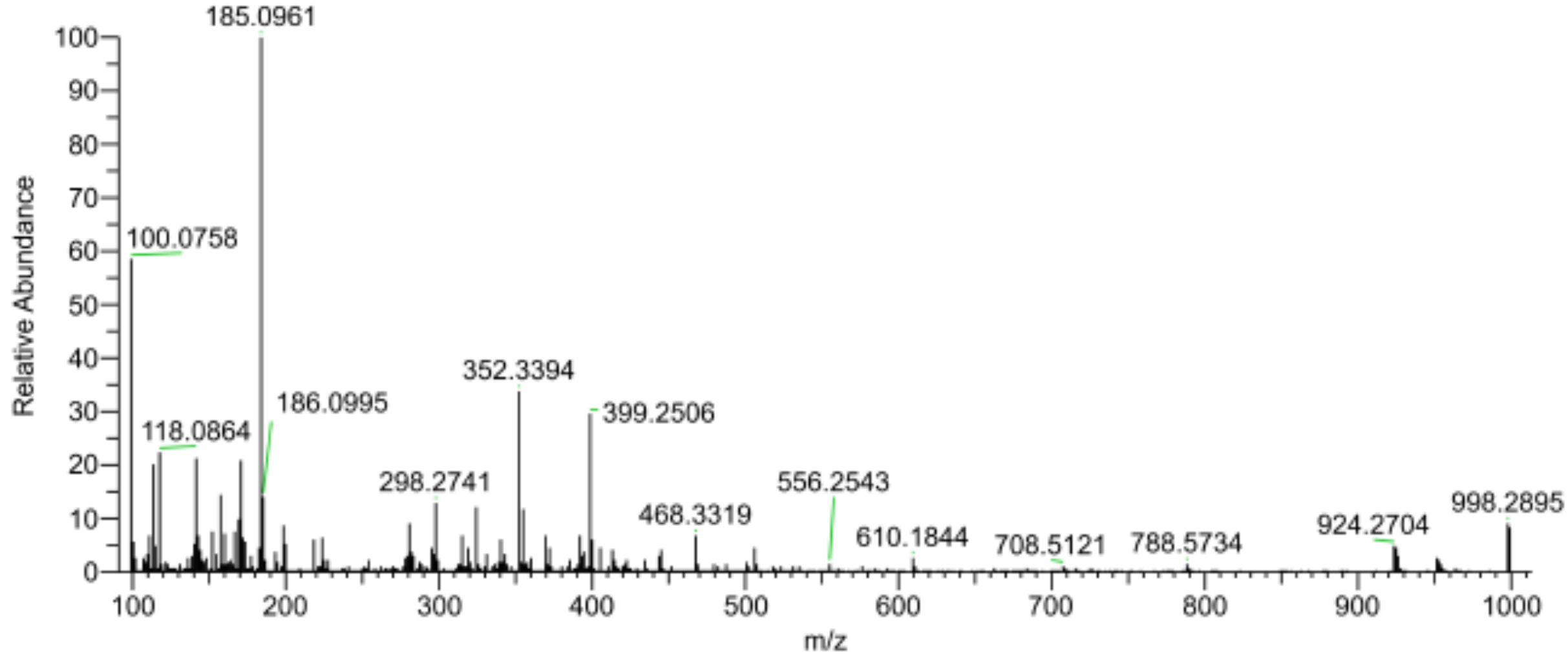

Index	% Area	RT (Mi...	RT (Sec)	Start RT	End RT	Base P...	Peak...	Peak...	Baseli...	Signal...	FileNa...	Scan F...	TraceId	% Hei...	Integr...	Name
1	95.99	8.28...	497....	8.24...	8.35...	0	2663...	2132....	0.10...	1.92E2	D:\D...	No F...	Trace1	97.39	Man...	
2	0.31	8.3834	503....	8.36...	8.41...	0	848....	702....	0.04...	6.52...	D:\D...	No F...	Trace1	0.32	Man...	
3	2.19	8.54...	512....	8.50...	8.61...	0	6075...	2731....	0.10...	2.56	D:\D...	No F...	Trace1	1.25	Man...	
4	0.19	8.72...	523....	8.69...	8.77...	0	520....	225....	0.07...	2.14...	D:\D...	No F...	Trace1	0.1	Man...	
5	0.32	8.9034	534....	8.83...	8.93...	0	884....	403....	0.09...	3.90...	D:\D...	No F...	Trace1	0.18	Man...	
6	0.24	9.11...	546....	9.06...	9.15...	0	652....	337....	0.08...	3.30...	D:\D...	No F...	Trace1	0.15	Man...	
7	0.06	9.29...	557....	9.27...	9.31...	0	170....	141....	0.04...	1.32...	D:\D...	No F...	Trace1	0.06	Man...	
8	0.32	9.3434	560....	9.32...	9.37...	0	883....	616....	0.05...	5.75...	D:\D...	No F...	Trace1	0.28	Man...	
9	0.27	9.47...	568....	9.43...	9.49...	0	736....	409....	0.05...	3.88...	D:\D...	No F...	Trace1	0.19	Man...	
10	0.13	9.8734	592....	9.83...	9.91...	0	349....	150....	0.07...	1.44...	D:\D...	No F...	Trace1	0.07	Man...	

1a

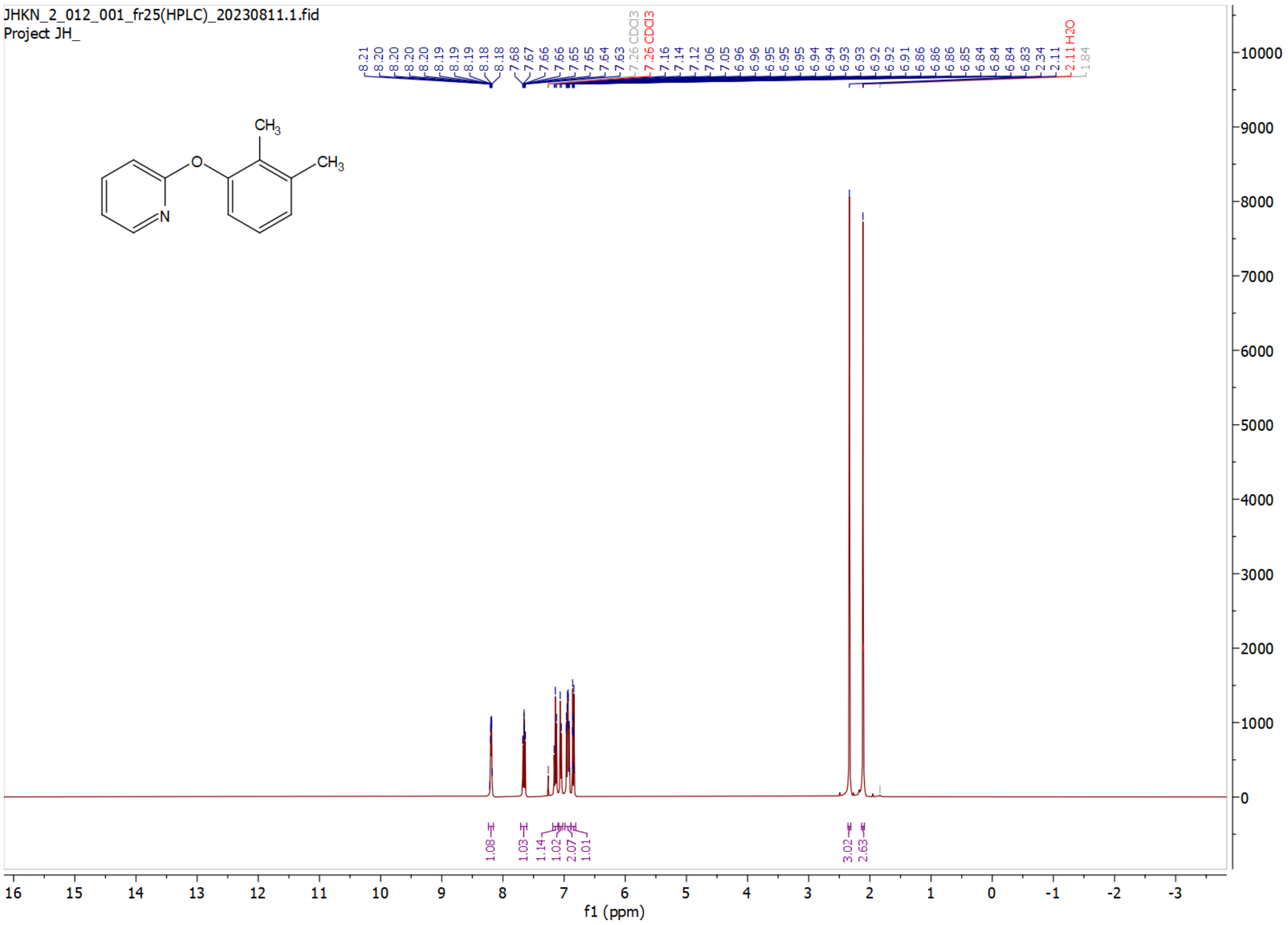

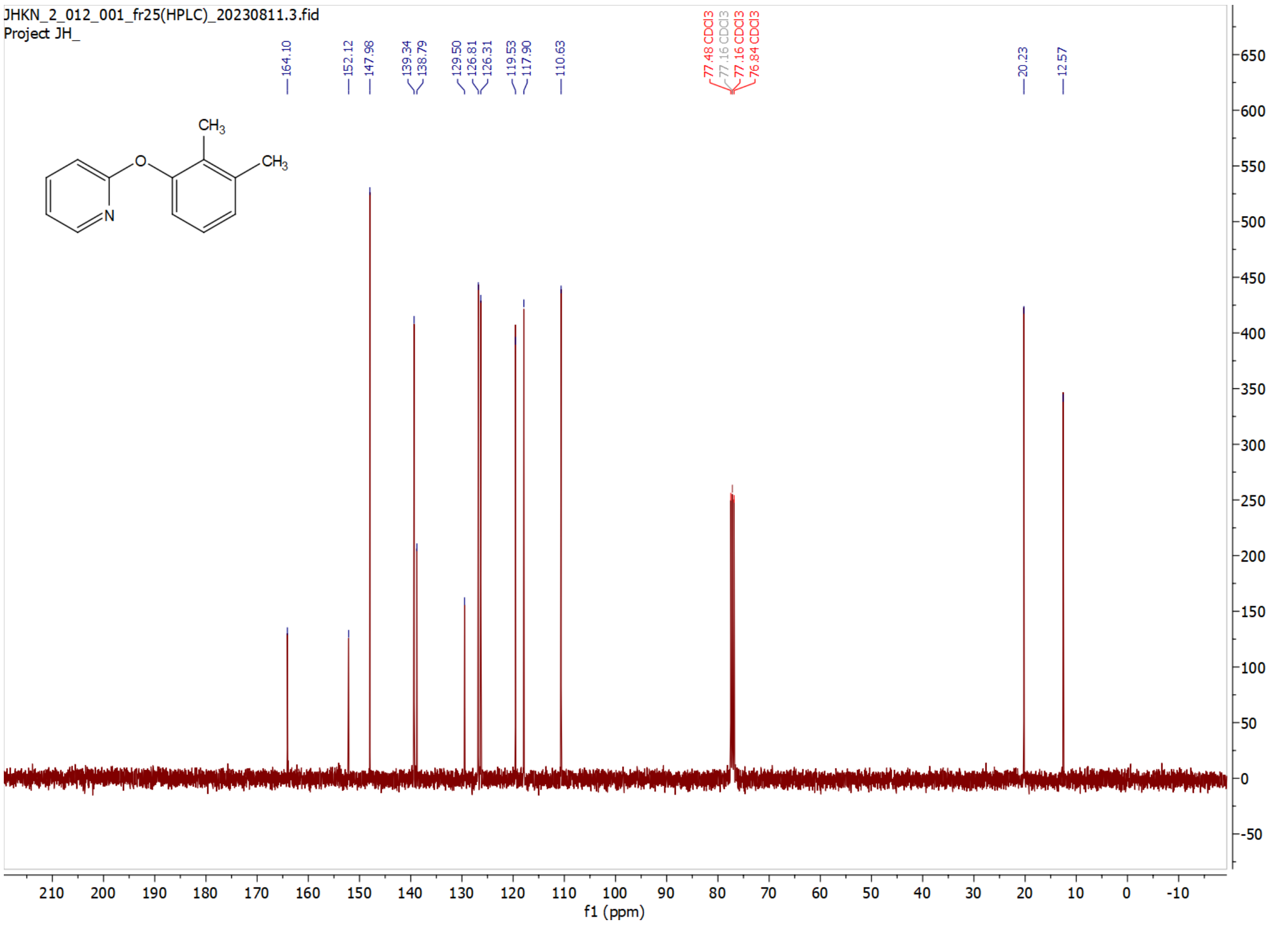

D:\Data\Karoline\LCMS-2022-06-03\JHKN_2_012_001_fr25(HPLC)_... 8/14/2023 1:28:25 PM

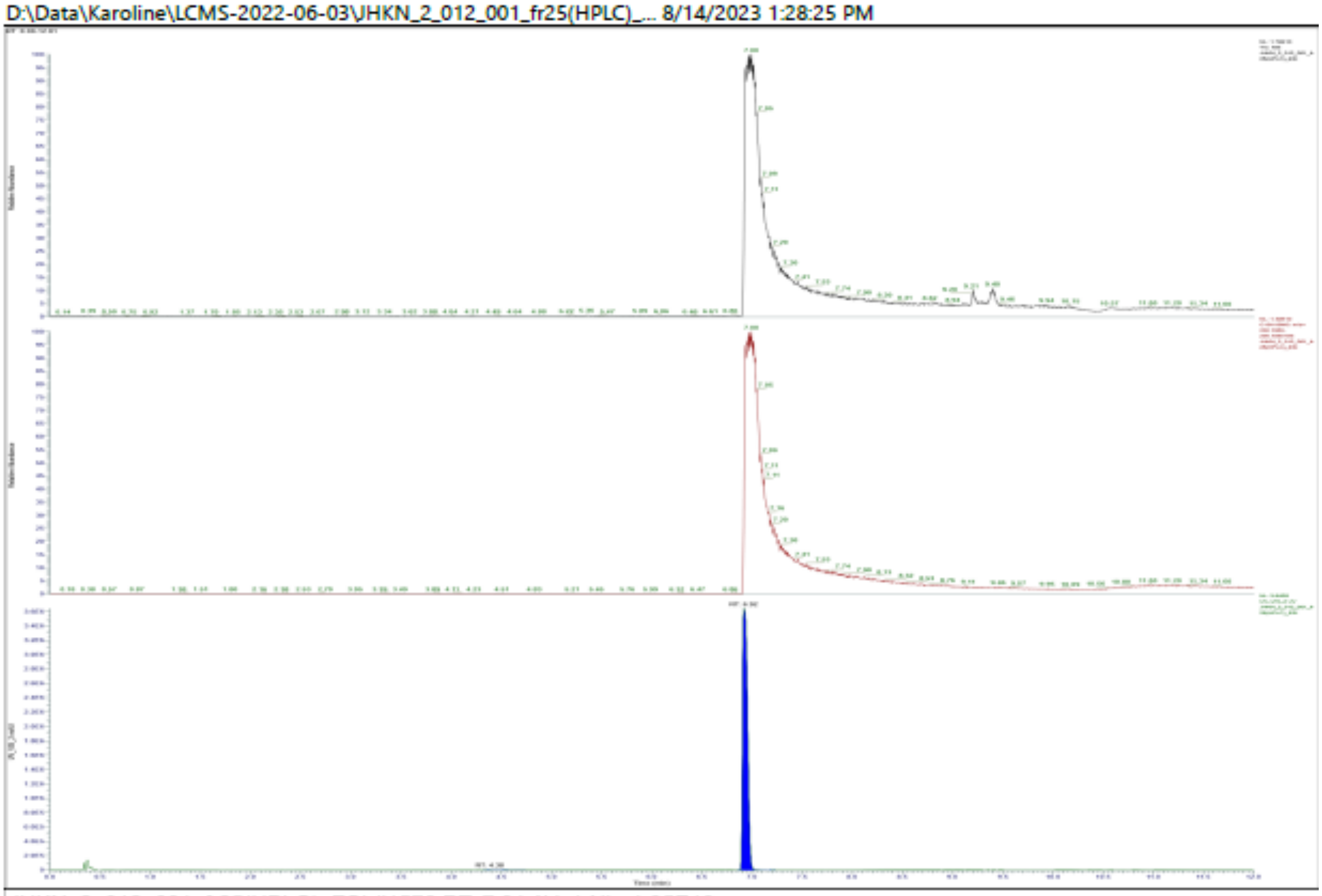

JHKN_2_012_001_fr25(HPLC)_ESI #1576 RT: 7.01 AV: 1 NL: 1.35E10
T: FTMS + c ESI Full ms [100.0000-1000.0000]

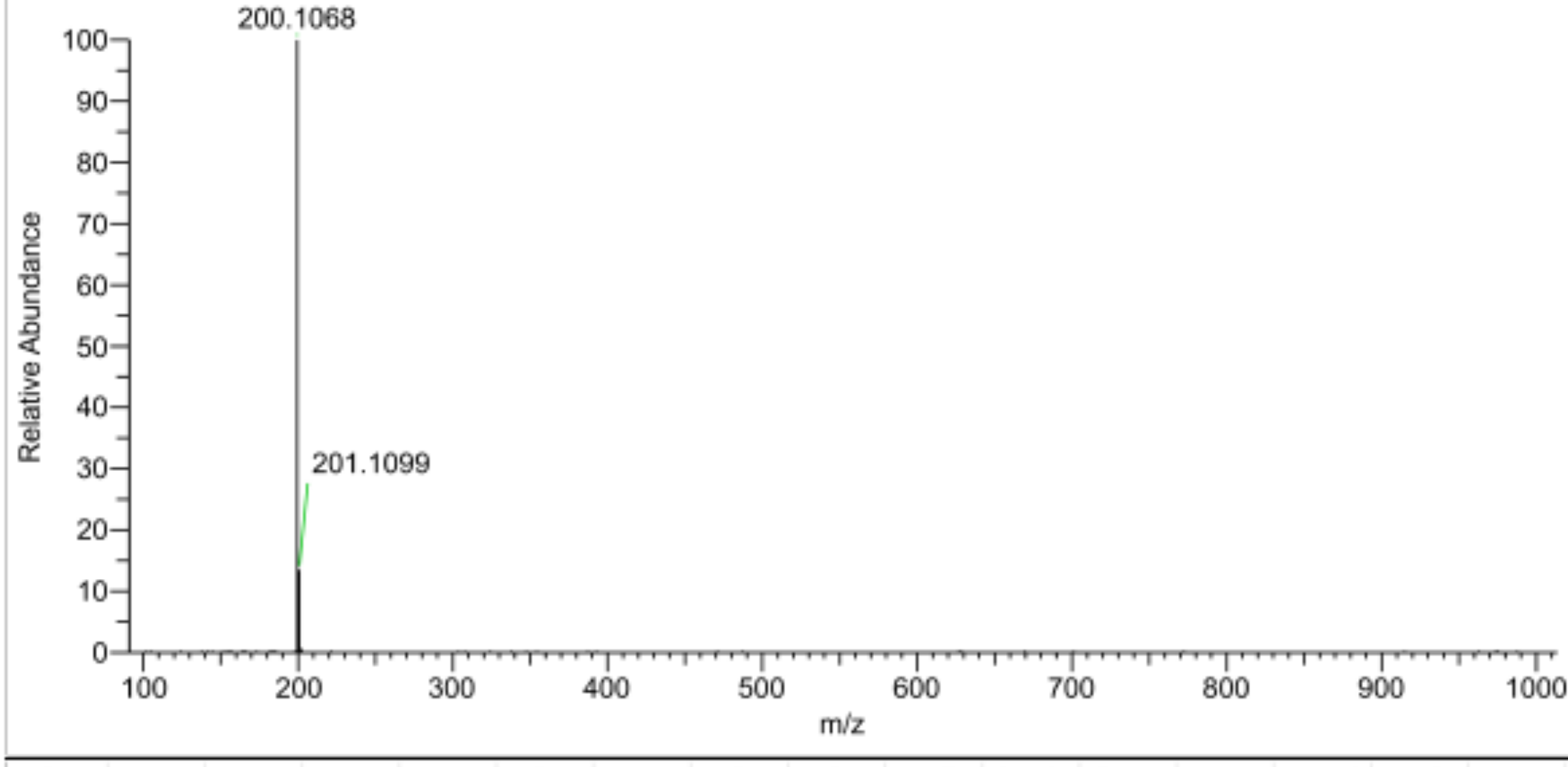

Index	% Area	RT (M...	RT (Sec)	Start RT	End RT	Base...	Peak...	Peak...	Baseli...	Signal...	FileN...	Scan...	TraceId	% Hei...	Integr...	Name
1	0.08	4.38...	262....	4.34...	4.53...	0	105...	355...	0.18...	2.69	D:\D...	No...	Trac...	0.1	Man...	
2	99.92	6.91...	415....	6.87...	7.19...	0	128...	363...	0.32...	2.73...	D:\D...	No...	Trac...	99.9	Man...	

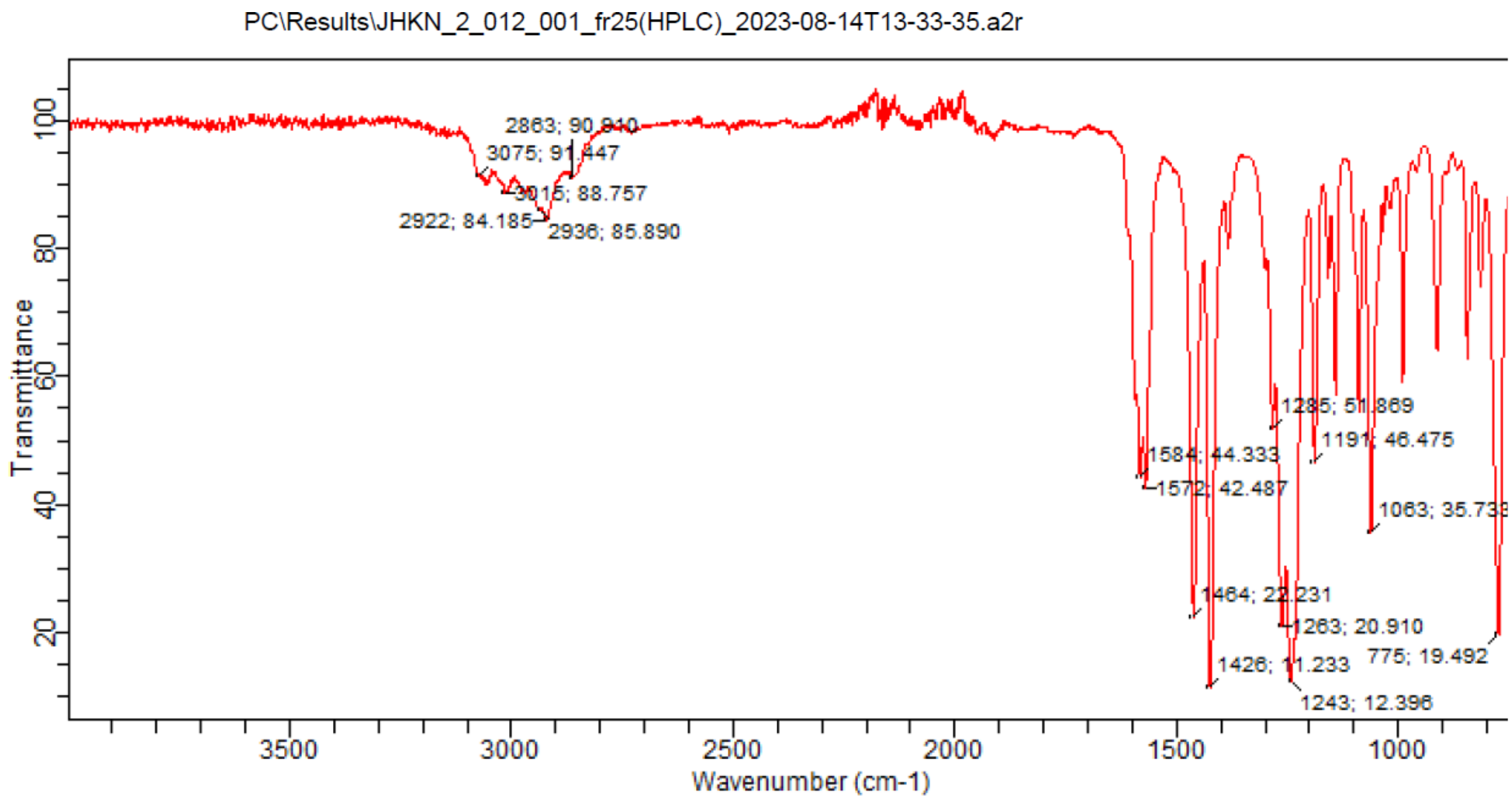
PC\Results\JHKN_2_012_001_fr25(HPLC)_2023-08-14T13-33-35.a2r
2863; 90.910
3075; 91.447
3015; 88.757
2922; 84.185
2936; 85.890
1285; 51.869
1191; 46.475
1584; 44.333
1572; 42.487
1063; 35.733
1464; 22.231
1263; 20.910
1426; 11.233
775; 19.492
1243; 12.396
Transmittance
Wavenumber (cm-1)

3a

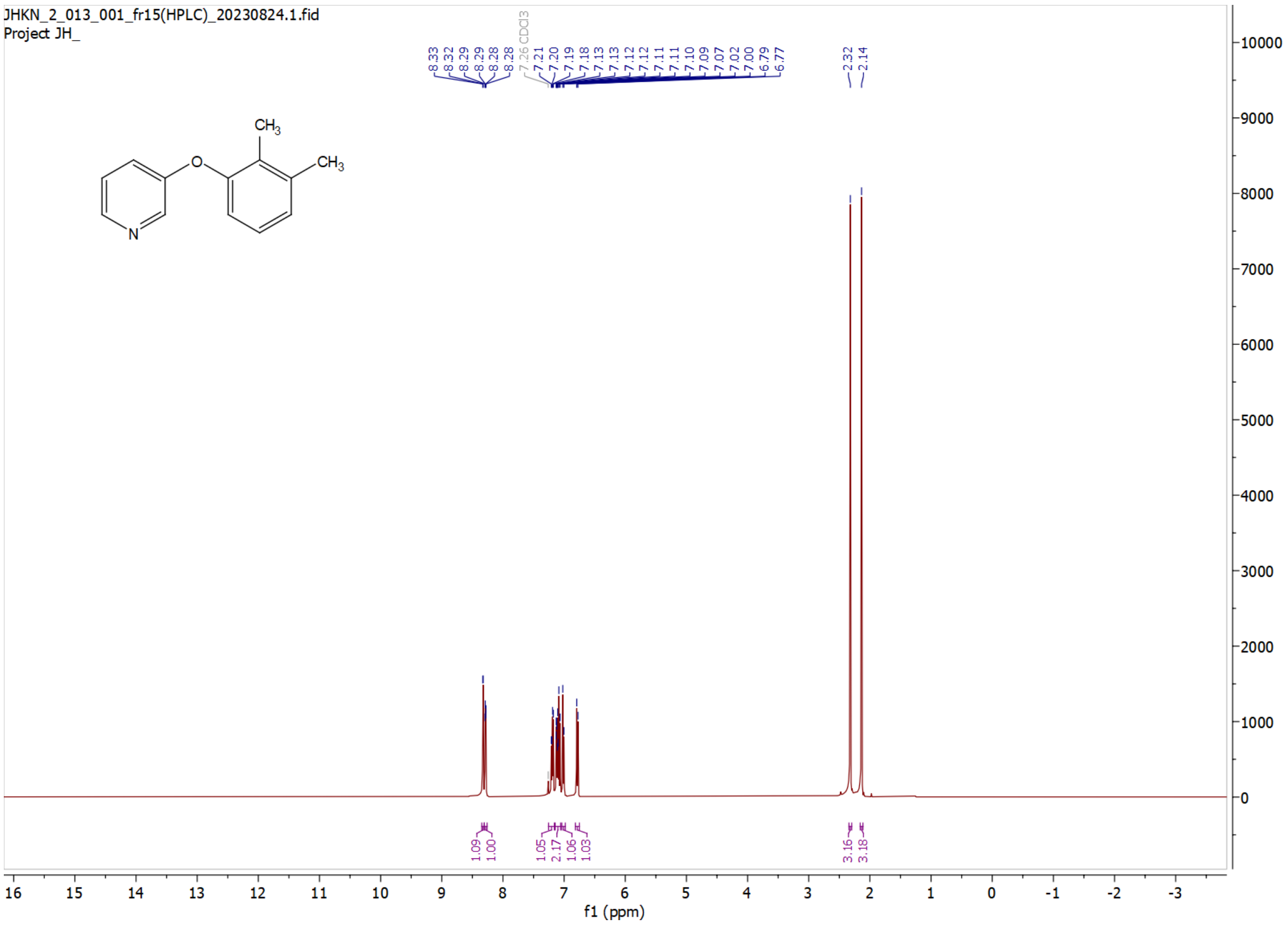
JHKN_2_013_001_fr15(HPLC)_20230824.1.fid
Project JH_
8.33
8.32
8.29
8.29
8.28
8.28
7.26 CDCl3
7.21
7.20
7.19
7.18
7.13
7.13
7.12
7.12
7.11
7.11
7.10
7.09
7.07
7.02
7.00
6.79
6.77
2.32
2.14
CH3
CH3
O
N
1.09
1.00
1.05
2.17
1.06
1.03
3.16
3.18
f1 (ppm)

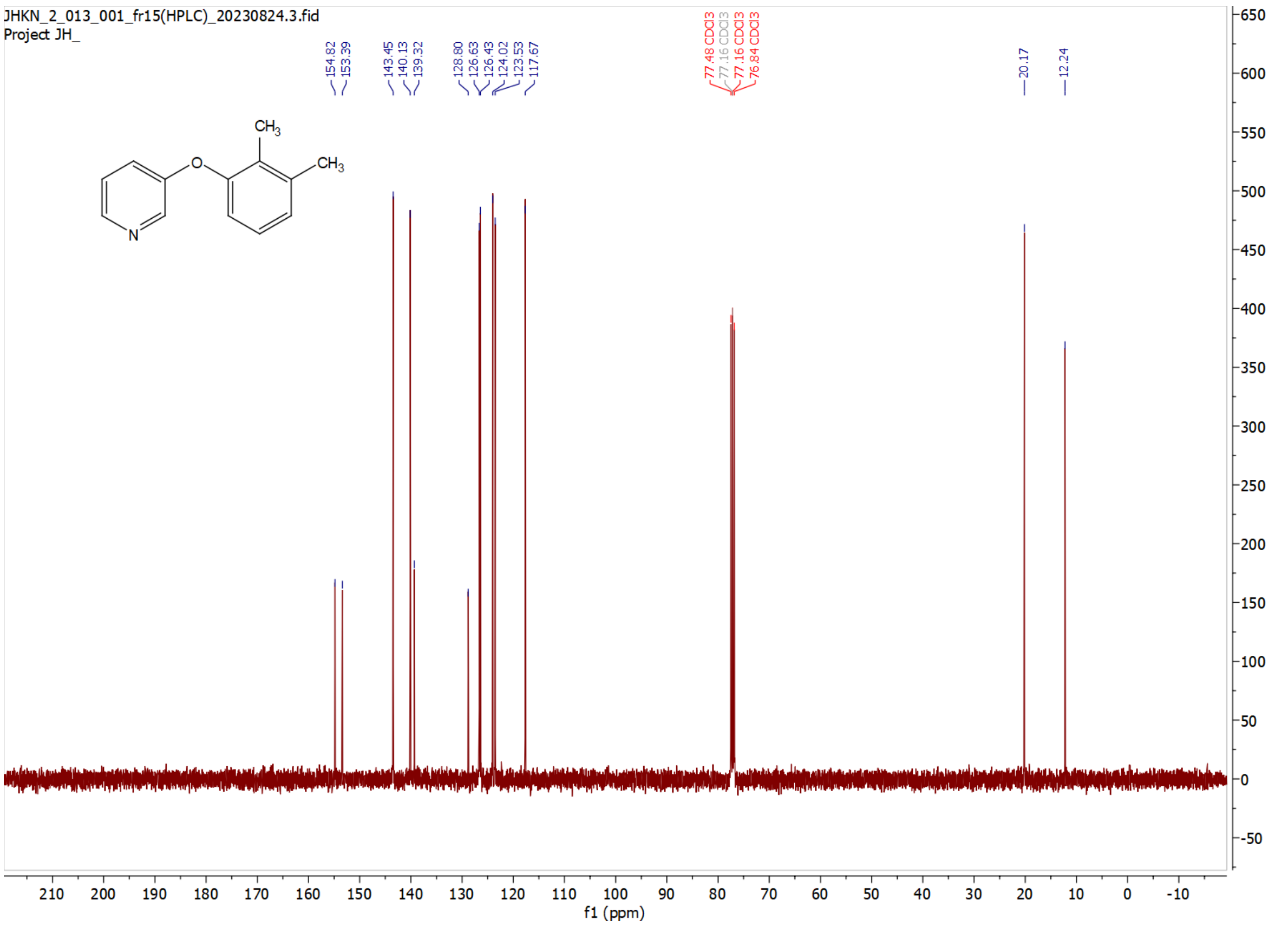
JHKN_2_013_001_fr15(HPLC)_20230824.3.fid
Project JH_
154.82
153.39
143.45
140.13
139.32
128.80
126.63
126.43
124.02
123.53
117.67
77.48 CDCl3
77.16 CDCl3
77.16 CDCl3
76.84 CDCl3
20.17
12.24
CH3
CH3
O
N
f1 (ppm)

D:\Data\Karoline\LCMS-2022-06-03\JHKN_2_013_001_fr41_ESI.raw 1/10/2023 11:32:28 AM

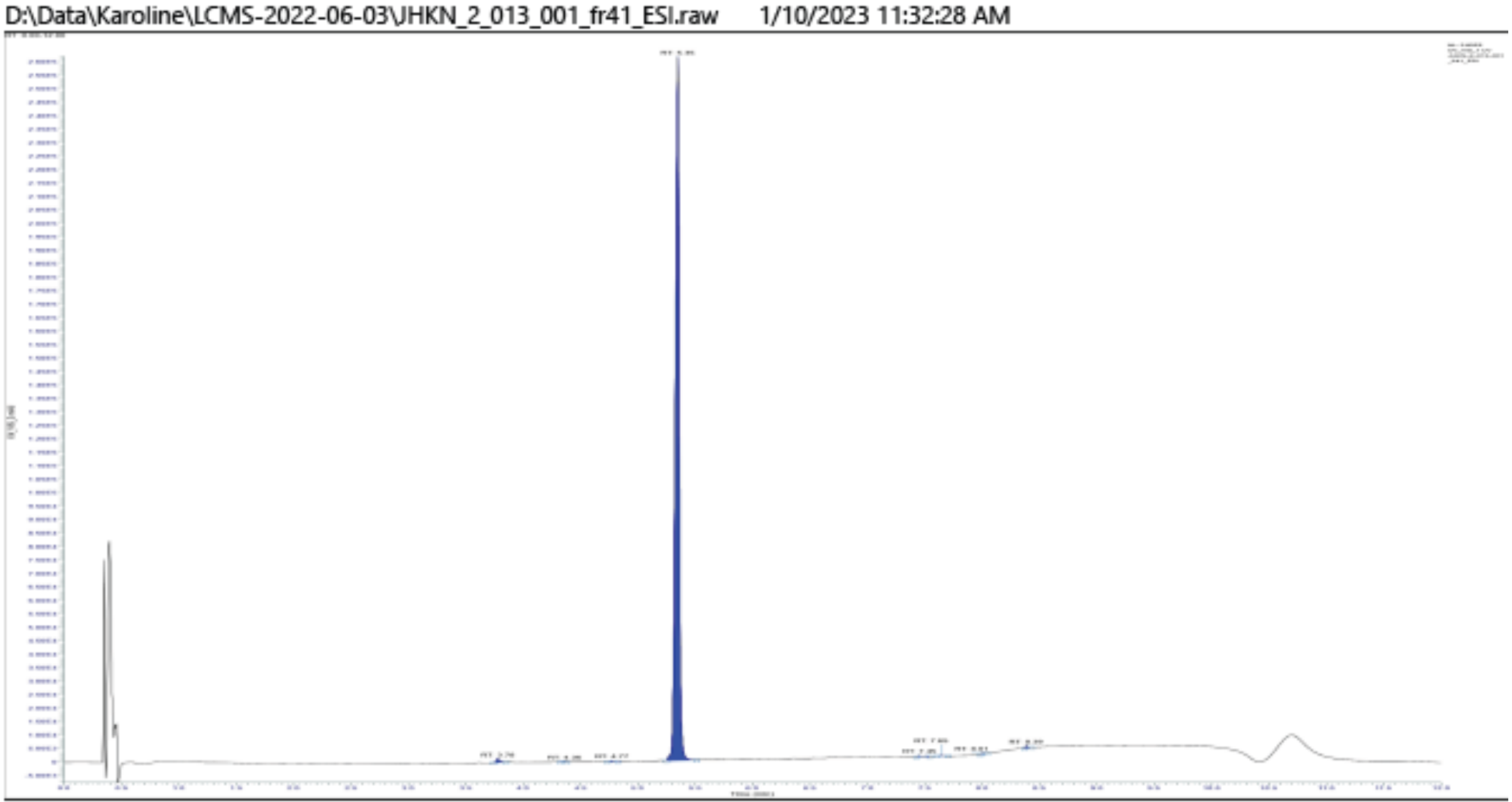

JHKN_2_013_001_fr41_ESI #1199 RT: 5.34 AV: 1 NL: 2.44E9
T: FTMS + c ESI Full ms [100.0000-1000.0000]

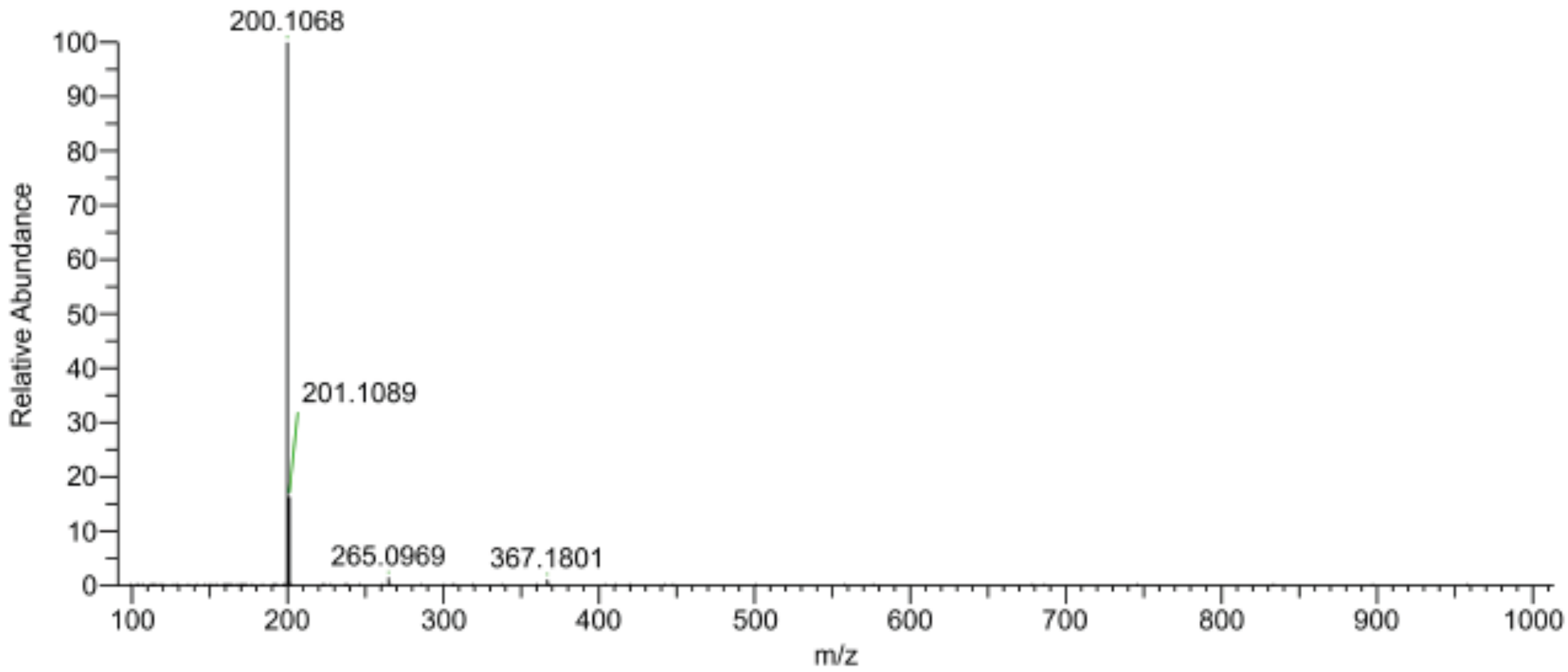

Index	% Area	RT (Mi...	RT (Sec)	Start RT	End RT	Base P...	Peak...	Peak...	Baseli...	Signal...	FileNa...	Scan F...	TraceId	% Hei...	Integr...	Name
1	0.51	3.77...	226....	3.73...	3.85...	0	3034...	1299...	0.11...	1.16	D:\D...	No F...	Trace1	0.49	Man...	
2	0.08	4.35...	261....	4.31...	4.37...	0	490....	215....	0.06...	1.94...	D:\D...	No F...	Trace1	0.08	Man...	
3	0.1	4.77...	286....	4.73...	4.81...	0	600....	336....	0.08...	3.03...	D:\D...	No F...	Trace1	0.13	Man...	
4	98.77	5.34...	320....	5.24...	5.50...	0	5908...	2613...	0.26...	2.34E2	D:\D...	No F...	Trace1	98.62	Man...	
5	0.19	7.45...	447....	7.42...	7.53...	0	1110...	398....	0.11...	3.58...	D:\D...	No F...	Trace1	0.15	Man...	
6	0.07	7.65...	459....	7.60...	7.69...	0	410....	233....	0.09...	2.10...	D:\D...	No F...	Trace1	0.09	Man...	
7	0.1	8.00...	480....	7.98...	8.03...	0	590....	373....	0.05...	3.36...	D:\D...	No F...	Trace1	0.14	Man...	
8	0.19	8.38...	503....	8.36...	8.42...	0	1141...	800....	0.06...	7.23...	D:\D...	No F...	Trace1	0.3	Man...	

2b

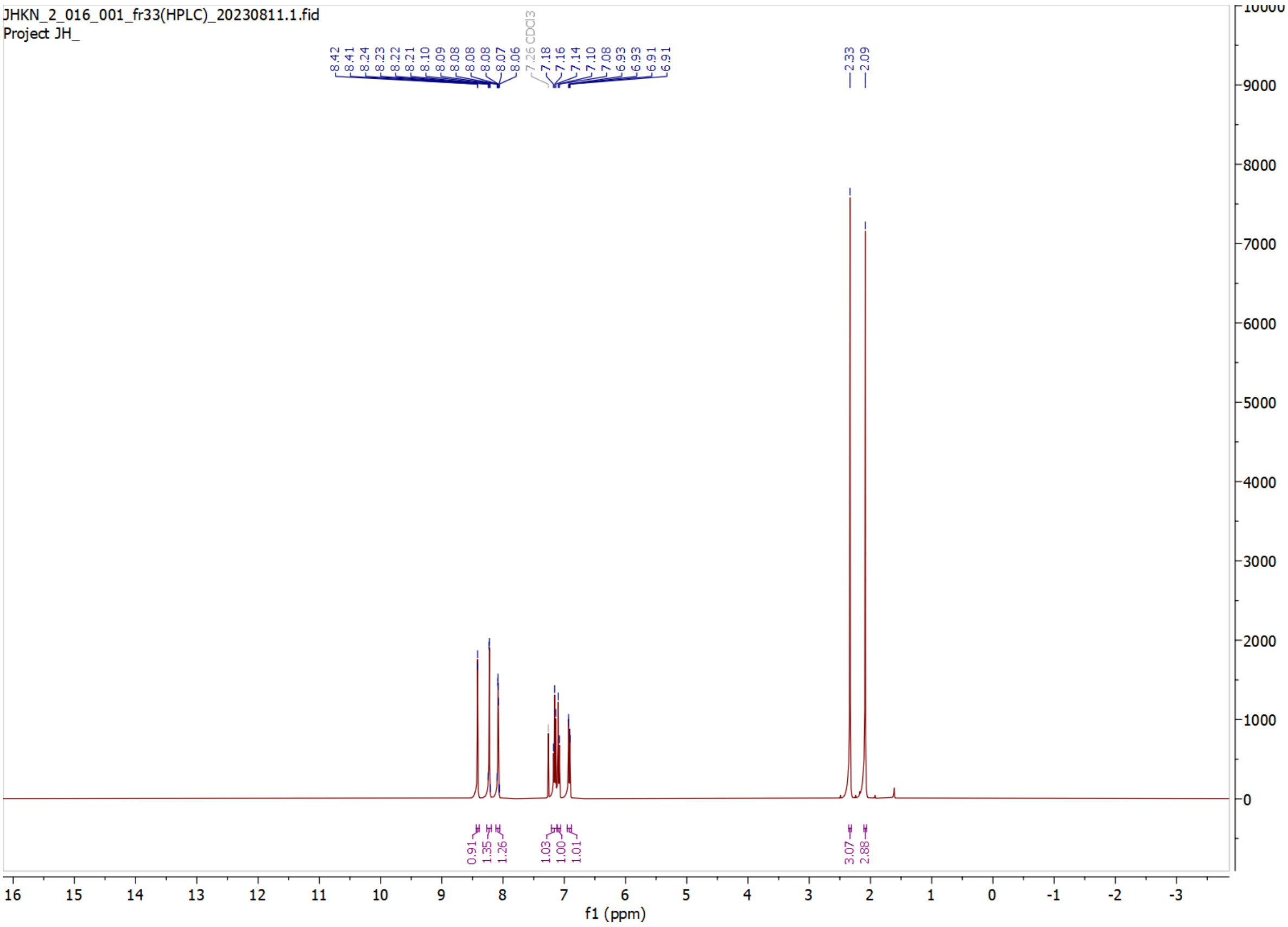
JHKN_2_016_001_fr33(HPLC)_20230811.1.fid
Project JH_
8.42 8.41 8.24 8.23 8.22 8.21 8.10 8.09 8.08 8.08 8.08 8.07 8.06
7.26 CDCl3
7.18 7.16 7.14 7.10 7.08 6.93 6.93 6.91 6.91
2.33
2.09
0.91 1.35 1.26 1.03 1.00 1.01 3.07 2.88
f1 (ppm)

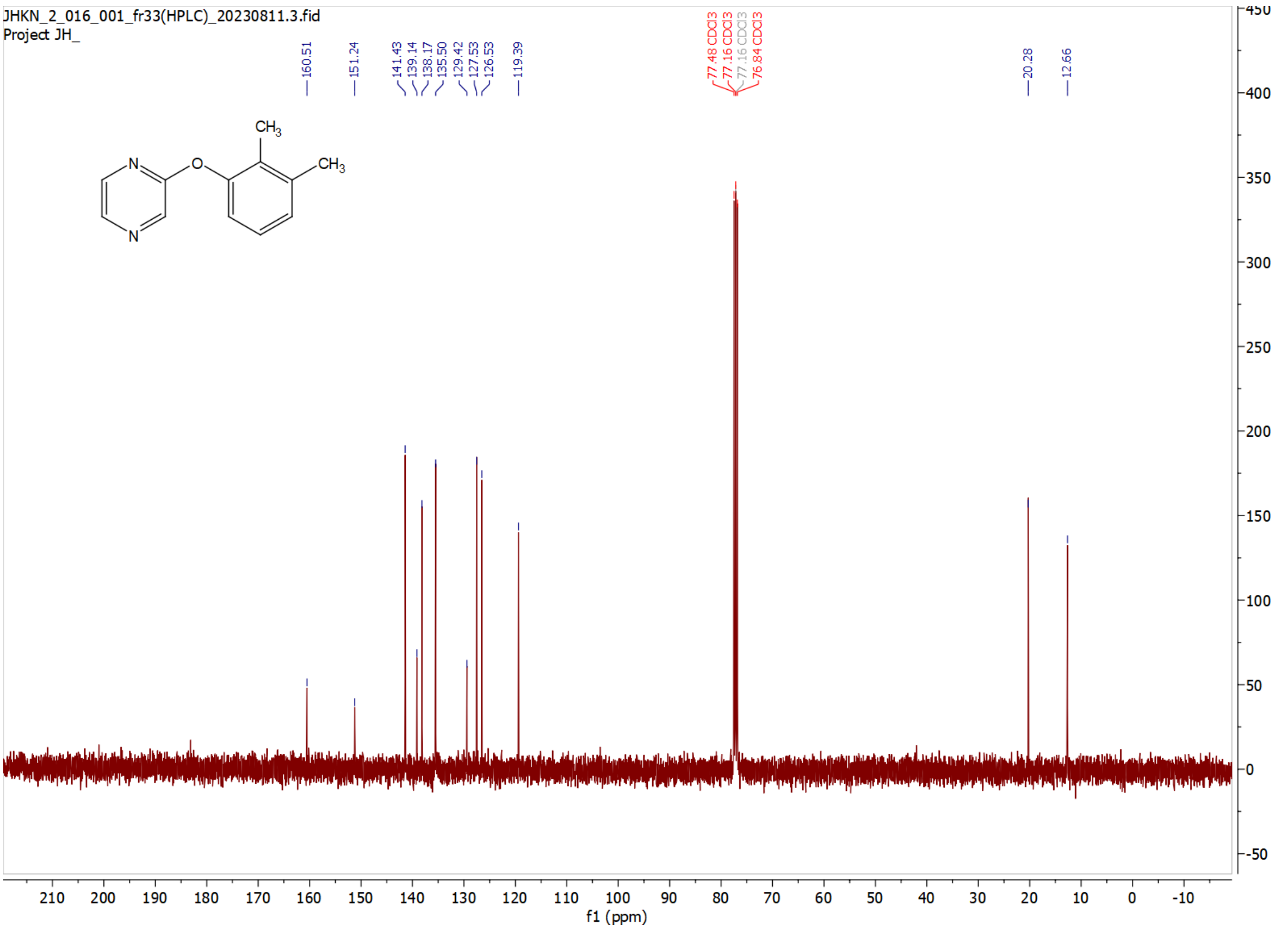
JHKN_2_016_001_fr33(HPLC)_20230811.3.fid
Project JH_
160.51
151.24
141.43 139.14 138.17 135.50 129.42 127.53 126.53
119.39
77.48 CDCl3
77.16 CDCl3
77.16 CDCl3
76.84 CDCl3
20.28
12.66
f1 (ppm)

D:\Data\Karoline\LCMS-2022-06-03\JHKN_2_016_001_fr15_ESI_2023... 1/10/2023 1:54:04 PM

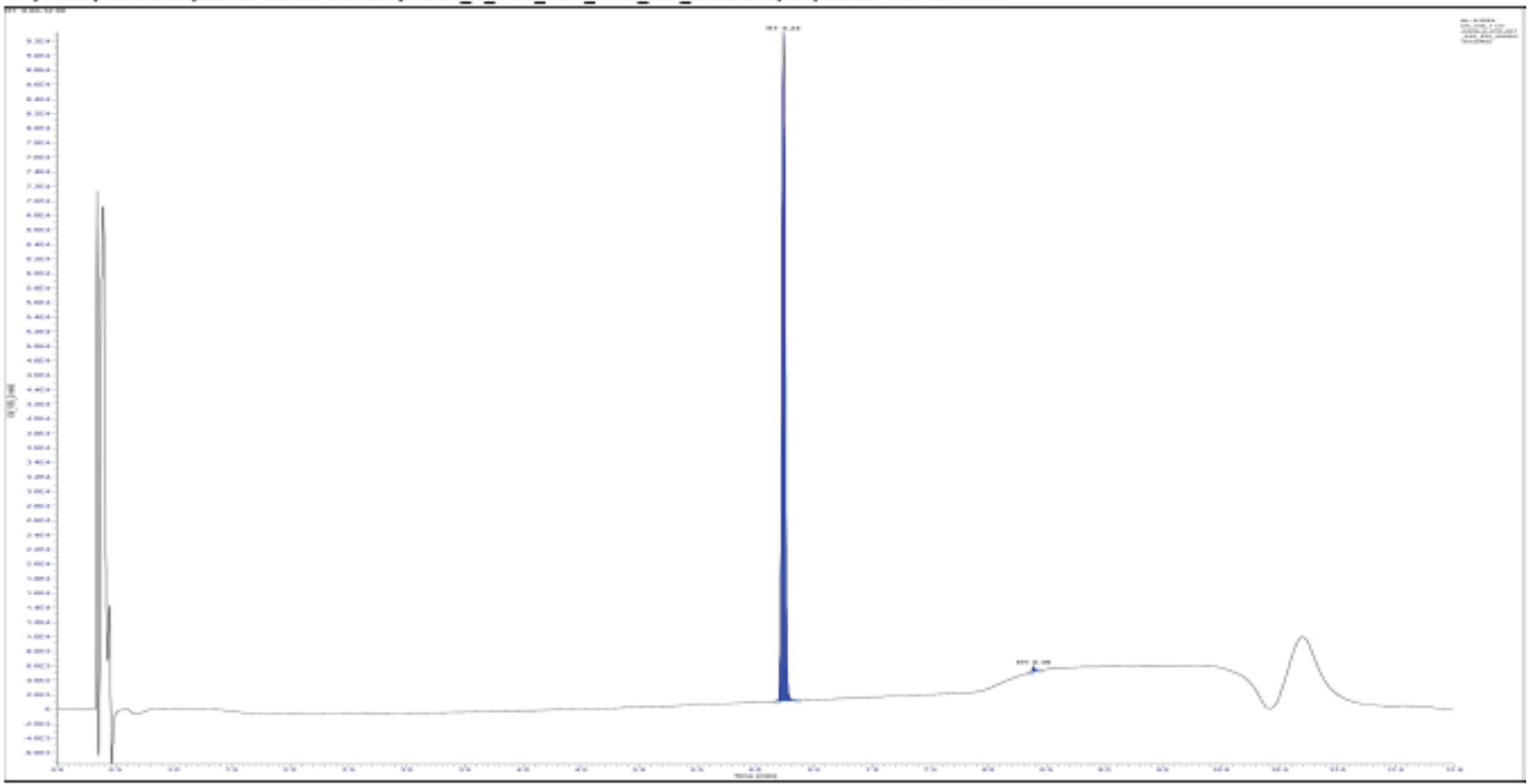

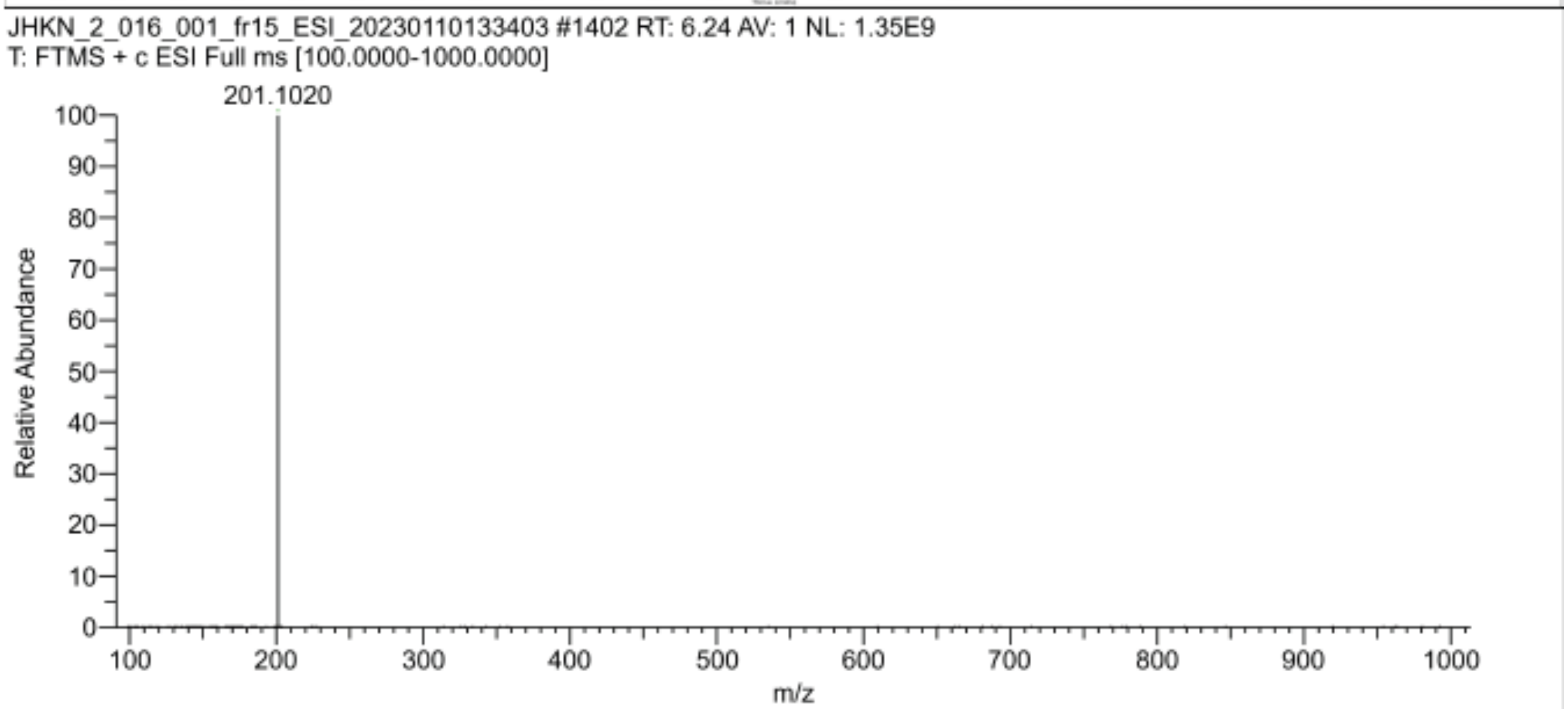

Index	% Area	RT (Mi...	RT (Sec)	Start RT	End RT	Base P...	Peak...	Peak...	Baseli...	Signal...	FileNa...	Scan F...	TraceId	% Hei...	Integr...	Name
1	99.44	6.2384	374....	6.17...	6.35...	0	1695...	9208...	0.18...	8.11E1	D:\D...	No F...	Trace1	99.22	Man...	
2	0.56	8.38...	503....	8.35...	8.43...	0	954....	728....	0.07...	6.54...	D:\D...	No F...	Trace1	0.78	Man...	

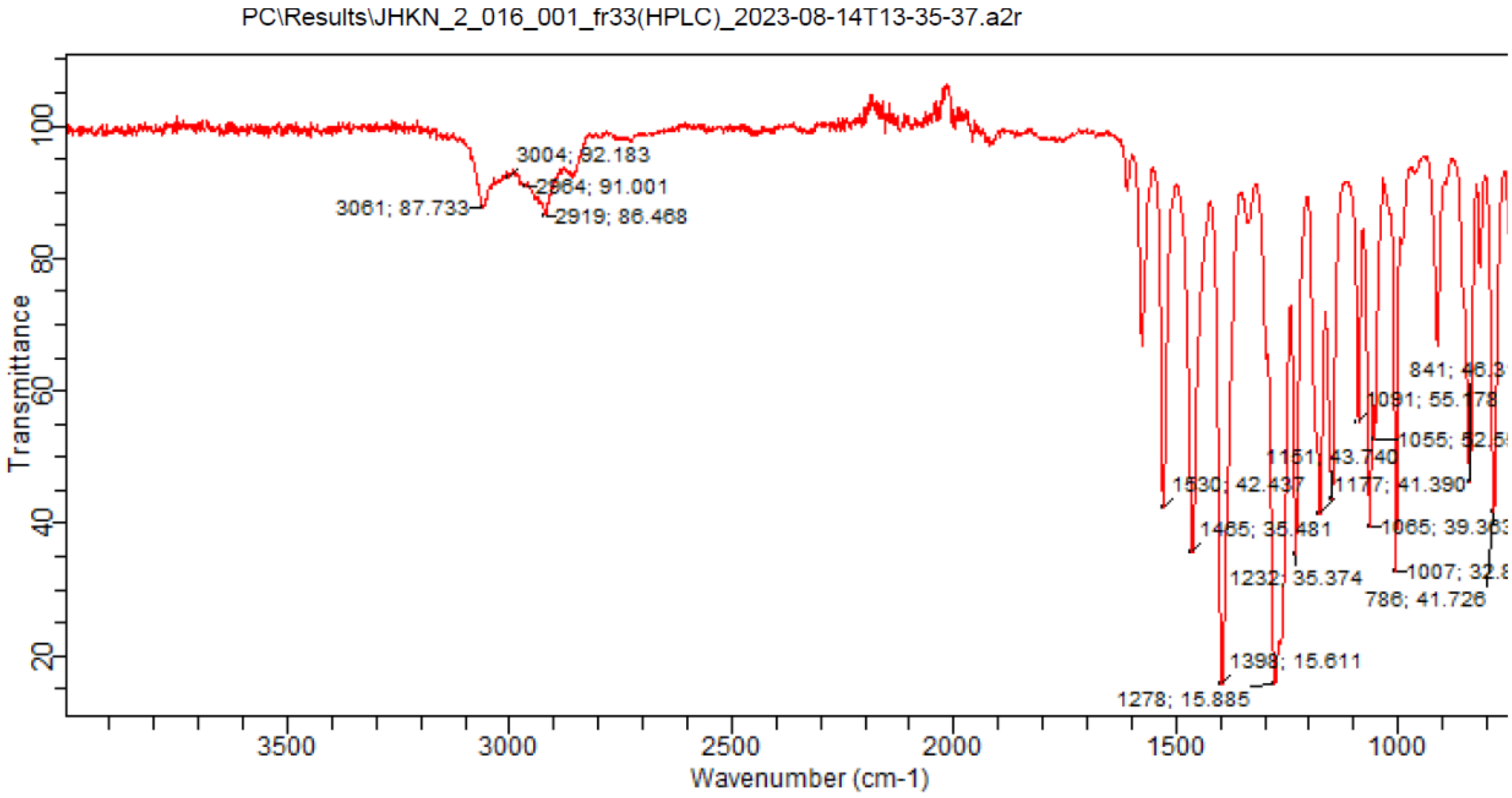

PC\Results\JHKN_2_016_001_fr33(HPLC)_2023-08-14T13-35-37.a2r
Transmittance
Wavenumber (cm-1)
3061; 87.733
3004; 92.183
2964; 91.001
2919; 86.468
1530; 42.437
1465; 35.481
1398; 15.611
1278; 15.885
1232; 35.374
1151; 43.740
1177; 41.390
1091; 55.178
1065; 39.363
1055; 52.58
1007; 32.8
841; 46.3
786; 41.726

2c

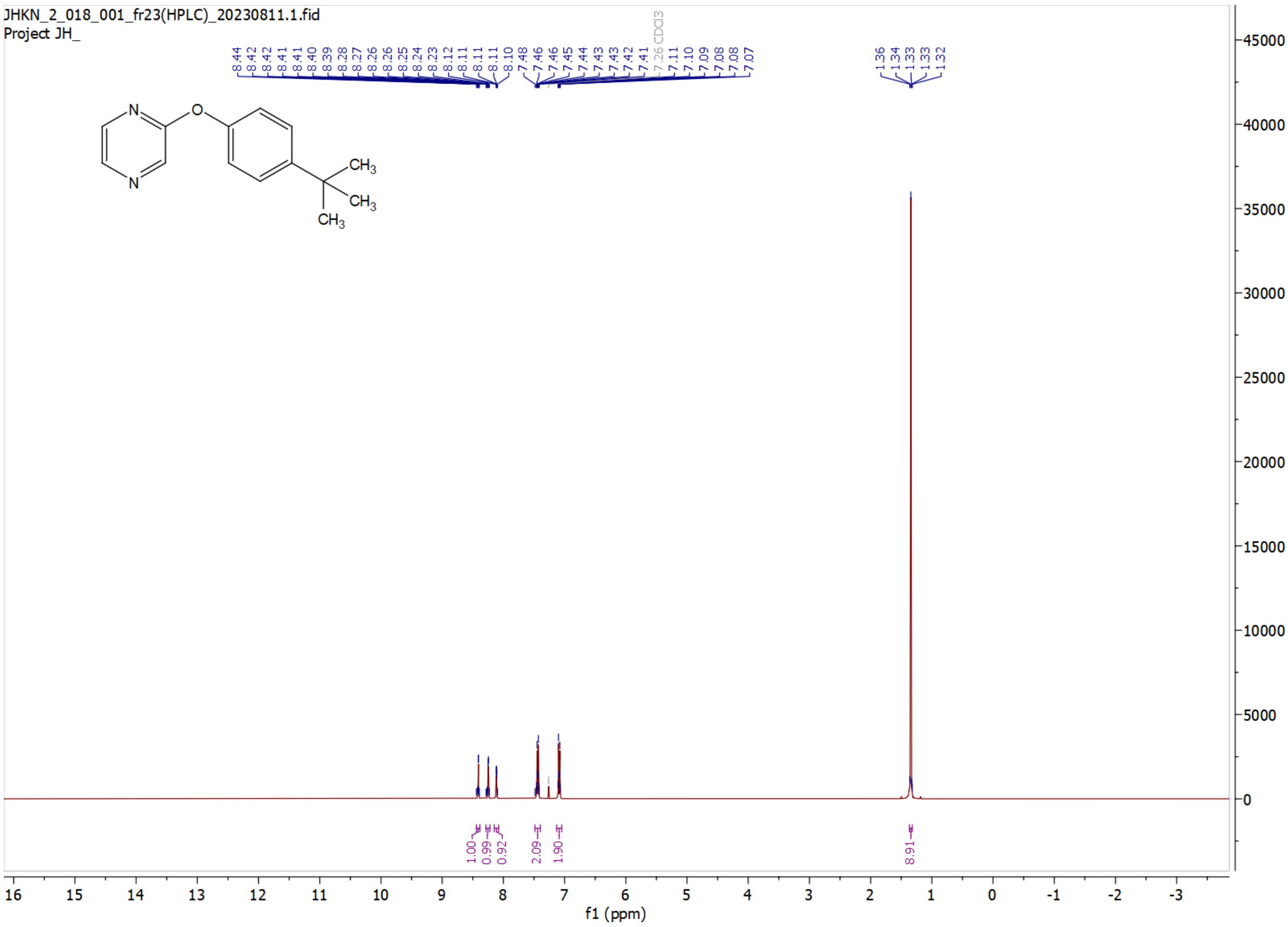
JHKN_2_018_001_fr23(HPLC)_20230811.1.fid
Project JH_
8.44
8.42
8.42
8.41
8.41
8.40
8.39
8.28
8.27
8.26
8.26
8.25
8.24
8.23
8.12
8.11
8.11
8.11
8.10
7.48
7.46
7.45
7.44
7.43
7.43
7.42
7.41
7.26 CDCl3
7.11
7.10
7.09
7.08
7.08
7.07
1.36
1.34
1.33
1.33
1.32
N
O
N
CH3
CH3
CH3
1.00
0.99
0.92
2.09
1.90
8.91
f1 (ppm)

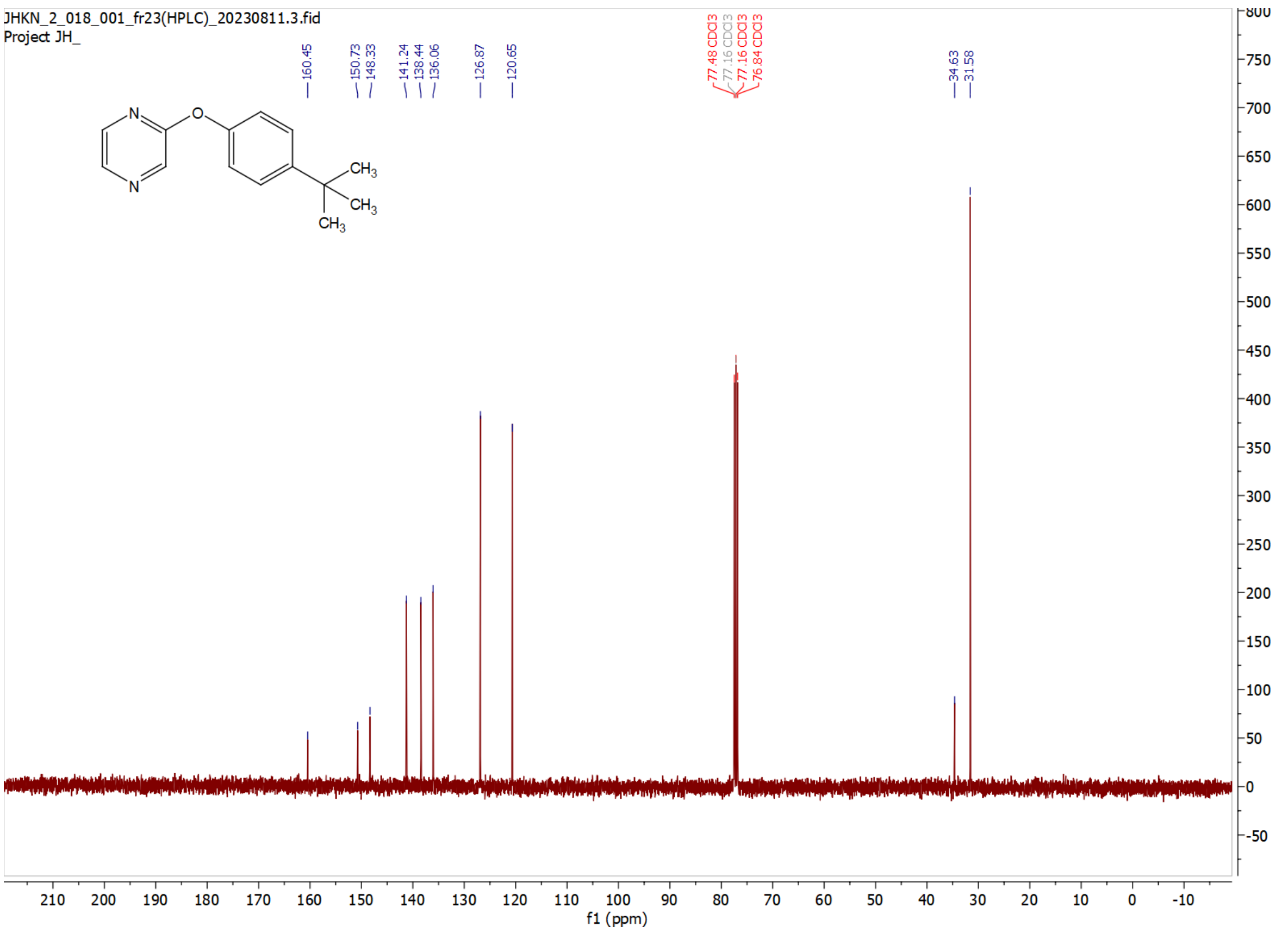
JHKN_2_018_001_fr23(HPLC)_20230811.3.fid
Project JH_
160.45
150.73
148.33
141.24
138.44
136.06
126.87
120.65
77.48 CDCl3
77.16 CDCl3
77.16 CDCl3
76.84 CDCl3
34.63
31.58
N
O
N
CH3
CH3
CH3
f1 (ppm)

D:\Data\Karoline\LCMS-2022-06-03\JHKN_2_018_001_fr15_ESI_2023... 1/10/2023 2:01:51 PM

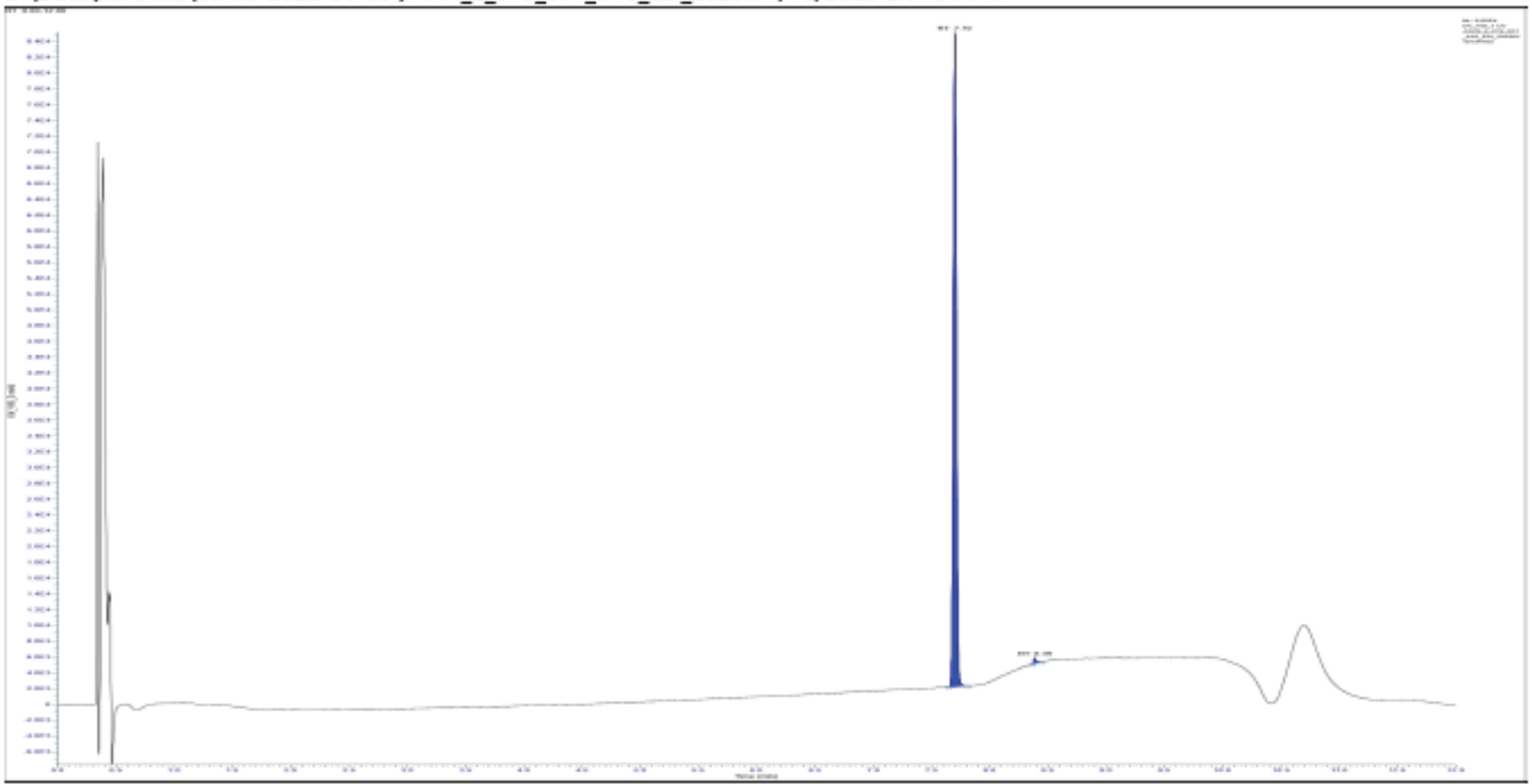

JHKN_2_018_001_fr15_ESI_20230110134703 #1731 RT: 7.70 AV: 1 NL: 1.69E9
T: FTMS + c ESI Full ms [100.0000-1000.0000]

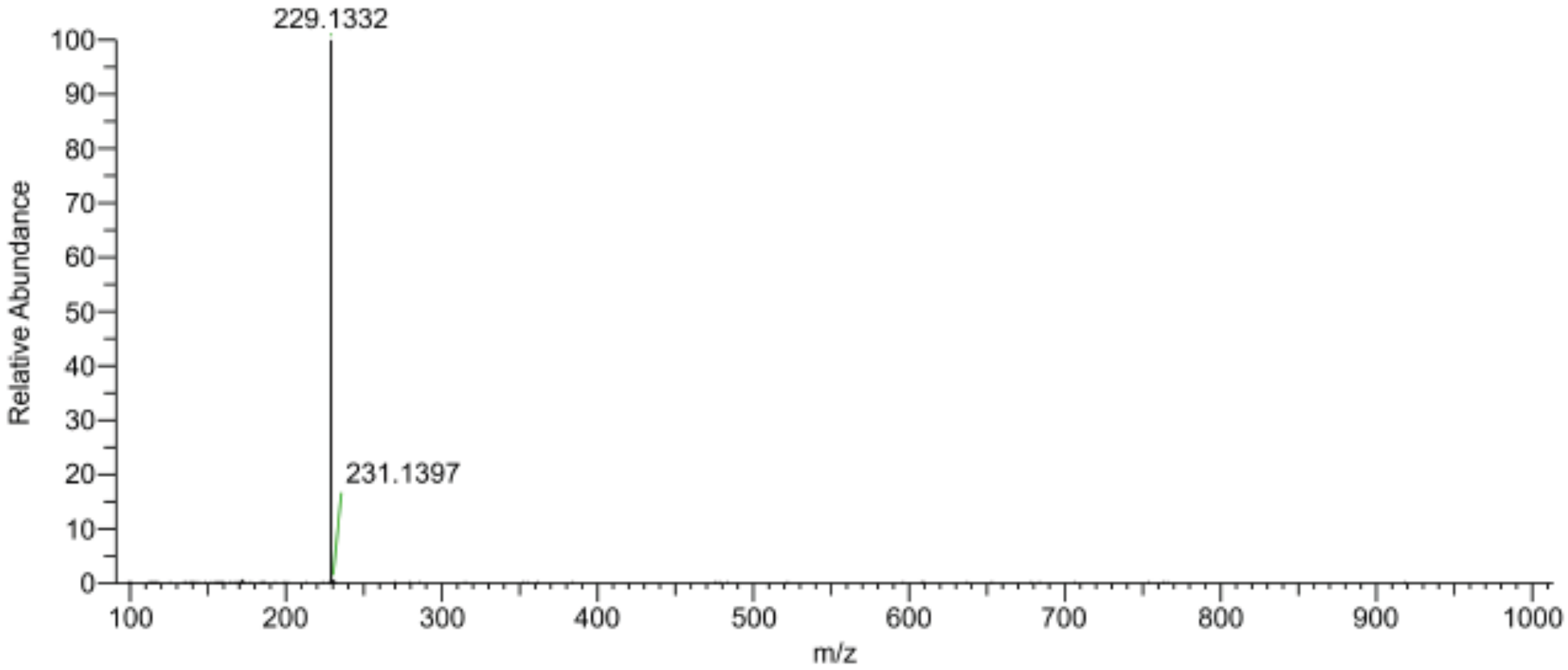

Index	% Area	RT (Mi...	RT (Sec)	Start RT	End RT	Base P...	Peak...	Peak...	Baseli...	Signal...	FileNa...	Scan F...	TraceId	% Hei...	Integr...	Name
1	99.27	7.7017	462....	7.64...	7.80...	0	1511...	8294...	0.16...	7.33E1	D:\D...	No F...	Trace1	99.1	Man...	
2	0.73	8.3867	503....	8.35...	8.43...	0	1104...	752....	0.08...	6.77...	D:\D...	No F...	Trace1	0.9	Man...	

PC\Results\JHKN_2_018_001_fr23(HPLC)_2023-08-14T13-37-47.a2r

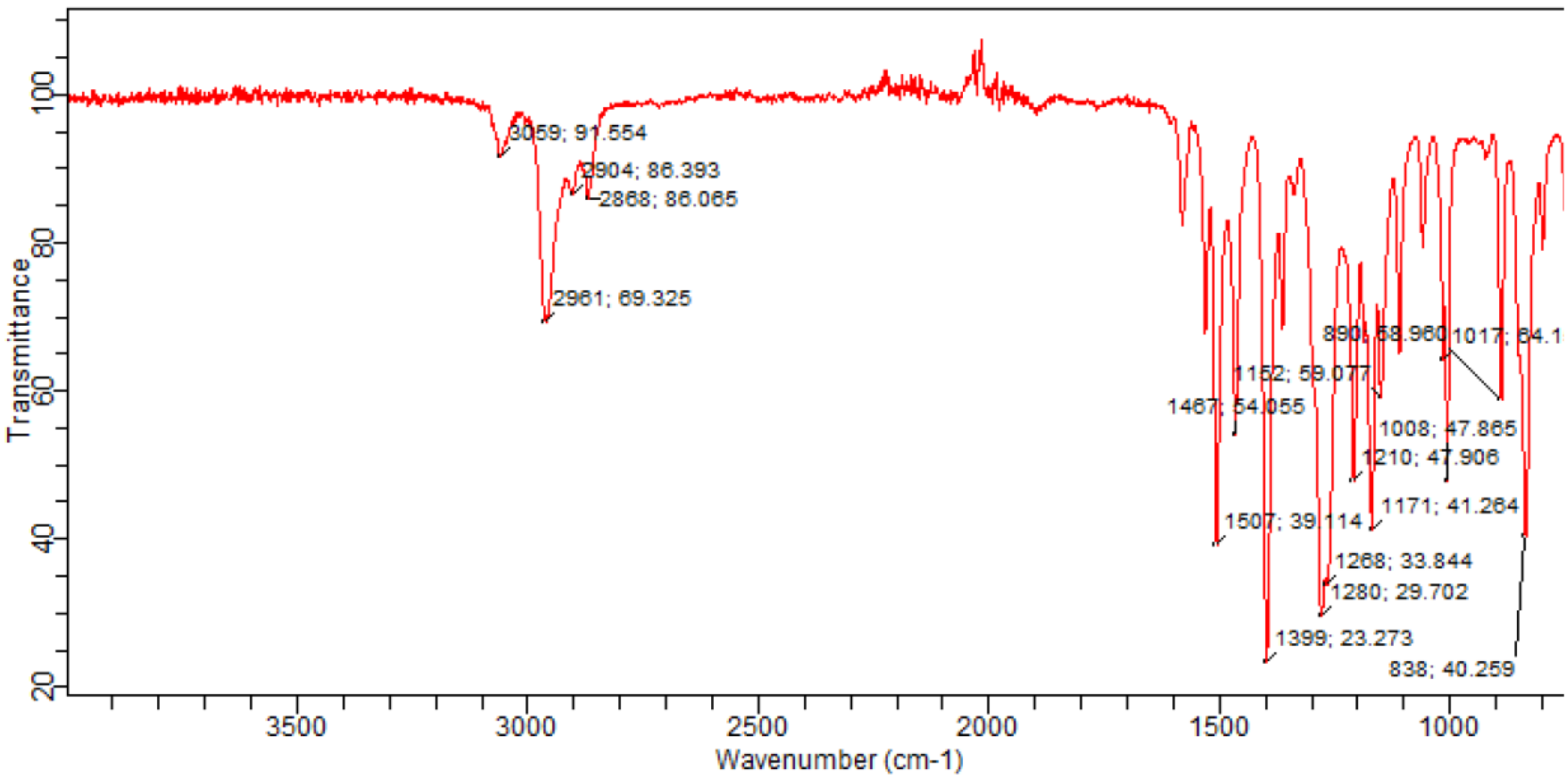

2d

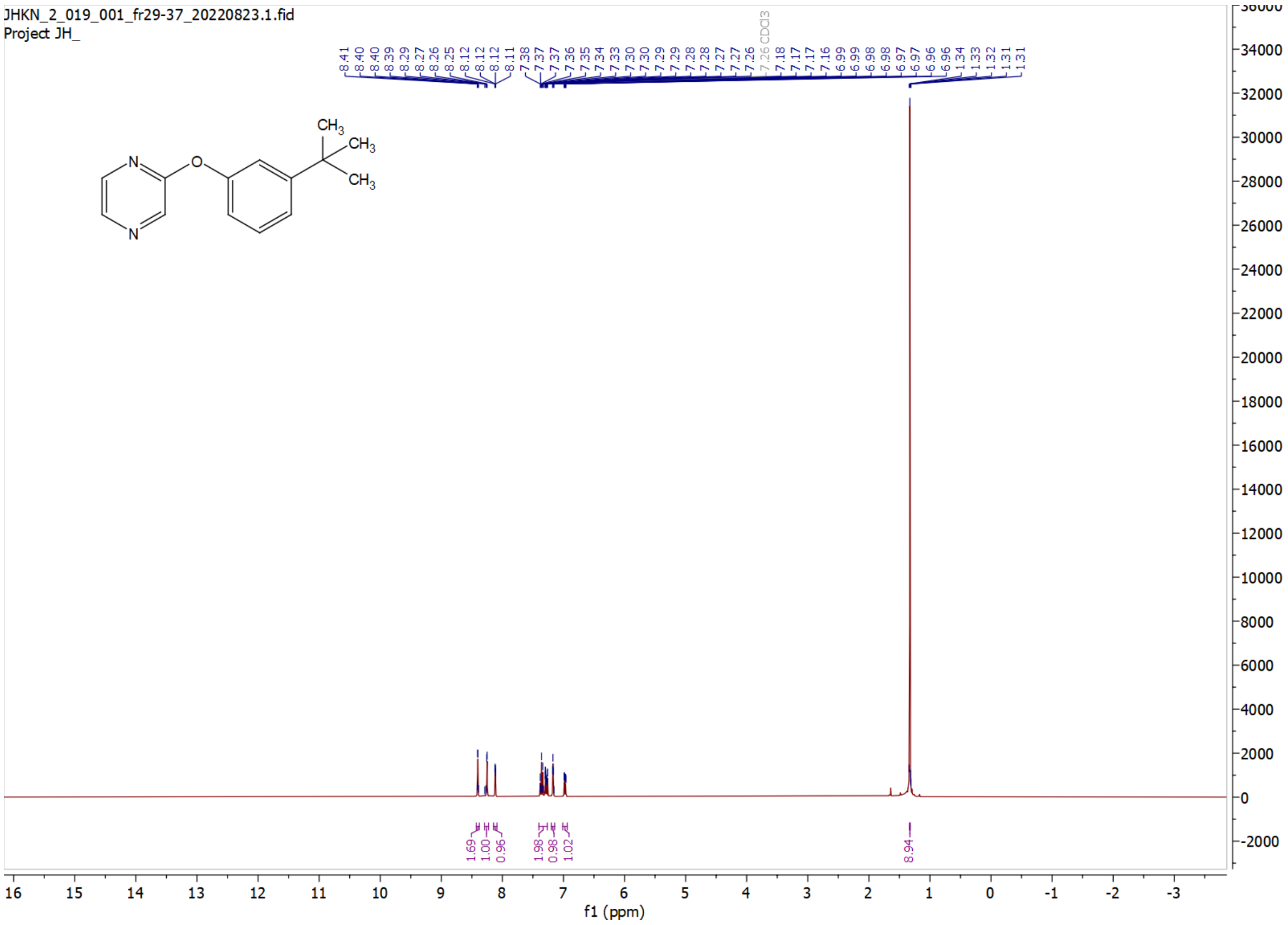
JHKN_2_019_001_fr29-37_20220823.1.fid
Project JH_
f1 (ppm)

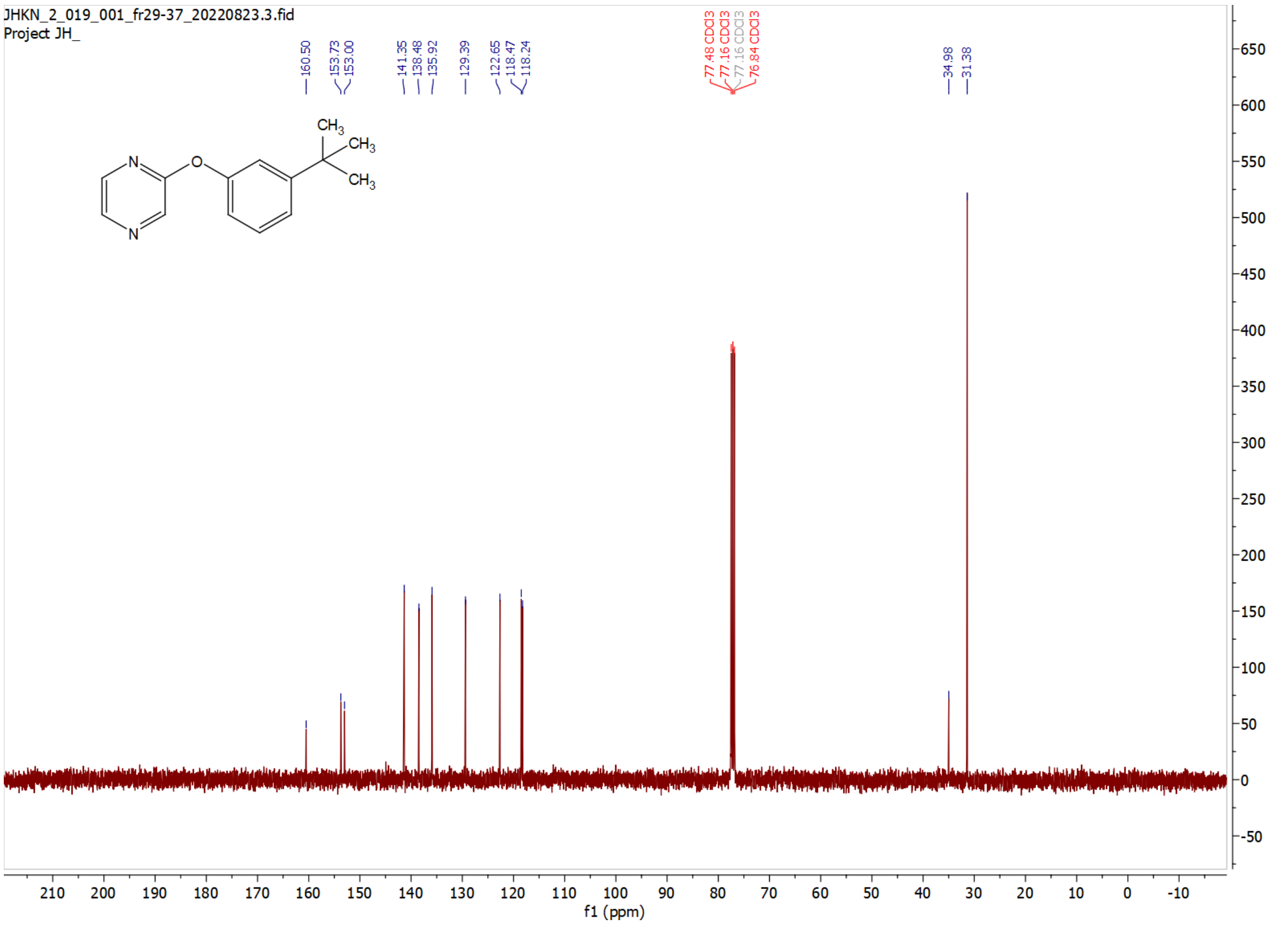
JHKN_2_019_001_fr29-37_20220823.3.fid
Project JH_
160.50
153.73
153.00
141.35
138.48
135.92
129.39
122.65
118.47
118.24
77.48 CDCl3
77.16 CDCl3
76.84 CDCl3
34.98
31.38
f1 (ppm)

D:\Data\Karoline\LCMS-2022-06-03\JHKN_2_019_001_fr28-37_ESI.raw 1/10/2023 12:02:13 PM

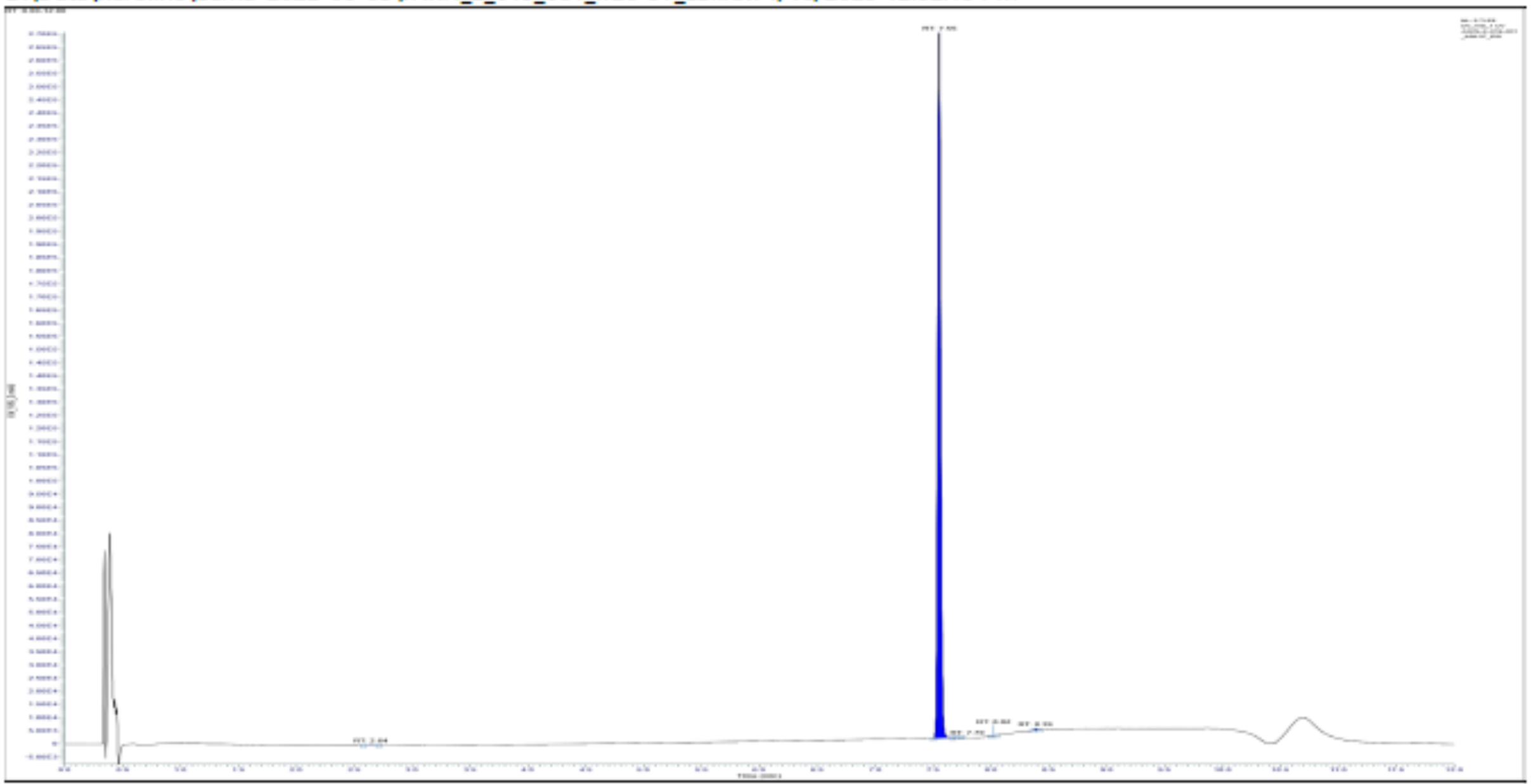

JHKN_2_019_001_fr28-37_ESI #1696 RT: 7.55 AV: 1 NL: 1.12E9
T: FTMS + c ESI Full ms [100.0000-1000.0000]

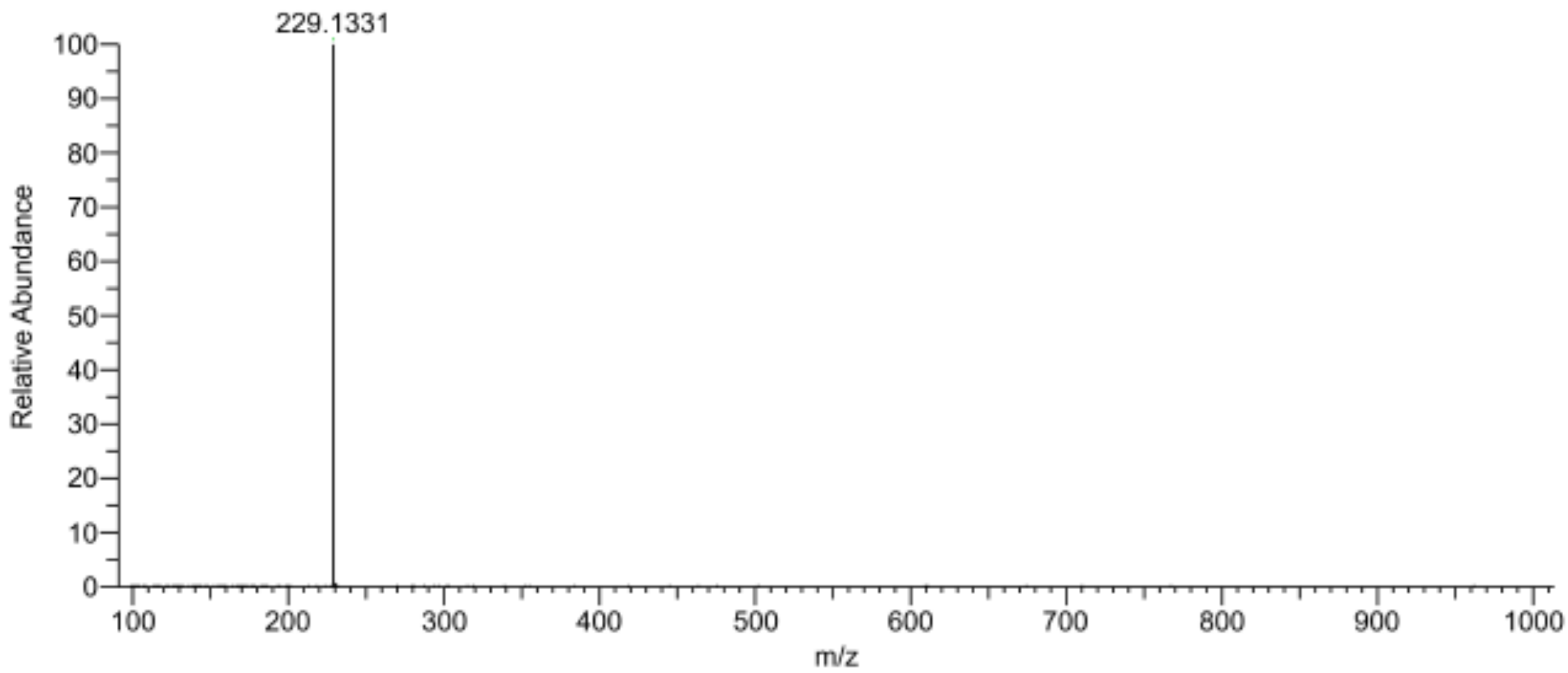

Index	% Area	RT (Mi...	RT (Sec)	Start RT	End RT	Base P...	Peak...	Peak...	Baseli...	Signal...	FileNa...	Scan F...	TraceId	% Hei...	Integr...	Name
1	0.12	2.64...	158....	2.57...	2.70...	0	606....	129....	0.13...	1.14...	D:\D...	No F...	Trace1	0.05	Man...	
2	99.56	7.55...	453....	7.49...	7.66...	0	4925...	2685...	0.16...	2.38E2	D:\D...	No F...	Trace1	99.55	Man...	
3	0.08	7.70...	462....	7.66...	7.74...	0	417....	240....	0.08...	2.14...	D:\D...	No F...	Trace1	0.09	Man...	
4	0.03	8.02...	481....	7.99...	8.04...	0	171....	137....	0.05...	1.22...	D:\D...	No F...	Trace1	0.05	Man...	
5	0.19	8.38...	503....	8.35...	8.42...	0	962....	716....	0.06...	6.39...	D:\D...	No F...	Trace1	0.27	Man...	

2e

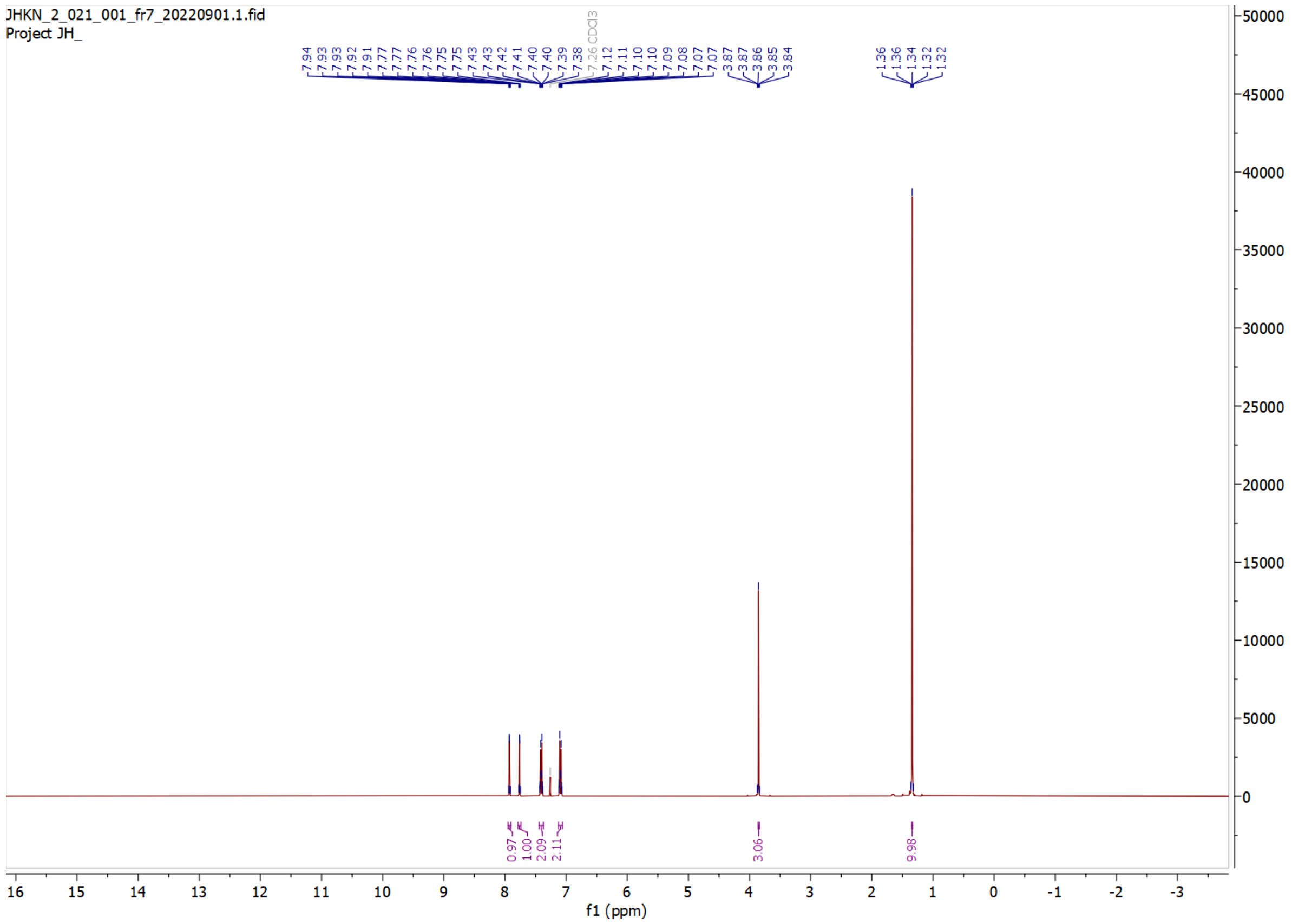
JHKN_2_021_001_fr7_20220901.1.fid
Project JH_
7.94 7.93 7.93 7.92 7.91 7.77 7.77 7.76 7.76 7.75 7.75 7.43 7.43 7.42 7.41 7.40 7.40 7.39 7.38 7.26 CDCl3 7.12 7.11 7.10 7.10 7.09 7.08 7.07 7.07 3.87 3.87 3.86 3.85 3.84
1.36 1.36 1.34 1.32 1.32
0.97 1.00 2.09 2.11 3.06 9.98
f1 (ppm)

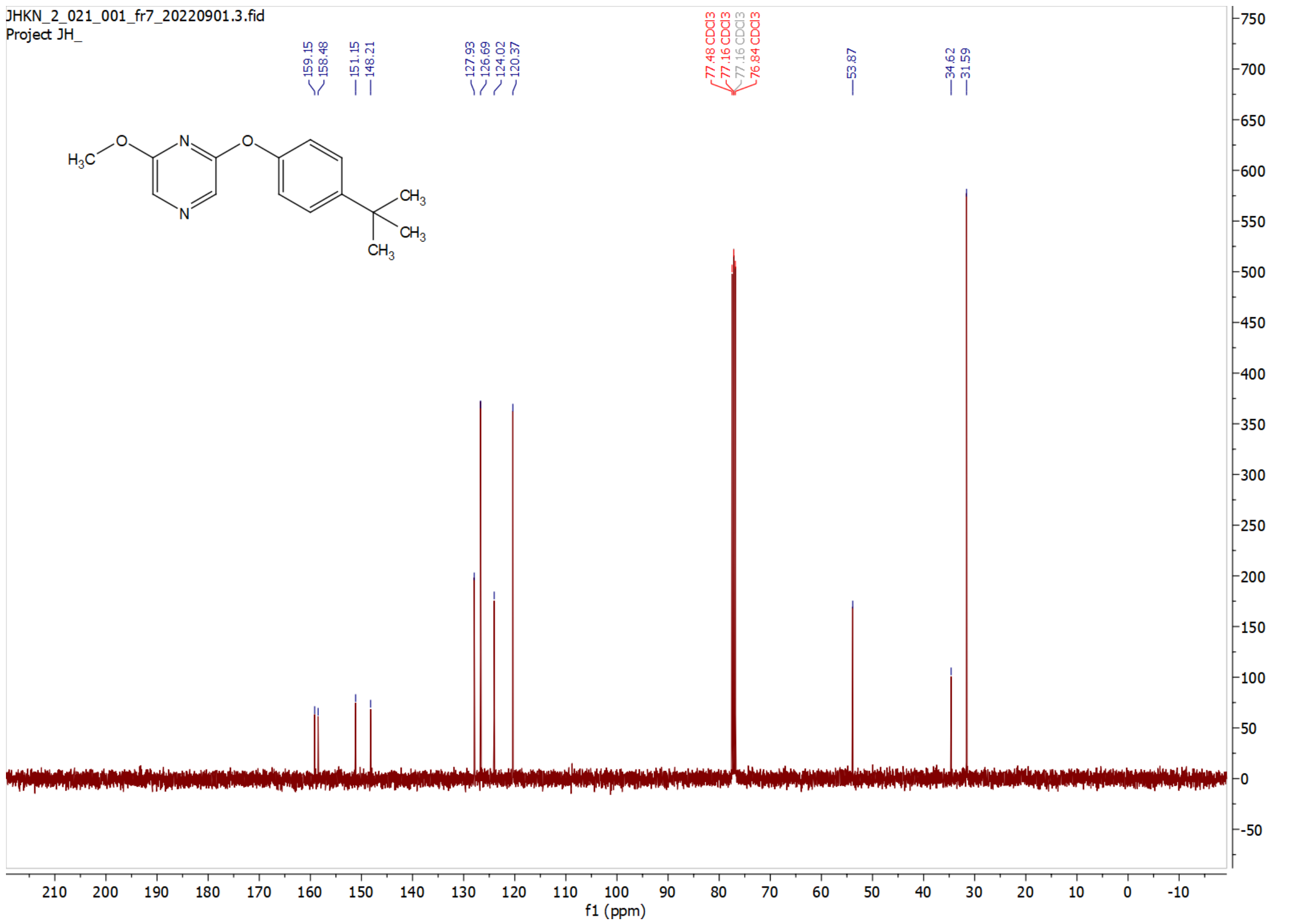
JHKN_2_021_001_fr7_20220901.3.fid
Project JH_
159.15 158.48 151.15 148.21 127.93 126.69 124.02 120.37
77.48 CDCl3 77.16 CDCl3 77.16 CDCl3 76.84 CDCl3
53.87 34.62 31.59
H3C O N O N CH3 CH3 CH3
f1 (ppm)

D:\Data\Karoline\LCMS-2022-06-03\JHKN_2_021_001_fr7-9_ESI.raw 1/9/2023 2:13:02 PM

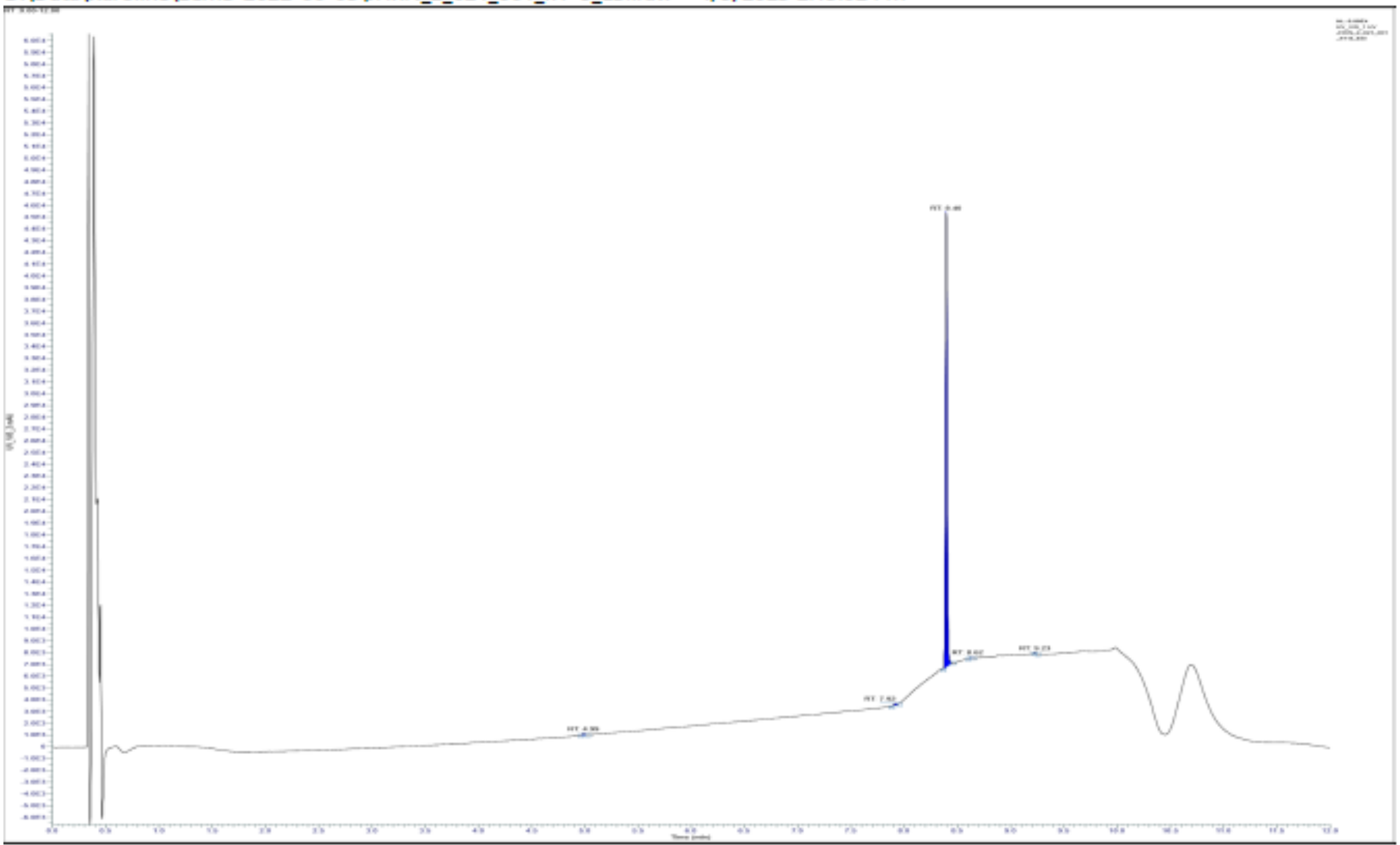

JHKN_2_021_001_fr7-9_ESI #1889 RT: 8.40 AV: 1 NL: 7.52E8
T: FTMS + c ESI Full ms [100.0000-1000.0000]

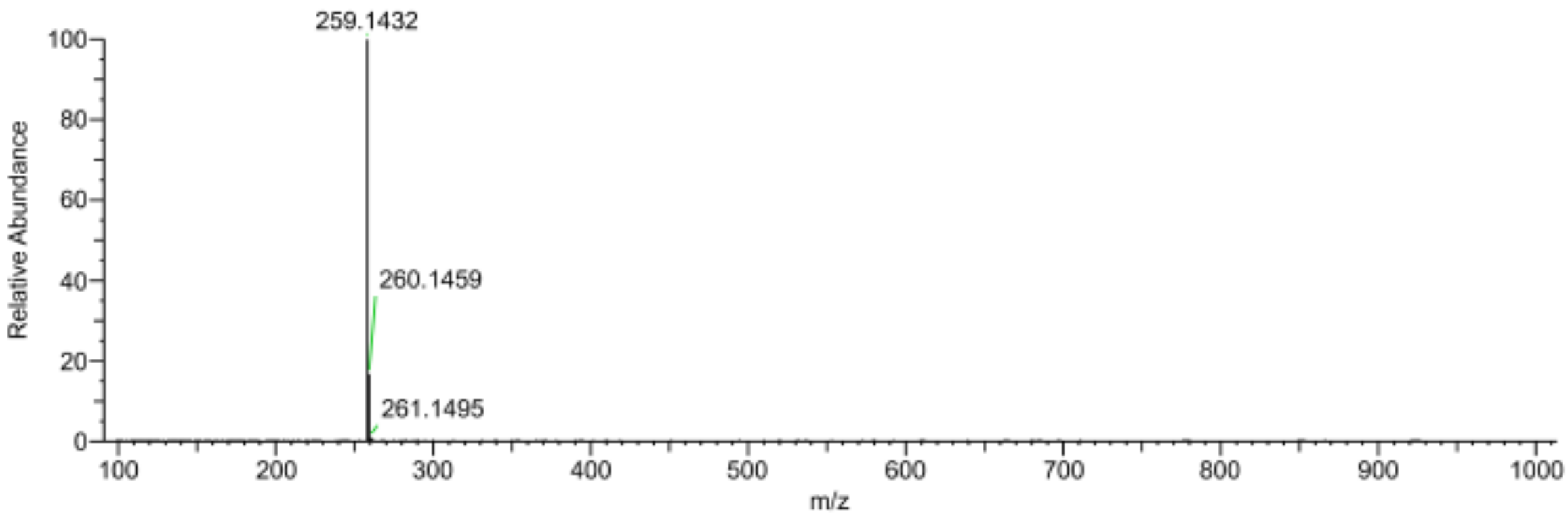

Index	% Area	RT (Mi...	RT (Sec)	Start RT	End RT	Base P...	Peak...	Peak...	Baseli...	Signal...	FileNa...	Scan F...	TraceId	% Hei...	Integr...	Name
1	0.66	4.9867	299....	4.95...	5.02...	0	288....	138....	0.06...	1.07...	D:\D...	No F...	Trace1	0.36	Man...	
2	0.92	7.91...	475....	7.88...	7.95...	0	405....	158....	0.07...	1.22...	D:\D...	No F...	Trace1	0.41	Man...	
3	97.99	8.3967	503....	8.36...	8.4517	0	4307...	3859...	0.08...	2.98E1	D:\D...	No F...	Trace1	98.79	Man...	
4	0.22	8.61...	517....	8.59...	8.64...	0	98.6...	79.6...	0.04...	6.16...	D:\D...	No F...	Trace1	0.2	Man...	
5	0.21	9.23...	553....	9.21...	9.25...	0	92.0...	96.2...	0.03...	7.43...	D:\D...	No F...	Trace1	0.25	Man...	

2f

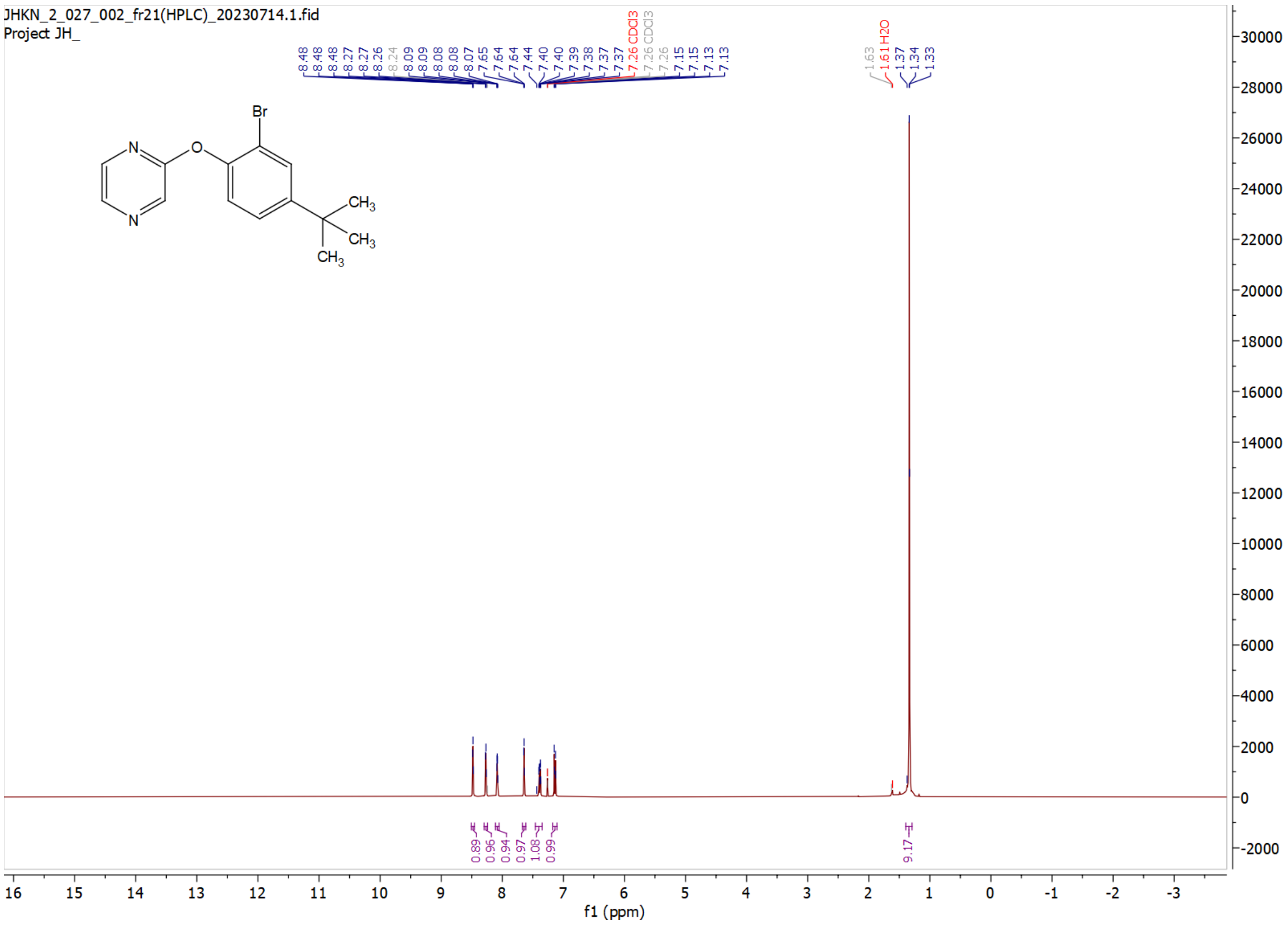
JHKN_2_027_002_fr21(HPLC)_20230714.1.fid
Project JH_
Br
O
N
N
CH3
CH3
CH3
f1 (ppm)

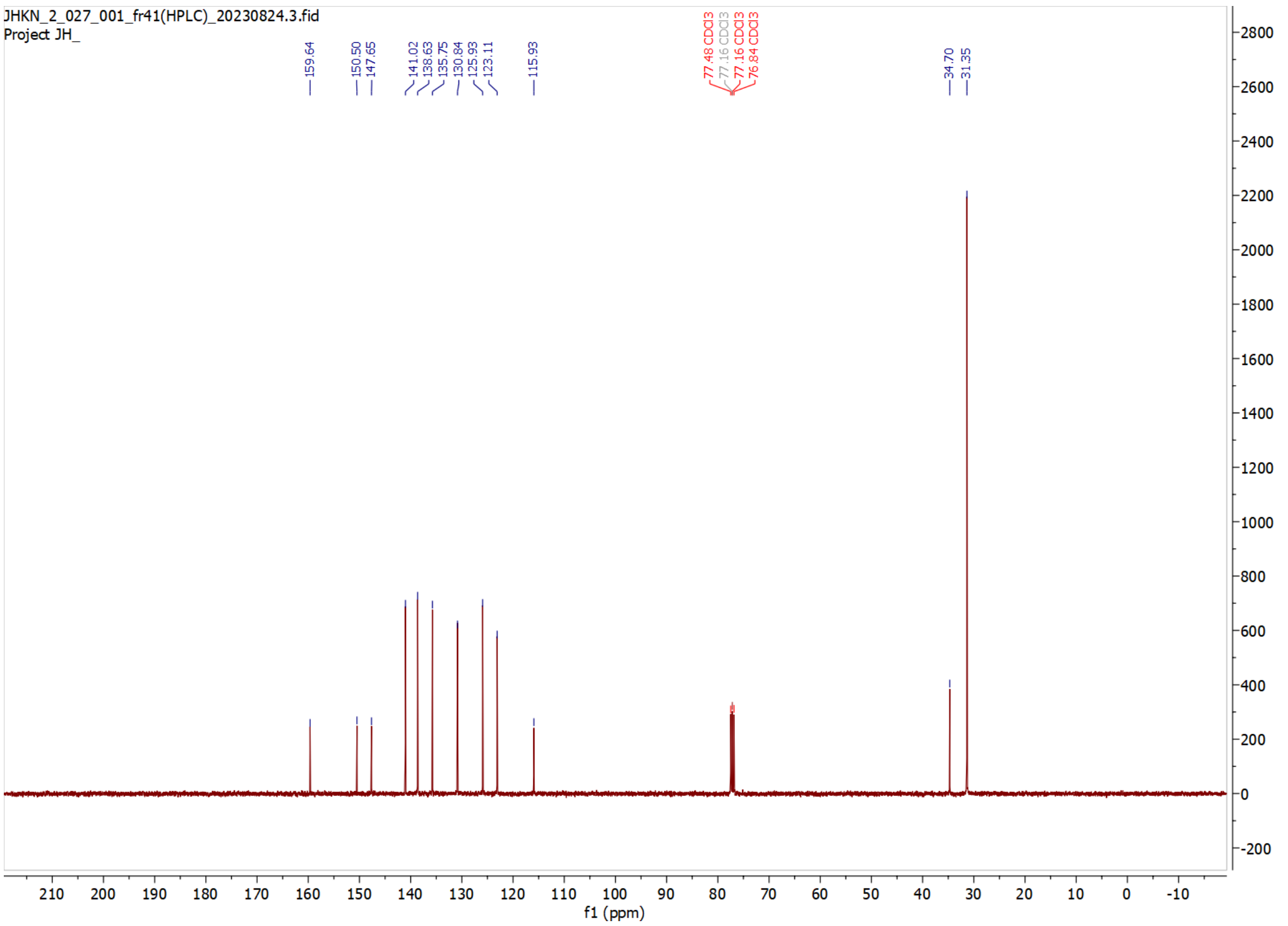
JHKN_2_027_001_fr41(HPLC)_20230824.3.fid
Project JH_
f1 (ppm)

D:\Data\Karoline\LCMS-2022-06-03\JHKN_2_027_002_fr21_ESI.raw 7/14/2023 12:10:24 PM

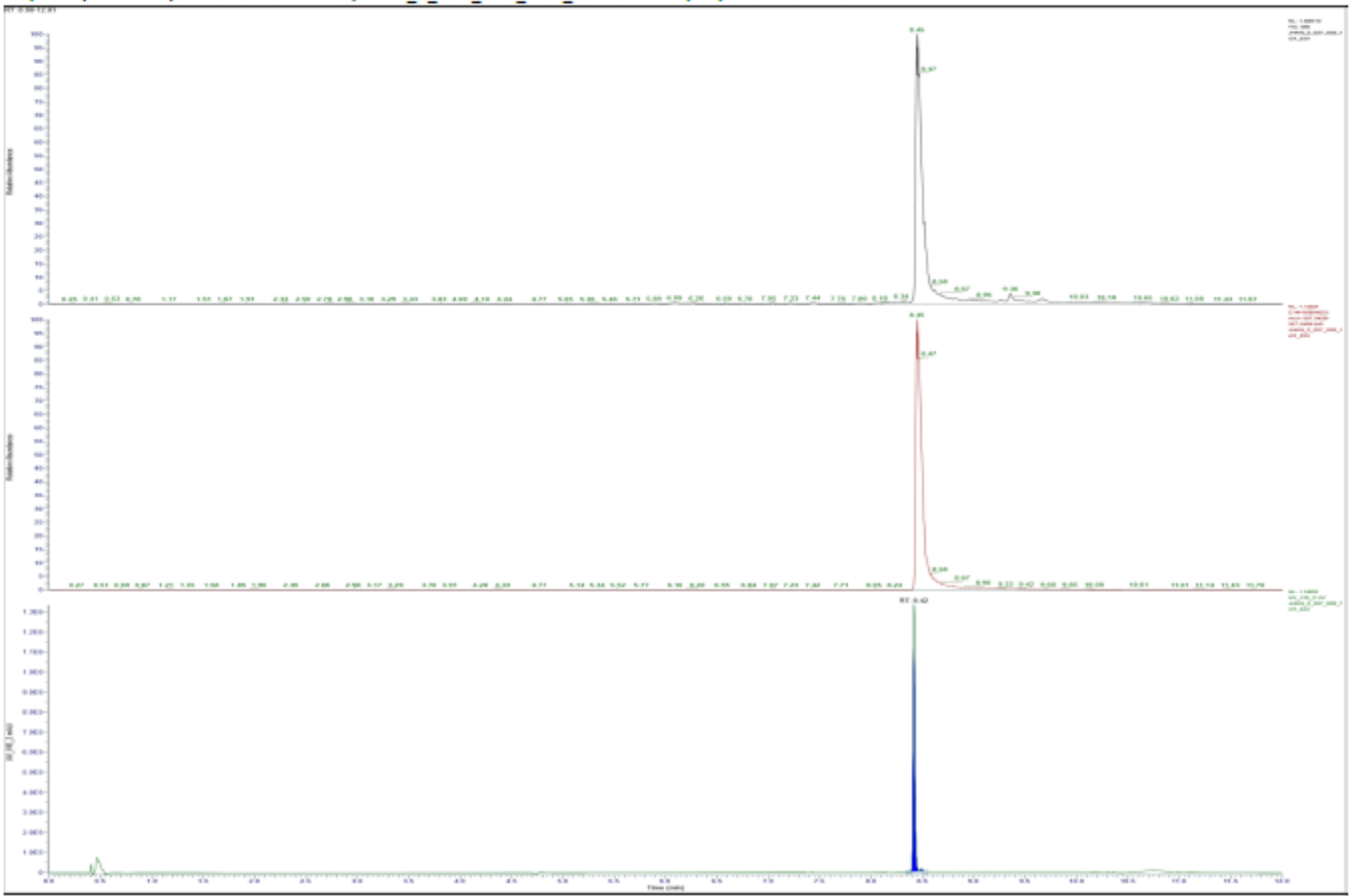

JHKN_2_027_002_fr21_ESI #1901 RT: 8.46 AV: 1 NL: 7.40E9
T: FTMS + c ESI Full ms [100.0000-1000.0000]

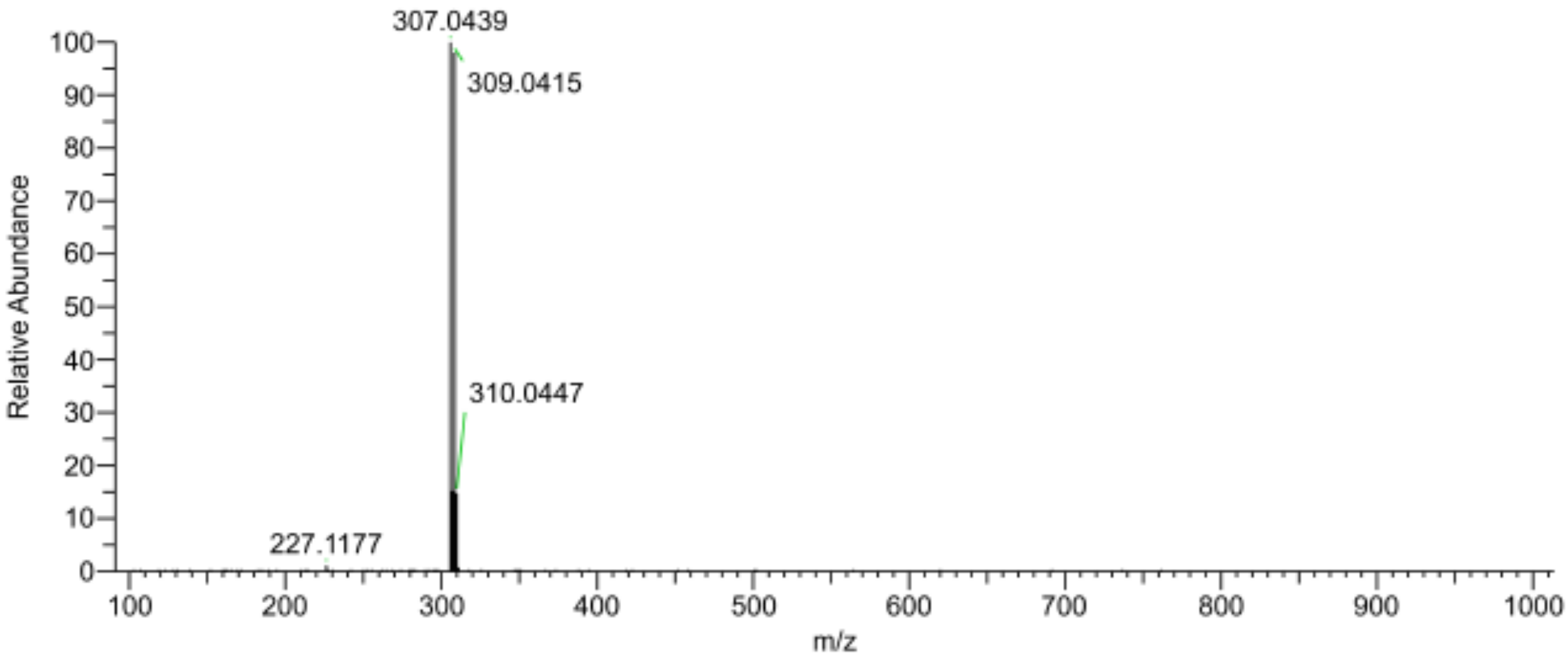

Index	% Area	RT (Mi...	RT (Sec)	Start RT	End RT	Base P...	Peak...	Peak...	Baseli...	Signal...	FileNa...	Scan F...	TraceId	% Hei...	Integr...	Name
1	100	8.42...	505....	8.36...	8.52...	0	1673...	1329...	0.16...	9.96E2	D:\D...	No F...	Trace3	100	Man...	

File Location: C:\Program Files (x86)\Agilent\MicroLab PC\Results\JHKN_2_027_002_fr21_2023-07-14T 11-38-43.a2r

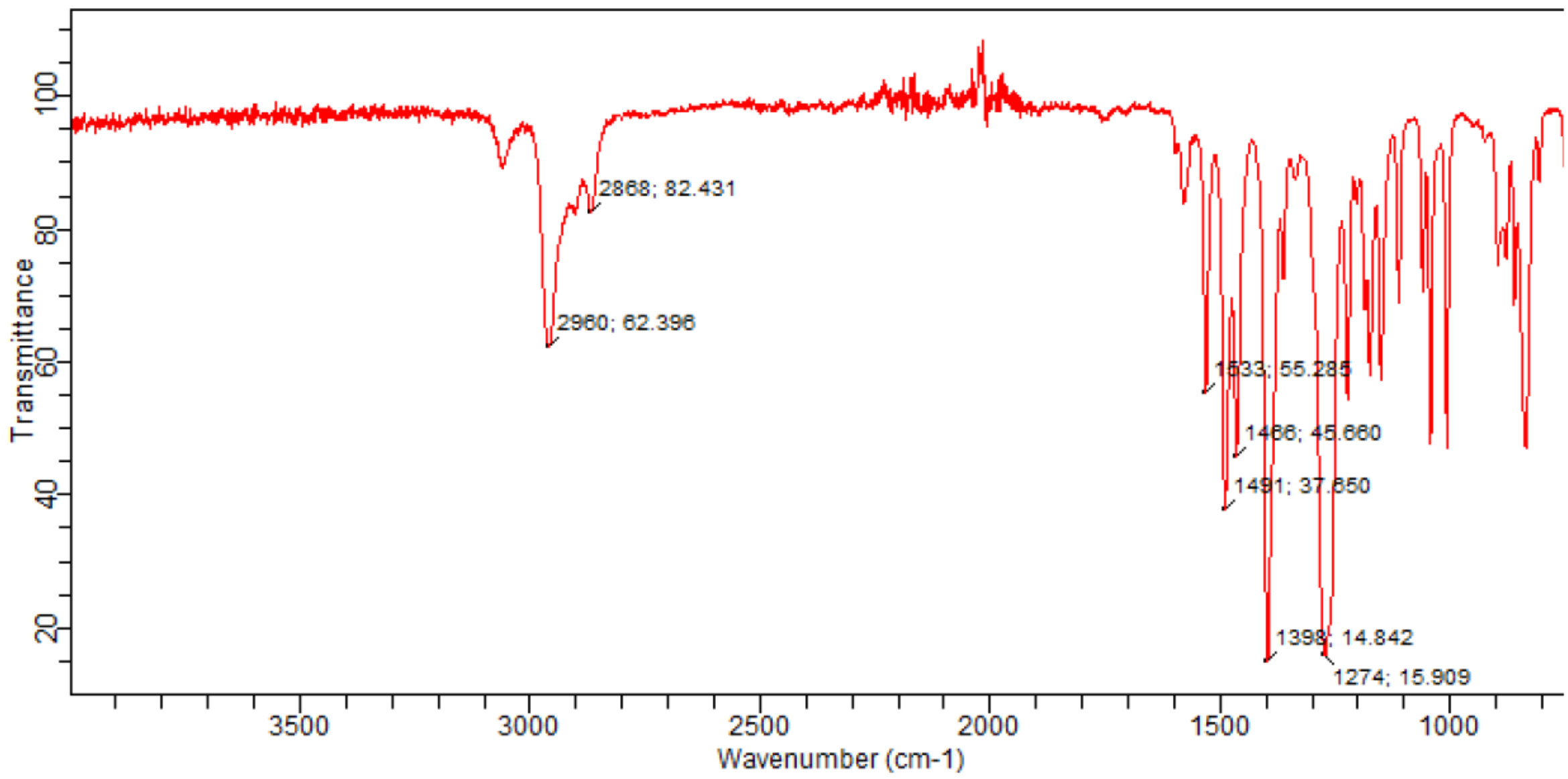

1b

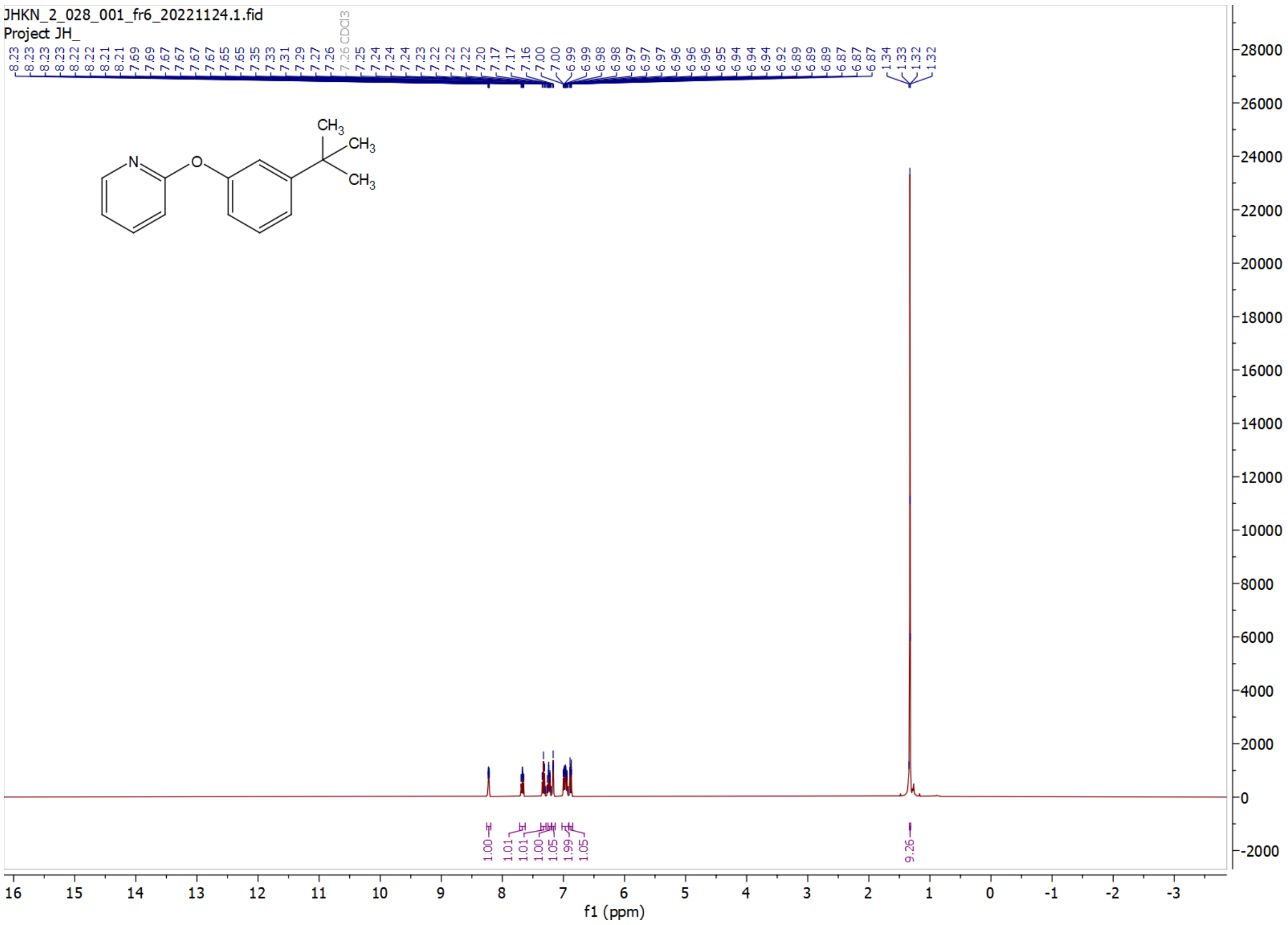

JHKN_2_028_001_fr6_20221124.1.fid
Project JH_
8.23 8.23 8.23 8.23 8.22 8.22 8.21 8.21 7.69 7.69 7.67 7.67 7.67 7.65 7.65 7.35 7.33 7.31 7.29 7.27 7.26 CDCl3 7.25 7.24 7.24 7.24 7.23 7.22 7.22 7.22 7.20 7.17 7.17 7.16 7.00 7.00 6.99 6.99 6.98 6.97 6.97 6.97 6.96 6.96 6.96 6.95 6.94 6.94 6.94 6.92 6.89 6.89 6.89 6.87 6.87 6.87 1.34 1.33 1.32 1.32
N
O
CH3
CH3
CH3
1.00 1.01 1.01 1.00 1.05 1.99 1.05 9.26
f1 (ppm)

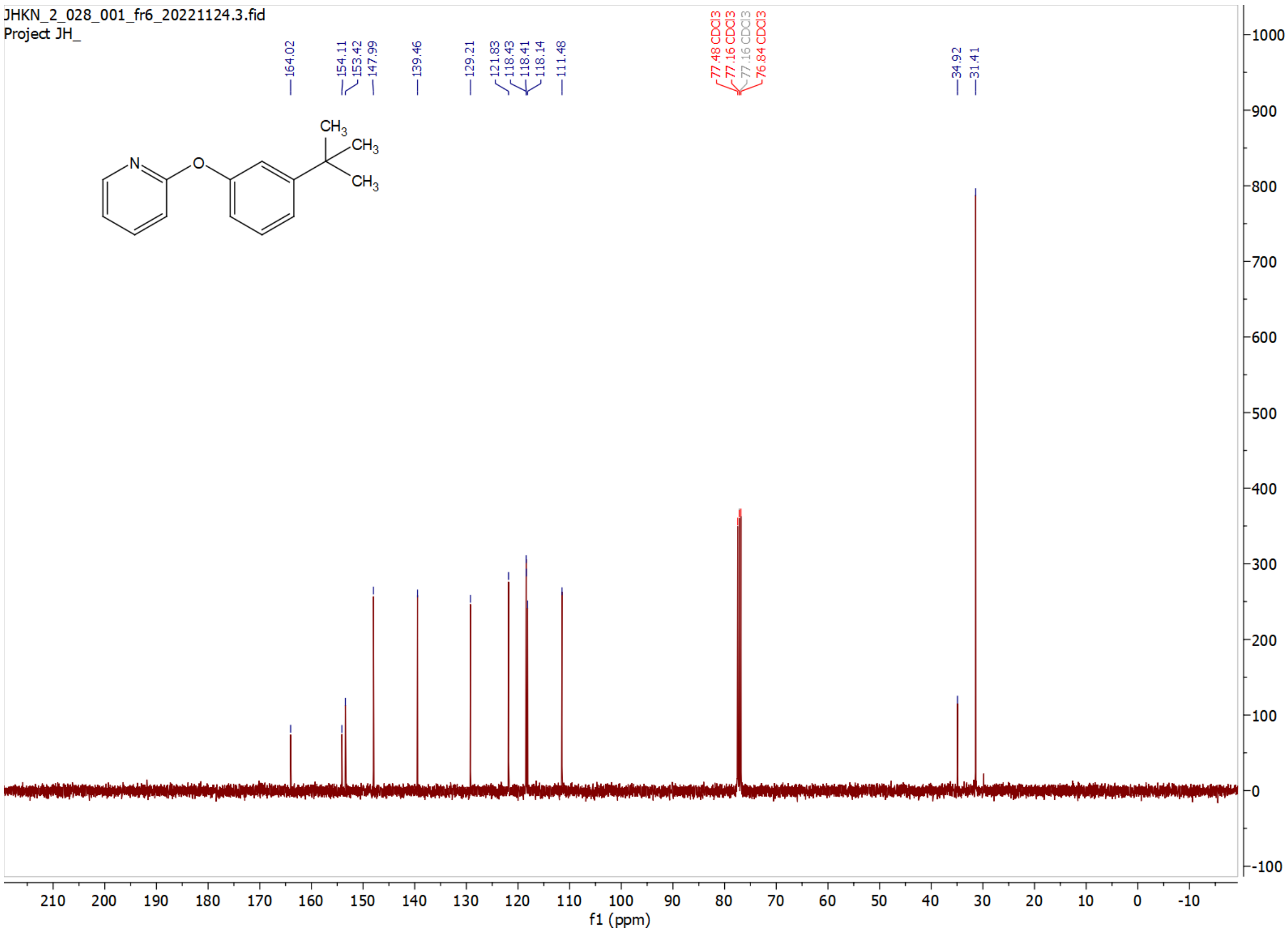

JHKN_2_028_001_fr6_20221124.3.fid
Project JH_
164.02 154.11 153.42 147.99 139.46 129.21 121.83 118.43 118.41 118.14 111.48
77.48 CDCl3 77.16 CDCl3 77.16 CDCl3 76.84 CDCl3
34.92 31.41
N
O
CH3
CH3
CH3
f1 (ppm)

D:\Data\Karoline\LCMS-2022-06-03\JHKN_2_028_001_fr6_ESI.raw 11/25/2022 10:41:51 AM

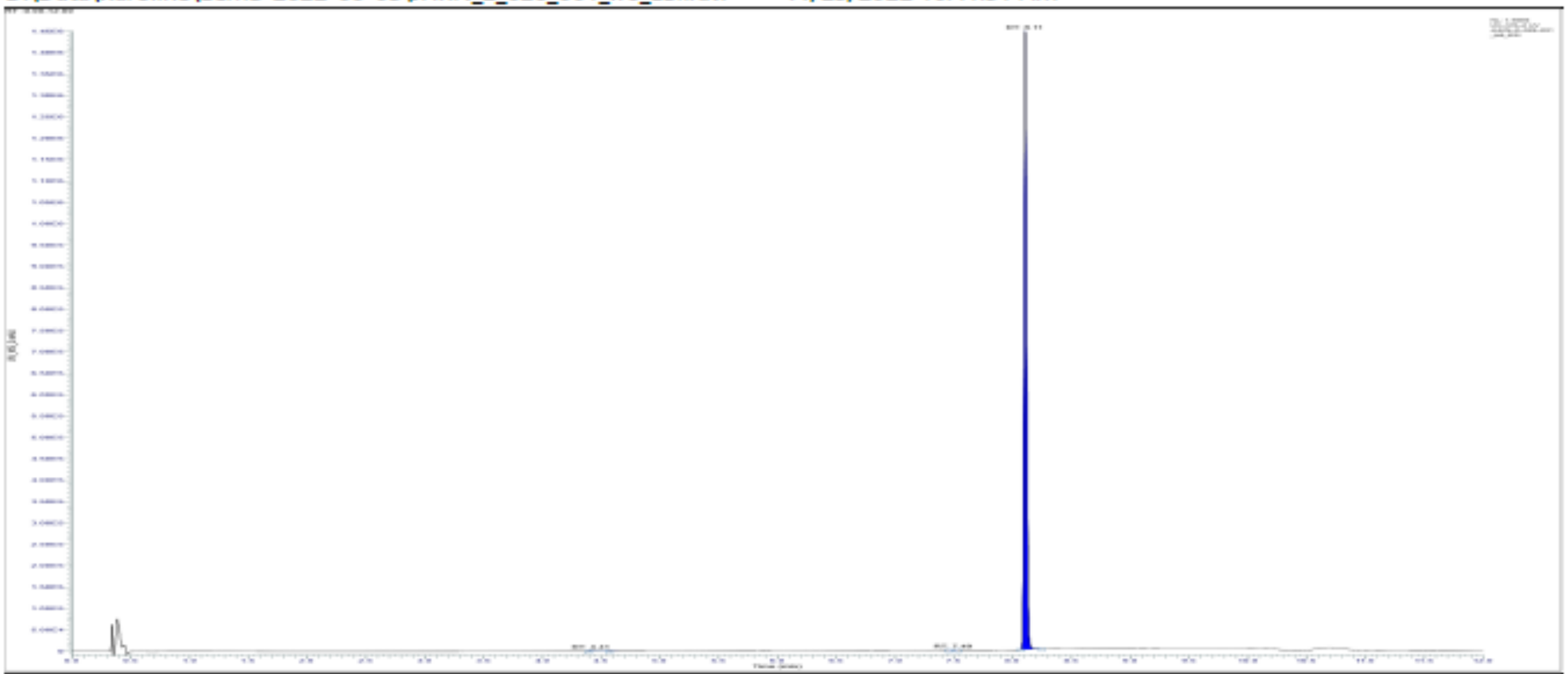

JHKN_2_028_001_fr6_ESI #1824 RT: 8.12 AV: 1 NL: 1.22E10
T: FTMS + c ESI Full ms [100.0000-1000.0000]

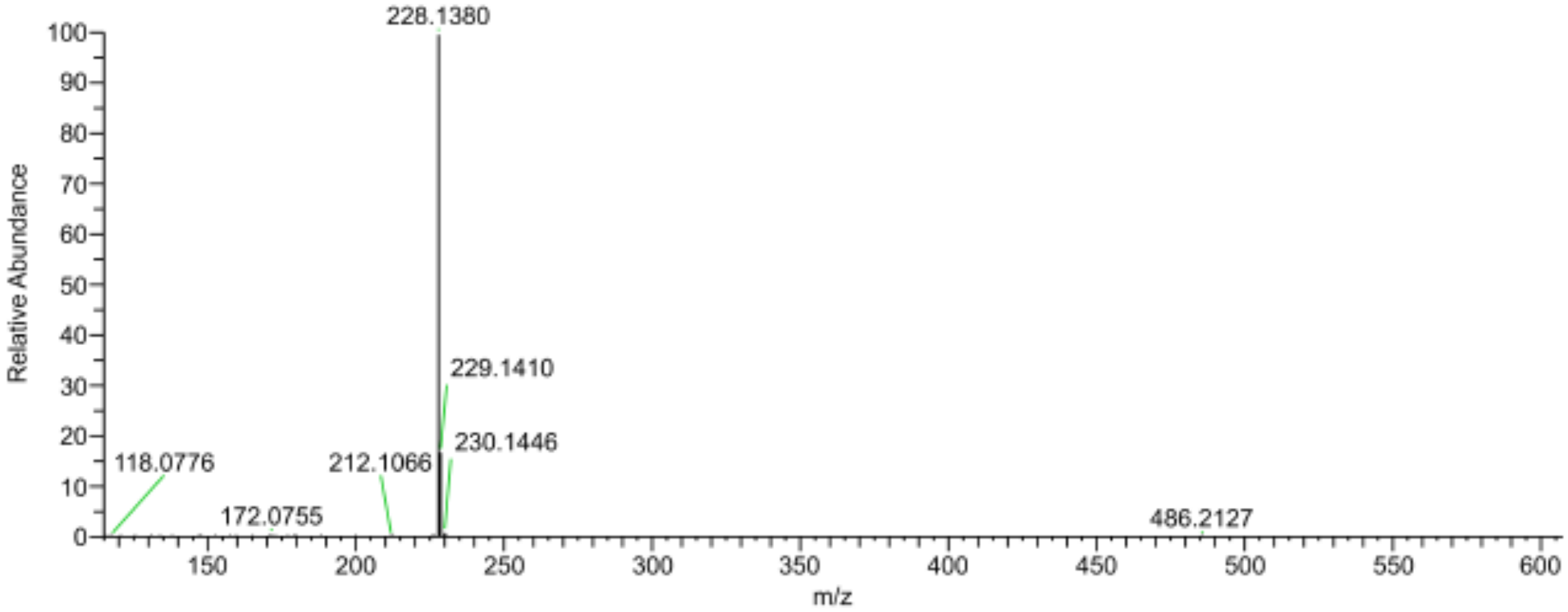

Index	% Area	RT (Mi...	RT (Sec)	Start RT	End RT	Base P...	Peak...	Peak...	Baseli...	Signal...	FileNa...	Scan F...	TraceId	% Hei...	Integr...	Name
1	0.16	4.41...	264....	4.38...	4.56...	0	3336...	588....	0.18...	4.87...	D:\D...	No F...	Trace1	0.04	Man...	
2	0.03	7.49...	449....	7.45...	7.53...	0	577....	284....	0.07...	2.37...	D:\D...	No F...	Trace1	0.02	Man...	
3	99.82	8.10...	486....	8.06...	8.24...	0	2137...	1446...	0.18...	1.21E3	D:\D...	No F...	Trace1	99.94	Man...	

1c

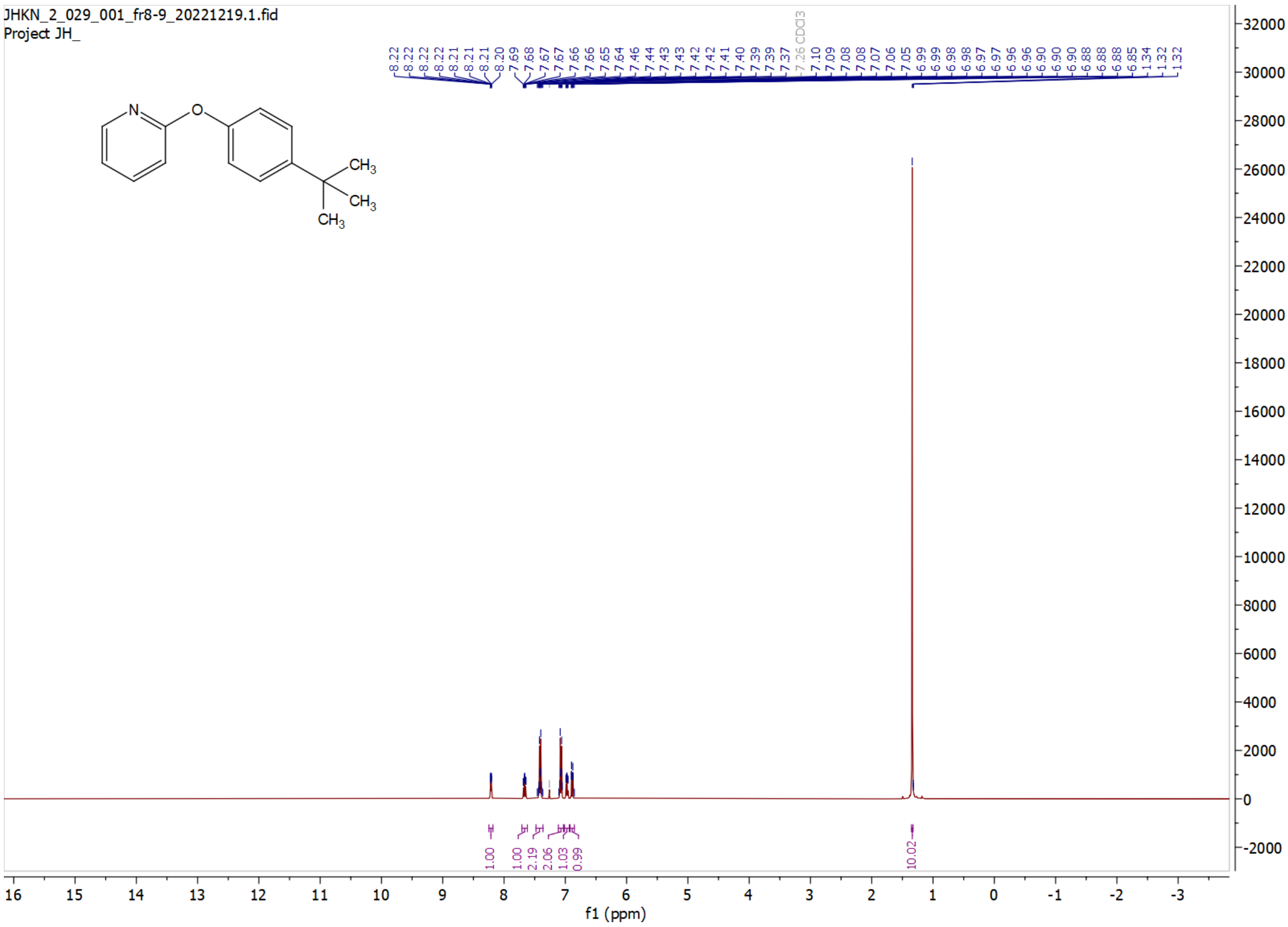
JHKN_2_029_001_fr8-9_20221219.1.fid
Project JH_
f1 (ppm)

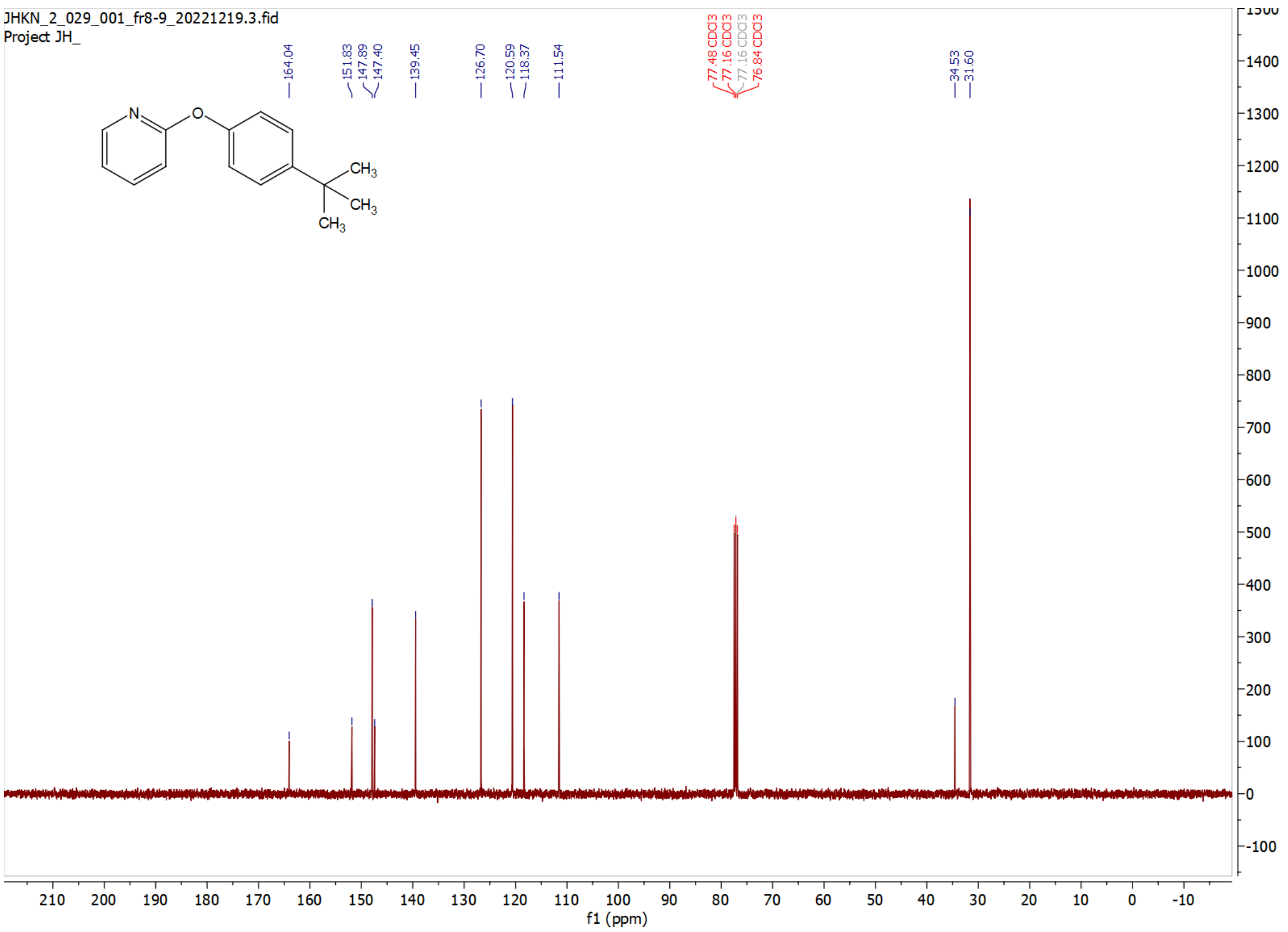
JHKN_2_029_001_fr8-9_20221219.3.fid
Project JH_
164.04
151.83
147.89
147.40
139.45
126.70
120.59
118.37
111.54
77.48 CDCl3
77.16 CDCl3
77.16 CDCl3
76.84 CDCl3
34.53
31.60
f1 (ppm)

D:\Data\Karoline\LCMS-2022-06-03\JHKN_2_029_001_fr8-9_ESI.raw   11/23/2022 11:29:58 AM

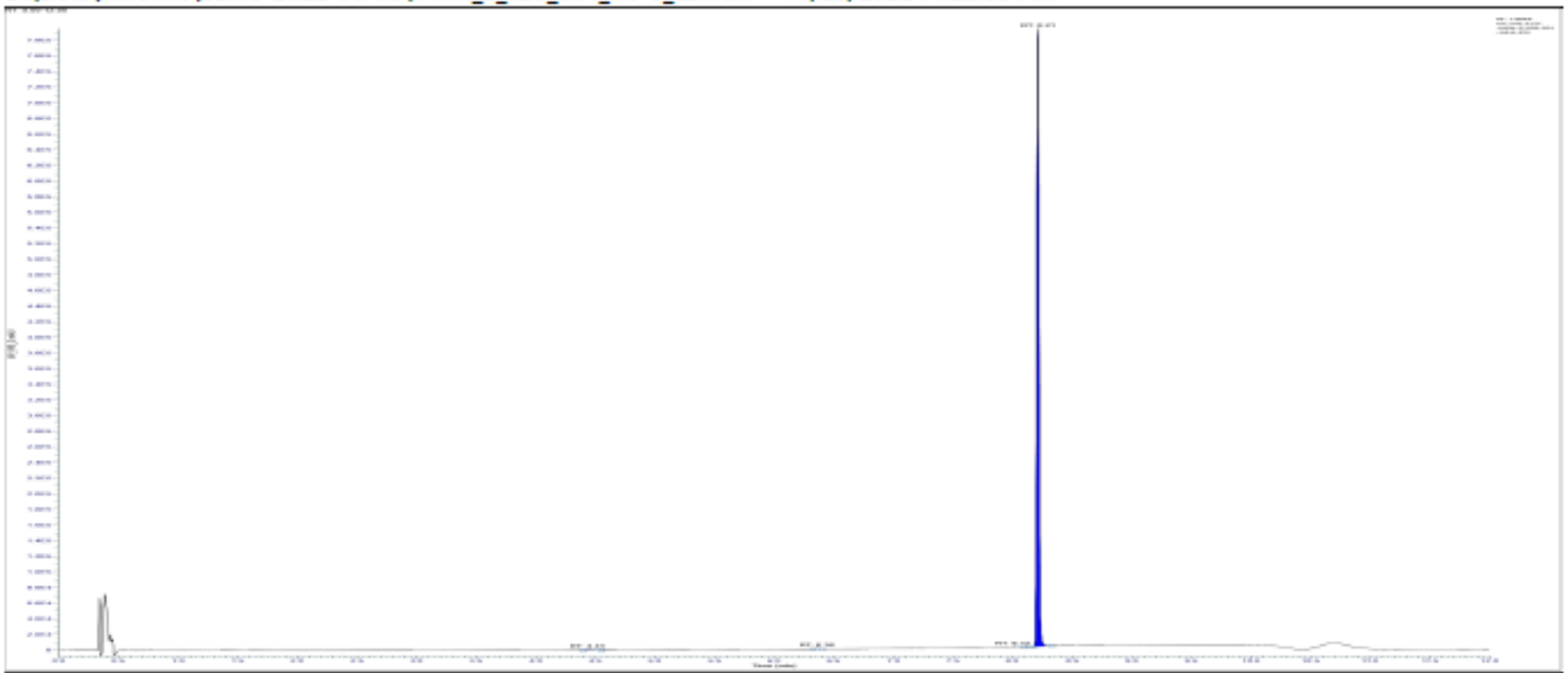

JHKN_2_029_001_fr8-9_ESI #1847 RT: 8.22 AV: 1 NL: 1.17E10
T: FTMS + c ESI Full ms [100.0000-1000.0000]

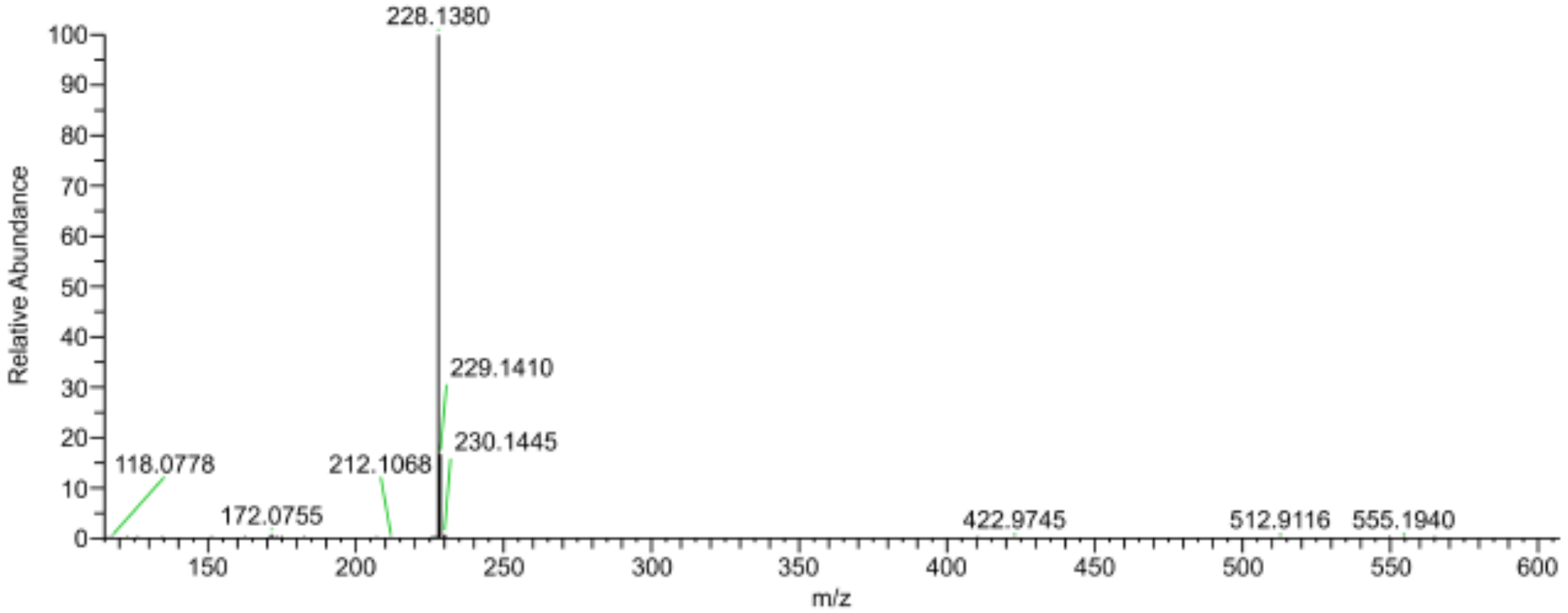

Index	% Area	RT (Mi...	RT (Sec)	Start RT	End RT	Base P...	Peak...	Peak...	Baseli...	Signal...	FileNa...	Scan F...	TraceId	% Hei...	Integr...	Name
1	0.27	4.43...	265....	4.39...	4.55...	0	2715...	518....	0.15...	4.30...	D:\D...	No F...	Trace1	0.07	Man...	
2	0.12	6.3584	381....	6.31...	6.39...	0	1216...	574....	0.08...	4.78...	D:\D...	No F...	Trace1	0.07	Man...	
3	0.05	8.12...	487....	8.09...	8.14...	0	470....	342....	0.04...	2.85...	D:\D...	No F...	Trace1	0.04	Man...	
4	99.57	8.21...	492....	8.17...	8.32...	0	1015...	7900...	0.15...	6.59E2	D:\D...	No F...	Trace1	99.82	Man...	

1d

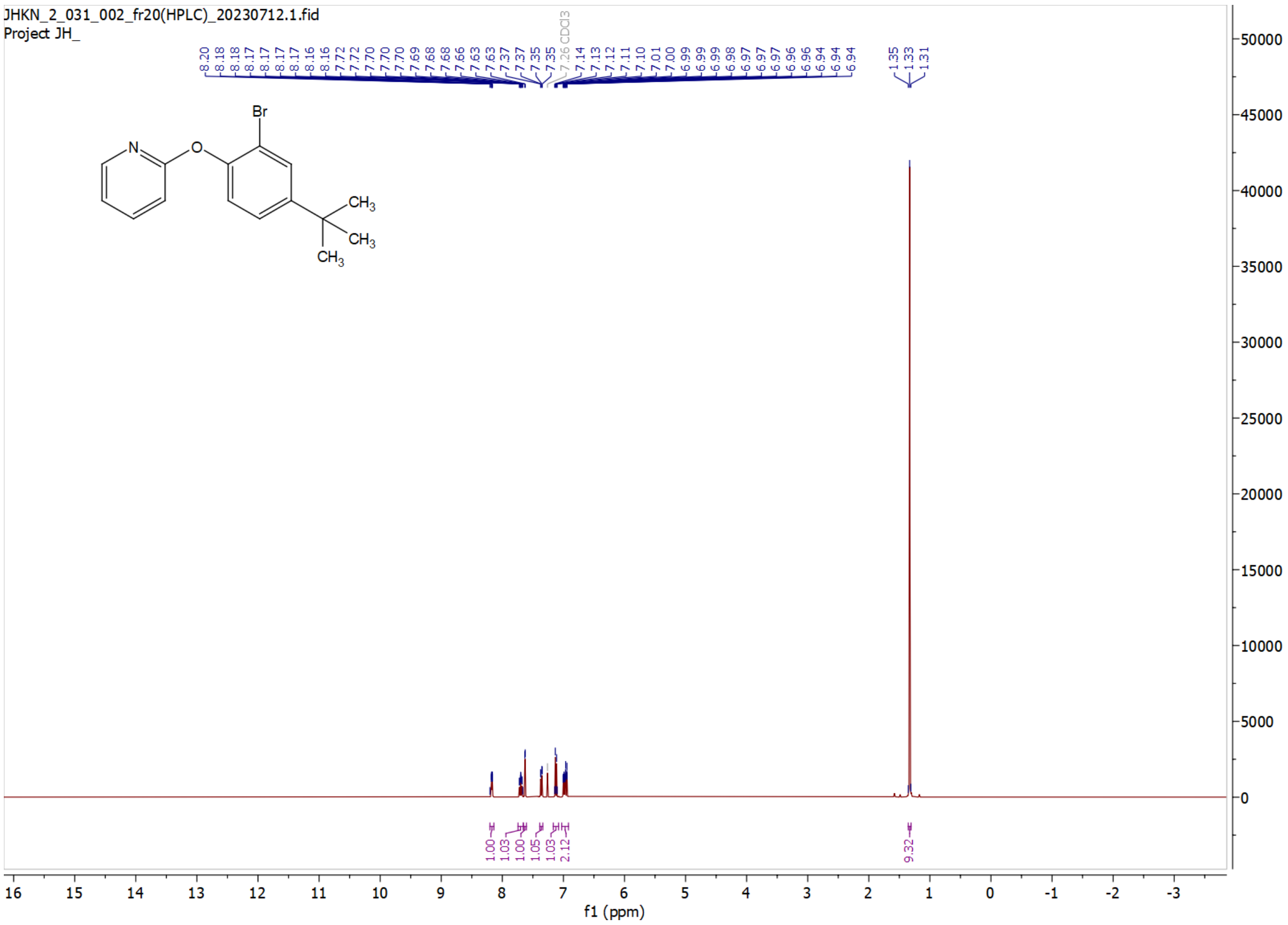
JHKN_2_031_002_fr20(HPLC)_20230712.1.fid
Project JH_
8.20 8.18 8.18 8.17 8.17 8.17 8.16 8.16 7.72 7.72 7.70 7.70 7.70 7.69 7.68 7.66 7.63 7.63 7.37 7.37 7.35 7.35 7.26 CDCl3 7.14 7.13 7.12 7.11 7.10 7.01 7.00 6.99 6.99 6.98 6.98 6.97 6.97 6.96 6.96 6.94 6.94
1.35 1.33 1.31
Br
N
O
CH3
CH3
CH3
1.00 1.03 1.00 1.05 1.03 2.12
9.32
f1 (ppm)

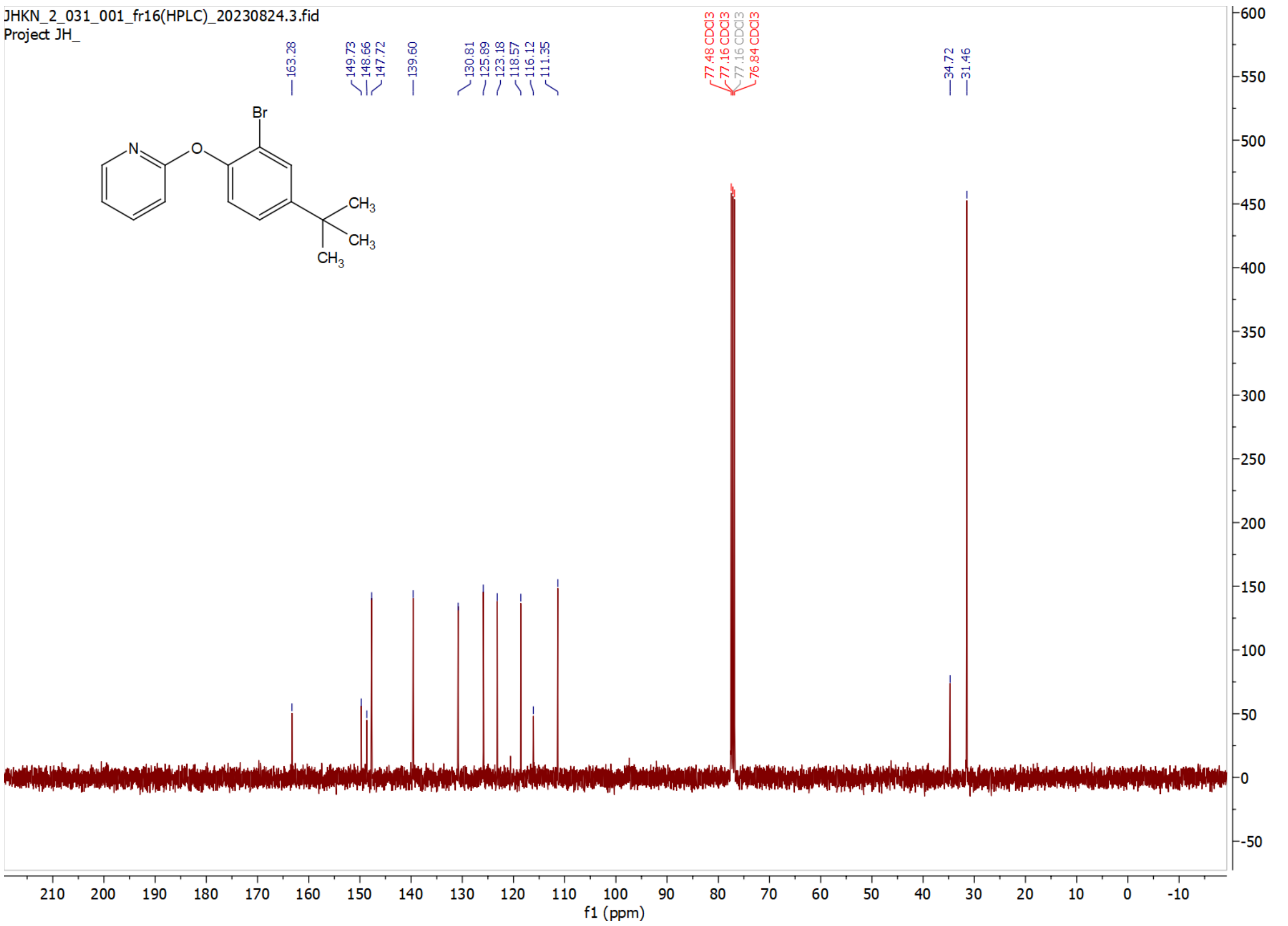
JHKN_2_031_001_fr16(HPLC)_20230824.3.fid
Project JH_
163.28
149.73 148.66 147.72
139.60
130.81 125.89 123.18 118.57 116.12 111.35
77.48 CDCl3 77.16 CDCl3 77.16 CDCl3 76.84 CDCl3
34.72 31.46
Br
N
O
CH3
CH3
CH3
f1 (ppm)

D:\Data\Karoline\LCMS-2022-06-03\JHKN_2_031_001_fr16(HPLC)_... 9/5/2023 9:54:16 AM

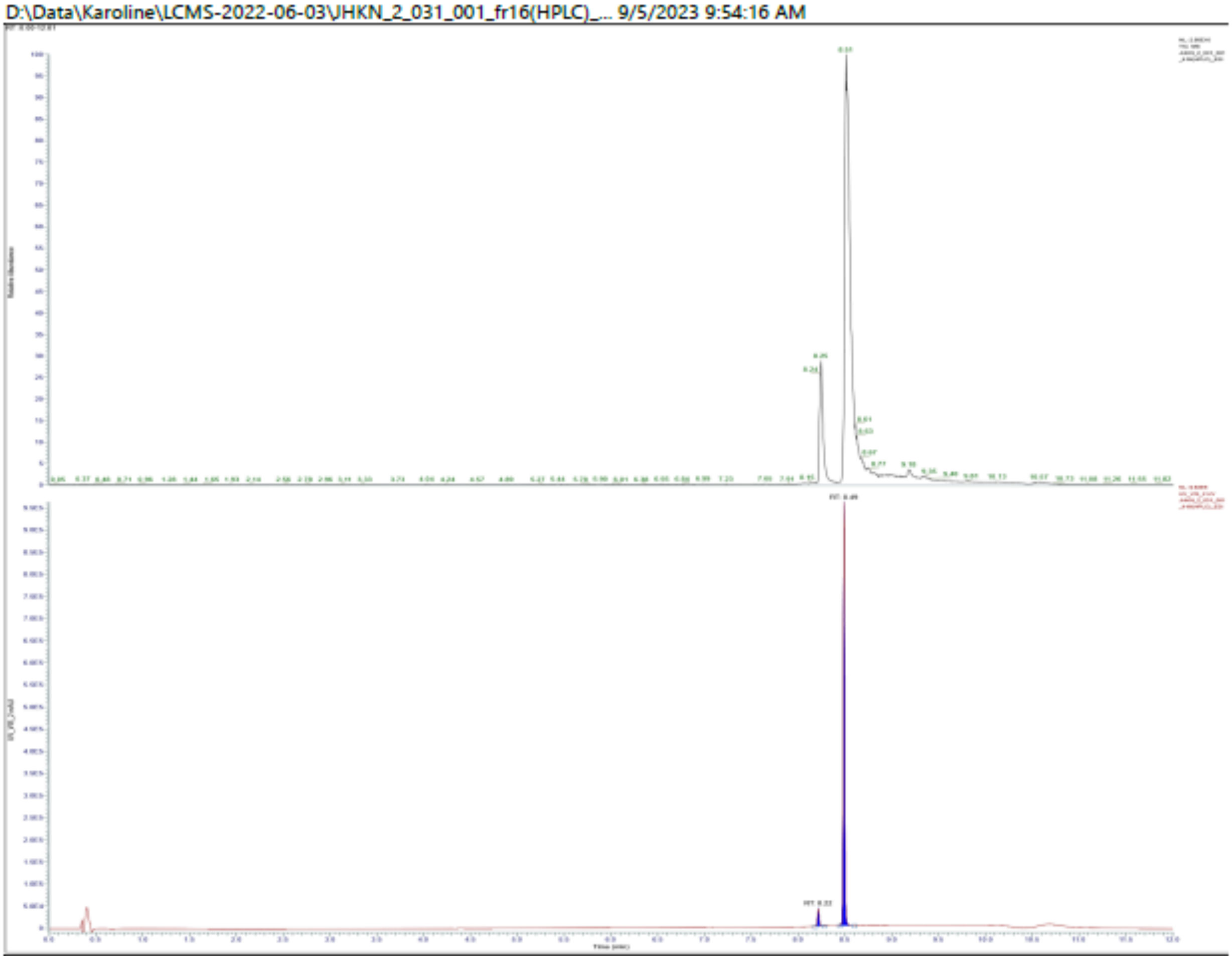

JHKN_2_031_001_fr16(HPLC)_ESI #1922 RT: 8.55 AV: 1 NL: 6.56E9
T: FTMS + c ESI Full ms [100.0000-1000.0000]

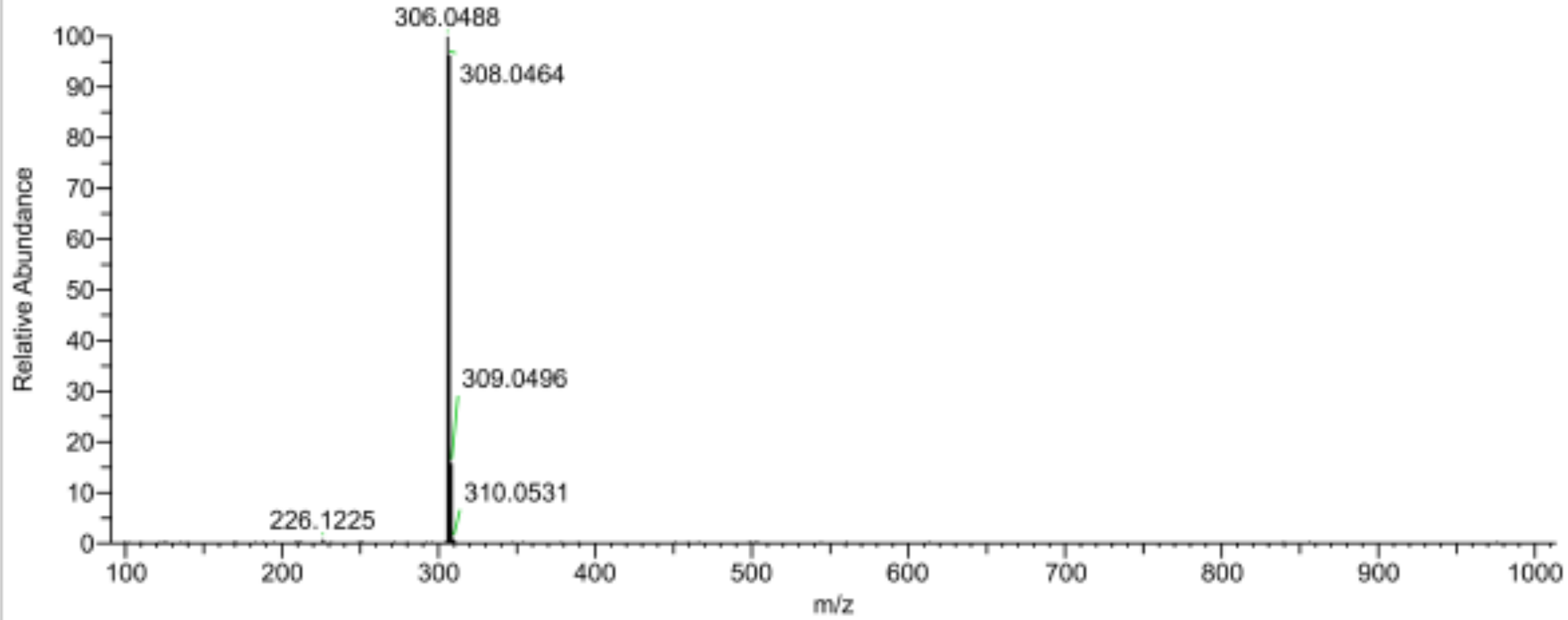

Index	% Area	RT (M...	RT (Sec)	Start RT	End RT	Base...	Peak...	Peak...	Baseli...	Signal...	FileN...	Scan...	TraceId	% Hei...	Integr...	Name
1	4.6	8.21...	493....	8.18...	8.27...	0	503...	398...	0.09...	3.01...	D:\D...	No...	Trac...	4	Man...	
2	95.4	8.49...	509....	8.43...	8.59...	0	104...	956...	0.16...	7.24...	D:\D...	No...	Trac...	96	Man...	

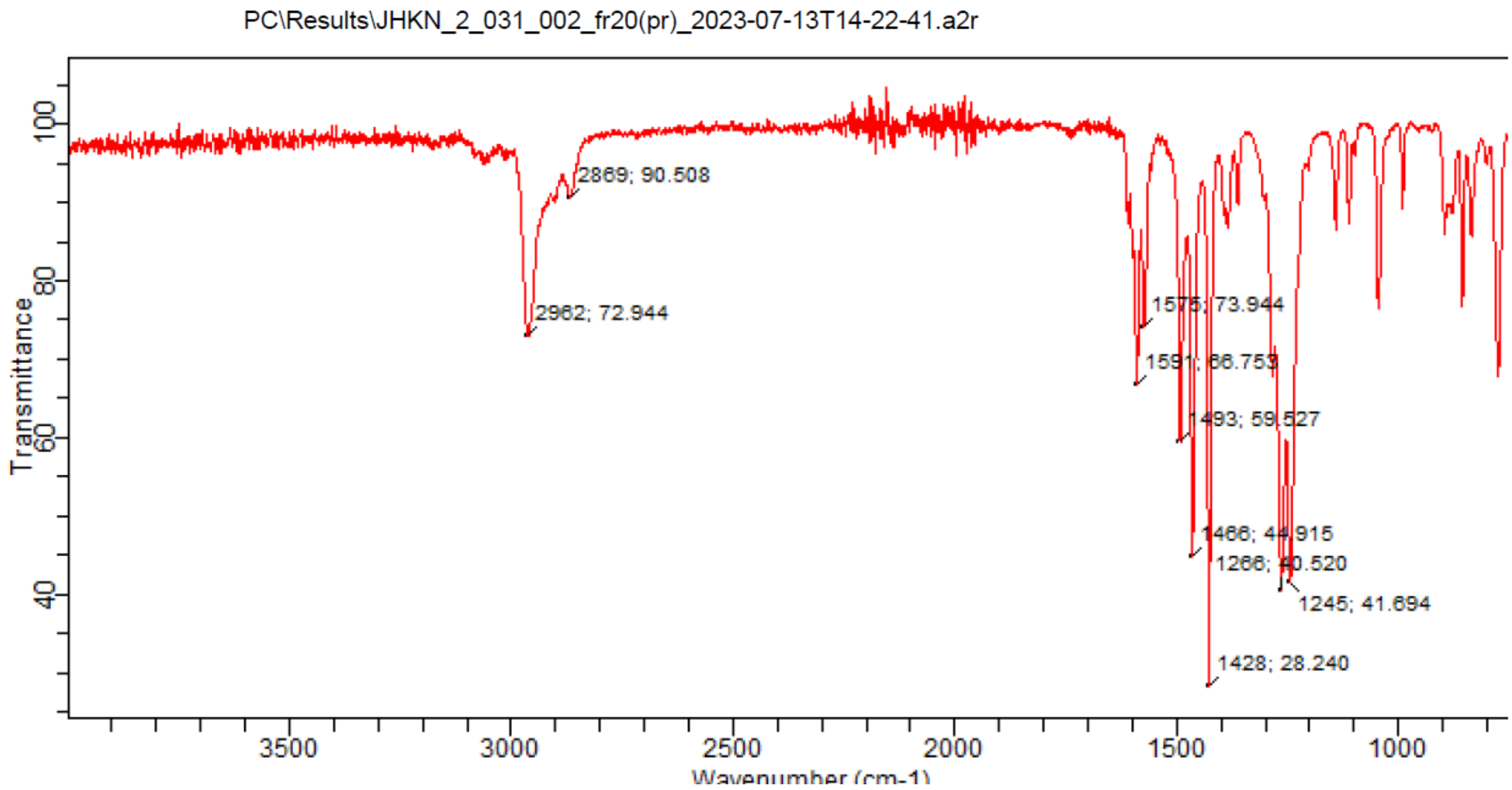
PC\Results\JHKN_2_031_002_fr20(pr)_2023-07-13T14-22-41.a2r
Transmittance
100
80
60
40
2869; 90.508
2962; 72.944
1575; 73.944
1591; 66.753
1493; 59.527
1466; 44.915
1266; 40.520
1245; 41.694
1428; 28.240
3500
3000
2500
2000
1500
1000
Wavenumber (cm-1)

1e

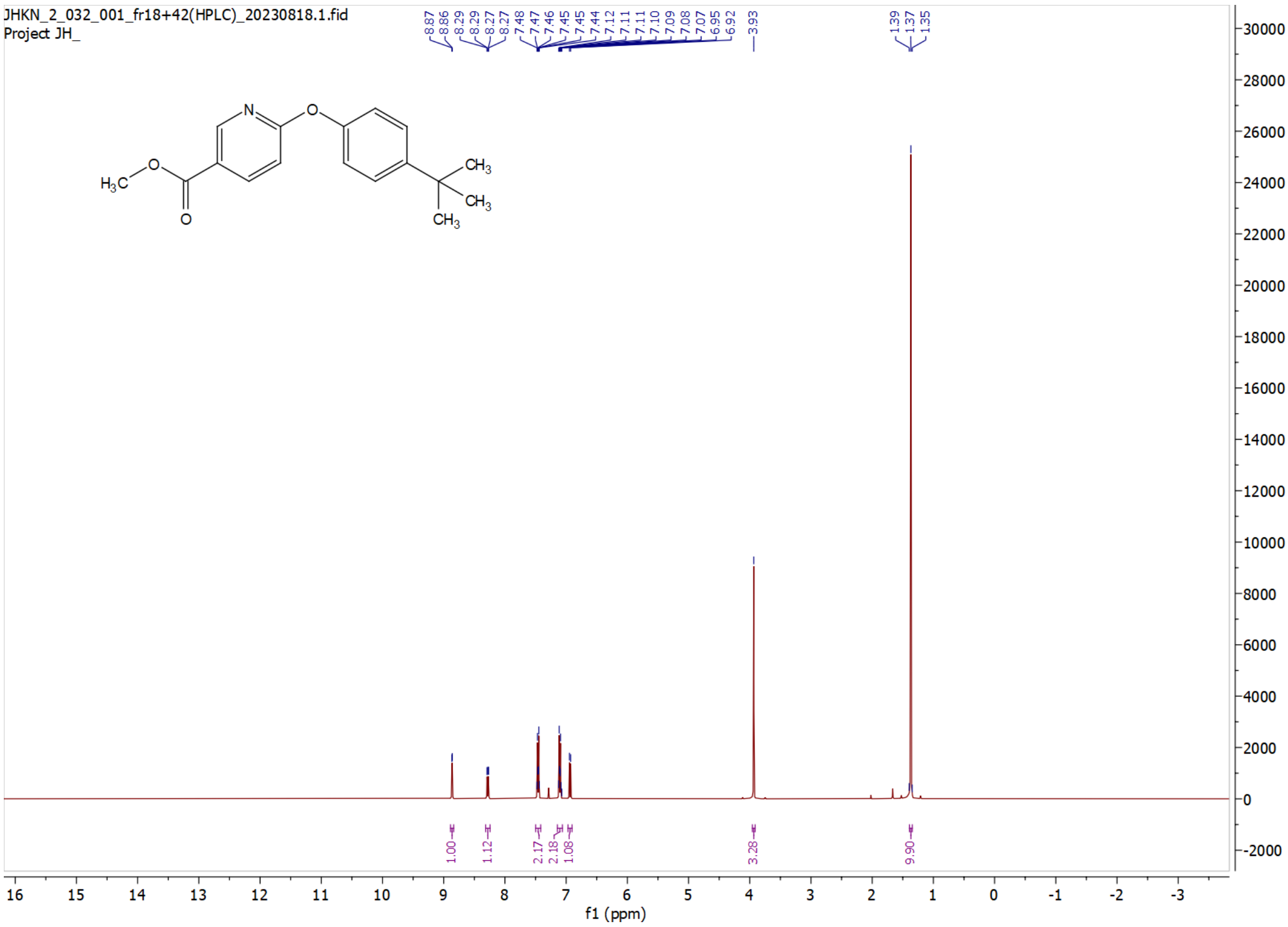

JHKN_2_032_001_fr18+42(HPLC)_20230818.1.fid
Project JH_
8.87 8.86 8.29 8.29 8.27 8.27 7.48 7.47 7.46 7.45 7.45 7.44 7.12 7.11 7.11 7.10 7.09 7.08 7.07 6.95 6.92
3.93
1.39 1.37 1.35
1.00 1.12 2.17 2.18 1.08 3.28 9.90
f1 (ppm)

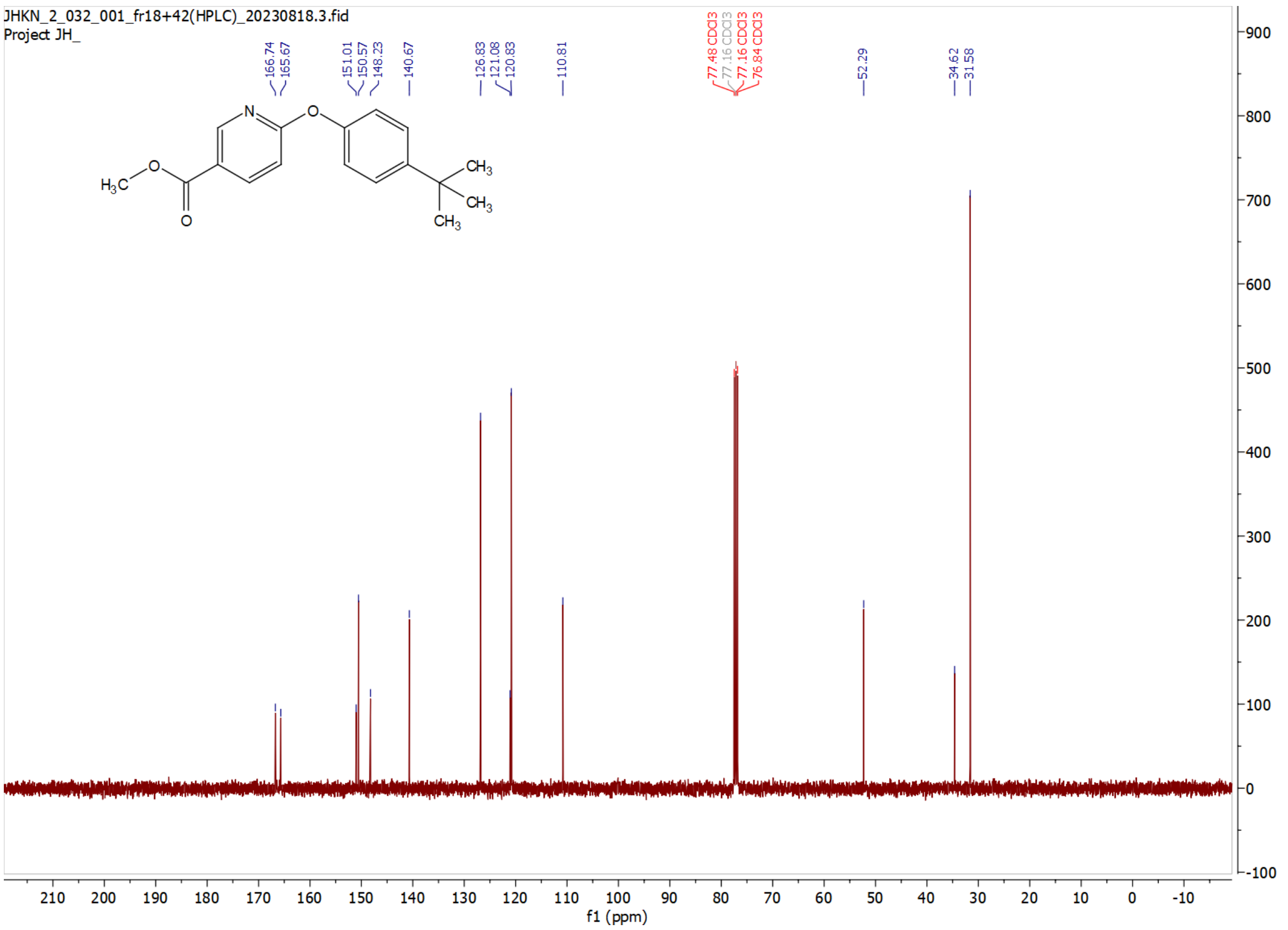

JHKN_2_032_001_fr18+42(HPLC)_20230818.3.fid
Project JH_
166.74 165.67 151.01 150.57 148.23 140.67 126.83 121.08 120.83 110.81
77.48 CDCl3 77.16 CDCl3 77.16 CDCl3 76.84 CDCl3
52.29 34.62 31.58
f1 (ppm)

D:\Data\Karoline\LCMS-2022-06-03\JHKN_2_032_001_fr5_ESI.raw 12/12/2022 1:57:41 PM

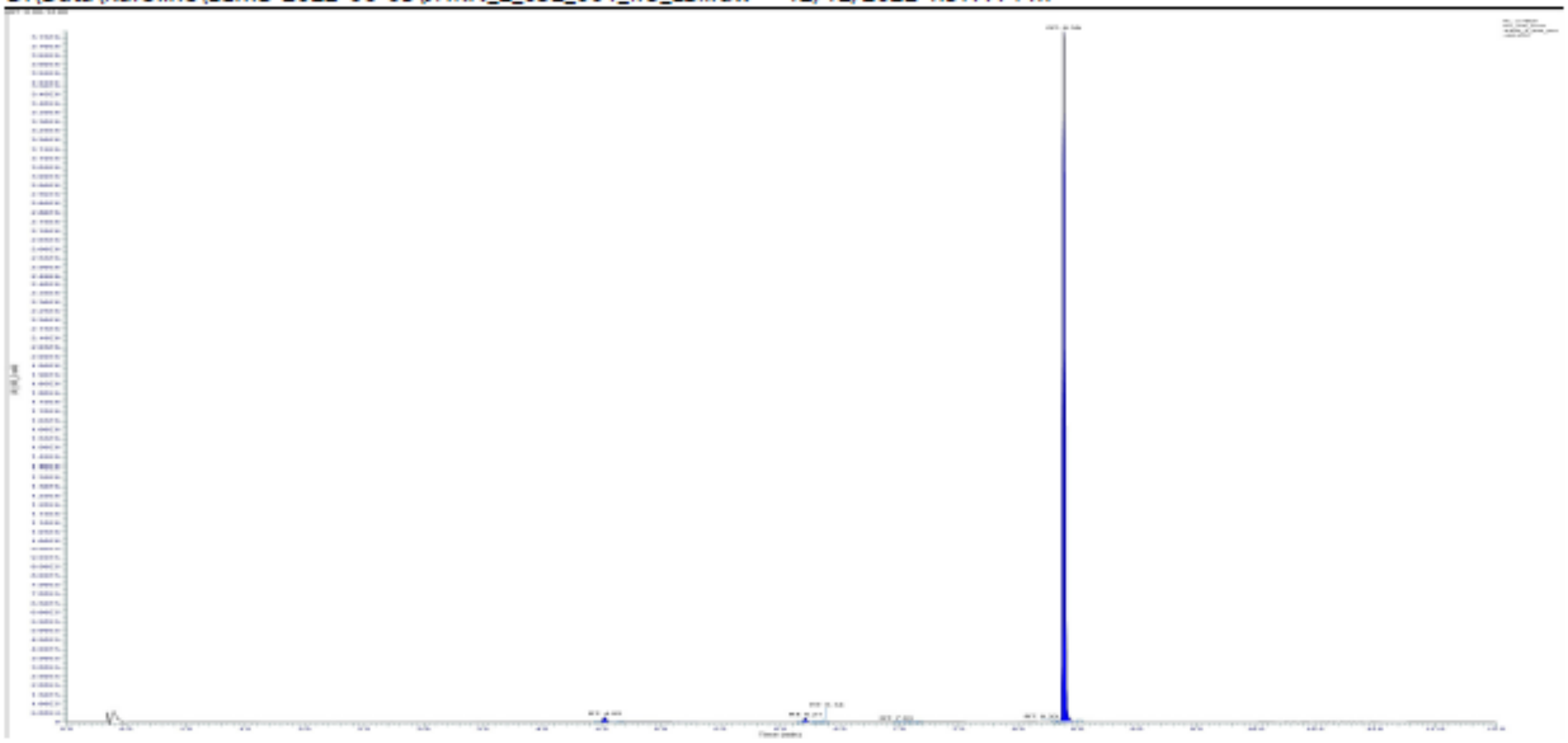

JHKN_2_032_001_fr5_ESI #1886 RT: 8.39 AV: 1 NL: 2.53E9
T: FTMS + c ESI Full ms [100.0000-1000.0000]

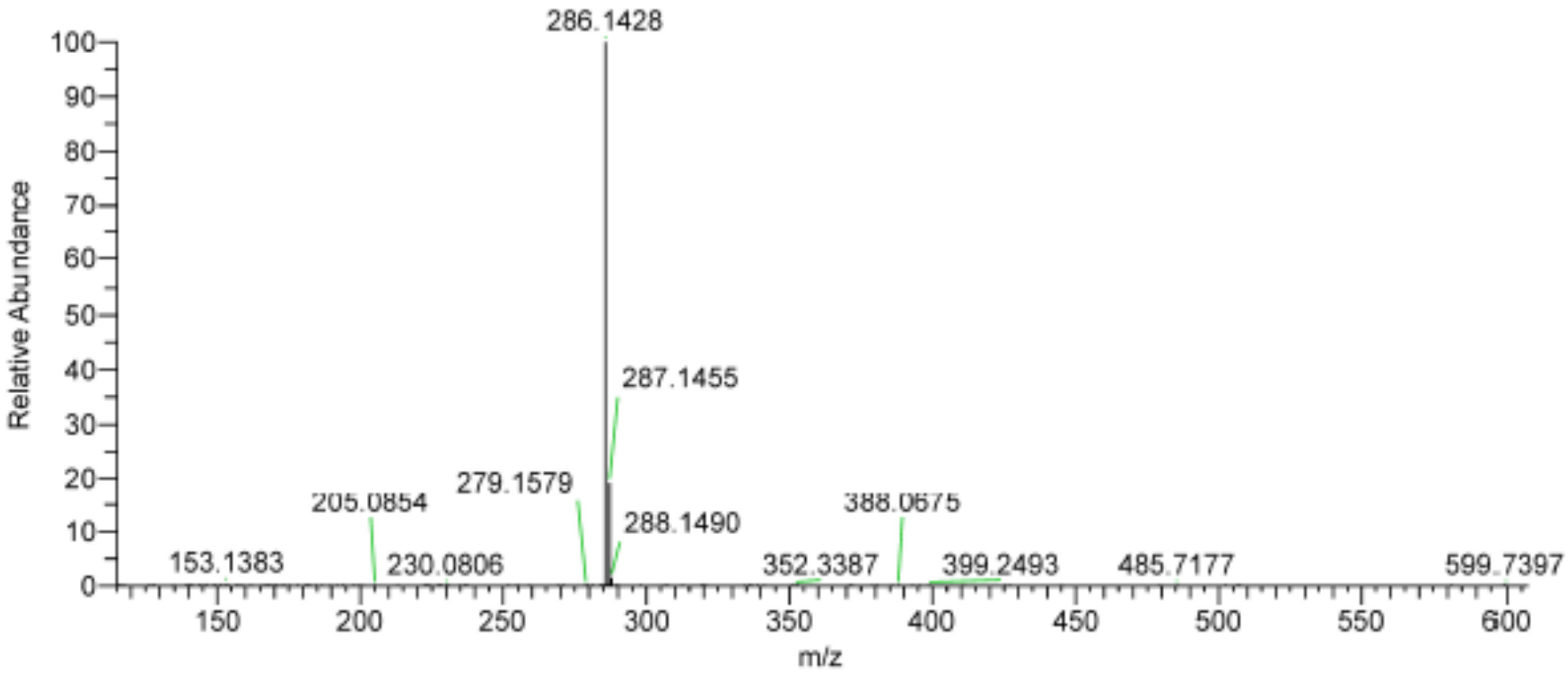

Index	% Area	RT (M...	RT (Sec)	Start RT	End RT	Base...	Peak...	Peak...	Baseli...	Signal...	FileN...	Scan...	TraceId	% Hei...	Integr...	Name
1	1.1	4.52...	271.3	4.44...	4.64...	0	558...	265...	0.20...	2.50...	D:\D...	No...	Trac...	0.69	Man...	
2	0.74	6.21...	372.7	6.15...	6.29...	0	375...	209...	0.13...	1.98...	D:\D...	No...	Trac...	0.55	Man...	
3	0.01	6.34...	380.6	6.31...	6.37...	0	755....	407....	0.05...	3.85...	D:\D...	No...	Trac...	0.01	Man...	
4	0.02	7.025	421.5	6.97...	7.05...	0	112...	348....	0.08...	3.29...	D:\D...	No...	Trac...	0.01	Man...	
5	0.01	7.14...	428.6	7.11...	7.16...	0	440....	324....	0.04...	3.06...	D:\D...	No...	Trac...	0.01	Man...	
6	0.09	8.33...	499.9	8.30...	8.35...	0	482...	459...	0.04...	4.36	D:\D...	No...	Trac...	0.12	Man...	
7	98.03	8.38...	503.2	8.35...	8.51...	0	499...	377...	0.15...	3.55...	D:\D...	No...	Trac...	98.61	Man...	

4

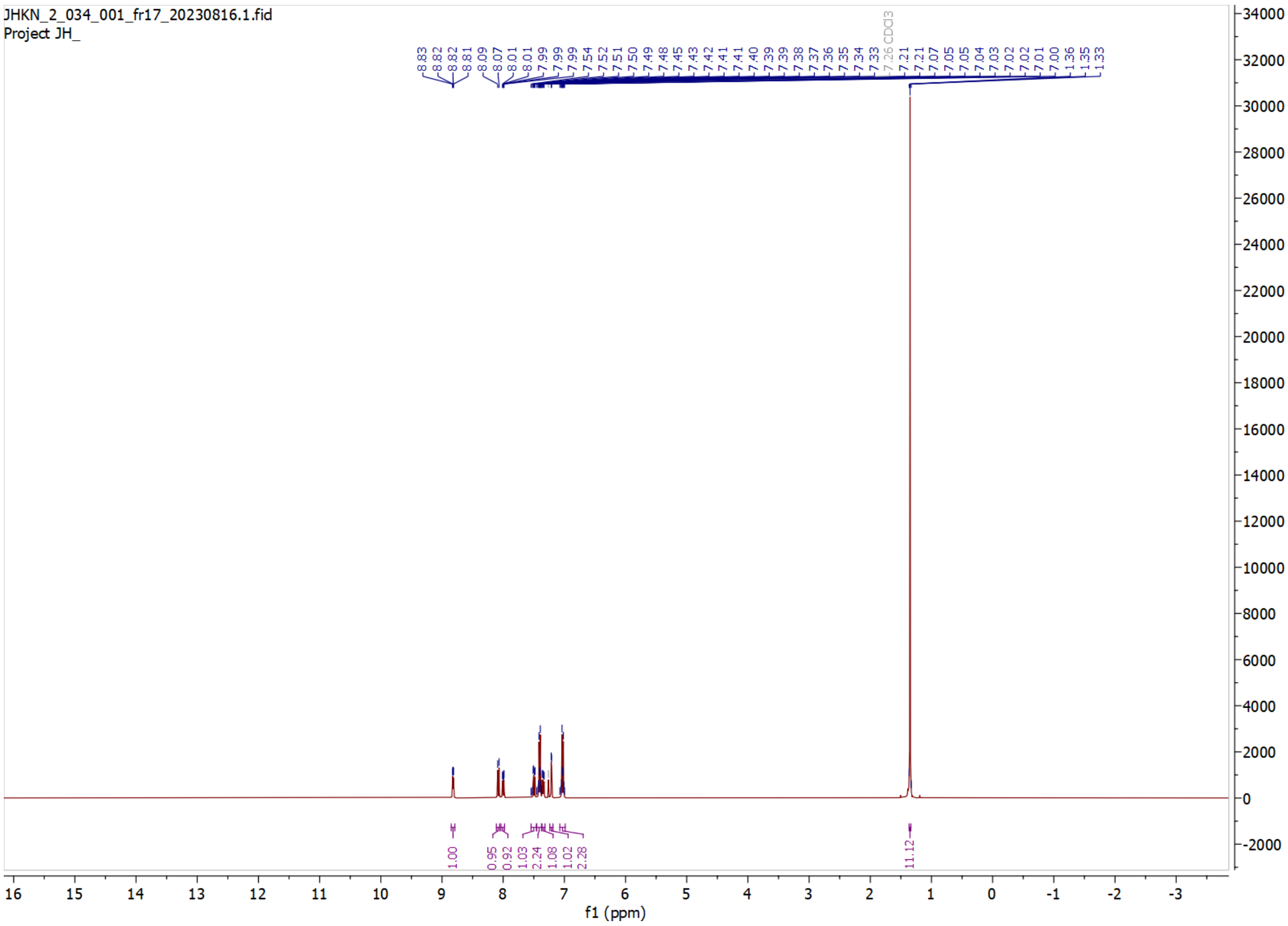
JHKN_2_034_001_fr17_20230816.1.fid
Project JH_
f1 (ppm)

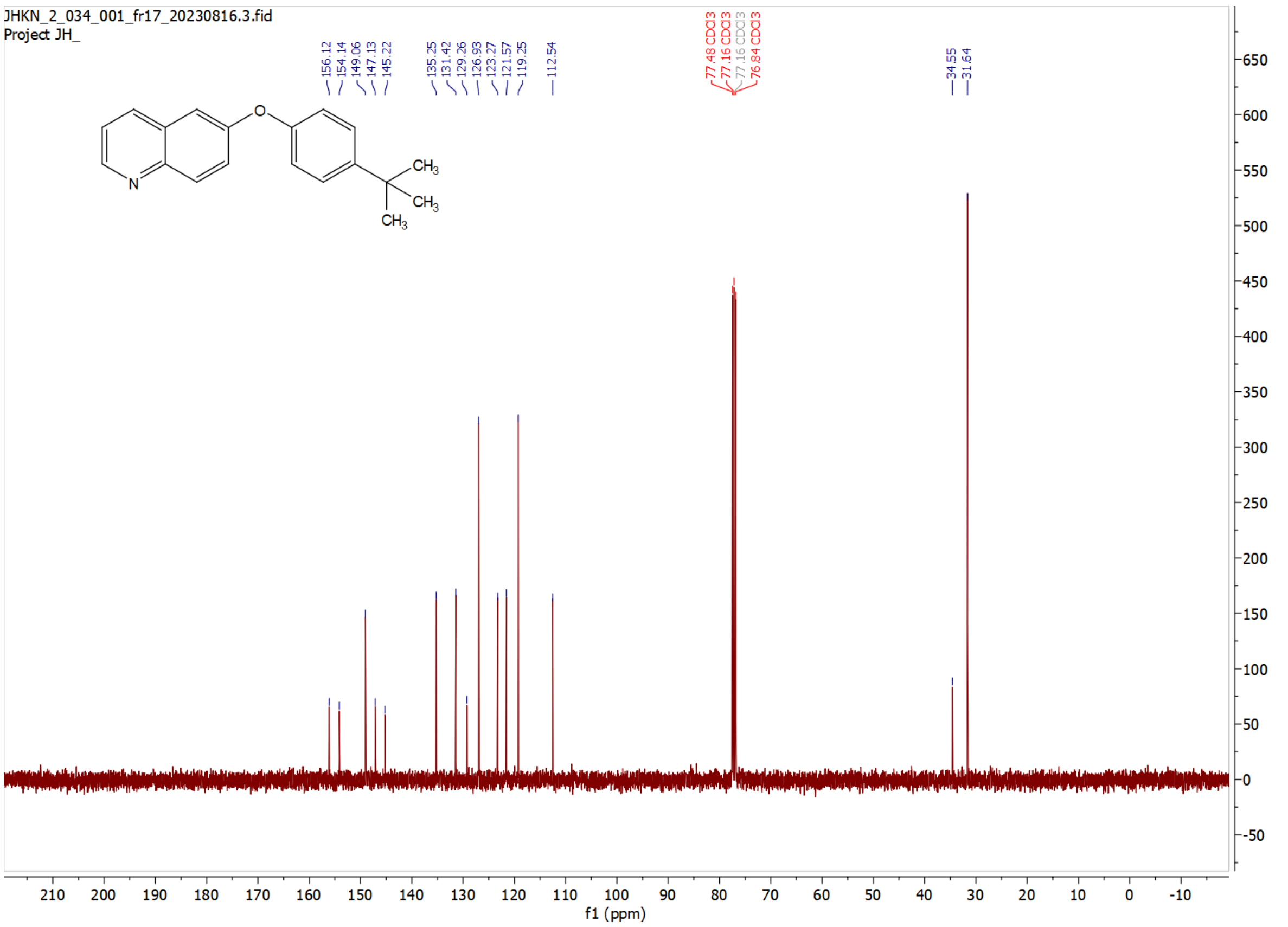
JHKN_2_034_001_fr17_20230816.3.fid
Project JH_
f1 (ppm)

D:\Data\Karoline\LCMS-2022-06-03\JHKN_2_034_001_fr10-21_ESI.raw 1/10/2023 9:52:26 AM

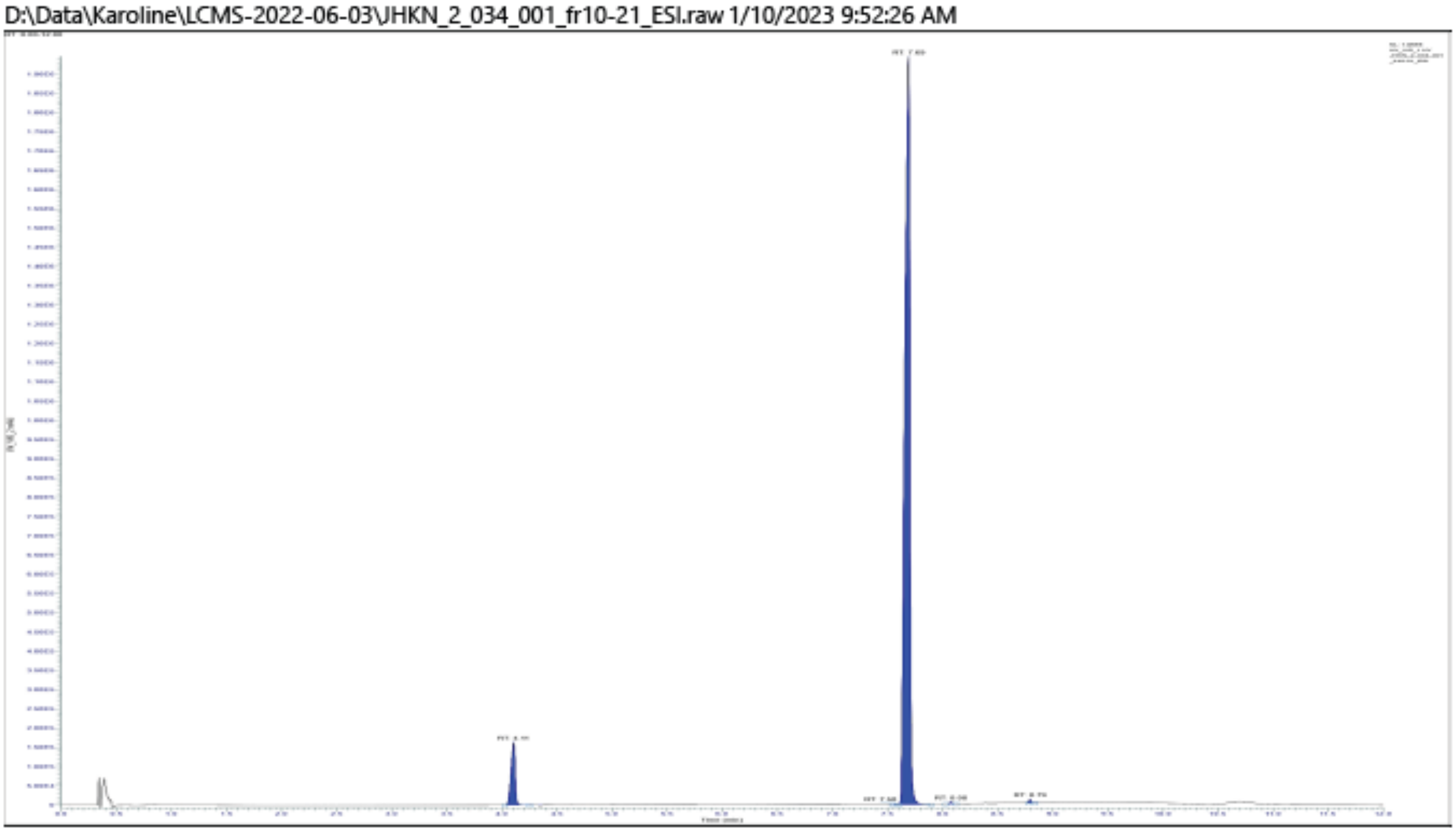

JHKN_2_034_001_fr10-21_ESI #1735 RT: 7.72 AV: 1 NL: 1.98E10
T: FTMS + c ESI Full ms [100.0000-1000.0000]

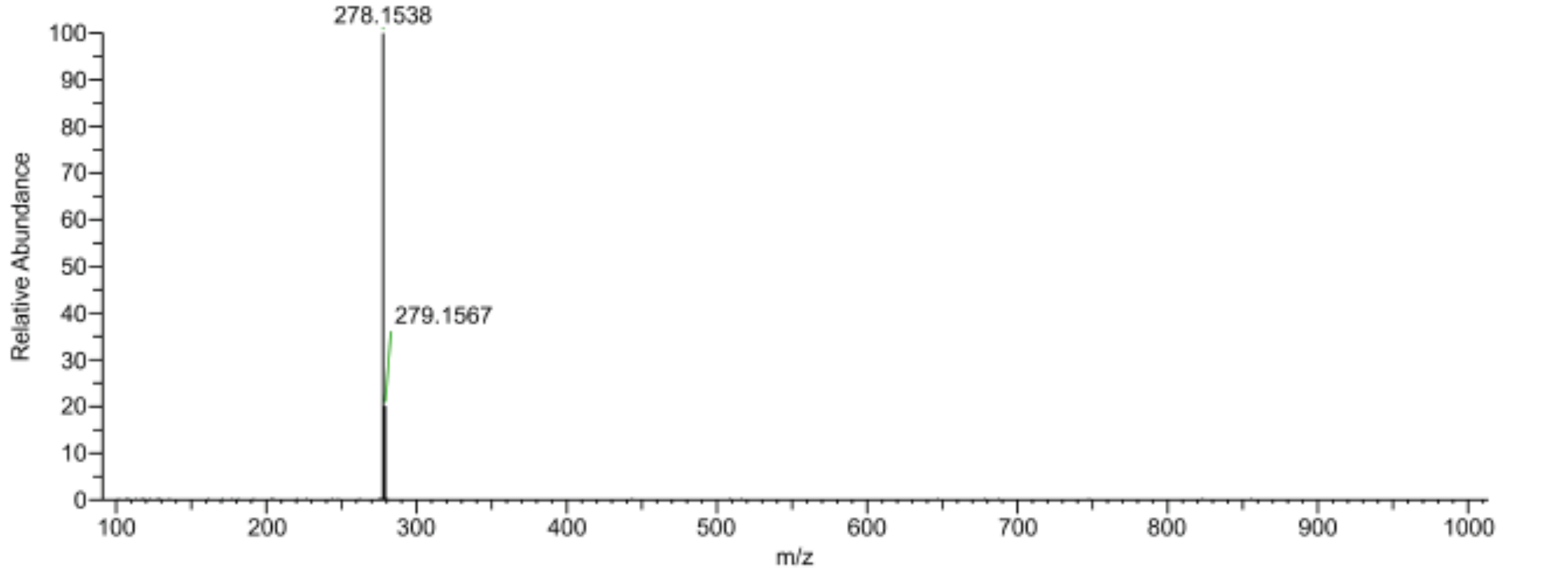

Index	% Area	RT (Mi...	RT (Sec)	Start RT	End RT	Base P...	Peak...	Peak...	Baseli...	Signal...	FileNa...	Scan F...	TraceId	% Hei...	Integr...	Name
1	6.48	4.10...	246....	4.02...	4.24...	0	4120...	1626...	0.22...	1.45E2	D:\D...	No F...	Trace1	7.67	Man...	
2	0.04	7.57...	454....	7.54...	7.58...	0	2763...	2278...	0.04...	2.00	D:\D...	No F...	Trace1	0.11	Man...	
3	93.18	7.69...	461....	7.60...	7.89...	0	5928...	1943...	0.29	1.72E3	D:\D...	No F...	Trace1	91.58	Man...	
4	0.12	8.07...	484....	8.022	8.11...	0	7665...	4263...	0.09...	3.81	D:\D...	No F...	Trace1	0.2	Man...	
5	0.18	8.79...	527....	8.77...	8.83...	0	1119...	9494...	0.06...	8.54	D:\D...	No F...	Trace1	0.45	Man...	

File Location: C:\Program Files (x86)\Agilent\MicroLab PC\Results\JHKN_2_034_001_fr17_2023-08-17T12-19-41.a2r

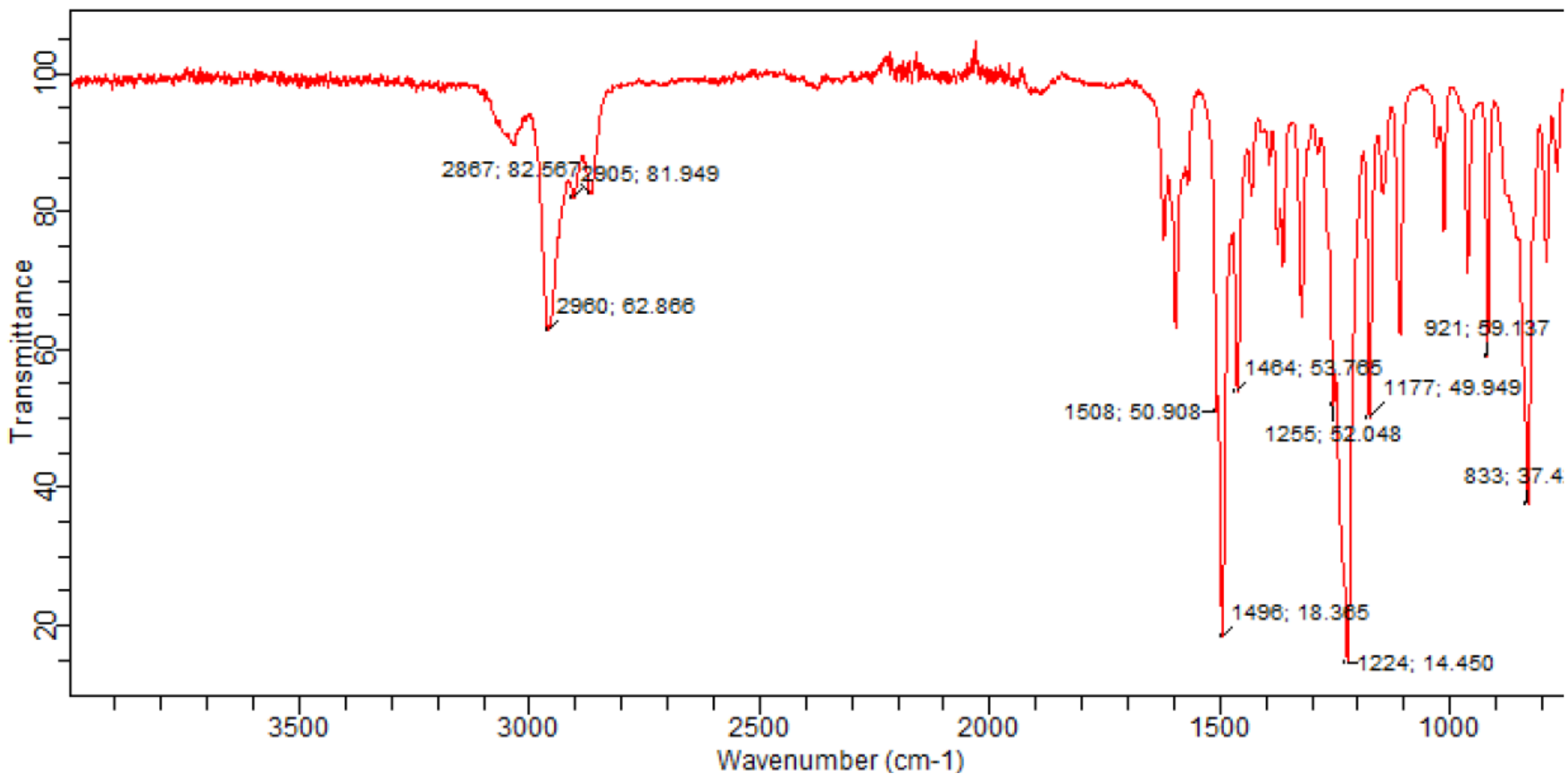

1f

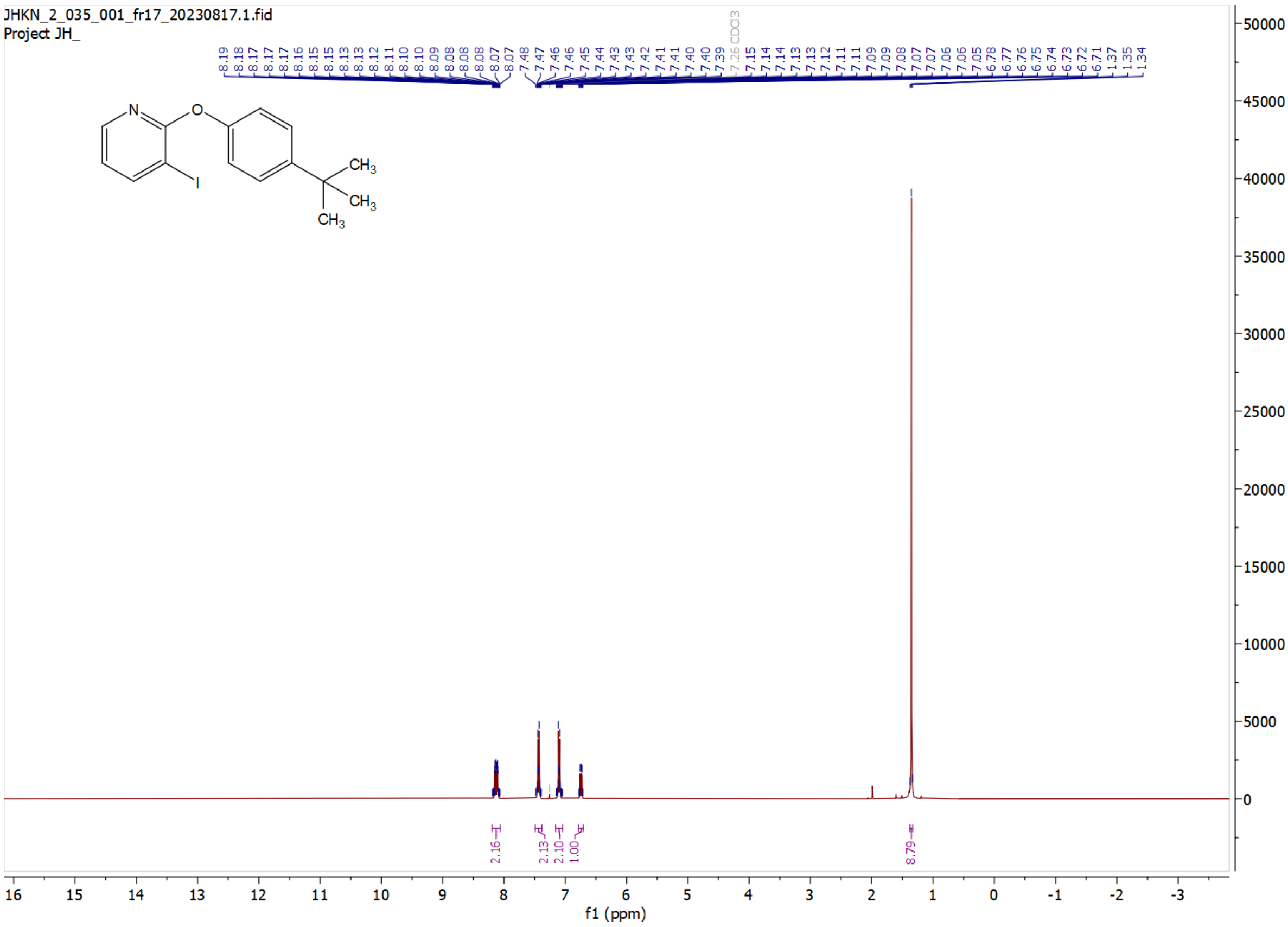
JHKN_2_035_001_fr17_20230817.1.fid
Project JH_
f1 (ppm)

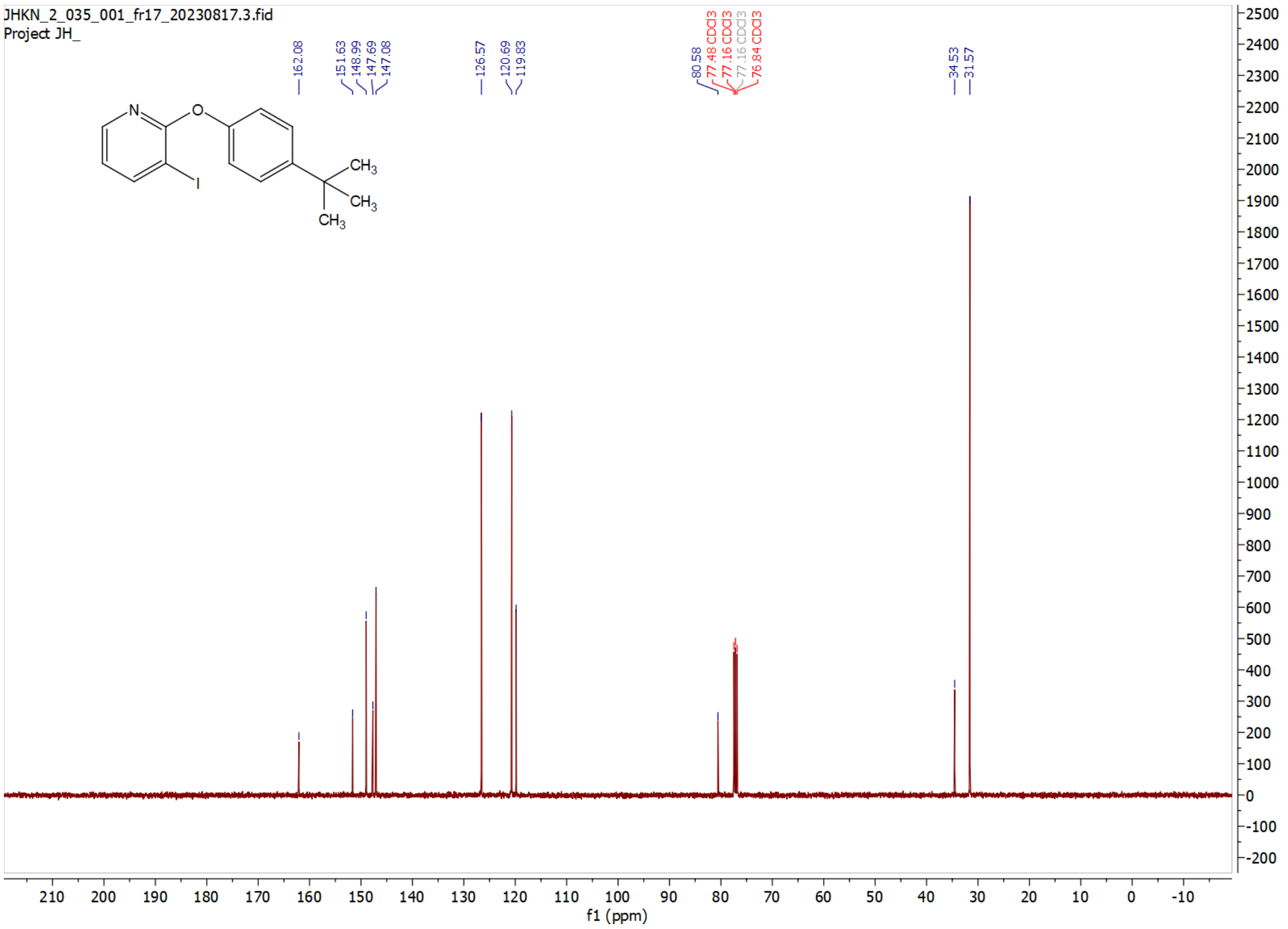
JHKN_2_035_001_fr17_20230817.3.fid
Project JH_
162.08
151.63
148.99
147.69
147.08
126.57
120.69
119.83
80.58
77.48 CDCl3
77.16 CDCl3
77.16 CDCl3
76.84 CDCl3
34.53
31.57
f1 (ppm)

D:\Data\Karoline\LCMS-2022-06-03\JHKN_2_035_001_fr17(HPLC)_ES...8/23/2023 9:21:12 AM

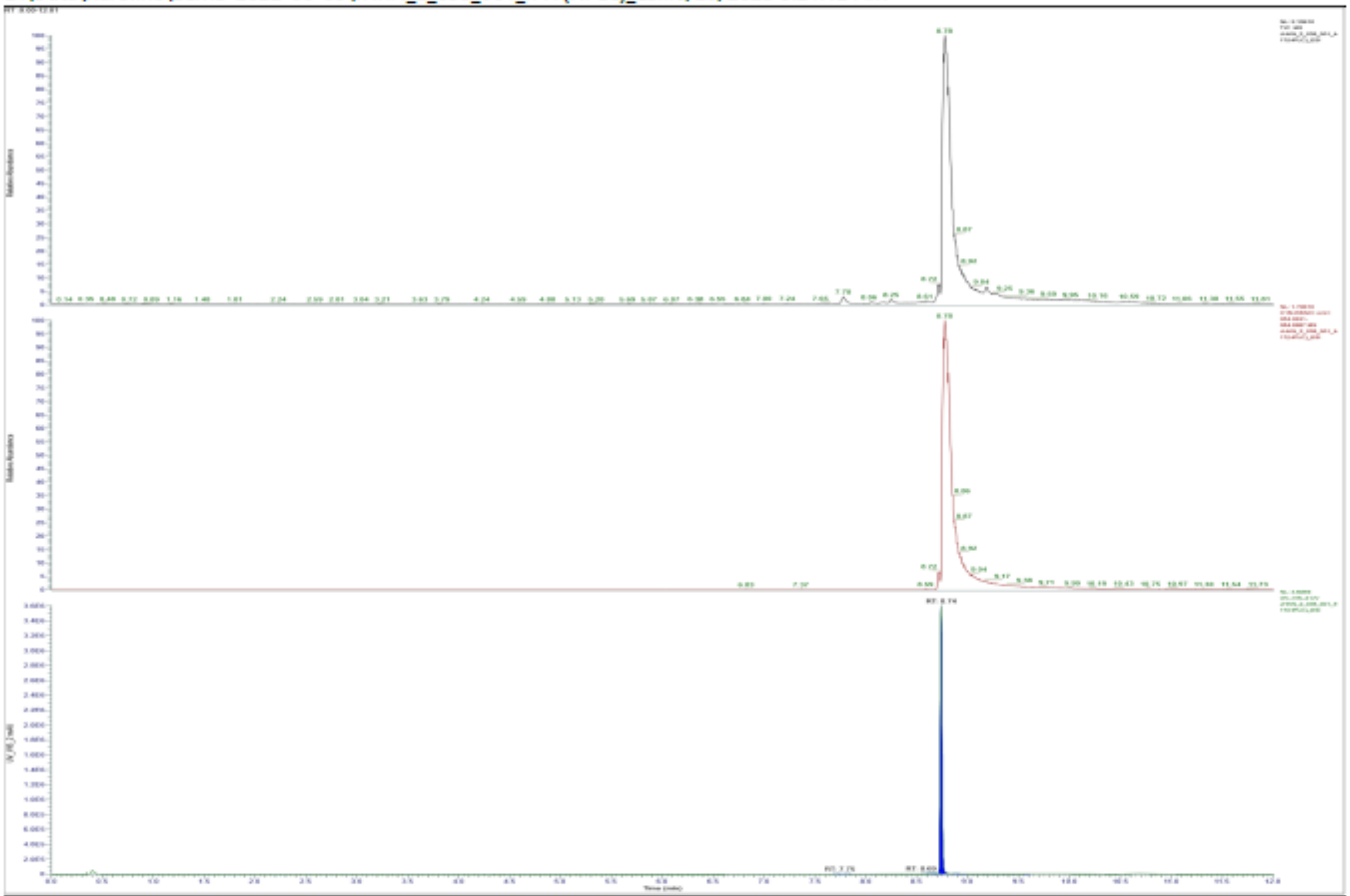

JHKN_2_035_001_fr17(HPLC)_ESI #1977 RT: 8.80 AV: 1 NL: 1.58E10
T: FTMS + c ESI Full ms [100.0000-1000.0000]

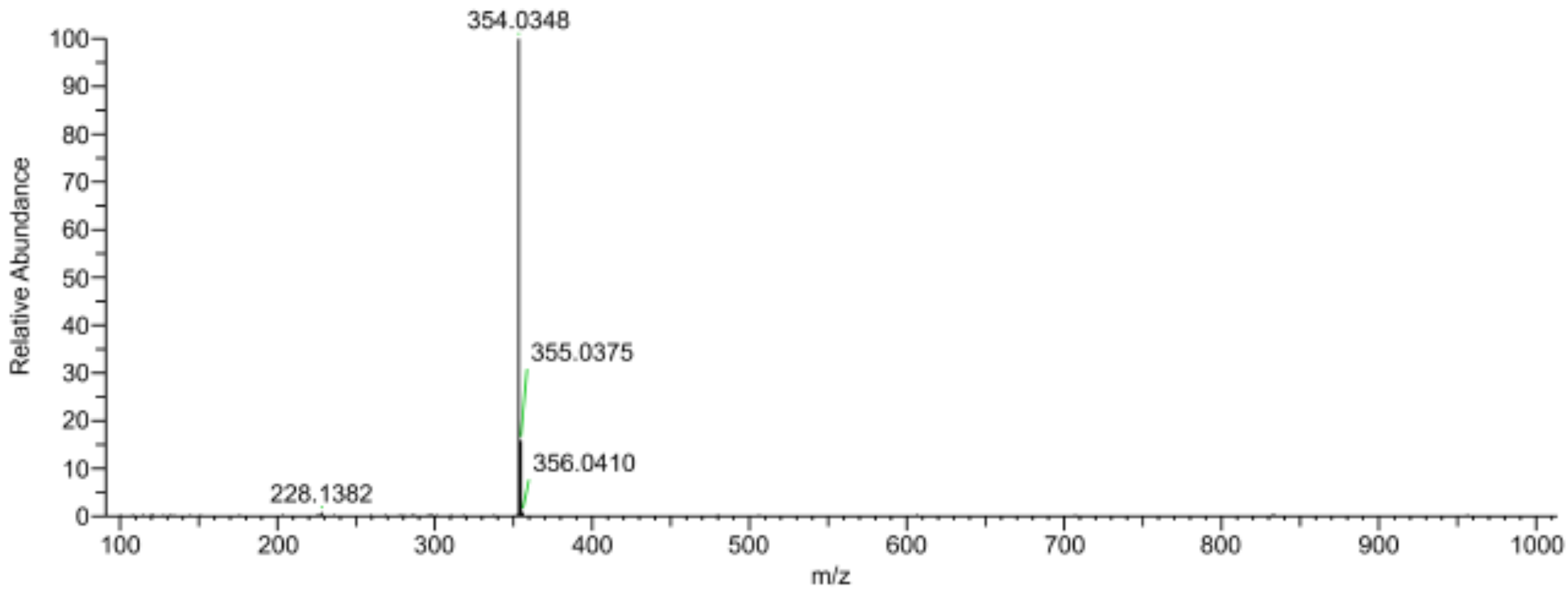

Index	% Area	RT (Mi...	RT (Sec)	Start RT	End RT	Base P...	Peak...	Peak...	Baseli...	Signal...	FileNa...	Scan F...	TraceId	% Hei...	Integr...	Name
1	0.2	7.7484	464....	7.70...	7.80...	0	9074...	4338...	0.10...	3.31	D:\D...	No F...	Trace3	0.12	Man...	
2	0.08	8.68...	521....	8.63...	8.70...	0	3485...	2674...	0.06...	2.06	D:\D...	No F...	Trace3	0.07	Man...	
3	99.72	8.74...	524....	8.71...	8.88...	0	4494...	3596...	0.17...	2.77E3	D:\D...	No F...	Trace3	99.81	Man...	

File Location: C:\Program Files (x86)\Agilent\MicroLab PC\Results\JHKN_2_035_001_fr17_2023-08-17T12-21-45.a2r

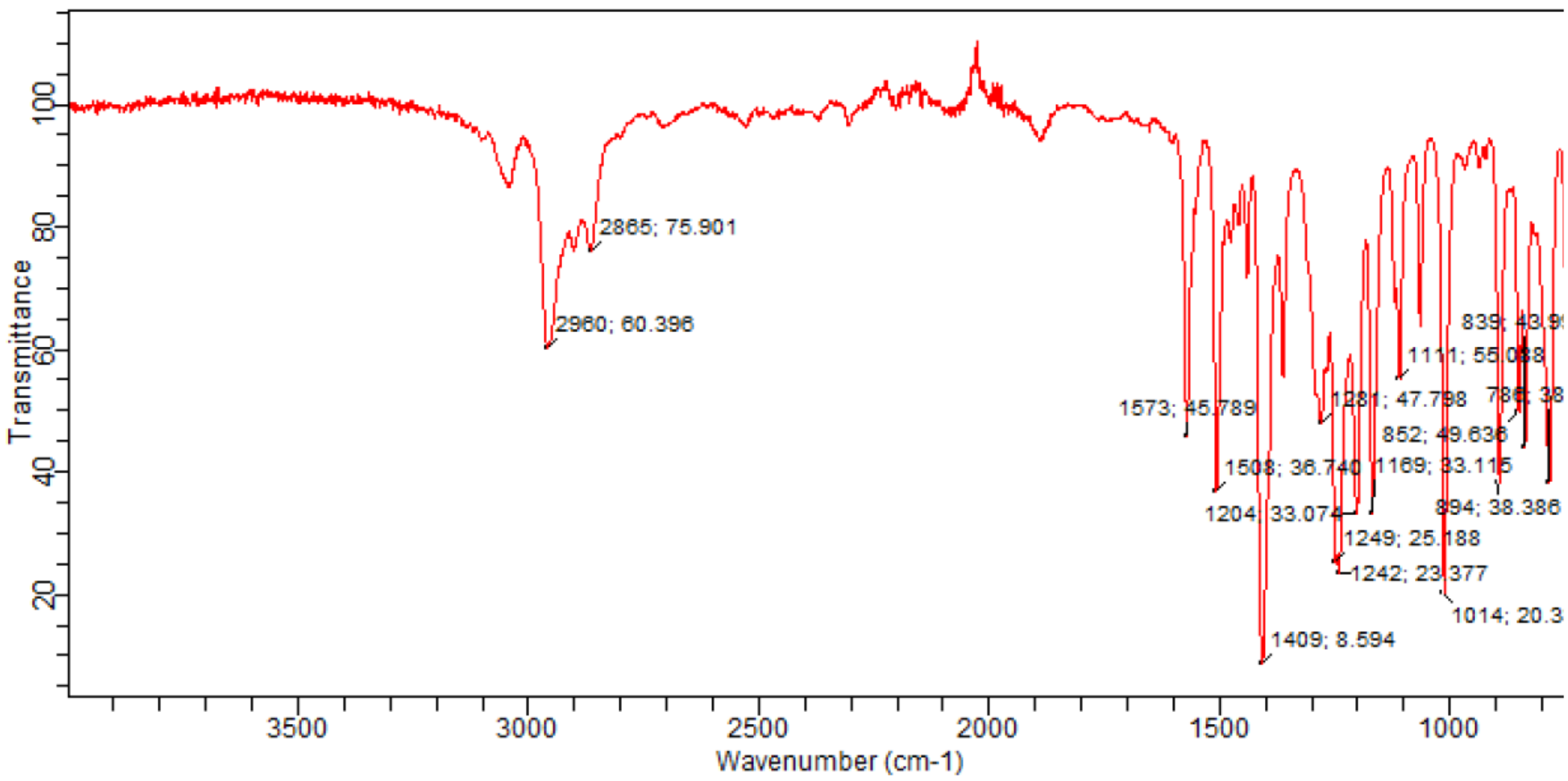

1g

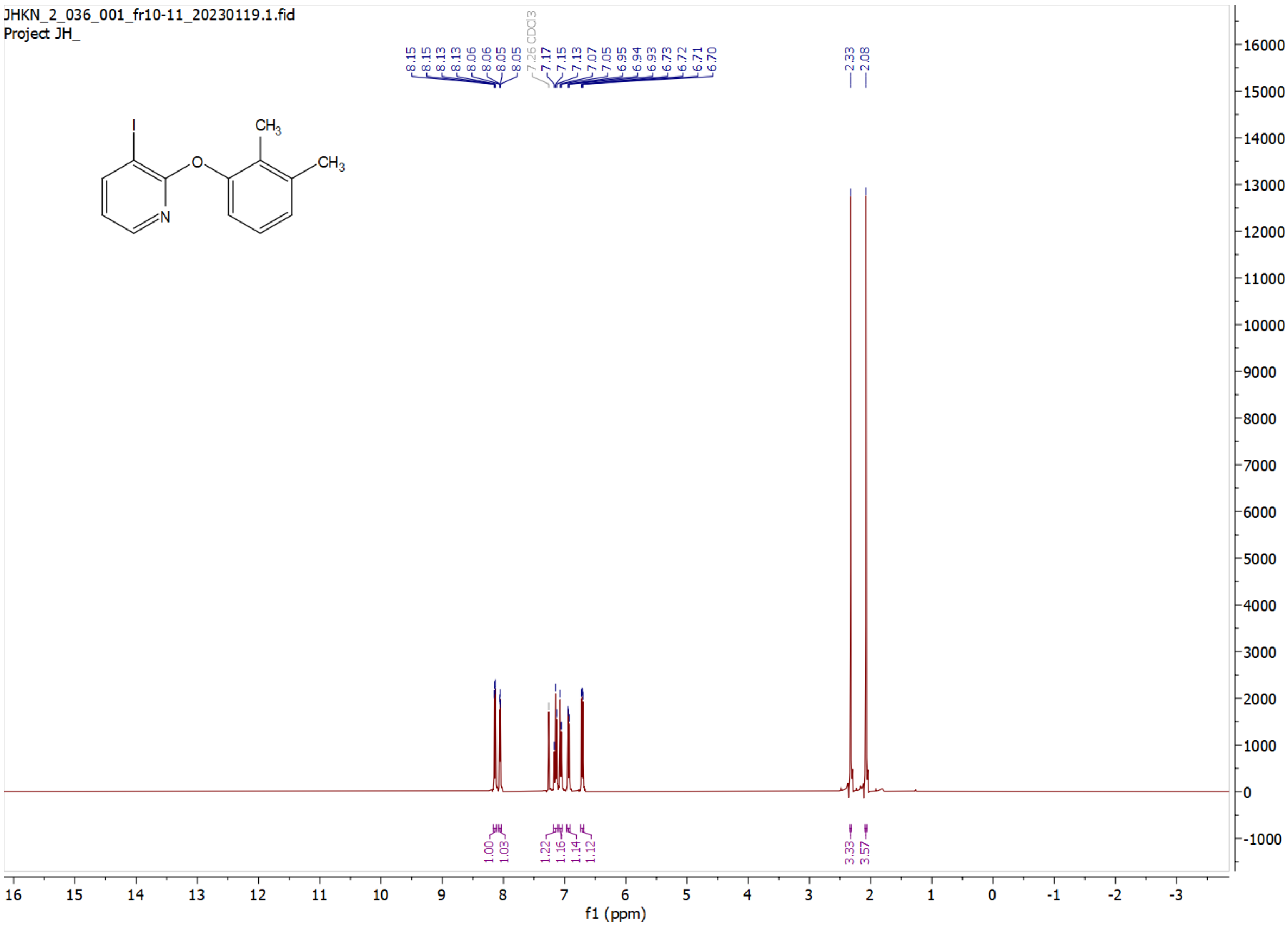

JHKN_2_036_001_fr10-11_20230119.1.fid
Project JH_
f1 (ppm)

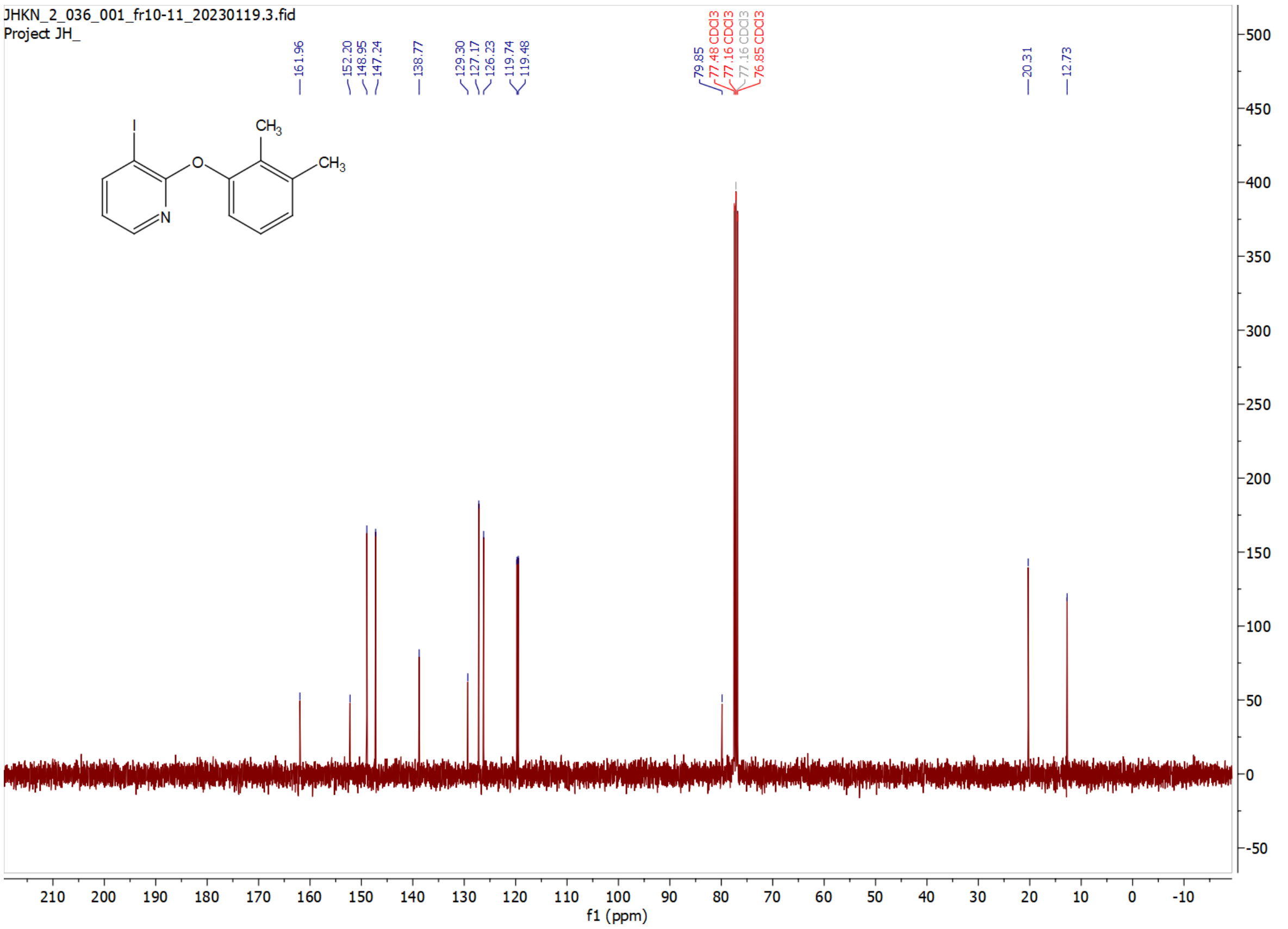

JHKN_2_036_001_fr10-11_20230119.3.fid
Project JH_
f1 (ppm)

D:\Data\Karoline\LCMS-2022-06-03\JHKN_2_036_001_fr48(HPLC)_ES...8/23/2023 9:19:25 AM

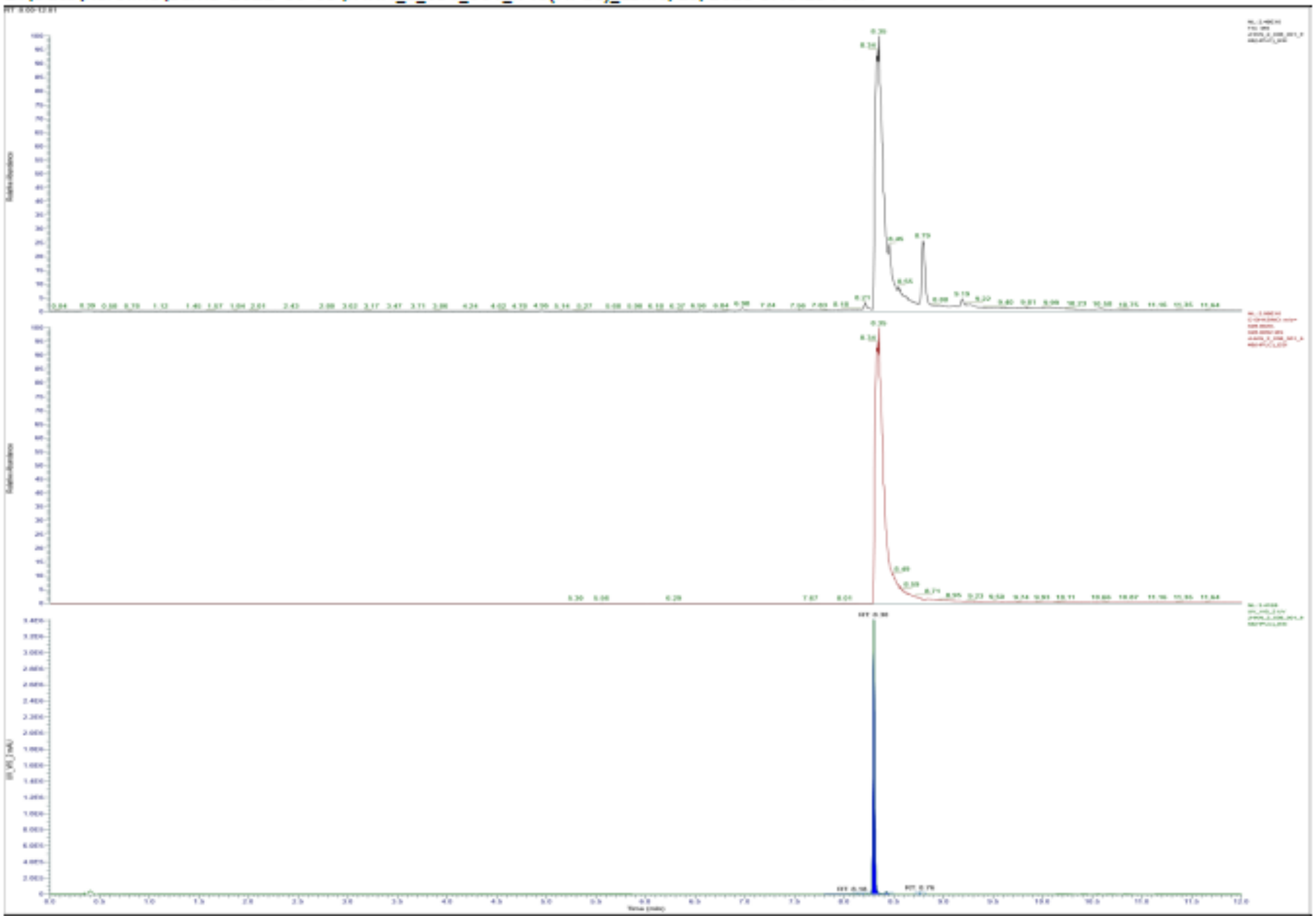

JHKN_2_036_001_fr48(HPLC)_ESI #1888 RT: 8.40 AV: 1 NL: 8.82E9
T: FTMS + c ESI Full ms [100.0000-1000.0000]

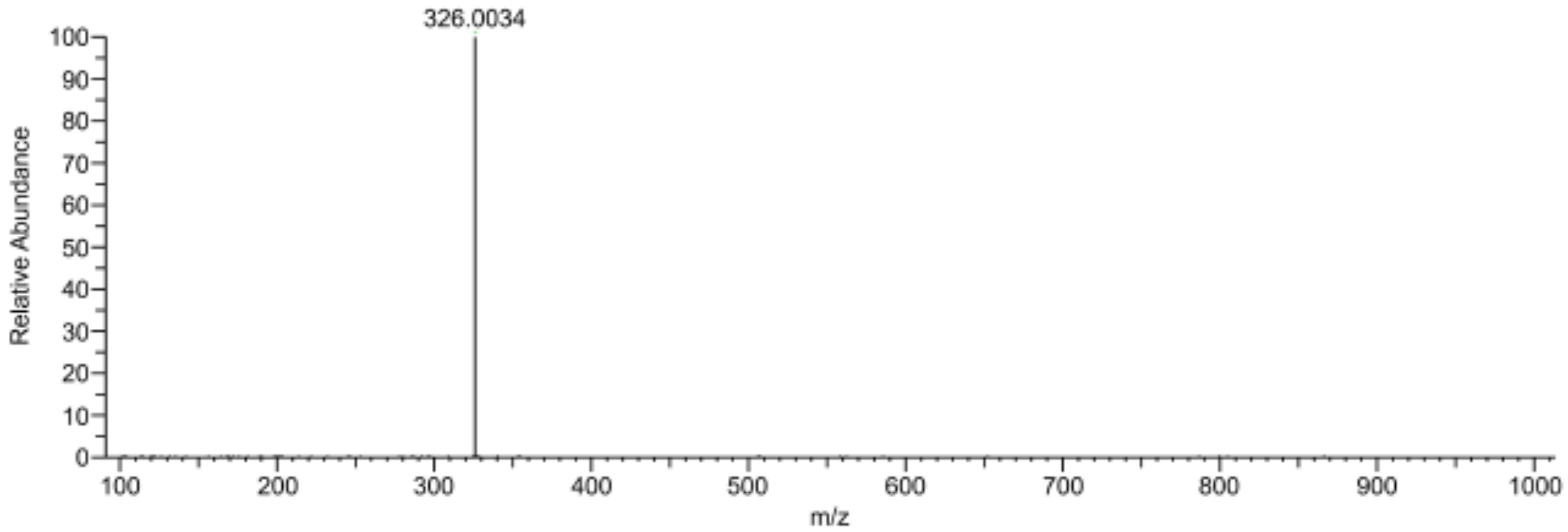

Index	% Area	RT (Mi...	RT (Sec)	Start RT	End RT	Base P...	Peak...	Peak...	Baseli...	Signal...	FileNa...	Scan F...	TraceId	% Hei...	Integr...	Name
1	0.06	8.18...	490....	8.15...	8.20...	0	2463...	1506...	0.04...	1.14	D:\D...	No F...	Trace3	0.04	Man...	
2	99.57	8.30...	498....	8.25...	8.46...	0	4369...	3407...	0.20...	2.59E3	D:\D...	No F...	Trace3	99.53	Man...	
3	0.37	8.76...	525....	8.73...	8.79...	0	1619...	1454...	0.05...	1.10E1	D:\D...	No F...	Trace3	0.42	Man...	

File Location: C:\Program Files (x86)\Agilent\MicroLab PC\Results\JHKN_2_036_001_fr48_2023-08-17T12-23-27.a2r

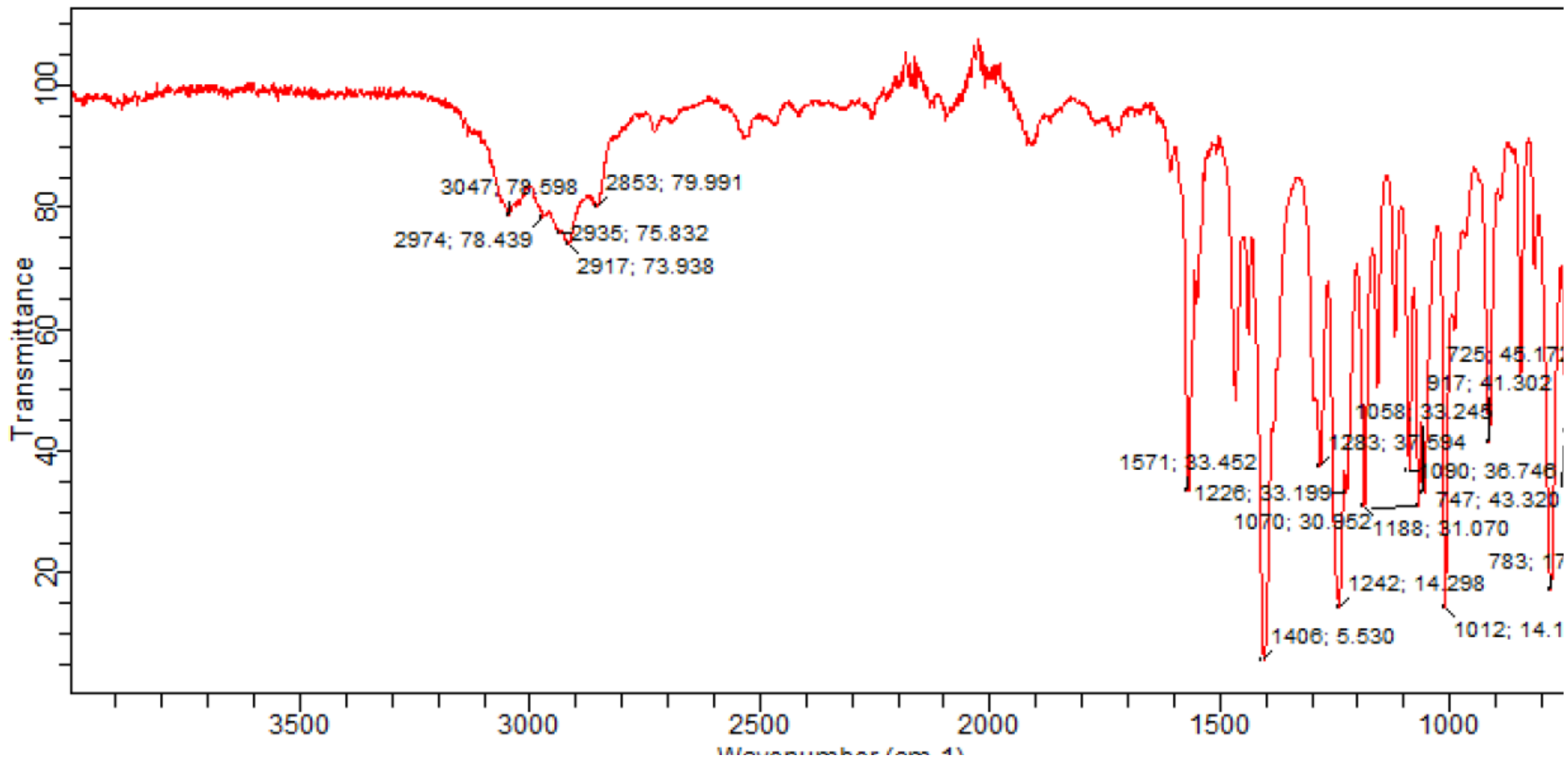

1h

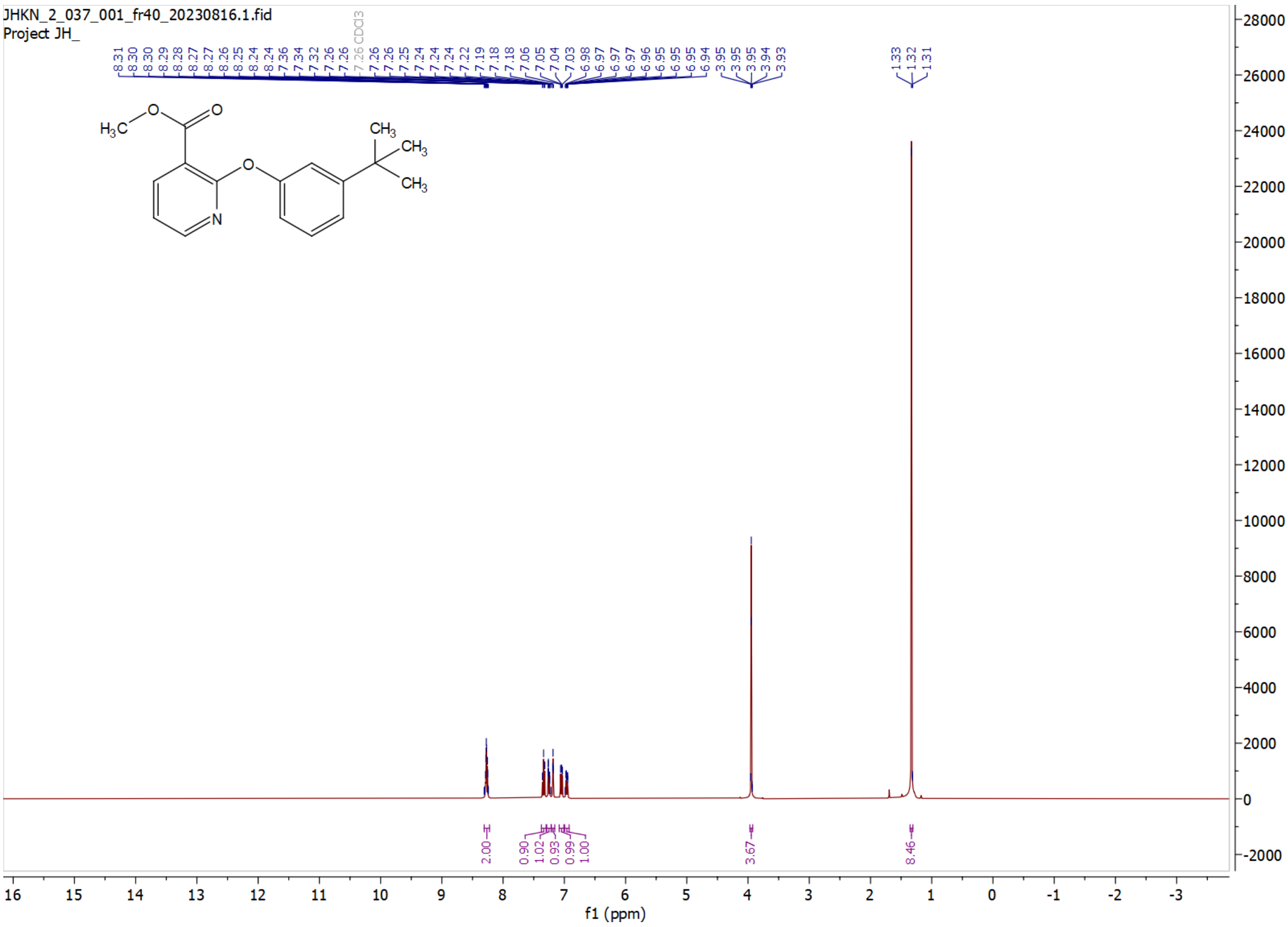
JHKN_2_037_001_fr40_20230816.1.fid
Project JH_
8.31
8.30
8.30
8.29
8.28
8.27
8.27
8.26
8.25
8.24
8.24
7.36
7.34
7.32
7.26
7.26 CDCl3
7.26
7.26
7.25
7.24
7.24
7.24
7.24
7.22
7.19
7.18
7.18
7.06
7.05
7.04
7.03
6.98
6.97
6.97
6.97
6.96
6.95
6.95
6.95
6.94
3.95
3.95
3.95
3.94
3.93
1.33
1.32
1.31
2.00
0.90
1.02
0.93
0.99
1.00
3.67
8.46
f1 (ppm)

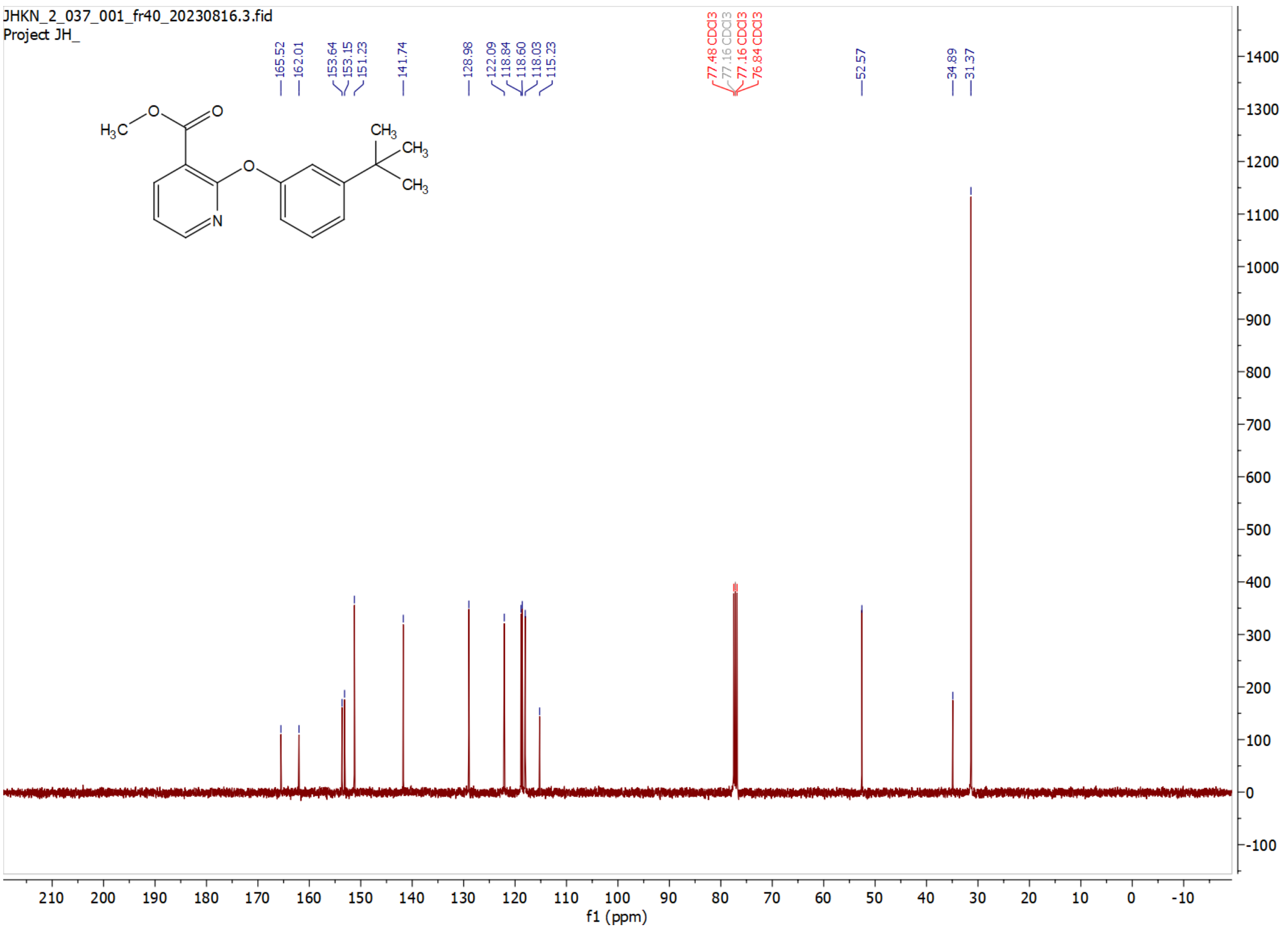
JHKN_2_037_001_fr40_20230816.3.fid
Project JH_
165.52
162.01
153.64
153.15
151.23
141.74
128.98
122.09
118.84
118.60
118.03
115.23
77.48 CDCl3
77.16 CDCl3
77.16 CDCl3
76.84 CDCl3
52.57
34.89
31.37
f1 (ppm)

D:\Data\Karoline\LCMS-2022-06-03\JHKN_2_037_001_fr13-19_ESI.raw 8/9/2023 11:44:03 AM

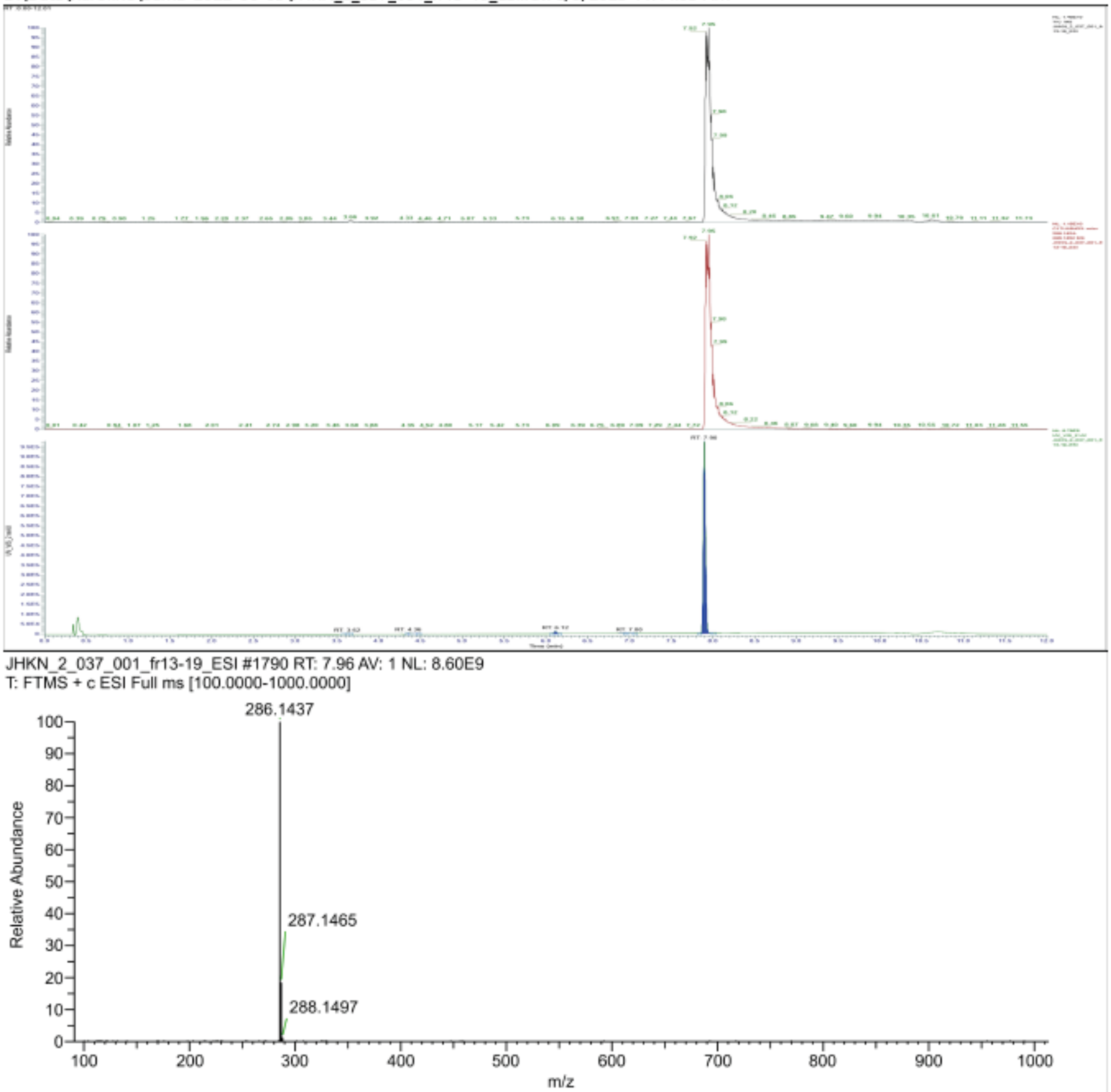

Index	% Area	RT (Mi...	RT (Sec)	Start RT	End RT	Base P...	Peak...	Peak...	Baseli...	Signal...	FileNa...	Scan F...	TraceId	% Hei...	Integr...	Name
1	0.18	3.62...	217....	3.57...	3.66...	0	3182...	1321...	0.08...	9.60...	D:\D...	No F...	Trace3	0.13	Man...	
2	0.66	4.35...	261....	4.33...	4.47...	0	1129...	3572...	0.14...	2.59	D:\D...	No F...	Trace3	0.36	Man...	
3	1.29	6.11...	367....	6.06...	6.16...	0	2211...	1214...	0.09...	8.85	D:\D...	No F...	Trace3	1.22	Man...	
4	0.3	6.99...	419....	6.95...	7.06...	0	5227...	1382...	0.10...	1.00	D:\D...	No F...	Trace3	0.14	Man...	
5	97.57	7.89...	473....	7.83...	8.00...	0	1679...	9758...	0.16...	7.10E2	D:\D...	No F...	Trace3	98.15	Man...	

File Location: C:\Program Files (x86)\Agilent\MicroLab PC\Results\JHKN_2_037_001_fr40_2023-08-17T12-32-57.a2r

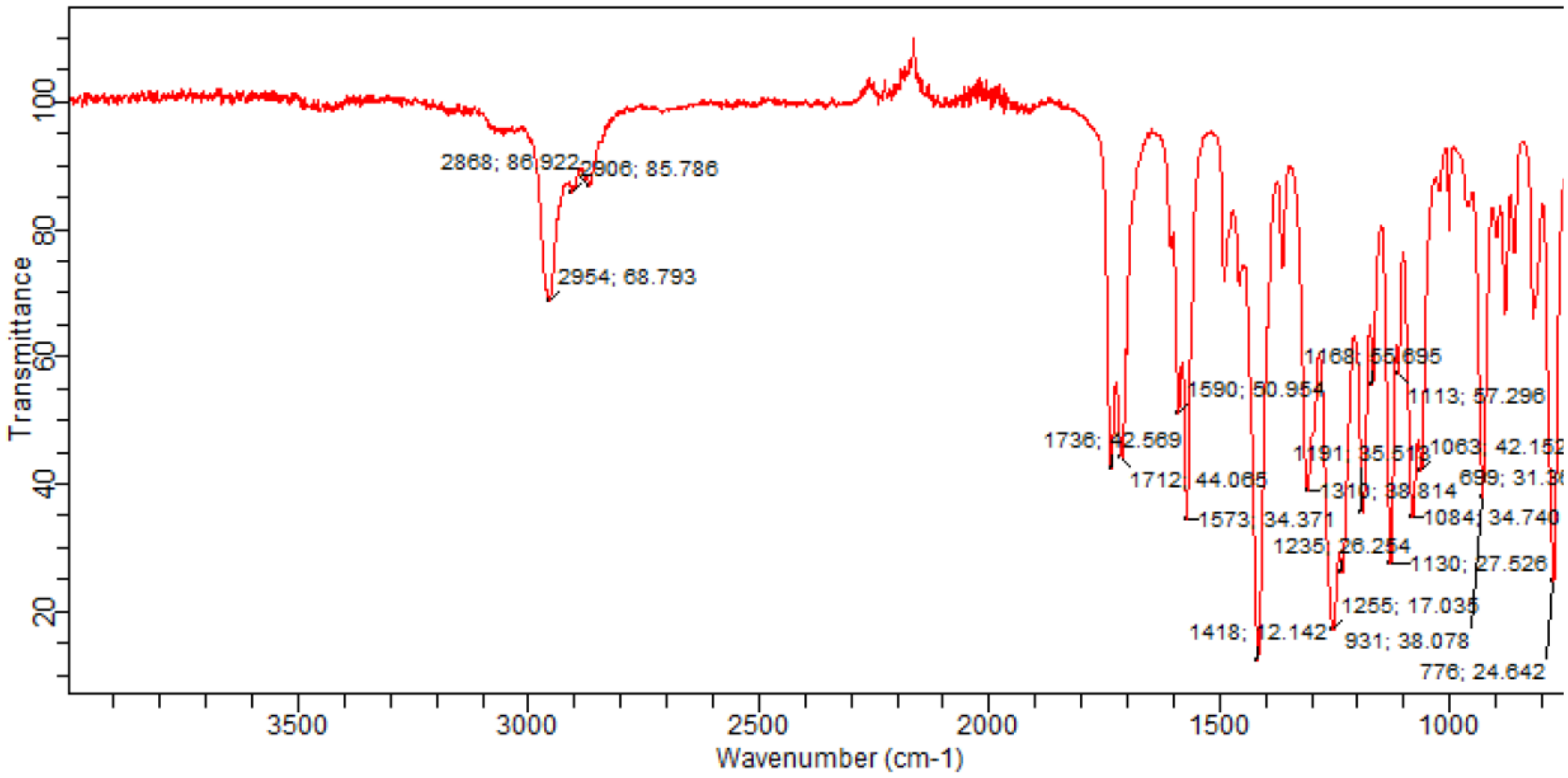

2a

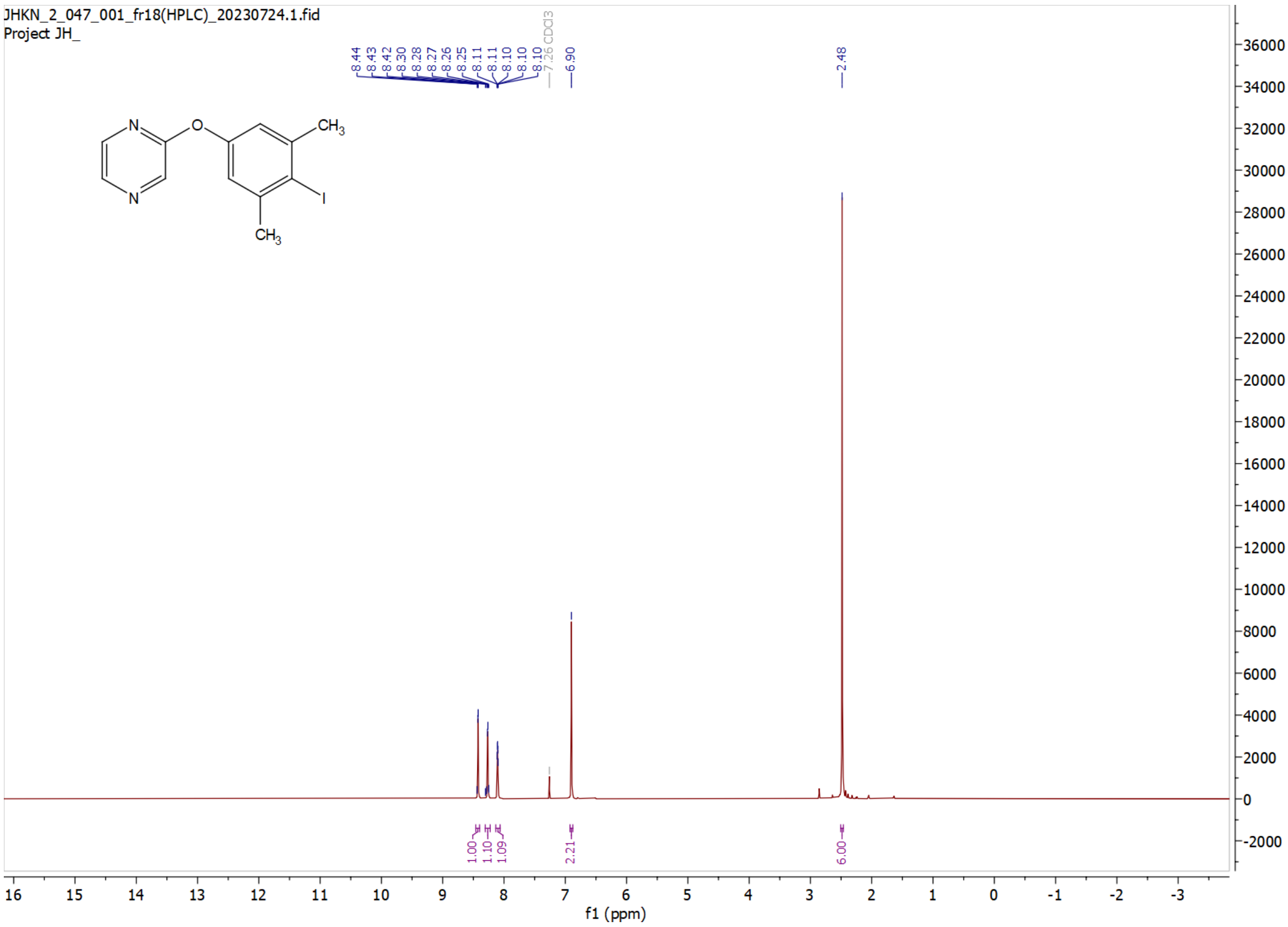
JHKN_2_047_001_fr18(HPLC)_20230724.1.fid
Project JH_
8.44
8.43
8.42
8.30
8.28
8.27
8.26
8.25
8.11
8.11
8.10
8.10
8.10
7.26 CDCl3
6.90
2.48
1.00
1.10
1.09
2.21
6.00
f1 (ppm)

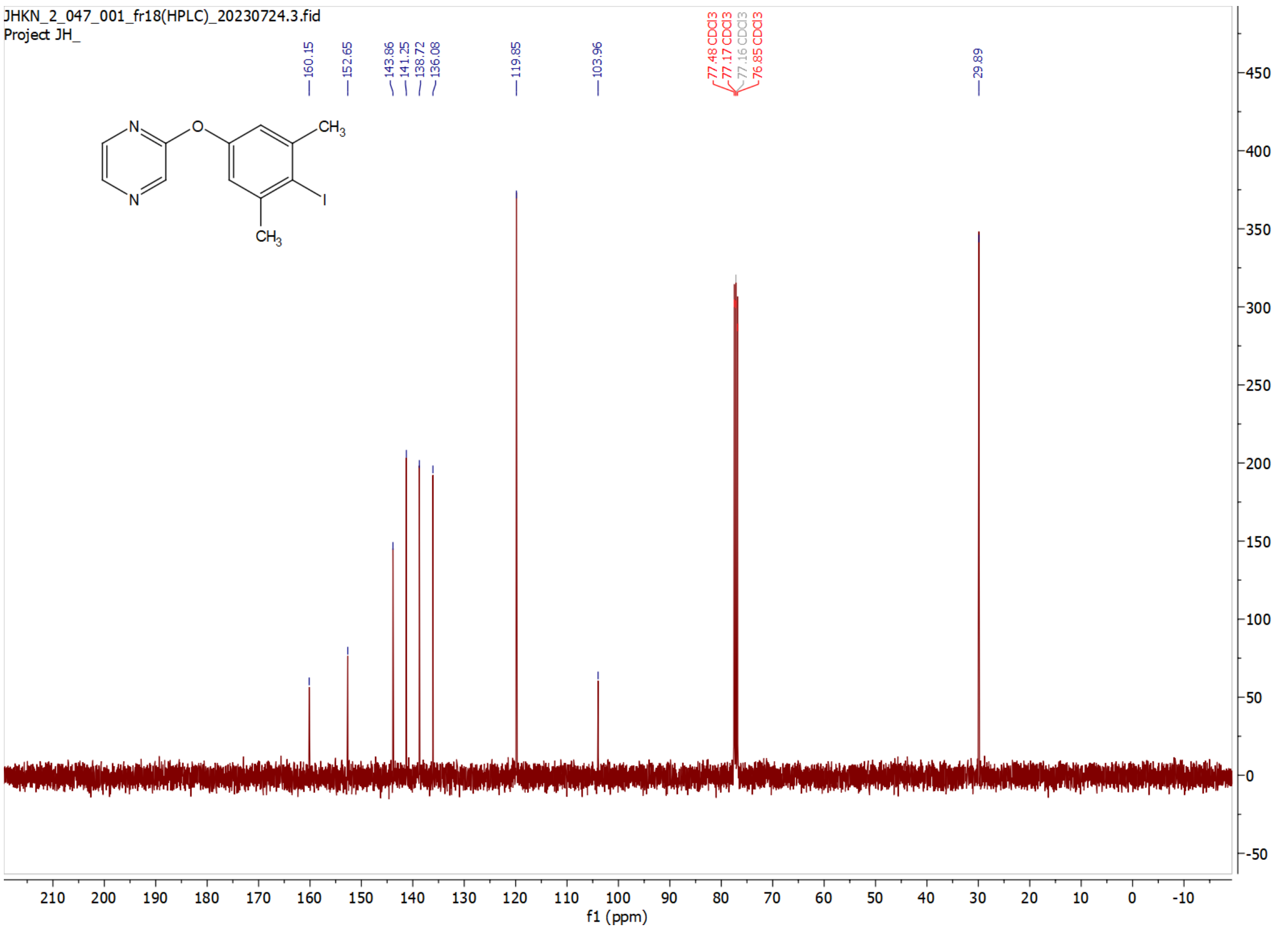
JHKN_2_047_001_fr18(HPLC)_20230724.3.fid
Project JH_
160.15
152.65
143.86
141.25
138.72
136.08
119.85
103.96
77.48 CDCl3
77.17 CDCl3
77.16 CDCl3
76.85 CDCl3
29.89
f1 (ppm)

D:\Data\Karoline\LCMS-2022-06-03\JHKN_2_047_001_fr18_ESI.raw   8/7/2023 1:15:44 PM

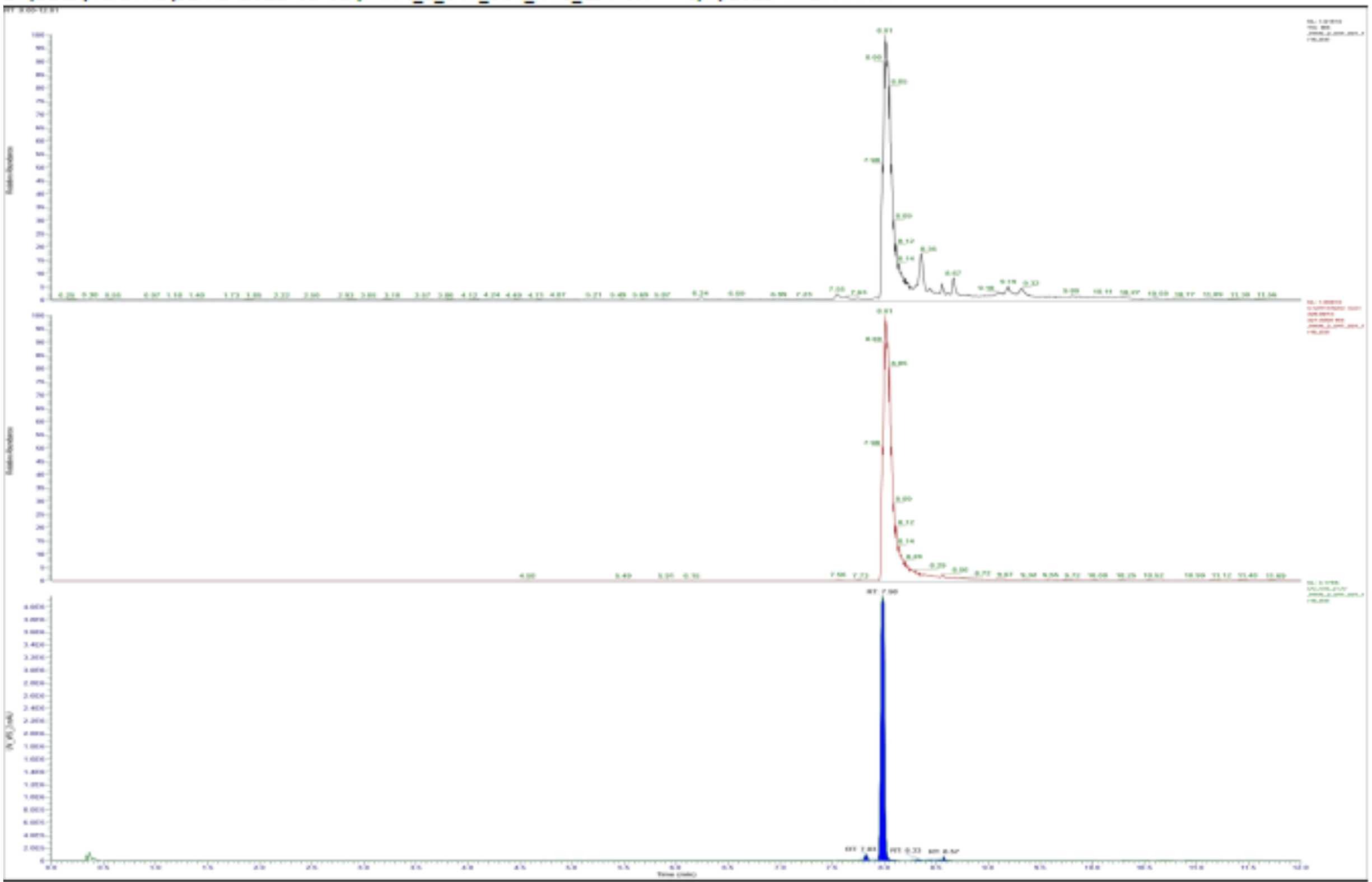

JHKN_2_047_001_fr18_ESI #1800 RT: 8.01 AV: 1 NL: 1.60E10
T: FTMS + c ESI Full ms [100.0000-1000.0000]

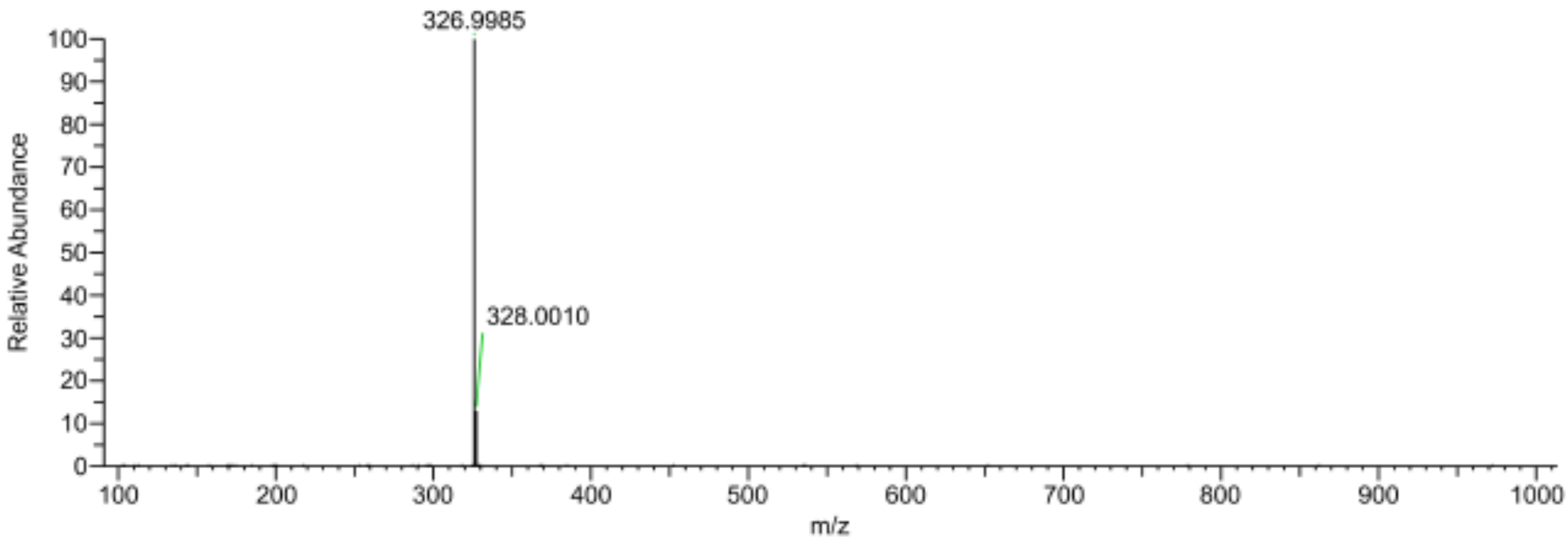

Index	% Area	RT (Mi...	RT (Sec)	Start RT	End RT	Base P...	Peak...	Peak...	Baseli...	Signal...	FileNa...	Scan F...	TraceId	% Hei...	Integr...	Name
1	1.81	7.82...	469....	7.78...	7.89...	0	2056...	1000...	0.10...	7.41E1	D:\D...	No F...	Trace3	2.29	Man...	
2	96.79	7.98...	478....	7.93...	8.11...	0	1097...	4162...	0.17...	3.10E3	D:\D...	No F...	Trace3	95.43	Man...	
3	0.39	8.33...	499....	8.28...	8.36...	0	4386...	2222...	0.07...	1.65E1	D:\D...	No F...	Trace3	0.51	Man...	
4	0.15	8.43...	506....	8.42...	8.47...	0	1719...	1401...	0.05...	1.04E1	D:\D...	No F...	Trace3	0.32	Man...	
5	0.86	8.57...	514....	8.51...	8.61...	0	9696...	6308...	0.09...	4.68E1	D:\D...	No F...	Trace3	1.45	Man...	

File Location: C:\Program Files (x86)\Agilent\MicroLab PC\Results\JHKN_2_047_001_fr18_2023-08-09T10-13-37.a2r

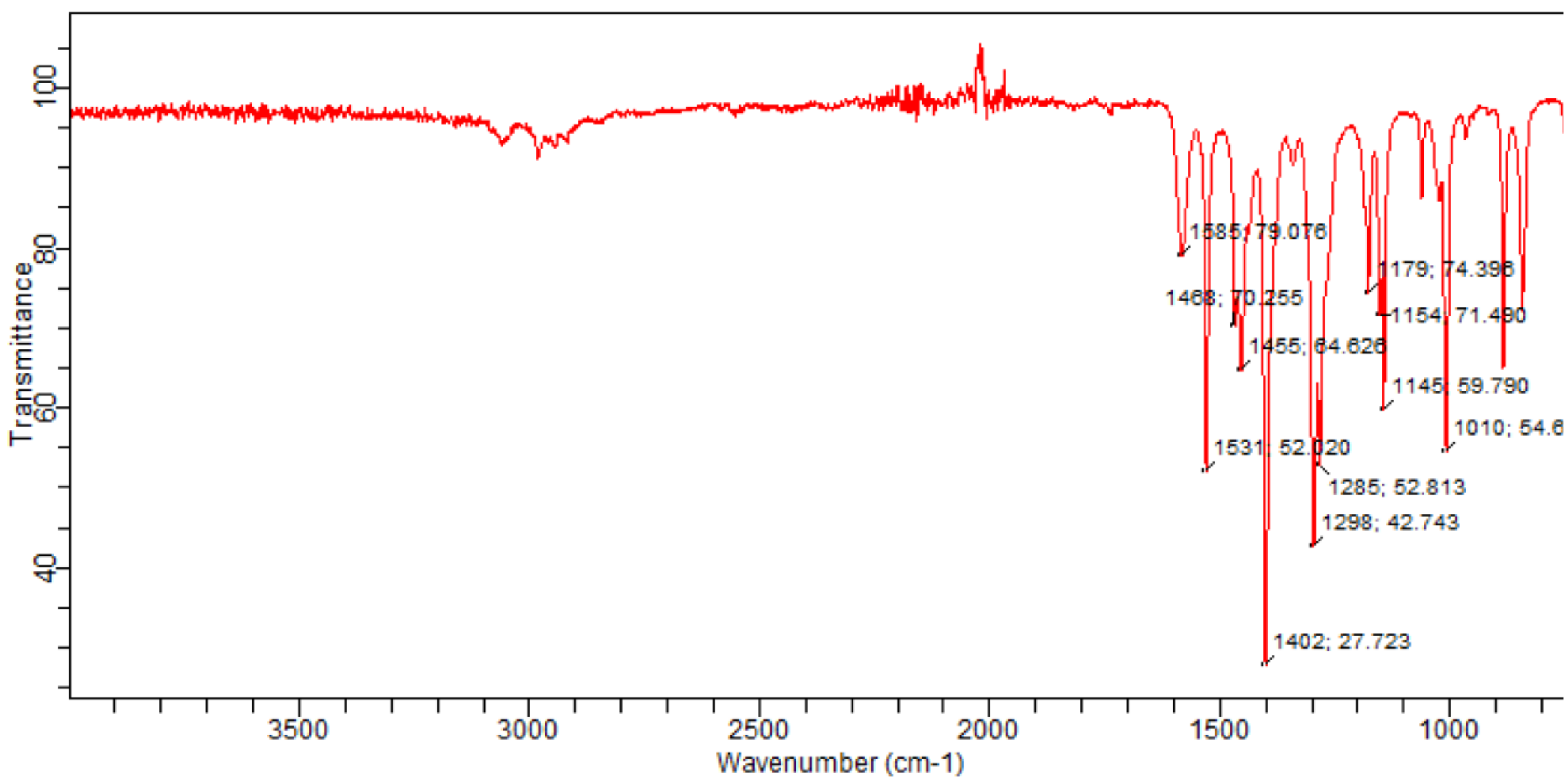

1i

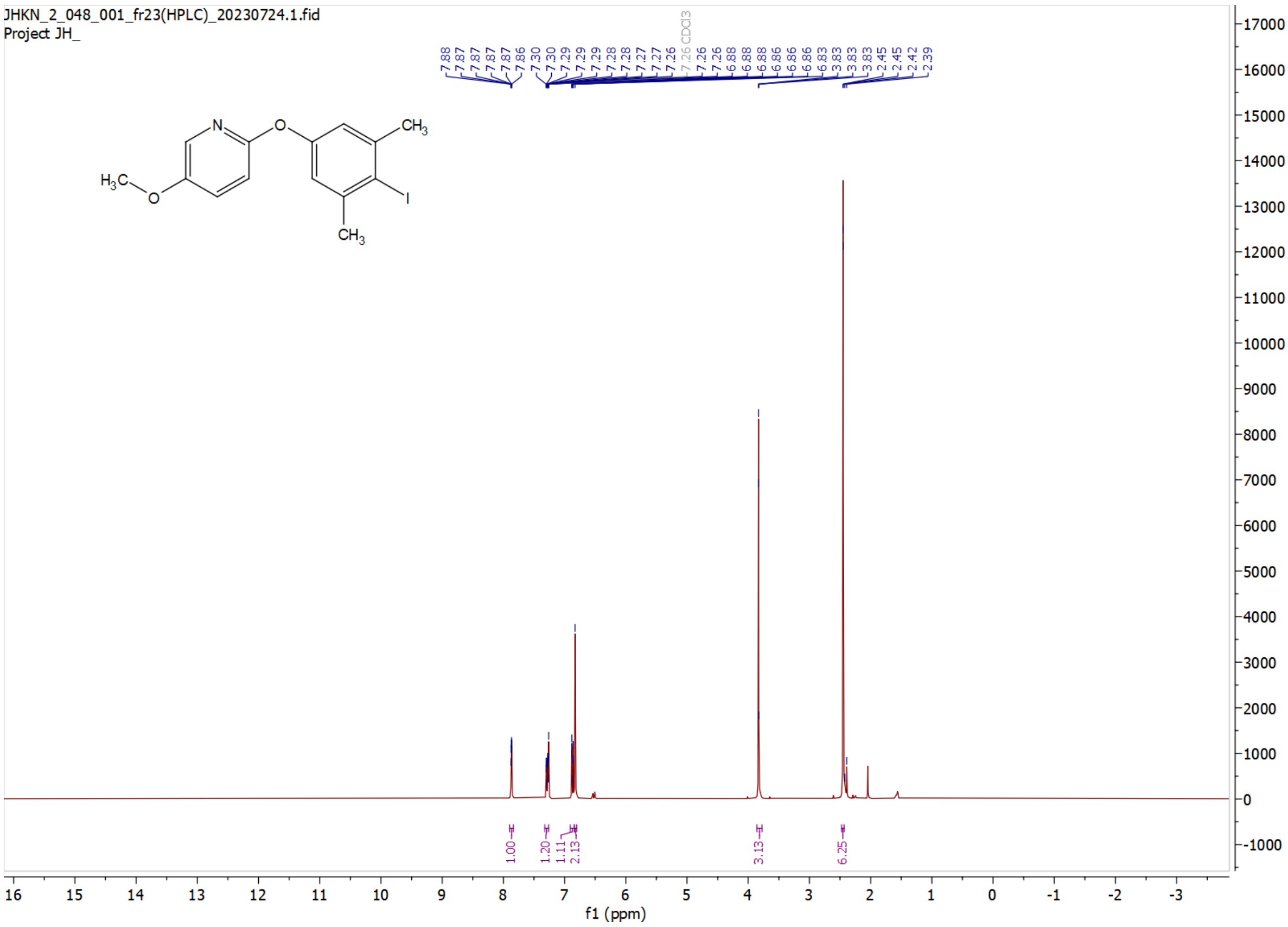
JHKN_2_048_001_fr23(HPLC)_20230724.1.fid
Project JH_
7.88
7.87
7.87
7.87
7.87
7.86
7.30
7.30
7.29
7.29
7.29
7.28
7.28
7.27
7.27
7.26
7.26 CDCl3
7.26
7.26
6.88
6.88
6.88
6.86
6.86
6.86
6.83
3.83
3.83
3.83
2.45
2.45
2.42
2.39
N
O
CH3
H3C
O
I
CH3
1.00
1.20
1.11
2.13
3.13
6.25
f1 (ppm)

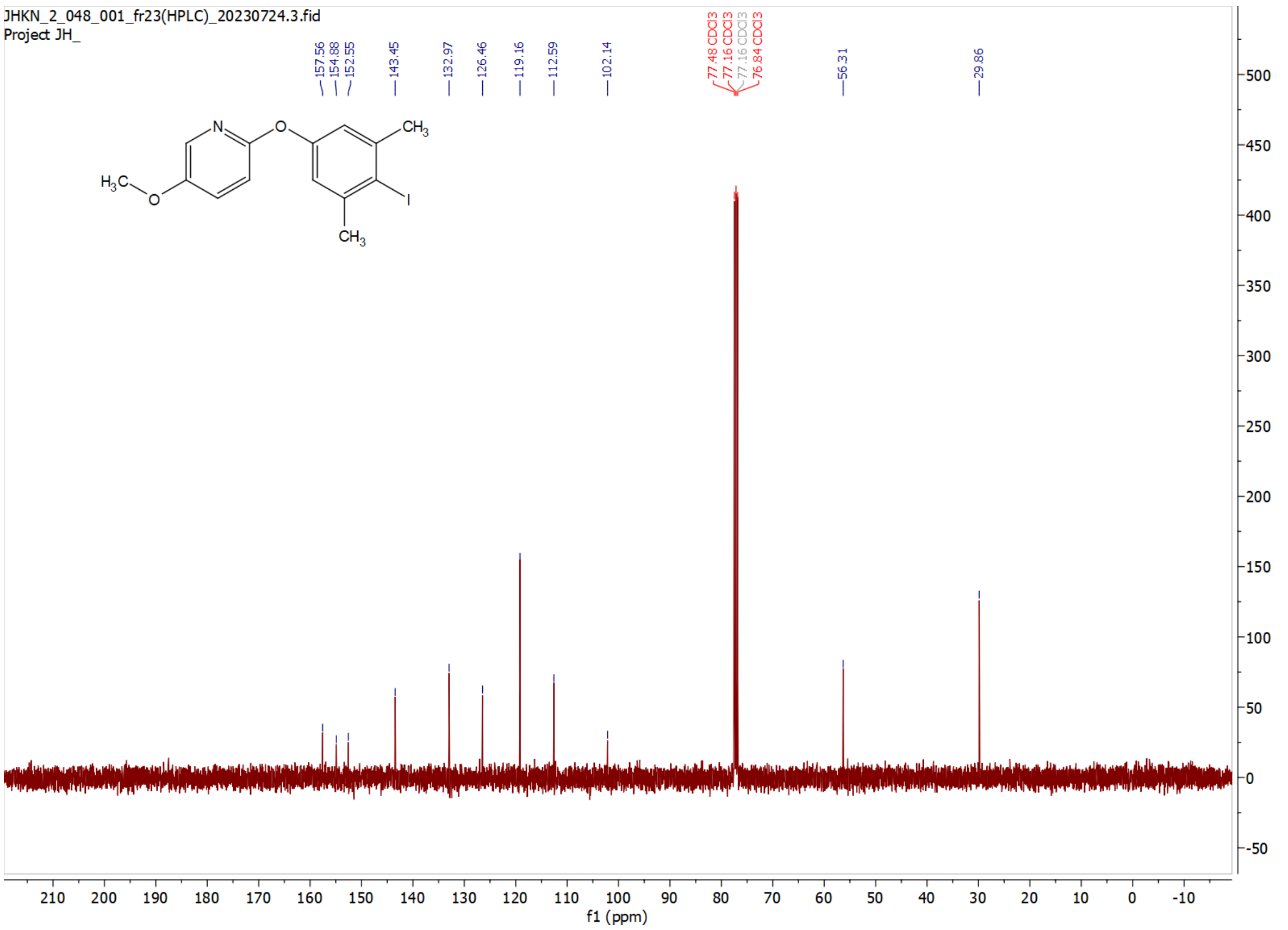
JHKN_2_048_001_fr23(HPLC)_20230724.3.fid
Project JH_
157.56
154.88
152.55
143.45
132.97
126.46
119.16
112.59
102.14
77.48 CDCl3
77.16 CDCl3
77.16 CDCl3
76.84 CDCl3
56.31
29.86
N
O
CH3
H3C
O
I
CH3
f1 (ppm)

D:\Data\Karoline\LCMS-2022-06-03\JHKN_2_048_001_fr23_ESI.raw 8/7/2023 1:20:12 PM

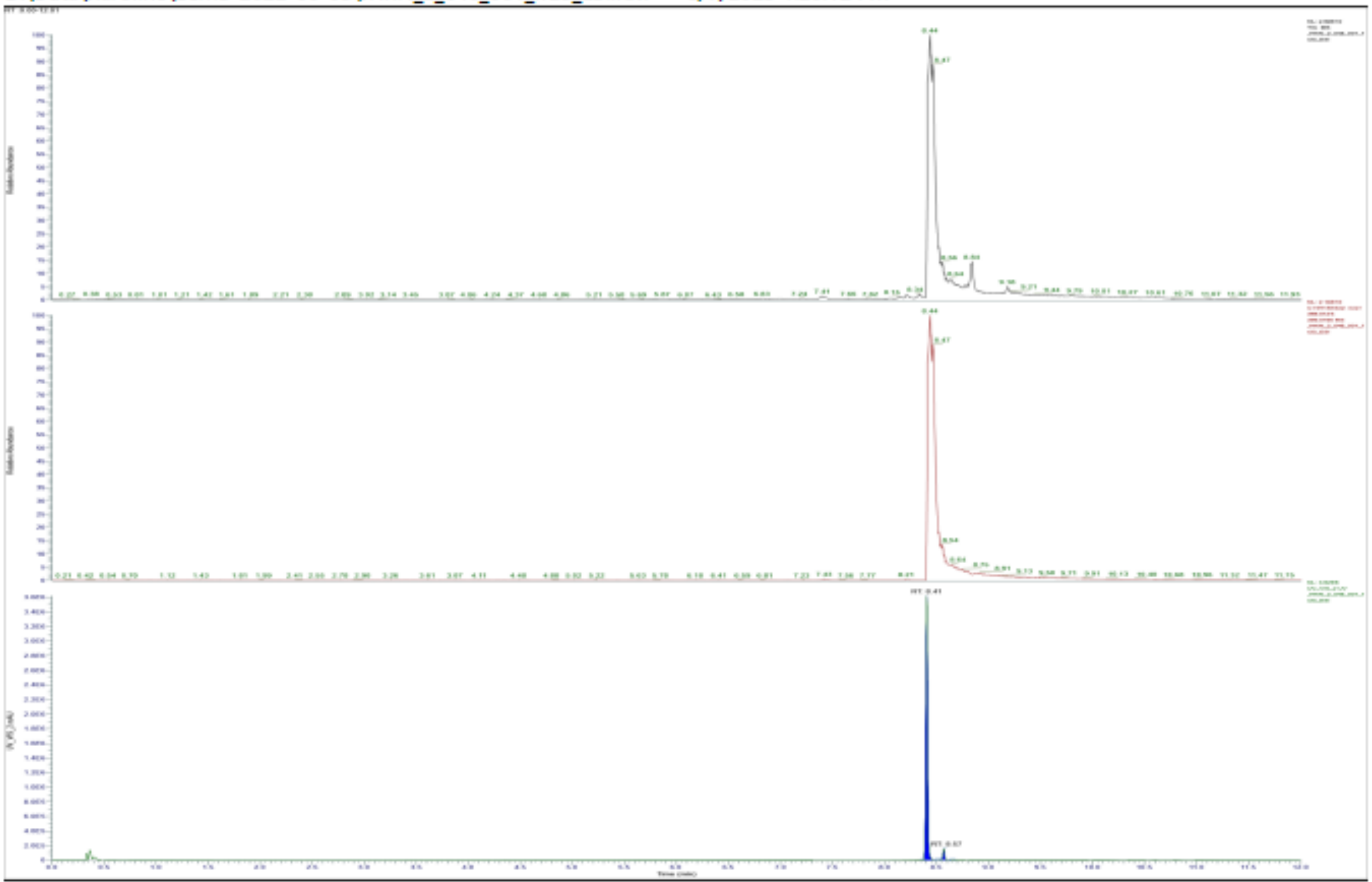

JHKN_2_048_001_fr23_ESI #1889 RT: 8.41 AV: 1 NL: 3.11E9
T: FTMS + c ESI Full ms [100.0000-1000.0000]

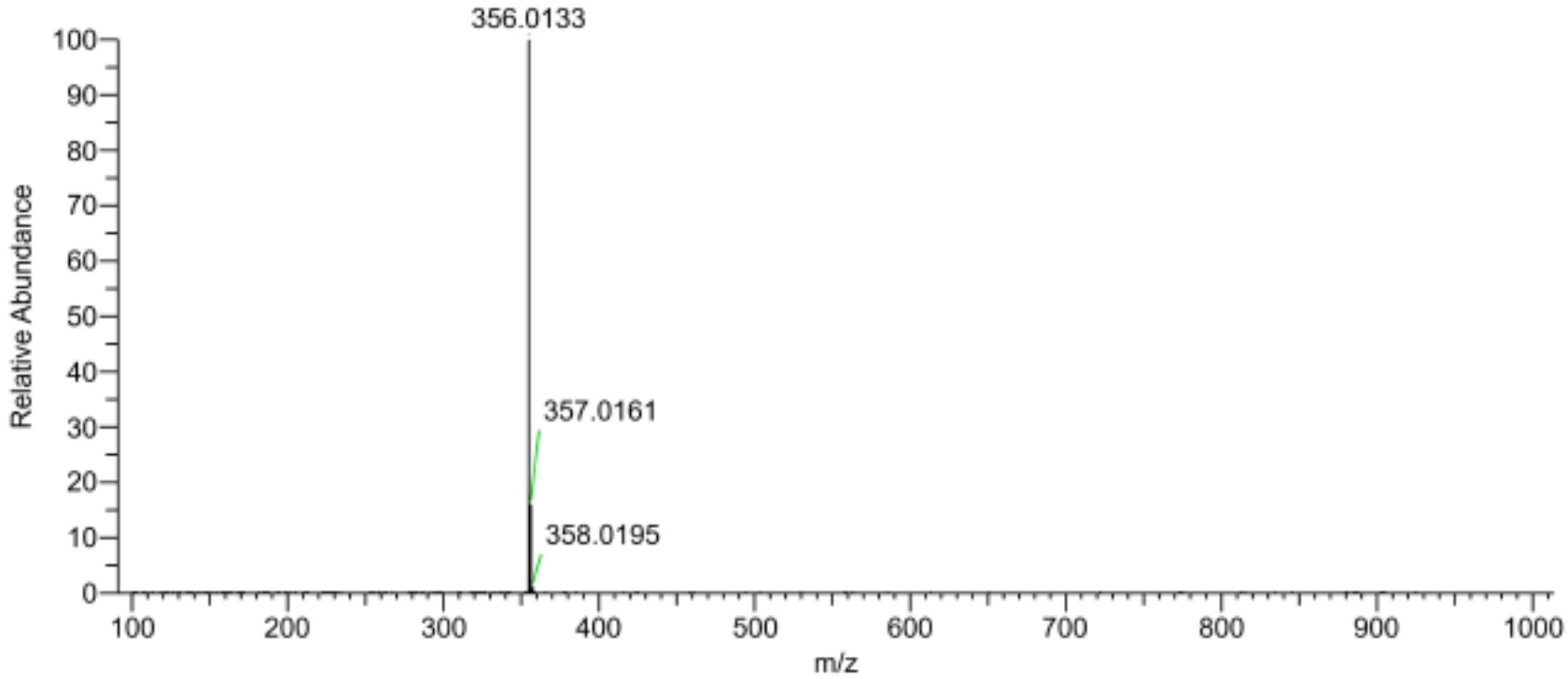

Index	% Area	RT (Mi...	RT (Sec)	Start RT	End RT	Base P...	Peak...	Peak...	Baseli...	Signal...	FileNa...	Scan F...	TraceId	% Hei...	Integr...	Name
1	96.5	8.40...	504....	8.36...	8.51...	0	4692...	3611...	0.14...	2.76E3	D:\D...	No F...	Trace3	95.99	Man...	
2	3.5	8.57...	514....	8.51...	8.65...	0	1703...	1508...	0.13...	1.15E2	D:\D...	No F...	Trace3	4.01	Man...	

1j

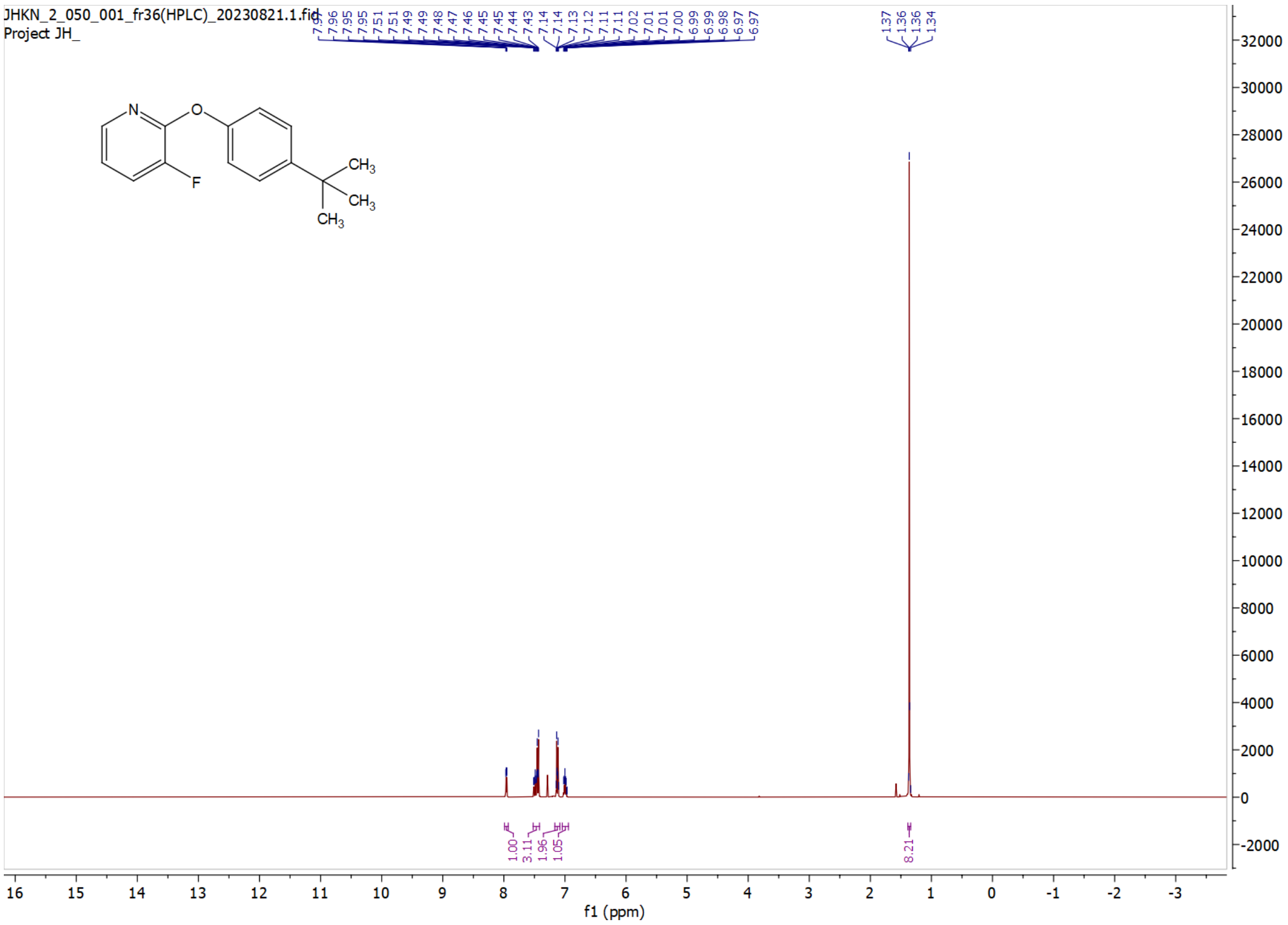
JHKN_2_050_001_fr36(HPLC)_20230821.1.fid
Project JH_
f1 (ppm)

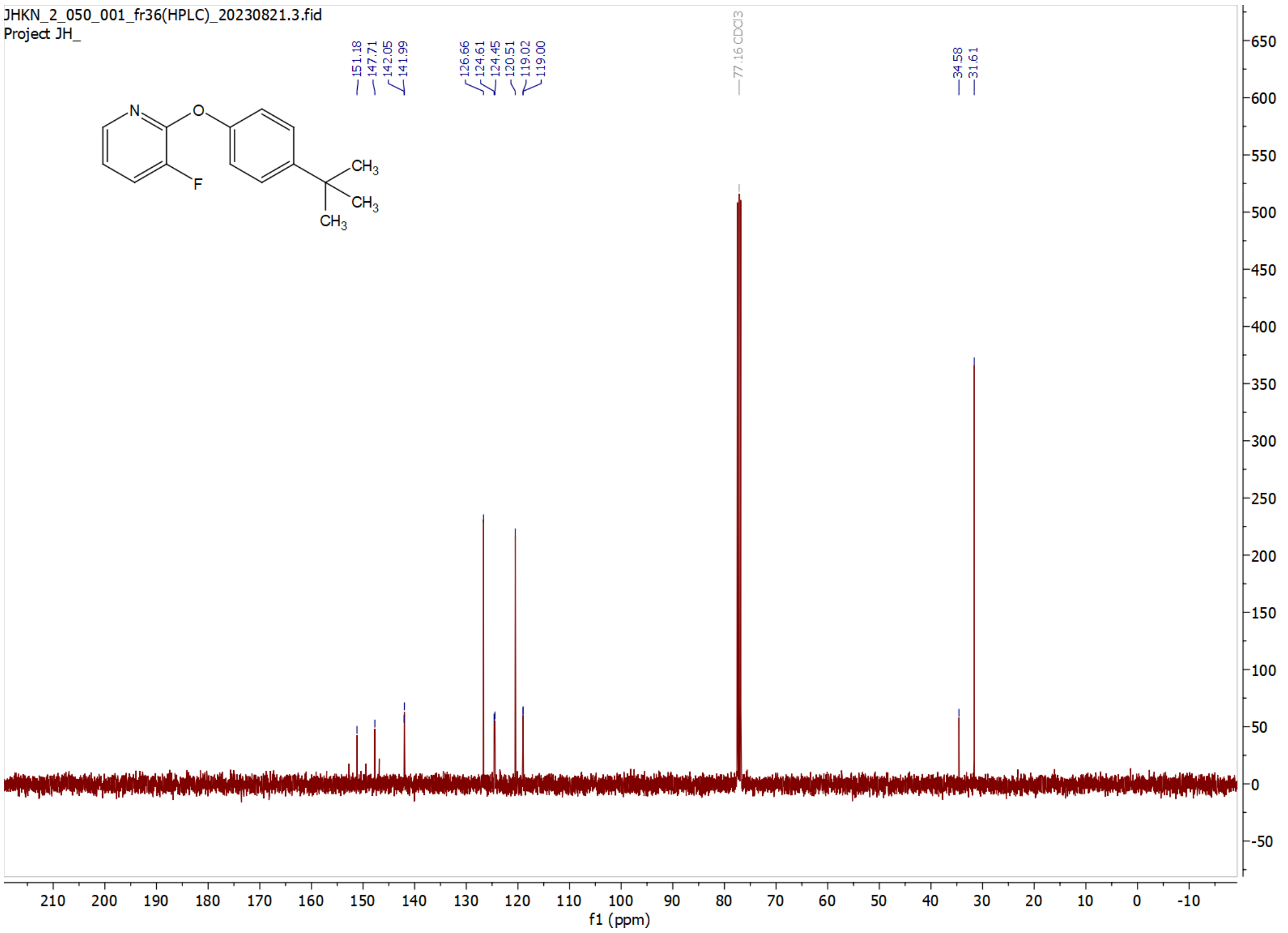
JHKN_2_050_001_fr36(HPLC)_20230821.3.fid
Project JH_
151.18
147.71
142.05
141.99
126.66
124.61
124.45
120.51
119.02
119.00
77.16 CDCl3
34.58
31.61
f1 (ppm)

D:\Data\Karoline\LCMS-2022-06-03\JHKN_2_050_001_fr36(HPLC)_ES...8/22/2023 9:45:34 AM

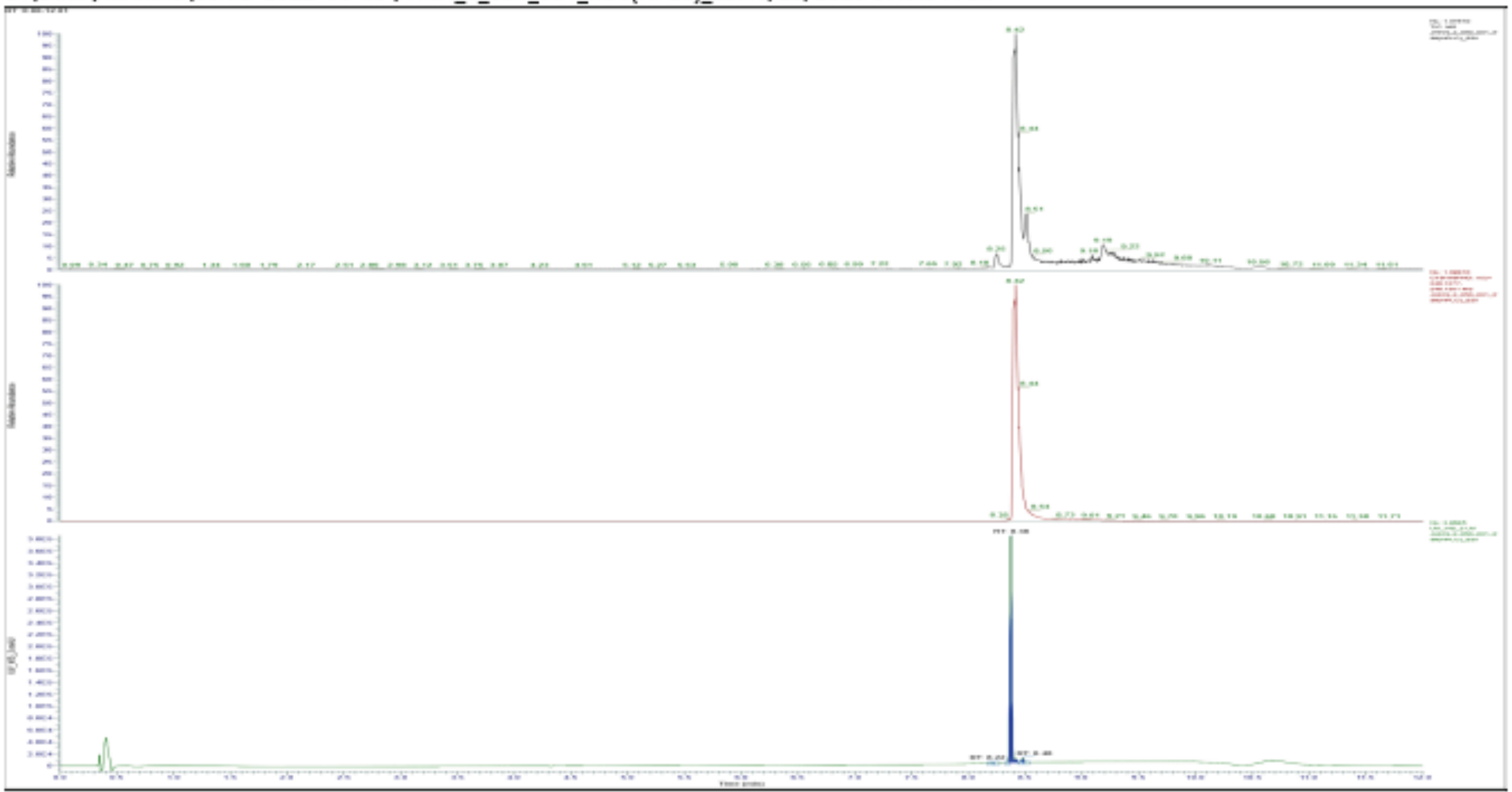

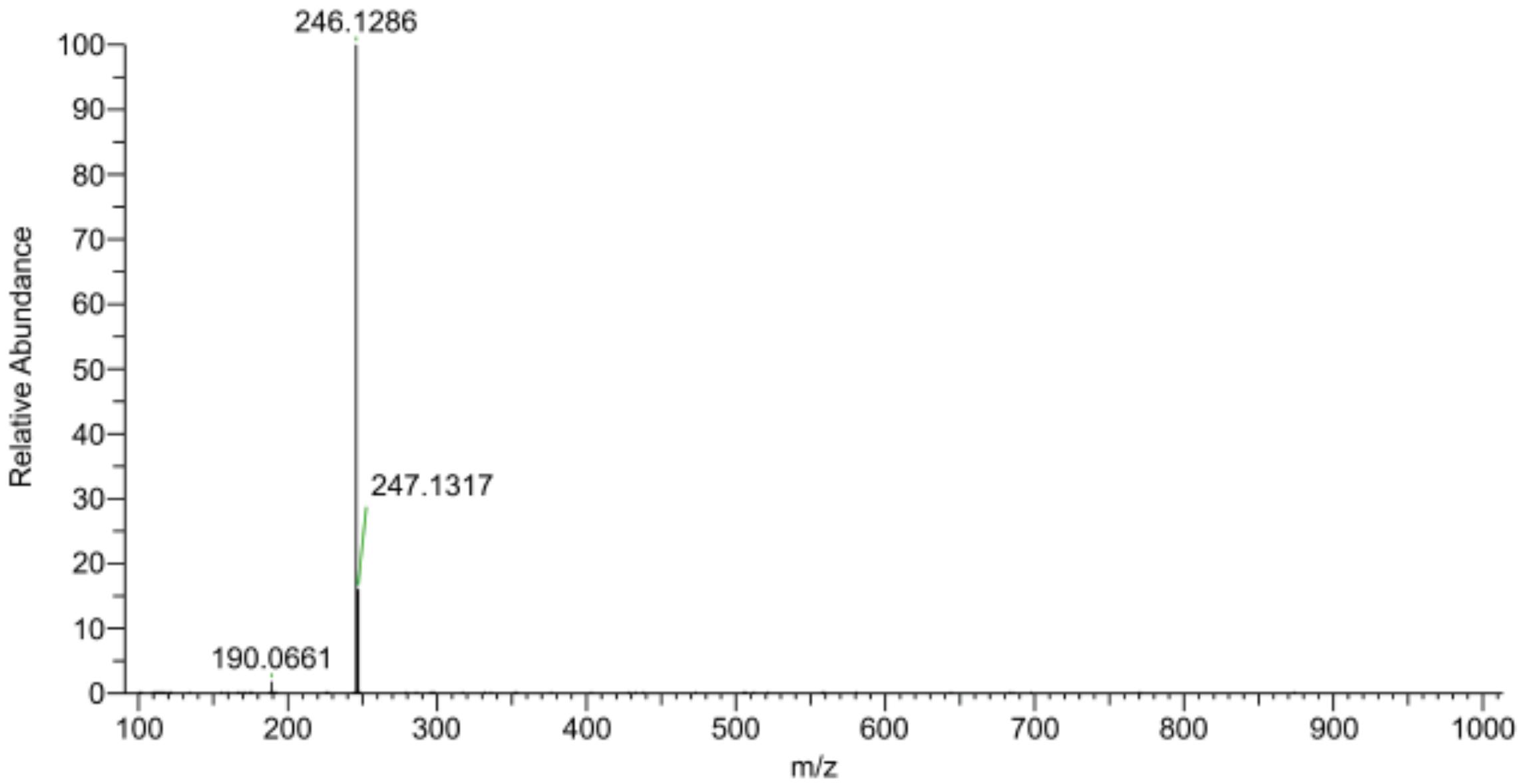

Index	% Area	RT (Mi...	RT (Sec)	Start RT	End RT	Base P...	Peak...	Peak...	Baseli...	Signal...	FileNa...	Scan F...	TraceId	% Hei...	Integr...	Name
1	0.39	8.21...	493....	8.18...	8.24...	0	1716...	1408...	0.05...	1.07	D:\D...	No F...	Trace3	0.36	Man...	
2	97.61	8.3767	502....	8.33...	8.45...	0	4257...	3802...	0.11...	2.88E2	D:\D...	No F...	Trace3	97.74	Man...	
3	2	8.48...	508....	8.46...	8.51...	0	8701...	7376...	0.04...	5.60	D:\D...	No F...	Trace3	1.9	Man...	

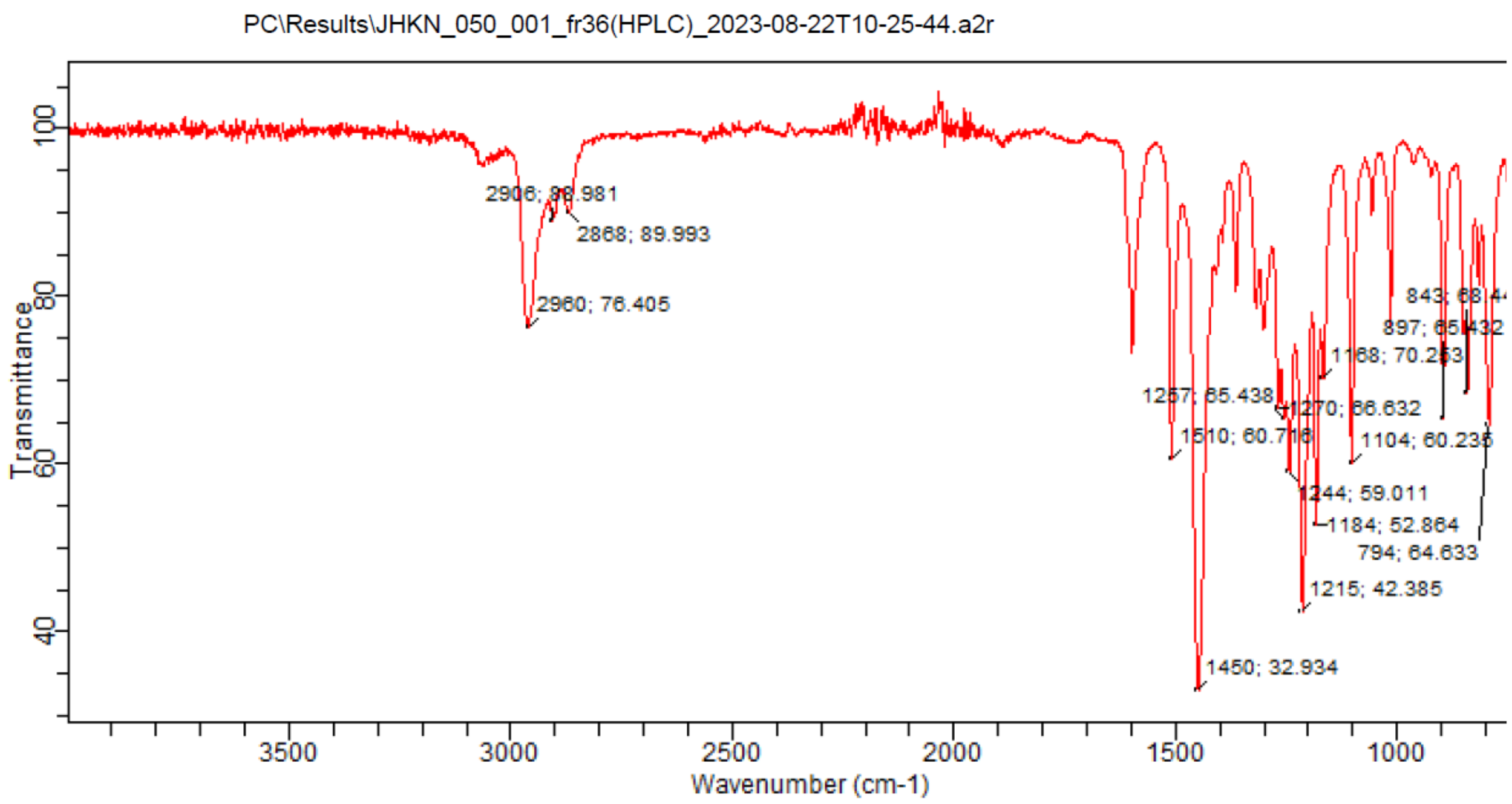

PC\Results\JHKN_050_001_fr36(HPLC)_2023-08-22T10-25-44.a2r
Transmittance
Wavenumber (cm-1)
2960; 76.405
2868; 89.993
1450; 32.934
1215; 42.385
1184; 52.864
1244; 59.011
1104; 60.235
1510; 60.716
1168; 70.253
794; 64.633

3b

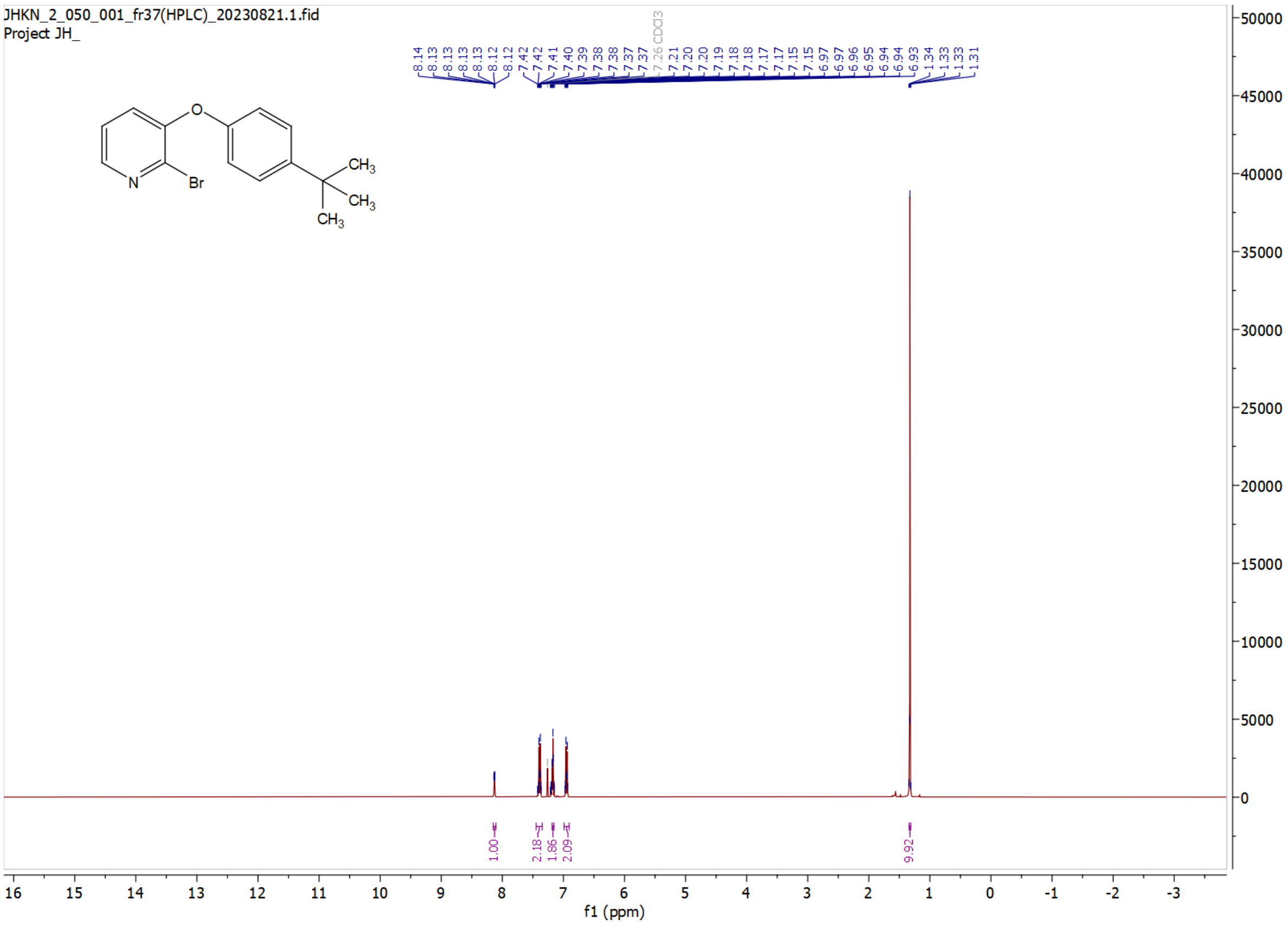
JHKN_2_050_001_fr37(HPLC)_20230821.1.fid
Project JH_
O
N
Br
CH3
CH3
CH3
8.14 8.13 8.13 8.13 8.13 8.12 8.12 7.42 7.41 7.40 7.39 7.38 7.38 7.37 7.37 7.26 CDCl3 7.21 7.20 7.20 7.19 7.18 7.18 7.17 7.17 7.15 7.15 6.97 6.97 6.96 6.95 6.94 6.94 6.93 1.34 1.33 1.33 1.31
1.00
2.18
1.86
2.09
9.92
f1 (ppm)

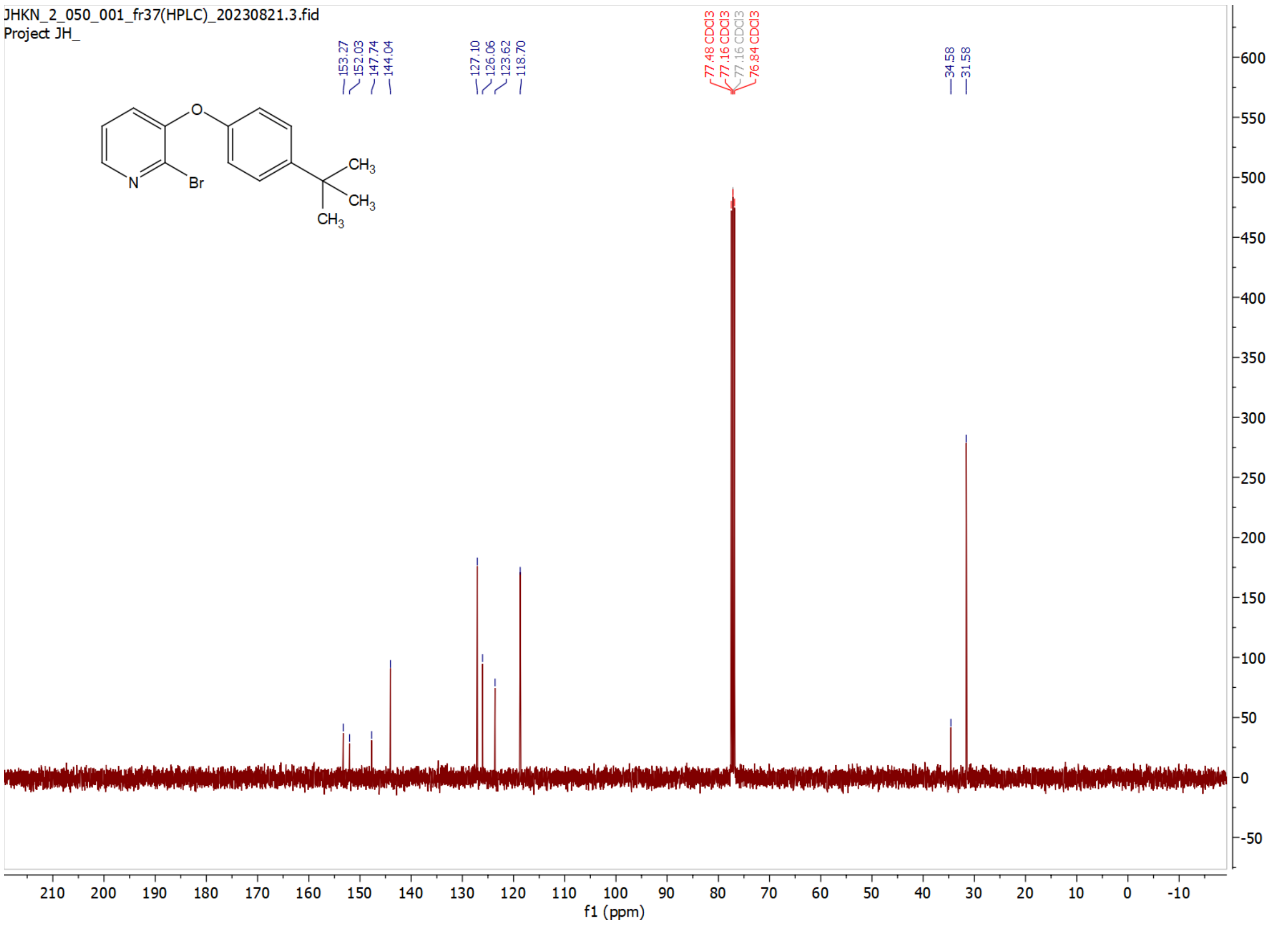
JHKN_2_050_001_fr37(HPLC)_20230821.3.fid
Project JH_
O
N
Br
CH3
CH3
CH3
153.27 152.03 147.74 144.04
127.10 126.06 123.62 118.70
77.48 CDCl3 77.16 CDCl3 77.16 CDCl3 76.84 CDCl3
34.58 31.58
f1 (ppm)

D:\Data\Karoline\LCMS-2022-06-03\JHKN_2_050_001_fr37(HPLC)_ES...8/22/2023 9:47:59 AM

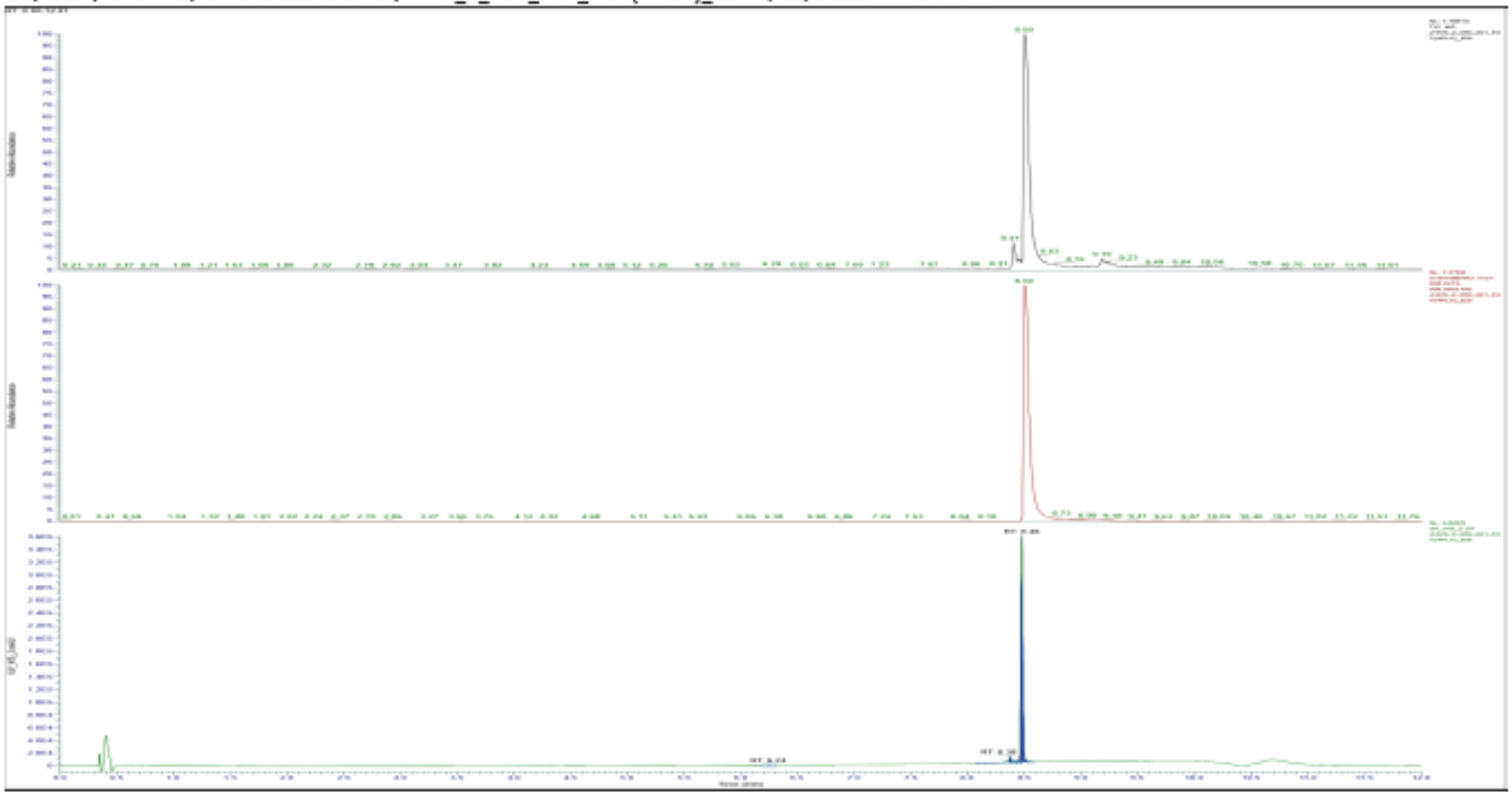

JHKN_2_050_001_fr37(HPLC)_ESI #1910 RT: 8.50 AV: 1 NL: 6.17E9
T: FTMS + c ESI Full ms [100.0000-1000.0000]

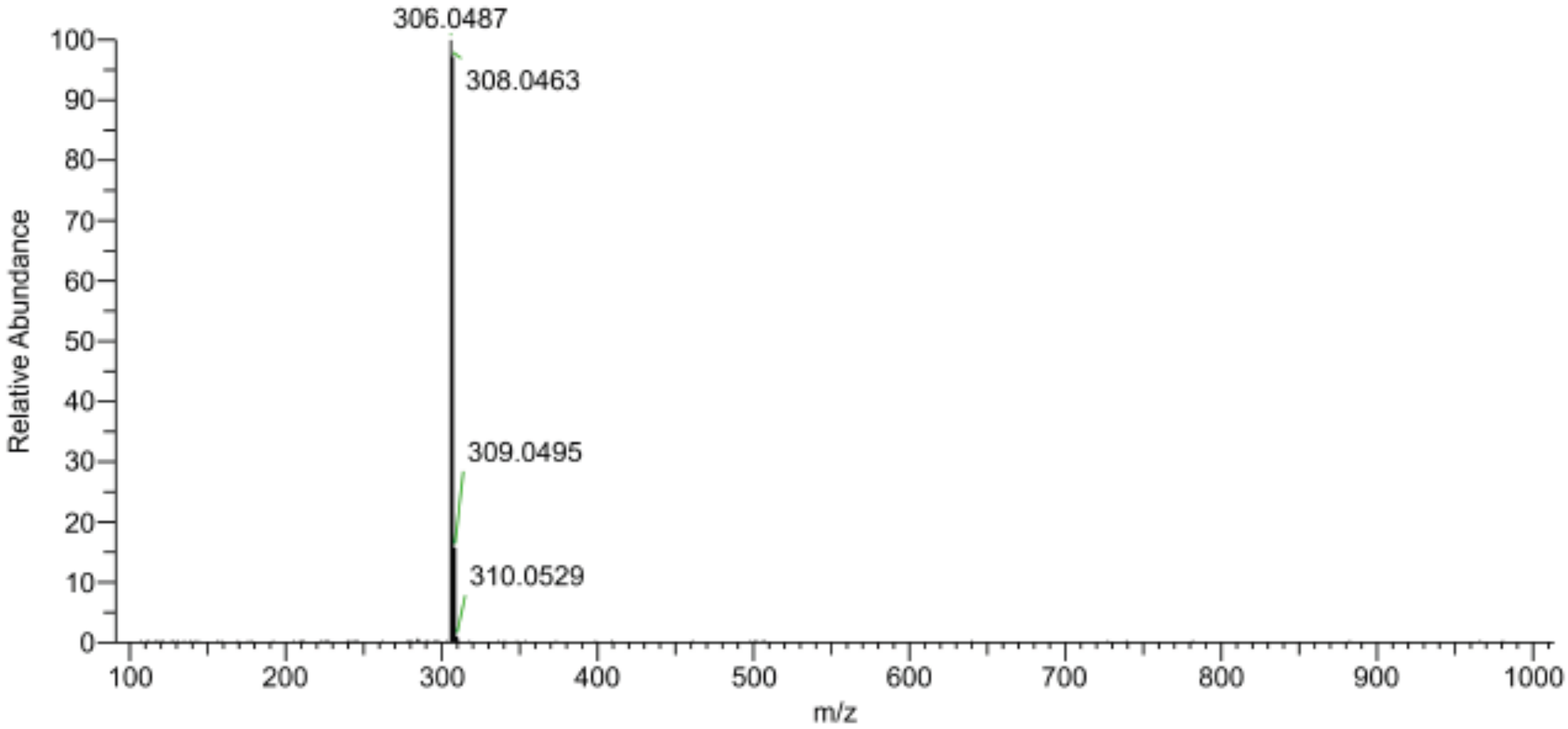

Index	% Area	RT (Mi...	RT (Sec)	Start RT	End RT	Base P...	Peak...	Peak...	Baseli...	Signal...	FileNa...	Scan F...	TraceId	% Hei...	Integr...	Name
1	0.18	6.24...	374....	6.21...	6.27...	0	726....	326....	0.06...	2.48...	D:\D...	No F...	Trace3	0.09	Man...	
2	2.42	8.3767	502....	8.34...	8.40...	0	9693...	8564...	0.06...	6.53	D:\D...	No F...	Trace3	2.34	Man...	
3	0.73	8.42...	505....	8.40...	8.44...	0	2930...	2332...	0.03...	1.77	D:\D...	No F...	Trace3	0.64	Man...	
4	96.66	8.47...	508....	8.44...	8.55...	0	3868...	3553...	0.10...	2.70E2	D:\D...	No F...	Trace3	96.94	Man...	

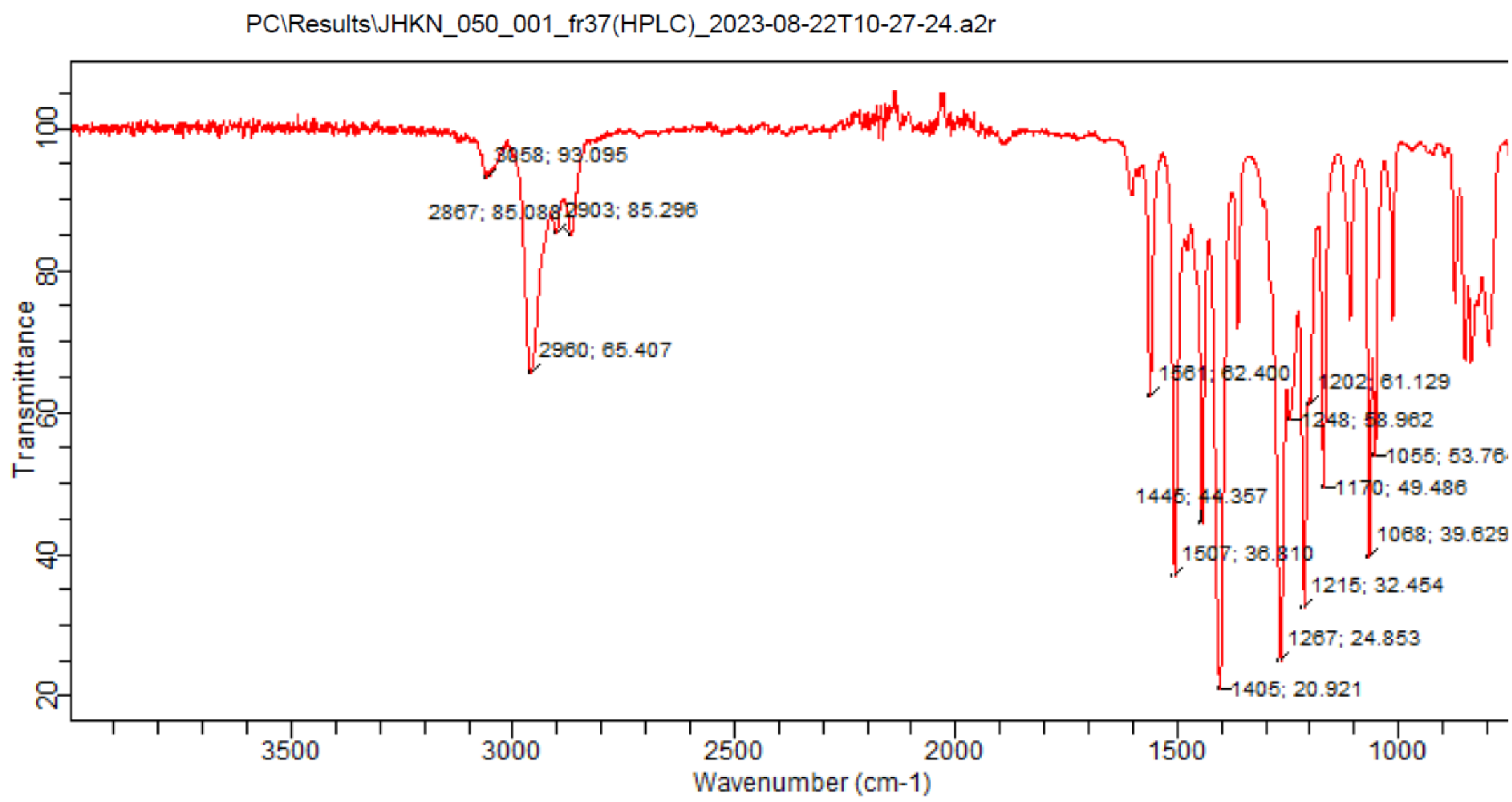

PC\Results\JHKN_050_001_fr37(HPLC)_2023-08-22T10-27-24.a2r
Transmittance
Wavenumber (cm-1)
3058; 93.095
2867; 85.088
2903; 85.296
2960; 65.407
1561; 62.400
1202; 61.129
1248; 58.962
1055; 53.764
1170; 49.486
1445; 44.357
1068; 39.629
1507; 36.810
1215; 32.454
1267; 24.853
1405; 20.921

1k

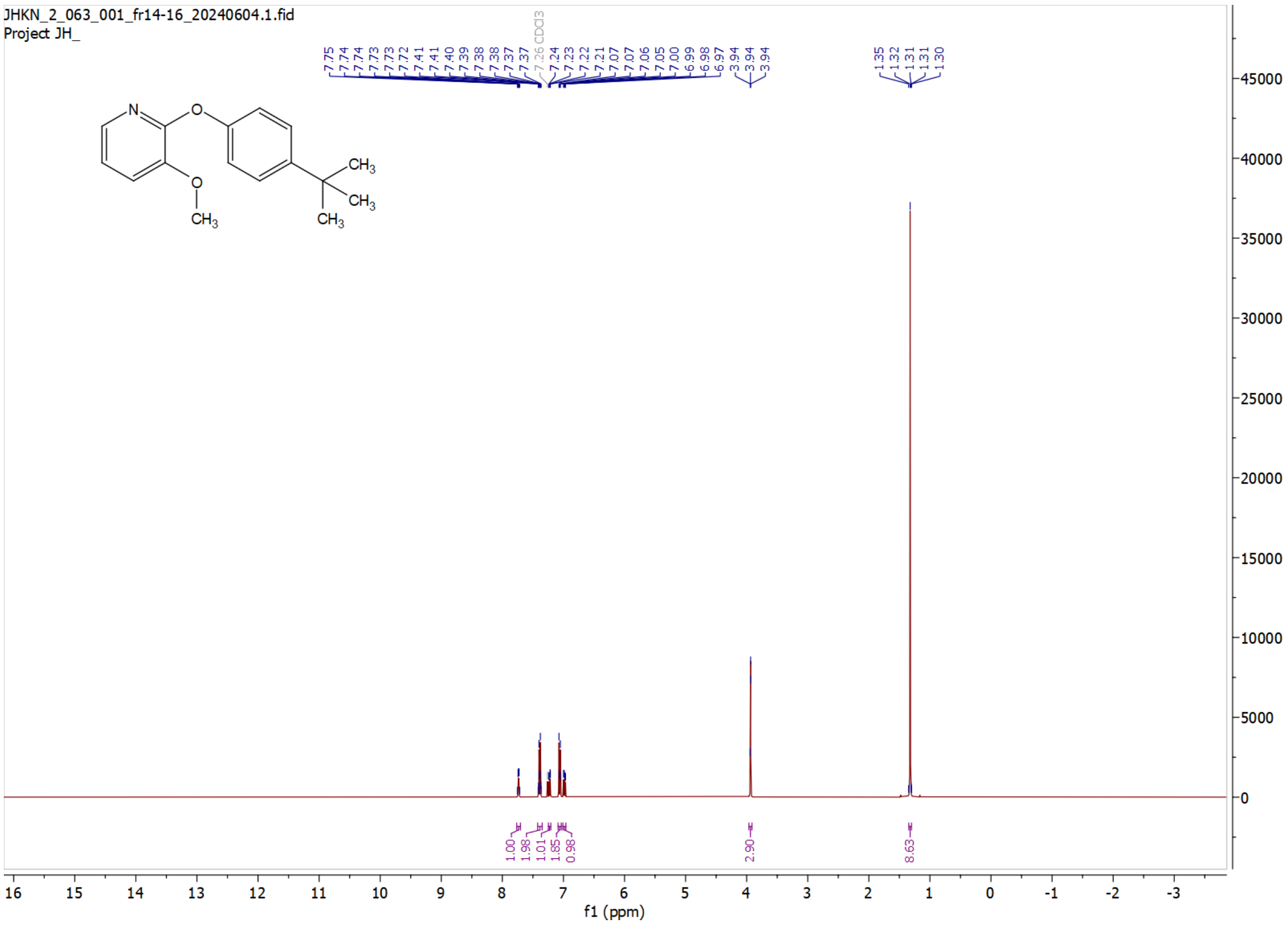
JHKN_2_063_001_fr14-16_20240604.1.fid
Project JH_
f1 (ppm)

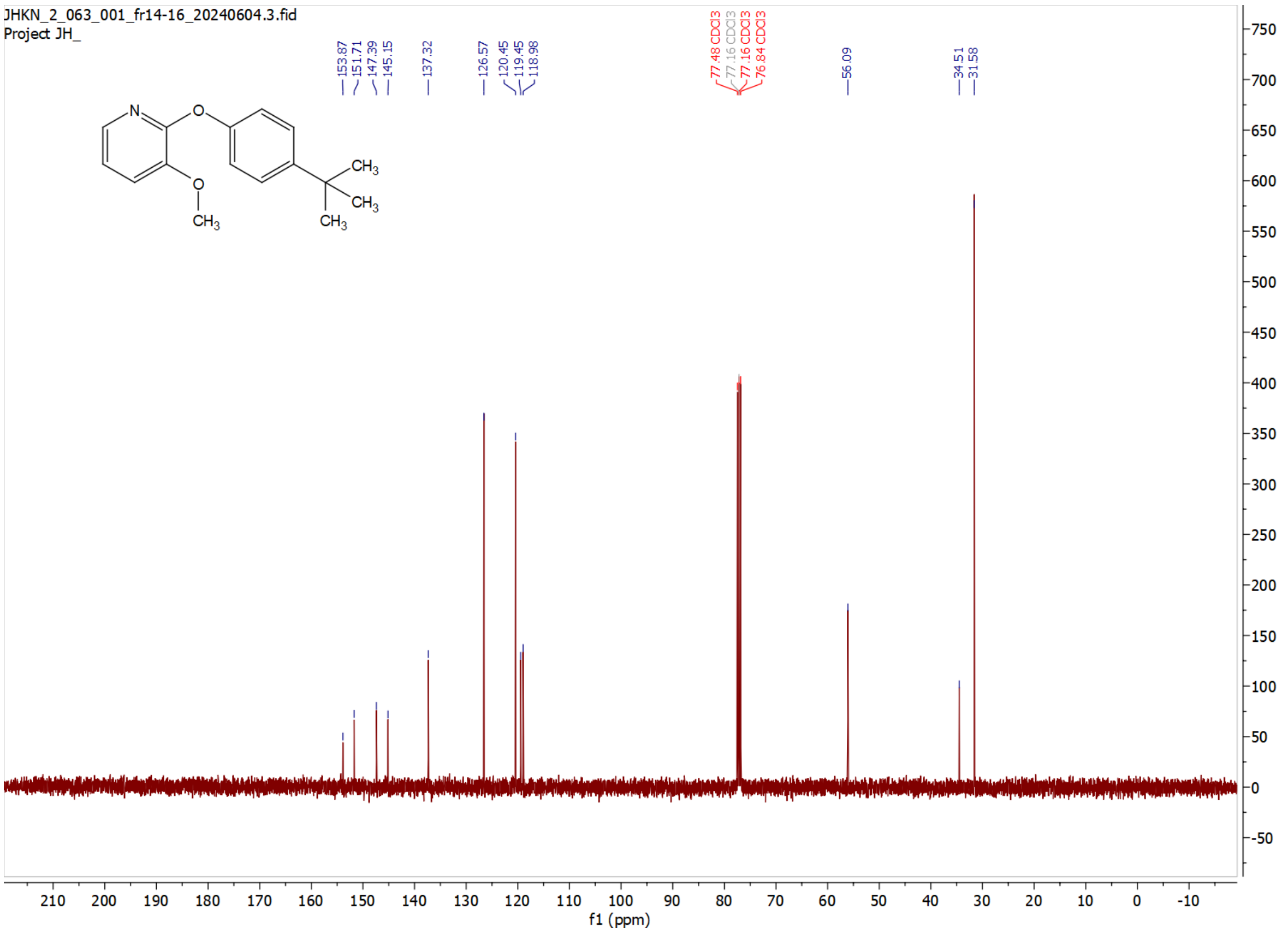
JHKN_2_063_001_fr14-16_20240604.3.fid
Project JH_
153.87
151.71
147.39
145.15
137.32
126.57
120.45
119.45
118.98
77.48 CDCl3
77.16 CDCl3
77.16 CDCl3
76.84 CDCl3
56.09
34.51
31.58
f1 (ppm)

JHKN_2_063_001_fr9-19_ESI_20240412134500 #1806 RT: 8.04 AV: 1 NL: 1.17E10
T: FTMS + c ESI Full ms [100.0000-1000.0000]

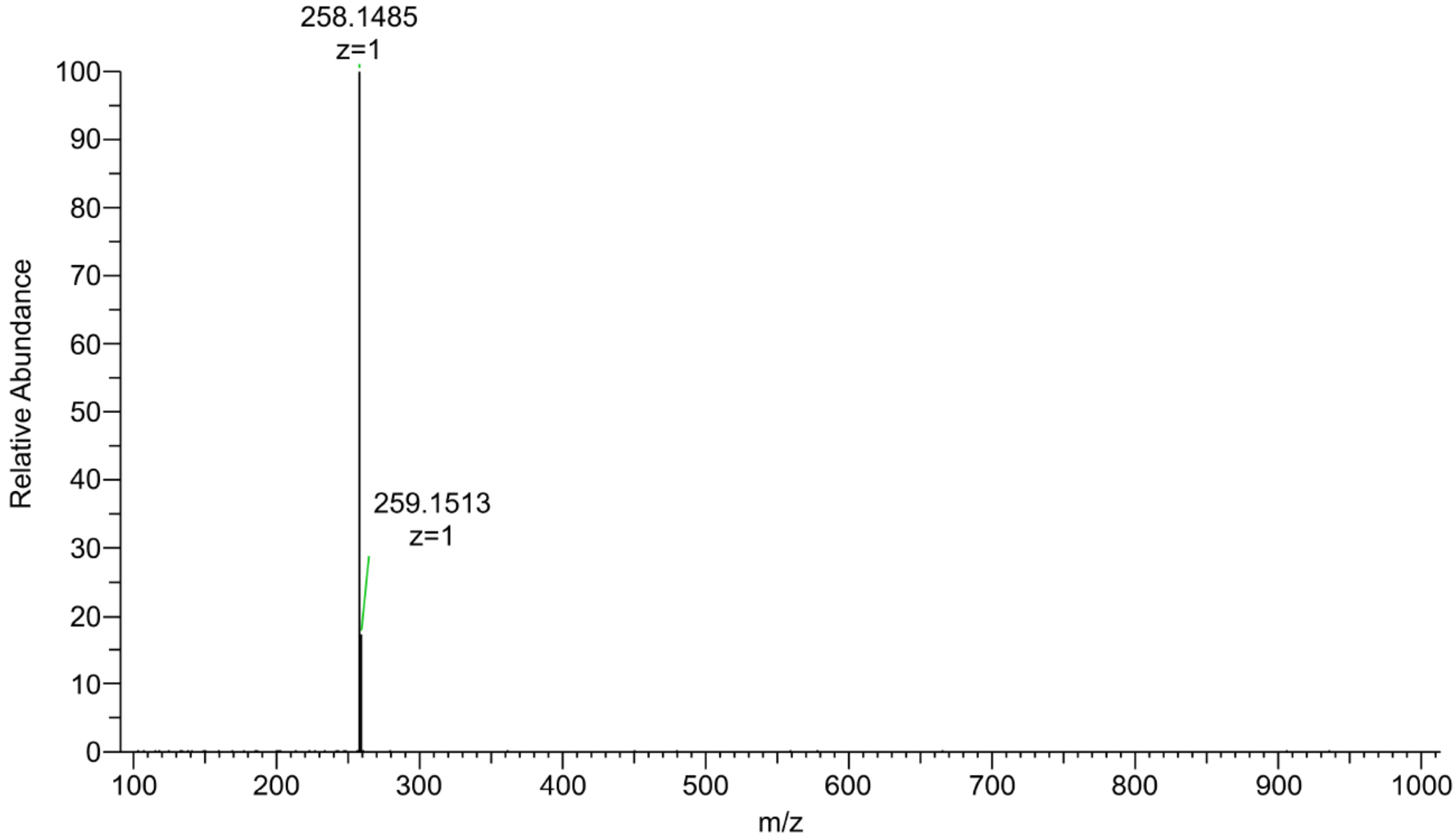

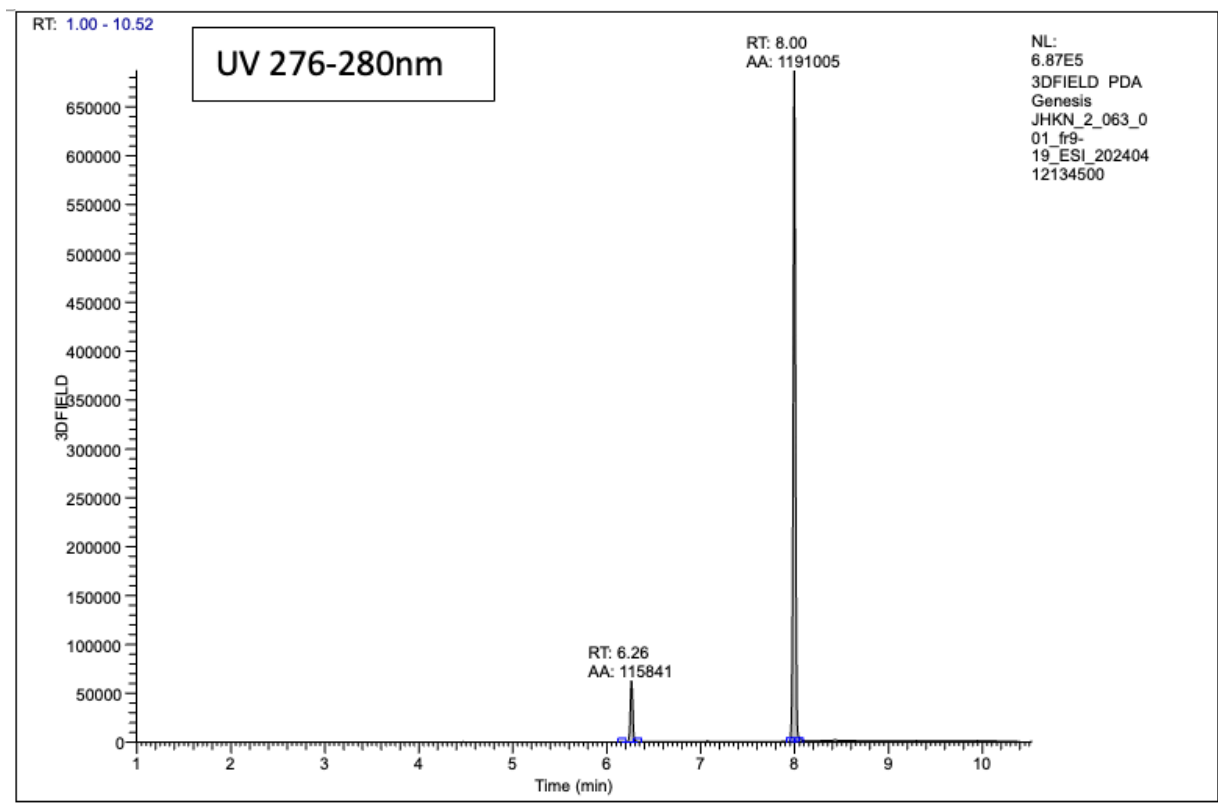

PEAK LIST
JHKN_2_063_001_fr9-19_ESI_20240412134500.raw
RT: 1.00 - 10.52
Number of detected peaks: 2

Apex RT	Start RT	End RT	Area	%Area	Height	%Height
6.26	6.16	6.33	115840.917	8.86	62507.413	8.35
8.00	7.95	8.05	1191004.650	91.14	686038.784	91.65

1l

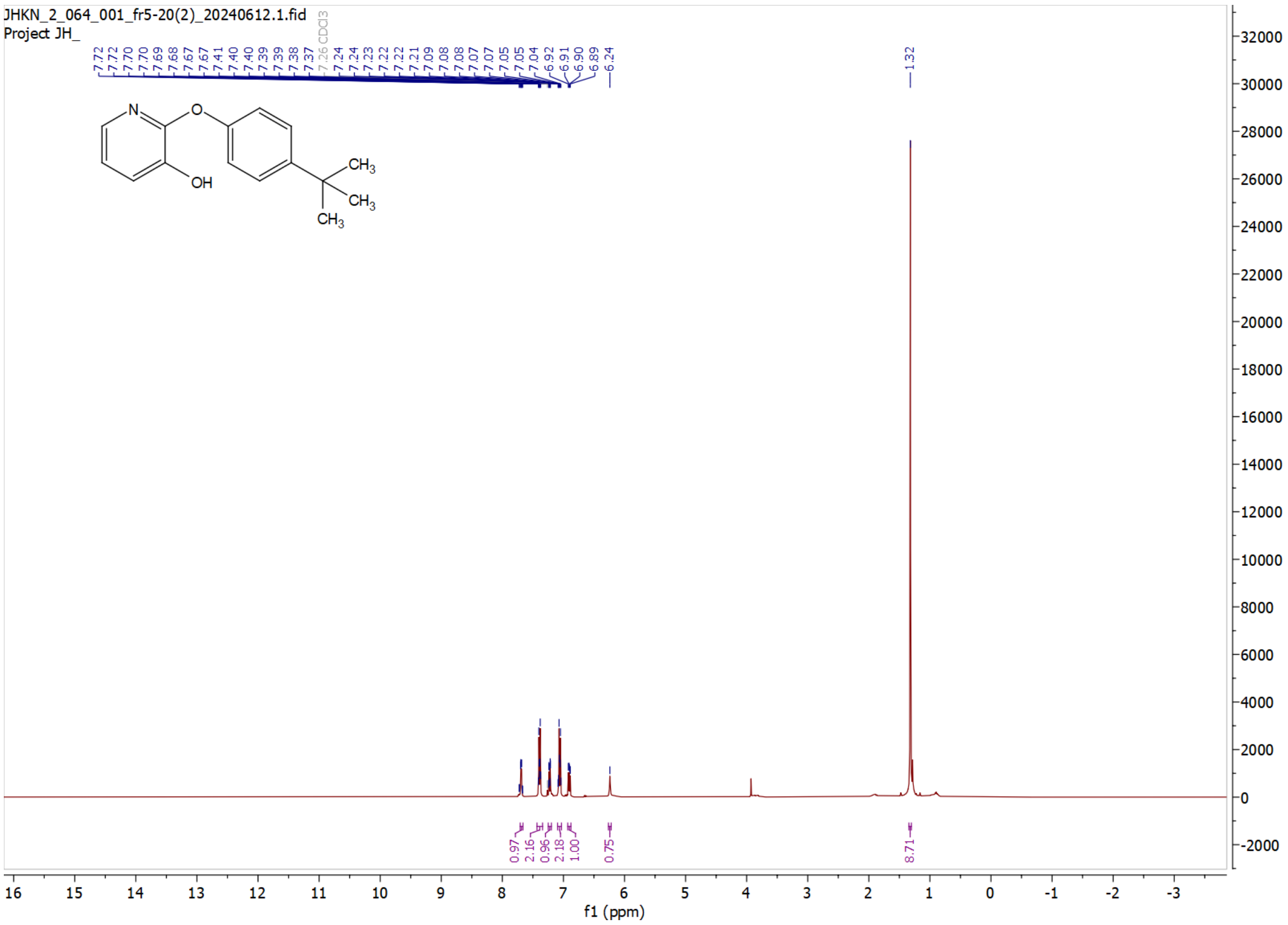

JHKN_2_064_001_fr5-20(2)_20240612.1.fid
Project JH_
1.32
0.97
2.16
0.96
2.18
1.00
0.75
8.71
f1 (ppm)

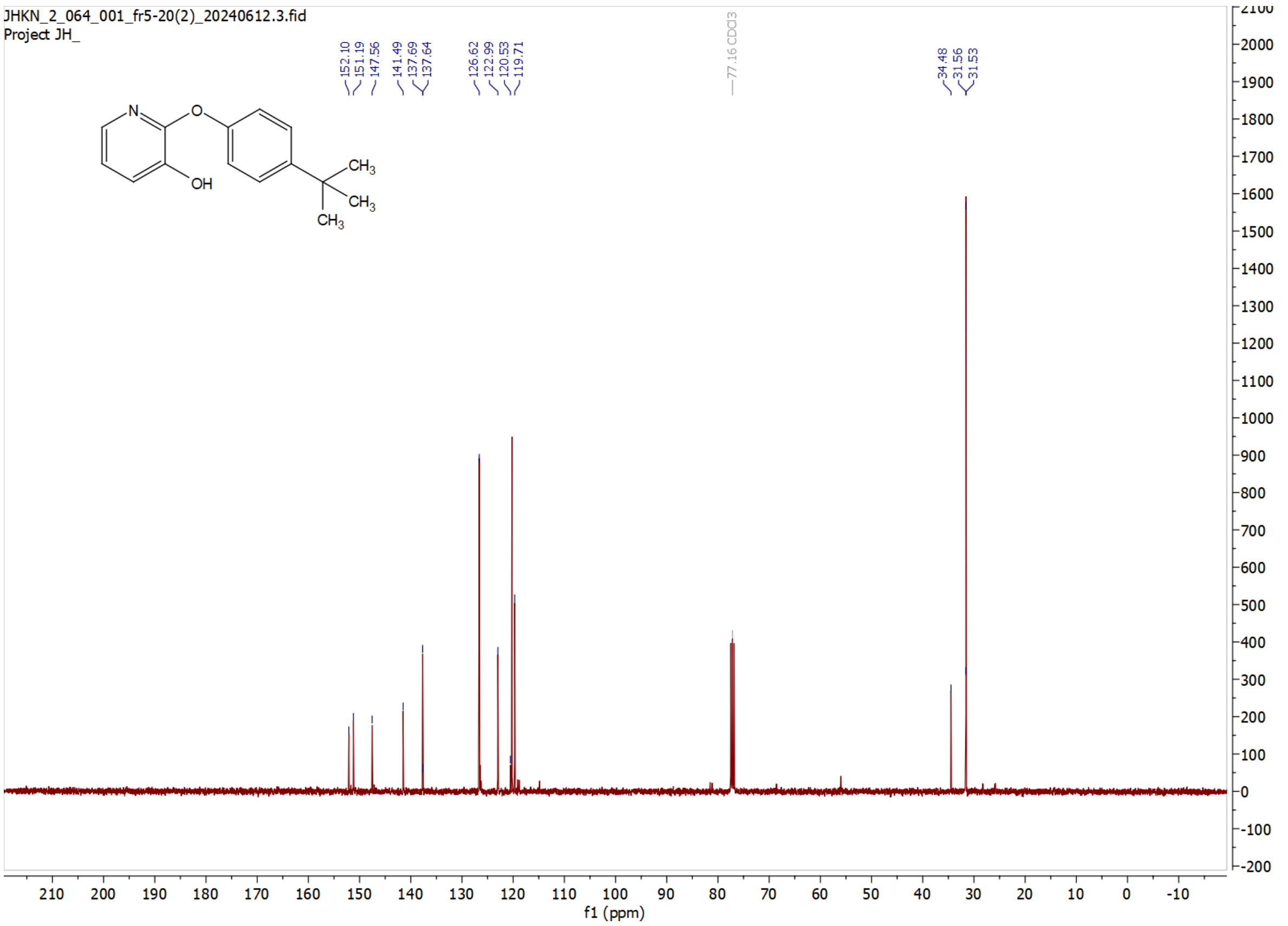

JHKN_2_064_001_fr5-20(2)_20240612.3.fid
Project JH_
152.10
151.19
147.56
141.49
137.69
137.64
126.62
122.99
120.53
119.71
77.16 CDCl3
34.48
31.56
31.53
f1 (ppm)

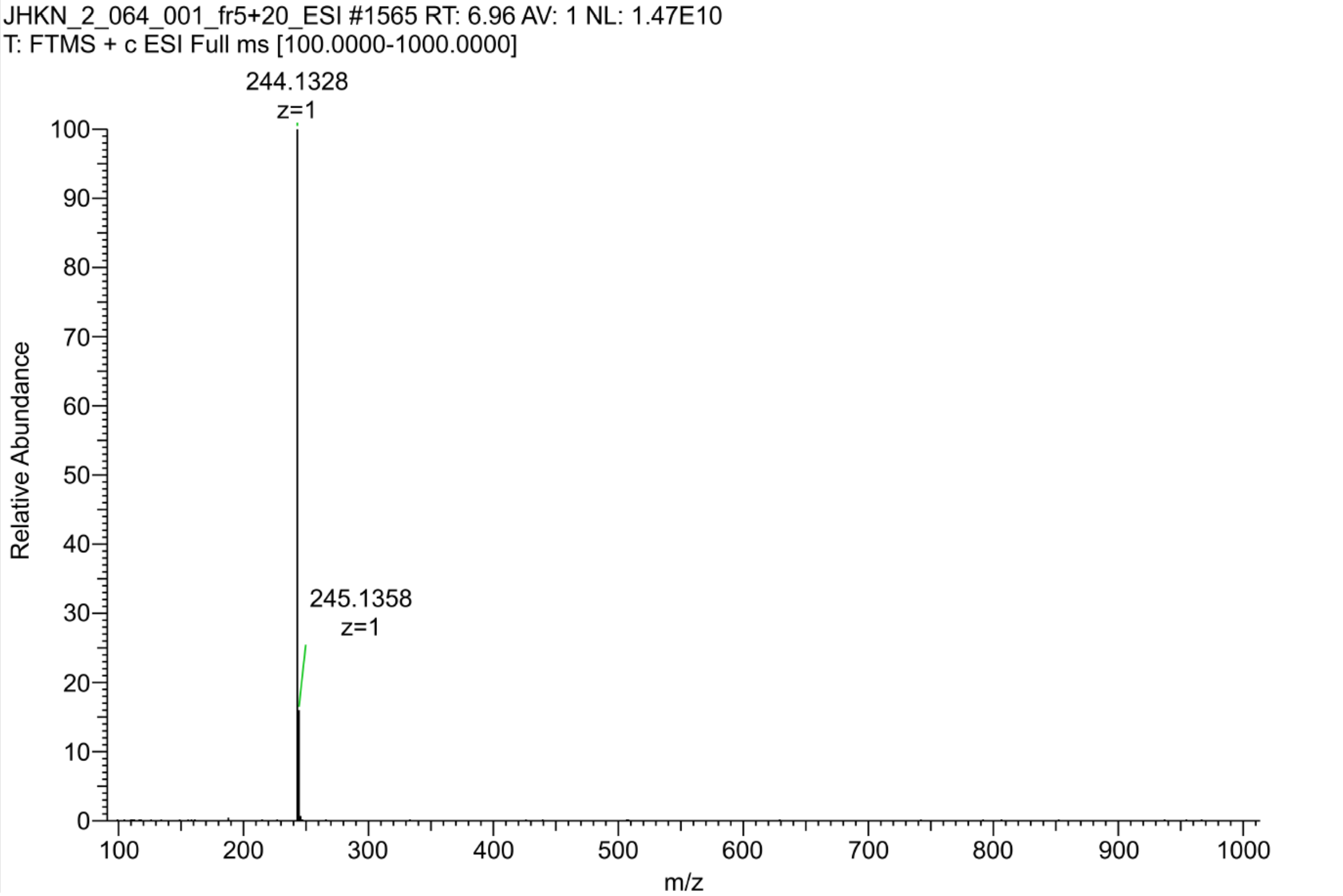

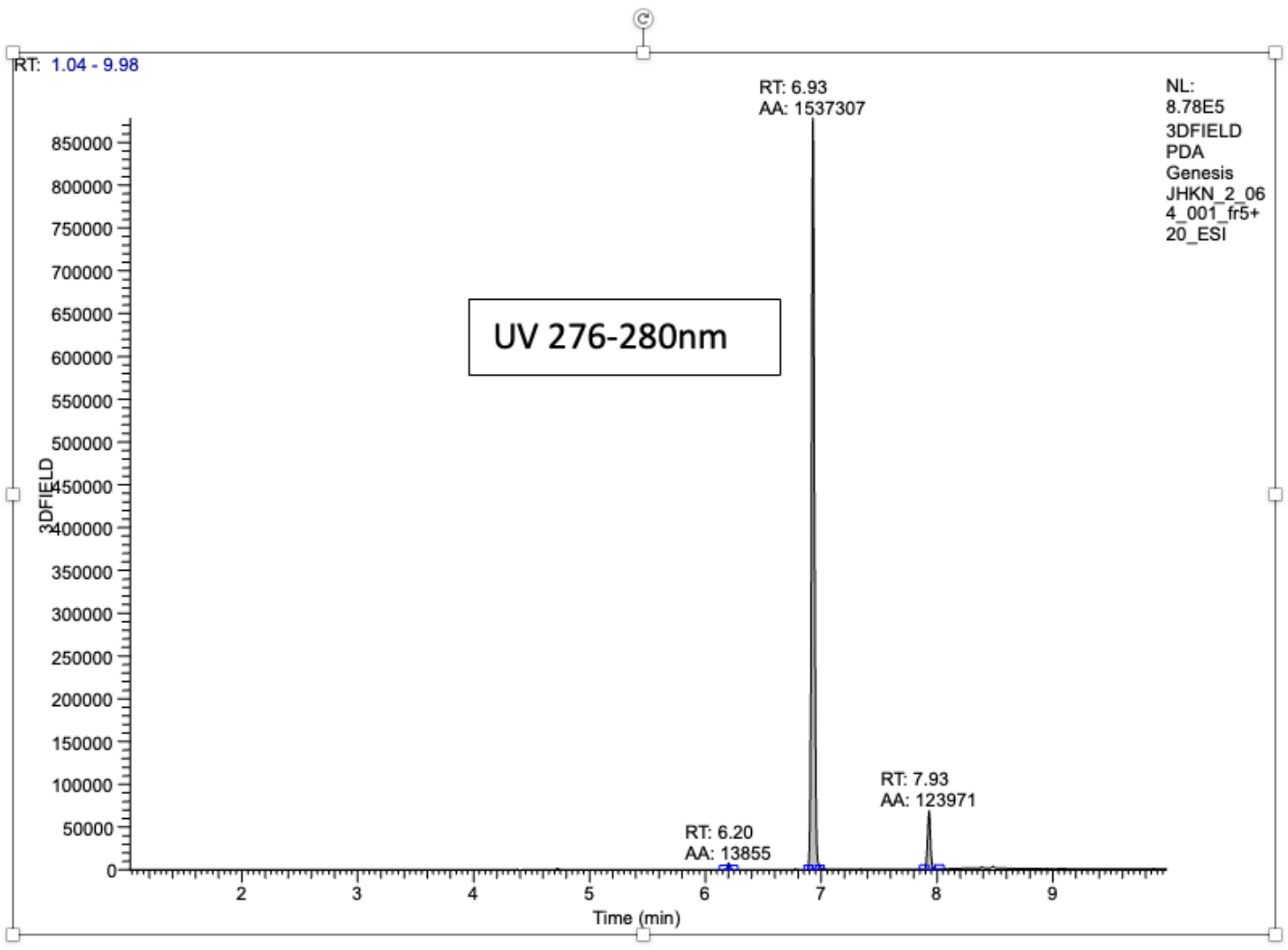

PEAK LIST

JHKN_2_064_001_fr5+20_ESI.raw

RT: 1.04 - 9.98

Number of detected peaks: 3

Apex RT	Start RT	End RT	Area	%Area	Height	%Height
6.20	6.16	6.24	13855.246	0.83	7607.513	0.80
6.93	6.89	6.99	1537306.979	91.77	877236.820	92.05
7.93	7.89	8.02	123971.391	7.40	68179.708	7.15

5. EU-OPENSCREEN primary screening

5.1 Experimental assay details copied from EU-OPENSCREEN.

C. albicans ATCC 64124 Anti-Fungal Assay

EOS300218

Screening of the 4th batch of the ACADEMIC Library (704 compounds) for 1 concentration (50 µM) with 2 replicates against C. albicans ATCC64124, according to "N8: Anti-Bacterial and Anti-Fungal Assays Bioprofiling Handbook" and following the protocols optimized during the validation step indicated in that proposal (Under the frame of the EU-OS bioprofiling tender process), to determine the antibacterial properties of these compounds against this human pathogen.
Briefly, compounds were tested in a liquid growth medium dispensed in 384 well plates inoculated with a standardized fungal suspension and after overnight incubation at 37ºC the plates were examined for bacterial growth as absorbance readout. The Genedata Screener software (Genedata, Inc., Basel, Switzerland) was used to process and analyze all the data from the screen. The reproducibility and sensitivity were supported by the statistical values derived from all the experiments performed. The activity of the compounds was expressed as a percentage of growth inhibition where 100% represented the inhibition growth of the target microorganism and 0% represented the total growth of target microorganism on the assay.

Assay setup

**Assay stage**	primary assay
**Bioassay**	cell growth assay
**Bioassay type**	functional phenotypic
**Bioassay setting**	in vitro
**Assay organism**	Candida albicans
**BAO tags**	high throughput screening, research institute, researcher, screening lab investigator, antifungal drug
**Screening site**	Fundación MEDINA - Screening and target validation, Microbiology and Chemistry
**Embargo until**	May 19, 2028

Detection method

**Physical detection method**	absorbance
**Detection instrument**	EnVision Multilabel Reader

Target

**Target name**	Organism Candida albicans
**Target type**	Organism
**Organism specification**	

Activities

**Concentration unit**	micromolar
**Activity determination method**	"Value" column is the activity of the compound calculated as an average of the percentage of inhibition. "Active" is defined as a value equal or greater than 70 (≥ 70). "Inconclusive" is defined as a value less than 70 (< 70) and equal or greater than 50 (≥ 50). "Inactive" is defined as a value less than 50 (< 50). A column with the calculated value of the standard deviation has been added to roughly estimate the reliability of the data .

MSSA ATCC 29213 Anti-Bacterial Assay

EOS300212

Screening of the 4rd batch of the Academic Library (704 compounds) for 1 concentration (50 µM) with 2 replicates against methicillin-susceptible Staphylococcus aureus ATCC29213, according to "N8: Anti-Bacterial and Anti-Fungal Assays Bioprofiling Handbook" and following the protocols optimized during the validation step indicated in that proposal (Under the frame of the EU-OS bioprofiling tender process), to determine the antibacterial properties of these compounds against this human pathogen.
Briefly, compounds were tested in a liquid growth medium dispensed in 384 well plates inoculated with a standardized bacterial suspension (CFU/mL) and after overnight incubation at 37ºC the plates were examined for bacterial growth as absorbance readout. The Genedata Screener software (Genedata, Inc., Basel, Switzerland) was used to process and analyze all the data from the screen. The reproducibility and sensitivity were supported by the statistical values derived from all the experiments performed. The activity of the compounds was expressed as a percentage of growth inhibition where 100% represented the inhibition growth of the target microorganism and 0% represented the total growth of target microorganism on the assay.

Assay setup

**Assay stage**	primary assay
**Bioassay**	cell growth assay
**Bioassay type**	functional phenotypic
**Bioassay setting**	in vitro
**Assay organism**	Staphylococcus aureus
**BAO tags**	high throughput screening, bacterial infectious disease, research institute, researcher, antibacterial drug, screening lab investigator
**Screening site**	Fundación MEDINA - Screening and target validation, Microbiology and Chemistry
**Embargo until**	May 19, 2028

Detection method

**Physical detection method**	absorbance
**Detection instrument**	EnVision Multilabel Reader

Target

**Target name**	Organism Staphylococcus aureus
**Target type**	Organism
**Organism specification**	

Activities

**Concentration unit**	micromolar
**Activity determination method**	"Value" column is the activity of the compound calculated as an average of the percentage of inhibition. "Active" is defined as a value equal or greater than 70 (≥ 70). "Inconclusive" is defined as a value less than 70 (< 70) and equal or greater than 50 (≥ 50). "Inactive" is defined as a value less than 50 (< 50). A column with the calculated value of the standard deviation has been added to roughly estimate the reliability of the data .

C. auris DSM21092 Anti-Fungal Assay

EOS300216

Screening of the 4th batch of the Academic Library (704 compounds) for 1 concentration (50 µM) with 2 replicates against C. auris DSM21092, according to "N8: Anti-Bacterial and Anti-Fungal Assays Bioprofiling Handbook" and following the protocols optimized during the validation step indicated in that proposal (Under the frame of the EU-OS bioprofiling tender process), to determine the antifungal properties of these compounds against this human pathogen.
Briefly, compounds were tested in a liquid growth medium dispensed in 384 well plates inoculated with a standardized fungal suspension and after overnight incubation at 37ºC the plates were examined for fungal growth as absorbance readout. The Genedata Screener software (Genedata, Inc., Basel, Switzerland) was used to process and analyze all the data from the screen. The reproducibility and sensitivity were supported by the statistical values derived from all the experiments performed. The activity of the compounds was expressed as a percentage of growth inhibition where 100% represented the inhibition growth of the target microorganism and 0% represented the total growth of target microorganism on the assay.

Assay setup

Assay stage	primary assay
Bioassay	cell growth assay
Bioassay type	functional phenotypic
Bioassay setting	in vitro
Assay organism	[Candida] auris
BAO tags	high throughput screening, research institute, researcher, screening lab investigator, antifungal drug
Screening site	Fundación MEDINA - Screening and target validation, Microbiology and Chemistry
Embargo until	May 19, 2028

Detection method

Physical detection method	absorbance
Detection instrument	EnVision Multilabel Reader

Target

Target name	Organism [Candida] auris
Target type	Organism
Organism specification	

Activities

Concentration unit	micromolar
Activity determination method	"Value" column is the activity of the compound calculated as an average of the percentage of inhibition. "Active" is defined as a value equal or greater than 70 (≥ 70). "Inconclusive" is defined as a value less than 70 (< 70) and equal or greater than 50 (≥ 50). "Inactive" is defined as a value less than 50 (< 50). A column with the calculated value of the standard deviation has been added to roughly estimate the reliability of the data .

E. faecalis ATCC 29212 Anti-Bacterial Assay

EOS300214

Screening of the 4rd batch of the Academic Library (704 compounds) for 1 concentration (50 µM) with 2 replicates against Enterococcus faecalis ATCC29212, according to "N8: Anti-Bacterial and Anti-Fungal Assays Bioprofiling Handbook" and following the protocols optimized during the validation step indicated in that proposal (Under the frame of the EU-OS bioprofiling tender process), to determine the antibacterial properties of these compounds against this human pathogen.
Briefly, compounds were tested in a liquid growth medium dispensed in 384 well plates inoculated with a standardized bacterial suspension (CFU/mL) and after overnight incubation at 37ºC the plates were examined for bacterial growth as absorbance readout. The Genedata Screener software (Genedata, Inc., Basel, Switzerland) was used to process and analyze all the data from the screen. The reproducibility and sensitivity were supported by the statistical values derived from all the experiments performed. The activity of the compounds was expressed as a percentage of growth inhibition where 100% represented the inhibition growth of the target microorganism and 0% represented the total growth of target microorganism on the assay.

Assay setup

Assay stage	primary assay
Bioassay	cell growth assay
Bioassay type	functional phenotypic
Bioassay setting	in vitro
Assay organism	Enterococcus faecalis
BAO tags	high throughput screening, bacterial infectious disease, research institute, researcher, antibacterial drug, screening lab investigator
Screening site	Fundación MEDINA - Screening and target validation, Microbiology and Chemistry
Embargo until	May 19, 2028

Detection method

Physical detection method	absorbance
Detection instrument	EnVision Multilabel Reader

Target

Target name	Organism Enterococcus faecalis ATCC 29212
Target type	Organism
Organism specification	

Activities

Concentration unit	micromolar
Activity determination method	"Value" column is the activity of the compound calculated as an average of the percentage of inhibition. "Active" is defined as a value equal or greater than 70 (≥ 70). "Inconclusive" is defined as a value less than 70 (< 70) and equal or greater than 50 (≥ 50). "Inactive" is defined as a value less than 50 (< 50). A column with the calculated value of the standard deviation has been added to roughly estimate the reliability of the data .

A. fumigatus ATCC 46645 Anti-Fungal Assay

EOS300219

Screening of the 4th batch of the ACADEMIC Library (704 compounds) for 1 concentration (50 µM) with 2 replicates against A. fumigatus ATCC46645, according to "N8: Anti-Bacterial and Anti-Fungal Assays Bioprofiling Handbook" and following the protocols optimized during the validation step indicated in that proposal (Under the frame of the EU-OS bioprofiling tender process), to determine the antifungal properties of these compounds against this human pathogen.
Briefly, compounds were tested in a liquid growth medium dispensed in 384 well plates inoculated with a standardized fungal suspension and after overnight incubation at 37ºC the plates were examined for fungal growth as fluorescence readout. The Genedata Screener software (Genedata, Inc., Basel, Switzerland) was used to process and analyze all the data from the screen. The reproducibility and sensitivity were supported by the statistical values derived from all the experiments performed. The activity of the compounds was expressed as a percentage of growth inhibition where 100% represented the inhibition growth of the target microorganism and 0% represented the total growth of target microorganism on the assay.

Assay setup

Assay stage	primary assay
Bioassay	cell growth assay
Bioassay type	functional phenotypic
Bioassay setting	in vitro
Assay organism	Aspergillus fumigatus
BAO tags	high throughput screening, research institute, researcher, screening lab investigator, antifungal drug
Screening site	Fundación MEDINA - Screening and target validation, Microbiology and Chemistry
Embargo until	May 19, 2028

Detection method

Physical detection method	fluorescence method
Detection instrument	EnVision Multilabel Reader

Target

Target name	Organism Aspergillus fumigatus
Target type	Organism
Organism specification	

Activities

Concentration unit	micromolar
Activity determination method	"Value" column is the activity of the compound calculated as an average of the percentage of inhibition. "Active" is defined as a value equal or greater than 70 (≥ 70). "Inconclusive" is defined as a value less than 70 (< 70) and equal or greater than 50 (≥ 50). "Inactive" is defined as a value less than 50 (< 50). A column with the calculated value of the standard deviation has been added to roughly estimate the reliability of the data .

Growth inhibition of Acinetobacter baumanii - academic batch 4 and 5

EOS300243

Acinetobacter baumanii, DSM30007, was cultivated in TSY in 384 well plates. 250nL of 10 mM solutions of each compound in DMSO were transferred with the acoustic dispenser Echo 550 to each well. The cultivation volume was 60 µL, plates were incubated at 37°C for 24h. The optical density at 600nm was determined and used as measure for bacterial growth. Data from control wells with medium only were used as 100% inhibition, and data from control wells with bacteria only were set as 0% growth inhibition. 50% growth inhibition was chosen as threshold to identify active compounds.

Assay setup

Assay stage	primary assay
Bioassay	cell growth assay
Bioassay type	functional phenotypic
Bioassay setting	in vitro
Assay organism	Acinetobacter baumannii
BAO tags	
Screening site	Helmholtz Centre for Infection Research - Department of Chemical Biology
Embargo until	Aug. 12, 2028

Detection method

Physical detection method	absorbance
Detection instrument	Synergy HT Multi-Mode Reader (BioTek)

Target

Target name	Organism Acinetobacter baumannii
Target type	Organism
Organism specification	

Activities

Concentration unit	micromolar
Activity determination method	primary data are single point measurements, and activity is defined as at least 50% growth inhibition after 24h.

Growth inhibition Pseudomonas aeruginosa - academic batch 4 and 5

EOS300241

Compound plates were prepared using the acoustic dispenser Echo 550 to transfer 250 nL into each well of a 384 well plate. Assay volume was 60 µL of the bacterial suspension, which was prepared from an overnight culture. Plates were incubated at 37°C for 24h. Growth was followed via determination of the optical density at 600 nm. The values resulting from 24h were analysed.

Assay setup

Assay stage	primary assay
Bioassay	cell growth assay
Bioassay type	functional phenotypic
Bioassay setting	in vitro
Assay organism	Pseudomonas aeruginosa group
BAO tags	
Screening site	Helmholtz Centre for Infection Research - Department of Chemical Biology
Embargo until	Aug. 12, 2028

Detection method

Physical detection method	absorbance
Detection instrument	Synergy HT Multi-Mode Reader (BioTek)

Target

Target name	Organism Pseudomonas aeruginosa
Target type	Organism
Organism specification	

Activities

Concentration unit	micromolar
Activity determination method	Optical density was normalized as growth inhibition with respect to control samples. Compounds are active, when they resulted in more than 50% growth inhibition.

Growth inhibition of Klebsiella pneumonia - academic batch 4 and 5

EOS300242

Klebsiella pneumonia, DSM681 = ATCC 10031, was cultivated in MHB in 384 well plates. 250nL of 10 mM solutions of each compound in DMSO were transferred with the acoustic dispenser Echo 550 to each well. The cultivation volume was 60 µL, plates were incubated at 37°C for 24h. The optical density at 600nm was determined and used as measure for bacterial growth. Data from control wells with medium only were used as 100% inhibition, and data from control wells with bacteria only were set as 0% growth inhibition. 50% growth inhibition was chosen as threshold to identify active compounds.

Assay setup

Assay stage	primary assay
Bioassay	cell growth assay
Bioassay type	functional phenotypic
Bioassay setting	in vitro
Assay organism	Klebsiella pneumoniae
BAO tags	
Screening site	Helmholtz Centre for Infection Research - Department of Chemical Biology
Embargo until	Aug. 12, 2028

Detection method

Physical detection method	absorbance
Detection instrument	Synergy HT Multi-Mode Reader (BioTek)

Target

Target name	Organism Klebsiella pneumoniae
Target type	Organism
Organism specification	

Activities

Concentration unit	micromolar
Activity determination method	primary data are single point measurements, and activity is defined as at least 50% growth inhibition after 24h.

Inhibition of Escherichia coli - academic batch 4 and 5

EOS300244

The growth inhibitory potential of compounds for E.coli ATCC 25922 is detected via the turbidity of the solutions at 600 nm (OD600).

Assay setup

Assay stage	primary assay
Bioassay	cell growth assay
Bioassay type	functional phenotypic
Bioassay setting	in vitro
Assay organism	Escherichia coli
BAO tags	
Screening site	Helmholtz Centre for Infection Research - Department of Chemical Biology
Embargo until	Aug. 12, 2028

Detection method

Physical detection method	transmittance
Detection instrument	Synergy HT Multi-Mode Reader (BioTek)

Target

Target name	Organism Escherichia coli
Target type	Organism
Organism specification	

Activities

Concentration unit	micromolar
Activity determination method	Data are normalised with respect to controls as % growth inhibition. Compounds are defined as active, when the growth is inhibited to more than 50%.

Academic Compound Collection (EUACC Batch 04) cell viability ATP quantification assay with HepG2 cells

EOS300246

Plate type: 384-well plate Corning 3764 Black clear bottom.
750 cells /well seeded into the plate.
Cell culture medium: DMEM (1.0 g/L glucose, without L-Glutamine, Lonza 12-707F), 10% FBS (Gibco 10270106), 2 mM L-glutamine (Lonza BE17-605E), 2% Pen/Strep (Lonza DE17-602E), + 1% NEAA (Gibco 11140-035)
48 hour incubation time.
Cell viability ATP quantification assay with HepG2 cells. Assay kit: CellTiter-Glo® 2.0 Cell Viability Assay - Promega Corporation.
15uL CellTiter-Glo/well added.
Screening concentration was 10 uM. Luminescence was detected using Pherastar FS reader. Data was normalized to the low (10 uM Sepantronium bromide - YM155) and high controls (25 nL/well Dimethyl Sulfoxide - DMSO) to generate percent inhibition values. The percent inhibition values uploaded.

Assay setup

Assay stage	primary assay
Bioassay	cell viability ATP quantitation assay
Bioassay type	functional
Bioassay setting	in vitro
Assay organism	Homo sapiens
BAO tags	time unit, hour, screening concentration, normalized data
Screening site	Institute for Molecular Medicine Finland - High Throughput Biomedicine Unit
Embargo until	Feb. 12, 2026

Detection method

Physical detection method	luminescence method
Detection instrument	BMG PHERAstar FS plate reader (BMG Labtech, Cary NC)

Target

Target name	Cell line permanent Hep-G2
Target type	Cell line
Cell-line type	Permanent

Activities

Concentration unit	nanomolar
Activity determination method	The compound was selected for validation if the average Percent Inhibition value (at 10 uM) was equal or greater than 30% in the primary screening (52 compounds were defined as active)

5.2 Results from primary screening at EU-OPENSCREEN.

See section 5.1 for definitions of activity and detection methods for the various assays.

Compound no	A. fumigatus ATCC 46645<br>Anti-Fungal Assay	C. albicans ATCC 64124<br>Anti-Fungal Assay	C. auris DSM21092<br>Anti-Fungal Assay	E. faecalis ATCC 29212<br>Anti-Bacterial Assay	MSSA ATCC 29213<br>Anti-Bacterial Assay
**1a**	-30.414 (inactive)	-5.040 (inactive)	-5.040 (inactive)	0.891 (inactive)	7.212 (inactive)
**1b**	-25.341 (inactive)	6.097 (inactive)	6.097 (inactive)	-12.770 (inactive)	5.981 (inactive)
**1c**	-12.012 (inactive)	-1.726 (inactive)	-1.726 (inactive)	-4.528 (inactive)	-1.680 (inactive)
**1d**	-17.934 (inactive)	19.306 (inactive)	19.306 (inactive)	-11.679 (inactive)	1.174 (inactive)
**1e**	-27.553 (inactive)	-2.922 (inactive)	-2.922 (inactive)	-15.618 (inactive)	0.036 (inactive)
**1f**	-27.582 (inactive)	21.381 (inactive)	21.381 (inactive)	-20.856 (inactive)	12.468 (inactive)
**1g**	-0.591 (inactive)	43.389 (inactive)	43.389 (inactive)	-15.254 (inactive)	-4.398 (inactive)
**1h**	19.684 (inactive)	36.084 (inactive)	36.084 (inactive)	-24.423 (inactive)	7.261 (inactive)
**1i**	3.863 (inactive)	32.090 (inactive)	32.090 (inactive)	-18.354 (inactive)	3.674 (inactive)
**1j**	-35.151 (inactive)	1.705 (inactive)	1.705 (inactive)	-12.675 (inactive)	2.423 (inactive)
**1k**	5.516 (inactive)	51.225 (inconclusive)	51.225 (inconclusive)	-27.574 (inactive)	0.037 (inactive)
**1l**	11.124 (inactive)	34.808 (inactive)	34.808 (inactive)	-15.860 (inactive)	-1.966 (inactive)
**2a**	20.082 (inactive)	55.478 (inconclusive)	55.478 (inconclusive)	-10.277 (inactive)	0.918 (inactive)
**2b**	-35.298 (inactive)	-5.515 (inactive)	-5.515 (inactive)	-14.804 (inactive)	2.505 (inactive)
**2c**	-16.747 (inactive)	5.710 (inactive)	5.710 (inactive)	-17.021 (inactive)	-12.860 (inactive)
**2d**	-34.405 (inactive)	5.822 (inactive)	5.822 (inactive)	-14.354 (inactive)	3.512 (inactive)
**2e**	-30.508 (inactive)	20.712 (inactive)	20.712 (inactive)	-13.592 (inactive)	-0.492 (inactive)
**2f**	-6.520 (inactive)	24.229 (inactive)	24.229 (inactive)	-11.021 (inactive)	-13.023 (inactive)
**3a**	-34.215 (inactive)	8.657 (inactive)	8.657 (inactive)	-15.038 (inactive)	5.500 (inactive)
**3b**	-18.311 (inactive)	30.519 (inactive)	30.519 (inactive)	-19.808 (inactive)	-2.446 (inactive)
**4**	-16.316 (inactive)	21.213 (inactive)	21.213 (inactive)	-20.527 (inactive)	4.683 (inactive)
**5**	-26.582 (inactive)	-4.311 (inactive)	-4.311 (inactive)	0.476 (inactive)	8.319 (inactive)

Compound no	Growth inhibition of Acinetobacter baumanii	Growth inhibition Pseudomonas aeruginosa	Growth inhibition of Klebsiella pneumonia	Inhibition of Escherichia coli	(EUACC Batch 04) cell viability ATP quantification assay with HepG2 cells
**1a**	0.891 (inactive)	-3.152 (inactive)	-10.713 (inactive)	-3.359 (inactive)	nd
**1b**	-12.770 (inactive)	0.674 (inactive)	-11.110 (inactive)	-2.626 (inactive)	11.6 (inactive)
**1c**	-4.528 (inactive)	6.065 (inactive)	-20.369 (inactive)	-3.033 (inactive)	nd
**1d**	-11.679 (inactive)	3.891 (inactive)	-5.555 (inactive)	-1.812 (inactive)	nd
**1e**	-15.618 (inactive)	1.630 (inactive)	-9.655 (inactive)	-3.196 (inactive)	-5.4 (inactive)
**1f**	-20.856 (inactive)	8.239 (inactive)	-2.778 (inactive)	-1.893 (inactive)	-1.6 (inactive)
**1g**	-15.254 (inactive)	8.500 (inactive)	-11.772 (inactive)	-0.428 (inactive)	nd
**1h**	-24.423 (inactive)	4.065 (inactive)	-3.836 (inactive)	-6.209 (inactive)	nd
**1i**	-18.354 (inactive)	5.804 (inactive)	-14.285 (inactive)	0.061 (inactive)	5.1 (inactive)
**1j**	-12.675 (inactive)	-0.370 (inactive)	-2.645 (inactive)	-4.255 (inactive)	nd
**1k**	-1.190 (inactive)	-1.649 (inactive)	-1.19 (inactive)	-1.65 (inactive)	nd
**1l**	-15.860 (inactive)	-0.283 (inactive)	-4.497 (inactive)	-1.812 (inactive)	-2.4 (inactive)
**2a**	-10.277 (inactive)	5.109 (inactive)	-13.359 (inactive)	-3.278 (inactive)	nd
**2b**	-14.804 (inactive)	-5.065 (inactive)	-5.423 (inactive)	-4.743 (inactive)	nd
**2c**	-17.021 (inactive)	1.370 (inactive)	-8.333 (inactive)	-5.314 (inactive)	nd
**2d**	-14.354 (inactive)	-4.457 (inactive)	-5.423 (inactive)	-2.463 (inactive)	-12.2 (inactive)
**2e**	-13.592 (inactive)	6.935 (inactive)	-3.968 (inactive)	-1.242 (inactive)	nd
**2f**	-11.021 (inactive)	11.109 (inactive)	-15.078 (inactive)	-0.509 (inactive)	nd
**3a**	-15.038 (inactive)	-3.587 (inactive)	0.397 (inactive)	-4.173 (inactive)	-4.8 (inactive)
**3b**	-19.808 (inactive)	8.239 (inactive)	-6.216 (inactive)	-1.649 (inactive)	13.3 (inactive)
**4**	-20.527 (inactive)	-3.500 (inactive)	-0.661 (inactive)	-6.372 (inactive)	nd
**5**	0.476 (inactive)	-2.022 (inactive)	-10.978 (inactive)	-2.871 (inactive)	0.6 (inactive)

nd = not determined

6. References

S. Bhilare, S. S. M. Bandaru, J. Shah, N. Chrysochos, C. Schulzke, Y. S. Sanghvi and A. R. Kapdi, *J. Org. Chem.*, 2018, **83**, 13088–13102.

M. M. Bradford, *Anal. Biochem.*, 1976, **72**, 248–254.

H. Kinuta, M. Tobisu and N. Chatani, *J. Am. Chem. Soc.*, 2015, **137**, 1593–1600.

G. Li, P. P. Gao, X. L. Lv, C. Qu, Q. K. Yan, Y. Wang, S. L. Yang and J. J. Wang, *Org. Lett.*, 2017, **19**, 2682–2685.

U. Lind, M. Alm Rosenblad, L. Hasselberg Frank, S. Falkbring, L. Brive, J. M. Laurila, K. Pohjanoksa, A. Vuorenpää, J. P. Kukkonen, L. Gunnarsson, M. Scheinin, L. G. E. Mårtensson Lindblad and A. Blomberg, *Mol. Pharmacol.*, 2010, **78**, 237–248.

A. T. Londregan, S. Jennings and L. Q. Wei, *Org. Lett.*, 2011, **13**, 1840–1843.

A. Marjamäki, S. Ala-Uotila, K. Luomala, M. Peräla, C. Jansson and M. Jalkanen, *Biochim. Biophys. Acta*, 1992, **1134**, 169–177.

A. Marjamäki, K. Luomala, S. Ala-Uotila and M. Scheinin, *Eur. J. Pharmacol*., 1993, **246**, 219–226.

J. M. Peltonen, M. Pihlavisto and M. Scheinin, *Eur. J. Pharmacol.*, 1998, **355**, 275–279

K. Pohjanoksa, C. C. Jansson, K. Luomala, A. Marjamäki, J. M. Savola and M. Scheinin, *Eur. J. Pharmacol.*, 1997, **335**, 53–63.

H. Y. Zeng, D. W. Cao, Z. H. Qiu and C. J. Li, *Angew. Chem. Int. Ed.,* 2018, **57**, 3752–3757.

Q. Zhang, D. Wang, X. Wang and K. Ding, *J. Org. Chem.*, 2009, **74,** 7187–7190.
